# Analysis of ESAFORM 2021 cup drawing benchmark of an Al alloy, critical factors for accuracy and efficiency of FE simulations

**DOI:** 10.1007/s12289-022-01672-w

**Published:** 2022-07-15

**Authors:** Anne Marie Habraken, Toros Arda Aksen, José L. Alves, Rui L. Amaral, Ehssen Betaieb, Nitin Chandola, Luca Corallo, Daniel J. Cruz, Laurent Duchêne, Bernd Engel, Emre Esener, Mehmet Firat, Peter Frohn-Sörensen, Jesús Galán-López, Hadi Ghiabakloo, Leo A. I. Kestens, Junhe Lian, Rakesh Lingam, Wencheng Liu, Jun Ma, Luís F. Menezes, Tuan Nguyen-Minh, Sara S. Miranda, Diogo M. Neto, André F. G. Pereira, Pedro A. Prates, Jonas Reuter, Benoit Revil-Baudard, Carlos Rojas-Ulloa, Bora Sener, Fuhui Shen, Albert Van Bael, Patricia Verleysen, Frederic Barlat, Oana Cazacu, Toshihiko Kuwabara, Augusto Lopes, Marta C. Oliveira, Abel D. Santos, Gabriela Vincze

**Affiliations:** 1 ArGEnCo dpt, MSM team, University of Liège, 9 Allée de la Découverte, 4000 Liège, Belgium; 2 Mechanical Engineering Department, University of Sakarya, Esentepe Campus, M7, 54050 Serdivan, Sakarya Turkey; 3 CMEMS, Microelectromechanical Systems Research Unit, University of Minho, Campus de Azurém, 4800-058 Guimarães, Portugal; 4 Institute of Science and Innovation in Mechanical and Industrial Engineering (INEGI), R. Dr. Roberto Frias 400, 4200-465 Porto, Portugal; 5 Department of Mechanical and Aerospace Engineering, University of Florida, REEF, 1350N. Poquito Rd, Shalimar, FL 32579 USA; 6 Department of Electromechanical, Systems and Metal Engineering, MST-DyMaLab Research Group, Ghent University, Technologiepark 46, 9052 Zwijnaarde, Belgium; 7 Forming Technology (UTS), Institute of Production Technologies, University of Siegen, Breite Strasse, 11, 57076 Siegen, Germany; 8 Mechanical Engineering Department, Bilecik Seyh Edebali University, Pelitozu, 11230 Bilecik, Turkey; 9 MSE, Faculty 3mE, Delft University of Technology, Mekelweg 2, 2628 CD Delft, The Netherlands; 10 Department of Materials Engineering, KU Leuven, Leuven, Belgium; 11 Department of Electromechanical, Systems and Metal Engineering, Ghent University, Ghent, Belgium; 12 Advanced Manufacturing and Materials, Department of Mechanical Engineering, Aalto University, Puumiehenkuja 3, 02150 Espoo, Finland; 13 Graduate Institute of Ferrous and Energy Materials Technology, Pohang University of Science and Technology, 77 Cheongam-ro, Nam-gu, Pohang, Gyeongbuk 37673 Republic of Korea; 14 Indian Institute of Technology Dharwad, P.B. Road, Dharwad, Karnataka 580011 India; 15 School of Civil Aviation, Northwestern Polytechnical University, Taicang, 215400 China; 16 Department of Mechanical and Industrial Engineering, Norwegian University of Science and Technology, 7491 Trondheim, Norway; 17 CEMMPRE, Department of Mechanical Engineering, University of Coimbra, Polo II, Pinhal de Marrocos, 3030-788 Coimbra, Portugal; 18 Center for Mechanical Technology and Automation (TEMA), Department of Mechanical Engineering, University of Aveiro, Aveiro, Portugal; 19 Mechanical Engineering Department, Yildiz Technical University, Yildiz Campus, Barbaros, 34349 Istanbul, Turkey; 20 Division of Advanced Mechanical Systems Engineering, Institute of Engineering, Tokyo University of Agriculture and Technology, 2-24-16 Nakacho, Koganei-shi, Tokyo, 184-8588 Japan; 21 Department of Materials and Ceramic Engineering, CICECO, Universidade de Aveiro, 3810-193 Aveiro, Portugal; 22 Faculty of Engineering, University of Porto, R. Dr. Roberto Frias 400, 4200-465 Porto, Portugal; 23 Steel Insititute, RWTH Aachen University, Intzestr. 1, 52072, Aachen, Germany

**Keywords:** Benchmark, 6016-T4 aluminium alloy, Deep drawing modelling, Model comparisons, Earing profile prediction, Force prediction, Thickness prediction

## Abstract

This article details the ESAFORM Benchmark 2021. The deep drawing cup of a 1 mm thick, AA 6016-T4 sheet with a strong cube texture was simulated by 11 teams relying on phenomenological or crystal plasticity approaches, using commercial or self-developed Finite Element (FE) codes, with solid, continuum or classical shell elements and different contact models. The material characterization (tensile tests, biaxial tensile tests, monotonic and reverse shear tests, EBSD measurements) and the cup forming steps were performed with care (redundancy of measurements). The Benchmark organizers identified some constitutive laws but each team could perform its own identification. The methodology to reach material data is systematically described as well as the final data set. The ability of the constitutive law and of the FE model to predict Lankford and yield stress in different directions is verified. Then, the simulation results such as the earing (number and average height and amplitude), the punch force evolution and thickness in the cup wall are evaluated and analysed. The CPU time, the manpower for each step as well as the required tests versus the final prediction accuracy of more than 20 FE simulations are commented. The article aims to guide students and engineers in their choice of a constitutive law (yield locus, hardening law or plasticity approach) and data set used in the identification, without neglecting the other FE features, such as software, explicit or implicit strategy, element type and contact model.

## Table of content

Abstract

**Introduction**

**Material experimental characterization**

     Material and initial texture

     Mechanical characterization tests

         Uniaxial tensile tests

         Biaxial tensile tests

         Monotonic and reverse simple shear tests

         EBSD measurements of the fully drawn cup of AA 6016-T4

**Cup forming and measurements**

     Experimental forming tools and process conditions

     Punch force

     Cup height and earing profile

     Cup thickness

     Measurement of tools and cup thickness by different methods

**Discussion about friction coefficient**

**Summary of features of FEM simulations**

**Main features of constitutive laws**

     Orthotropic yield functions

         2-D orthotropic yield functions (Yld89, Yld2000-2D, HomPol4, HomPol6)

         3-D orthotropic yield functions (Hill48, CB2001, Yld2004-18p, CPB06ex2, Caz2018-Orth)

     Crystal plasticity based constitutive models

         Facet-3D model linked to ALAMEL crystal plasticity model

         Caz2018polycrys, a polycrystalline model based on Cazacu single crystal law Caz2018singlecrys

         Minty model, an interpolation approach

         Crystal plasticity model used in DAMASK solver

         Viscoplastic Self Consistent Model (VPSC)

**Parameter identification and validation of constitutive models**

     Applied identification and validation methodology for crystal plasticity models

         Representative textures and microstructures

             Texture description for ALAMEL

             Texture description for Caz2018polycrys

             Texture description for Minty

             Texture description for Damask

             Texture description for VPSC model

             About the representative number of grains used to model the texture

         Parameter identification, validation and/or use of crystal plasticity models

             Facet-3D model

             Caz2018polycrys

             Minty law

             RVE Damask Simulations

             VPSC model

             Caz2018singlecrys

     Parameter identification and validation of phenomenological models

         Hill48 yield locus (associated law)

         4^th^ order polynomial model (HomPol4)

         6^th^ order polynomial model (HomPol6)

         Non quadratic plane-stress yield locus Barlat (Yld89)

         CB2001 by Cazacu and Barlat (2001)

         Yld2000-2D and Yld 2004-18p

         CPB06 criterion by Cazacu, Plunket and Barlat (2006)

         Caz2018-Orth or Cazacu (2018) orthotropic yield criterion

         Hill48 and non-associated flow rule

     Identification of hardening laws and data used

     Discussion on the methodology for identification of the models

**Cup drawing simulations, analysis and discussion**

     Results of phenomenological models identified with physical tests

         3-D orthotropic yield functions and solid or shell elements

            Hill48 with associated flow rule

            Hill48 with non-associated flow rule

            CB2001 yield criterion

            Yld2004-18p yield criterion

            CPB06ex2 yield criterion

            Caz2018-Orth yield criterion

         2-D orthotropic yield functions and solid or shell elements

            Yld89 yield criterion

            Yld2000-2D yield criterion

            HomPol4 and HolmPol6 yield criteria

            HomPol laws with isotropic hardening

            HomPol laws with isotropic and kinematic hardening

     Results of phenomenological models identified with physical and virtual crystal plasticity tests

         Yld2004-18p based on physical experiments and virtual tests with DAMASK

         Yld2000-2D based on virtual tests with VPSC

         Caz2018singlecrys assuming or not a pure cube texture

         Facet-3D based on ALAMEL virtual tests

     Results with crystal plasticity based constitutive models

         Caz2018polycrys based on Caz2018singlecrys

         Minty

     Summary of the results and discussion

**Conclusion**

**Acknowledgement**

Conflict of Interest

**References**

## Introduction

Why to launch a series of Benchmarks within European Scientific Association for material FORMing (ESAFORM) community? Still today, in the United States of America, the National Institute of Standards and Technology (NIST) founded in 1901 provides data for engineers and materials scientists to develop accurate simulations and processes. This fact demonstrates that benchmarking is a long-term need. Since 1986, NAFEMS provides sets of independent “standard” tests that can be applied to any Finite Element System. In the specific field of sheet forming, a 1^st^ congress with benchmark (the precursor of Numisheet series) called VDI 1991, in Zurich, gathered international teams eager to compare their results and to discuss them within a conference. Since the nineties, the Numisheet benchmarks are references in the sheet forming community. The analysis of the cylindrical cup forming was first addressed in the NUMISHEET’99 [36]: (i) Benchmark B1: Limiting drawing height of a cylindrical cup; (ii) Benchmark B2: Limiting drawing height of a cylindrical cup with hydraulic counter pressure [92]; and (iii) Benchmark C: Reverse deep drawing of a cylindrical cup [25]. These benchmarks were focused on predicting the strain distribution, including necking occurrence, in case of Benchmark B. In 2002, another benchmark involving a cylindrical cup was proposed, Benchmark Test A: Deep Drawing of a Cylindrical Cup. In this case, the aim was to evaluate the accuracy in predicting the earing profile, when considering a high blank holder force, and the wrinkling behaviour, for a low blank holder force [108]. In 2011, a case study was proposed addressing the influence of the anisotropic behaviour on the cylindrical cup height, Benchmark 1: Earing Evolution During Drawing and Ironing Processes [26]. The NUMISHEET 2014 also considered an example involving a cylindrical cup, focusing on the prediction of wrinkles, Benchmark 4 - Wrinkling during cup drawing [27]. In 2016, the cylindrical cup geometry was once again selected for a case study, entitled Benchmark 1: Failure Prediction after Cup Drawing, Reverse Redrawing and Expansion [105]. The challenges involving failure prediction when dealing with complex strain paths, lead to the selection of a similar example for the NUMISHEET 2020 (postponed to 2022, due to the COVID-19 pandemic). Meanwhile, in 2016, the case study (Benchmark 3: Springback of an Al-Mg alloy in warm forming conditions), focusing on the analysis of warm forming conditions also considered the forming of a cylindrical cup, in this case at different temperatures [68]. Finally, in 2018, the Benchmark 2: Cup drawing of an anisotropic thick steel sheet, considered different process conditions to evaluate the prediction ability of different forming defects, including springback, wrinkles and fracture during embossing [51].

The benchmark B1, performed under the NUMISHEET’99, had 5 participants doing the experimental tests [36], while 7 teams contributed with experimental results for benchmark A, of NUMISHEET 2002 [108]. Regarding the number of participants contributing with numerical simulation results, an average of around 10 was observed, with small fluctuations. The analysis of the data indicates that at the beginning there was an increase in the number of participating teams, accompanied by a decrease in the number of solvers, mainly due to the abandonment of some academic ones, but also to the merger of others. In addition, the increasing robustness of the numerical results made the dispersion in the experimental results, obtained by the various participants, more evident. The fact that the experimental range covers all numerical results disables a more rigorous analysis of the quality of the formulations and strategies adopted in the numerical models. Nevertheless, it should be noted that since 2002, there was only one team contributing with experimental results for the different benchmarks.

The approach adopted by the NUMISHEET conference series is that teams make a blind submission of their numerical results, which will be discussed in a public session during the conference. This approach is adopted taking into account that the previous knowledge of the experimental results can lead to “champion results”. Anyway, the commitment of the participants in the presentation of rigorous results can lead to the use of numerical parameters that distance the case studies from industrial practice [67]. Thus, the blind submission contributes to an interesting comparison of the approaches more commonly adopted by the different teams, but disables the possibility for a rigorous discussion about different numerical formulations and strategies, which requires a more careful analysis of the results, not possible within a conference public discussion.

ESAFORM association promotes applied research in University and Industry, spreads scientific information and develops education. These objectives explain why this new Benchmark series is launched. An ESAFORM Benchmark is not seen as a competitive event but as an opportunity to gather senior and young researchers to discuss and bring a state-of-the-art information about any scientific challenge related to material forming. The target of ESAFORM Benchmarks can be focused on any materials (polymers, composites, metals …), based on experimental work or applied simulations, software developments or forming process innovations, forming processes impact and sustainability, etc. The topic covered in 2021 by ESAFORM benchmark presents some overlapping with former case studies from Numisheet as reminded previously. However, the ESAFORM benchmarks target a broader spectrum than that covered by the Numisheet conferences. The specificity of ESAFORM benchmark is the intention to provide data, but also to exchange about how they are treated or collected. For instance within this article, the generation of data, the identification method of the material model parameters and the mandatory choices within the simulations (friction, element type, constitutive laws …) are analysed and published.

This state-of-the-art article constitutes a deliverable of the EXACT Benchmark (Experiment and Analysis of Aluminium Cup Drawing Test). For this first ESAFORM Benchmark edition, the ESAFORM board selected the proposal of a group of senior scientists either dedicated to numerical or experimental metal fields (*Frederic Barlat*, *Oana Cazacu*, *Anne Marie Habraken*, *Toshihiko Kuwabara, Augusto Lopes, Marta Oliveira*, *Abel Santos*, *Gabriela Vincze)****.*** They worked a large part of their career developing new yield locus formulations, crystal plasticity simulations, measuring textures, trying to validate sheet model predictions vs. experimental tests. However, they still need to interact among them and with young researchers to understand and analyse the advantages and drawbacks of the different constitutive models. This article deals with a strong cube texture aluminium sheet which enhances the challenge for the phenomenological yield loci as it generates interesting curvatures within the plastic surface description.

The Artificial Intelligence (AI) algorithms are now entering within material science problems [48, 77, 106, 107]. Sheet forming can benefit from these new approaches, saving computation time at various steps of the classical ‘old fashion’ way of simulations. However, Deep Learning methods cannot only be fed by experimental data. Therefore, more than ever, this review paper will help to develop FE models and to point where AI can help. Success stories are already present as for instance, [37] where Recurrent Neural Networks model the behaviour of AA5182 aluminium alloy and DC05 steel. The Deep Learning surrogate model predicts accurate results for arbitrary loading paths, after a training step based on FE simulation results. In this specific case, a Barlat Yld2000-2D yield locus coupled with Homogeneous Anisotropic Hardening was selected to model the material behaviour. So, let us be prepared for these new approaches.

Hereafter, the article tries to answer interrogations such as: is the final discrepancy between experimental and numerical results related to measurement errors, model inaccuracies or phenomena forgotten in the simulation? Which model to choose under time constraint or lack of data? After all these years of debates about solid, shell, solid-shell elements [1, 18, 53, 79], constitutive laws [6, 12, 19, 41, 46, 54, 72, 84, 100], after all the Numisheet Benchmarks dedicated to folding, deep drawing or incremental forming etc. of different steel, aluminium, magnesium grades, what can be added?

The current article gathers information allowing industries and young researchers to easily select a rheological model as well as perhaps a multiscale modelling strategy. For instance, the interest of the identification of macroscopic material parameters based on microscopic computations is presented vs. the classical approach (tensile tests and phenomenological laws). Virtual tests are described, relying on crystal plasticity and different representative volume elements. However, these advanced approaches are simpler than the one proposed by [69] where not only the grain behaviour but also the grain boundaries are taken into account. Within the sheet forming simulation process, the constitutive law is not the only key feature. The finite element type (shell, solid-shell, solid element), the mesh refinement and the contact models are parts of the finite element simulation accuracy. These choices are however not the main focus of this benchmark, even if they are somehow included in the discussion.

The ESAFORM Benchmark organizing team has attracted many other colleagues within this Benchmark adventure and Table 1 provides the acronyms used hereafter for their institutions.

**Table 1:** The participants and the organizers of ESAFORM 2021 Benchmark, their affiliation and single acronym for each group

Team members affiliation	Acronym
Katholieke Universiteit Leuven and Ghent University (Belgium) Hadi Ghiabakloo, Albert Van Bael (Leuven), Tuan Nguyen-Minh, Leo A.I. Kestens (Ghent)	KUL
Norwegian University of Science and Technology (Norway) and Northwestern Polytechnical University (China) Jun Ma (Trondheim), Wencheng Liu (Taicang)	NTNU
Pohang University of Science and Technology and Indian Institute of Technology Dharwad (Korea) Rakesh Lingam, Frederic Barlat (Pohang)	POSTECH
University of Florida (US) Benoit Revil-Baudard, Oana Cazacu, Nitin Chandola (Shalimar)	REEF
Aalto University (Finland) Junhe Lian, Fuhui Shen (Espoo)	UAalto
University of Coimbra and University of Minho (Portugal) Marta C. Oliveira, André F. G. Pereira, Pedro A. Prates, Diogo M. Neto, Luís F. Menezes (Coimbra), José L. Alves (Minho)	UCoimbra
Ghent University (Belgium) and Delft University of Technology (Netherlands) Luca Corallo, Patricia Verleysen (Ghent), Jesus Galan Lopez (Delft)	UGent
University of Liege (Belgium) Ehssen Betaieb, Carlos Rojas, Laurent Dûchene, Anne Marie Habraken (Liege)	ULiege
University of Porto (Portugal) Rui L. Amaral, Daniel J. Cruz, Sara S. Miranda, Abel D. Santos (Porto)	UPorto
University of Sakarya, Bilecik Seyh Edebali University and Yildiz Technical University (Turkey) Bora Sener (Yildiz), Emre Esener (Bilecik), Toros Arda Aksen, Mehmet Firat (Sakarya)	USakarya
University of Siegen (Germany) Jonas Reuter, Bernd Engel, Peter Frohn-Sörensen (Siegen)	USiegen
University of Aveiro Gabriela Vincze, Augusto Lopes (Aveiro)	UA
Tokyo University of Agriculture and Technology Toshihoko Kuwabara (Tokyo)	TUAT

As explained above, this ESAFORM 2021 Benchmark article offers a holistic story, from the data generation to the final simulation validations:

1. the material characterization behaviour by macroscopic classical mechanical tests (tensile tests, monotonic and reverse simple shear tests, biaxial tensions) is the result of the collaboration of 3 laboratories that duplicated some tests (“Mechanical characterization tests” Section);
2. the analysis of the initial and updated textures by EBSD maps (“Material and initial texture” and “EBSD measurements of the fully drawn cup of AA 6016-T4” Sections);
3. the cup forming process description as well as the measurement techniques used to characterize earing profile and thickness evolution (“Cup forming and measurements” Section);
4. some considerations about friction and how it affects simulation results (“Discussion about friction coefficient” Section);
5. the summary of the simulation features performed by the Benchmark participants (“Summary of features of FEM simulations” Section);
6. a short description of all the constitutive laws used, either phenomenological ones or based on crystal plasticity (“Main features of constitutive laws” Section);
7. the clear identification methodology followed to reach the material parameter sets for each constitutive model as well as a model validation step through the predictions of Lankford coefficient and the evolution of initial yield limit with tensile directions (“Parameter identification and validation of constitutive models” Section);
8. the comparisons between the FE predictions (cup average height, number of ears and their average amplitude, thickness in the cup wall and punch force) vs. the experimental results and their analysis (“Cup drawing simulations, analysis and discussion” Section).

The ratio between the Central Processing Unit (CPU) time, the results accuracy as well as the ratio between the engineering time, to prepare and post process the data, vs. the confidence in the results are also commented. The “Conclusion” Section summarizes the interesting points emerging from this Benchmark study.

## Material experimental characterization

### Material and initial texture

**Fig. 1:**
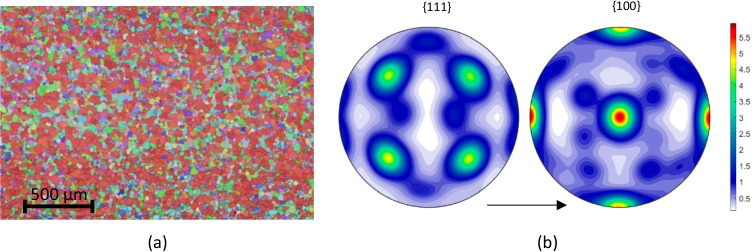
Initial texture of the 6016-T4 aluminium alloy: **a** EBSD map; **b** {111} and {100} pole figures; the arrow indicates the rolling direction (RD)

The material studied in this work is an aluminium alloy 6016-T4 produced by the UACJ Co., Japan and supplied in a sheet form with 1 mm thickness. A Bruker CrystAlign QC 400 EBSD system interfaced to a Hitachi SU-70 SEM was employed in UA to map the crystallographic orientations of the grains. From the EBSD raw data (Fig. 1(a)), a set of 1000 orientations representing the crystallographic texture were extracted using the MTEX Matlab Toolbox [3]. The pole figures are shown in Fig. 1(b) and the main texture components present in the microstructure are given in Table 2, where *ϕ*_1_, Φ, *ϕ*_2_ are the Euler angles (Bunge convention). Considering a misorientation of 3°, it is obtained that 52% volume fraction of grains are spread around the {100}<001 > orientation (cube component). This material selection with a strong cube texture enhancing anisotropy is seen as an ideal example to test the capability of the constitutive models. Note also that the material presents equiaxed grains with an average size of around 50 μm.

**Table 2:** The volume fractions for the main texture components for the AA 6016-T4 initial sheet

$\phi_{1\;}\lbrack^{\circ}\rbrack$	Φ [°]	$\phi_2\;\lbrack^{\circ}\rbrack$	Volume
204.96	0.05885	155.608	52%
330.862	56.1717	72.9679	16%
238.861	35.9114	156.439	13%
210.481	58.1962	108.63	11%
300.531	34.0291	27.1024	8%

### Mechanical characterization tests

The material has been characterized in 2018 at Tokyo University of Agriculture and Technology (TUAT) based on uniaxial tensile and biaxial tensile tests on flat specimens and multiaxial tube expansion tests. Sheets from the same batch were provided for this benchmark, which enabled to further investigate the mechanical response for other loading paths, by performing simple and reverse shear tests and conducting additional cup drawing tests. Four laboratories have been involved in the mechanical characterization campaign conducted in 2020, which was aimed at: (i) generating complementary data and (ii) assessing the influence of the testing procedures by replicating some of the tests in at least two laboratories. This section is organized in subsections corresponding to each type of test conducted. In each subsection are presented succinctly the equipment used and a summary of the test results.

#### Uniaxial tensile tests

**Fig. 2:**
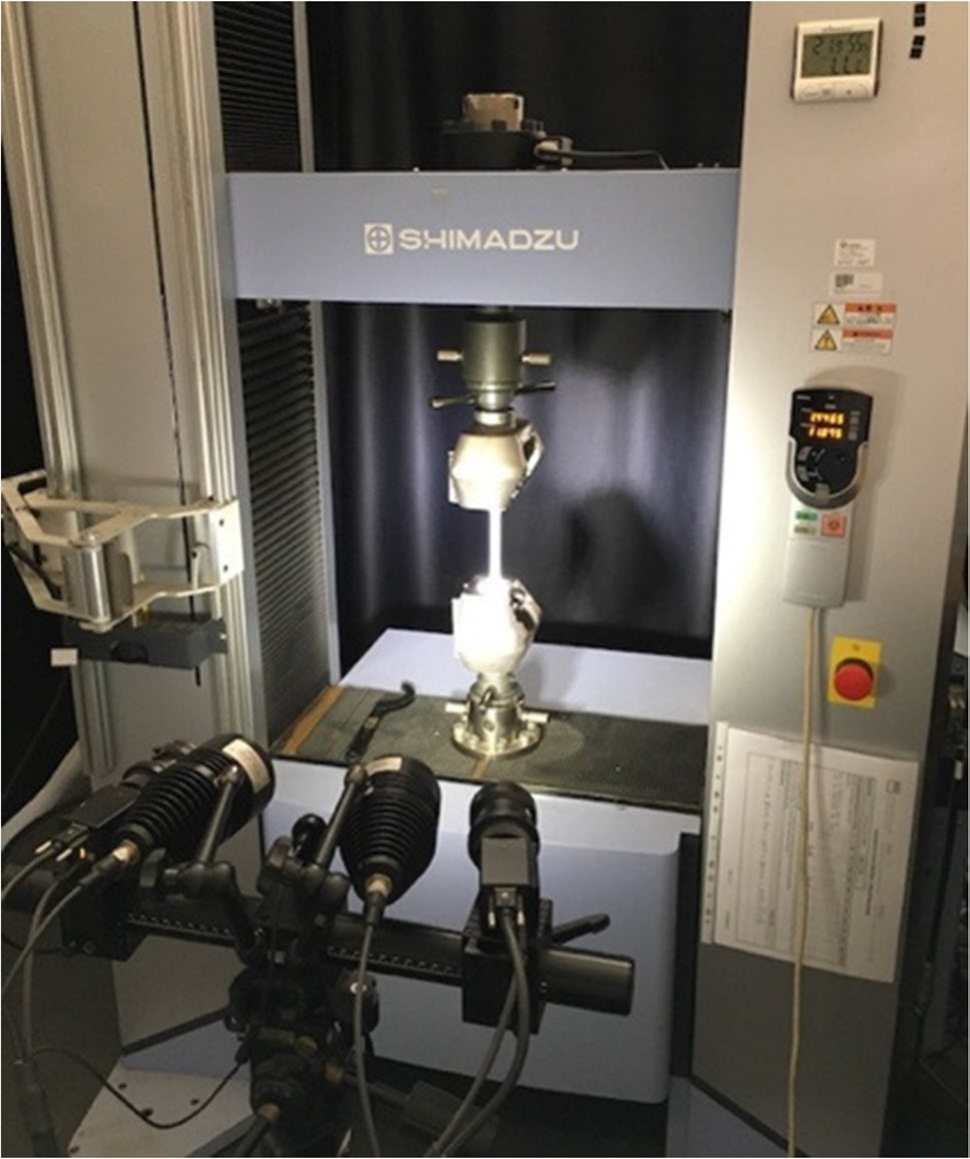
Shimadzu AG-X100kN and DIC (GOM)

Uniaxial tensile tests have been conducted at TUAT in 2018 and 2020. Standard specimens (JIS Z 2241) with 50 mm gauge length and 12.5 mm width were used. In 2018, samples were cut at each 15° from the rolling direction (RD) or 0° orientation, in the plane of the sheet. All the tests were conducted at a strain rate 10^−3^ s^−1^ using a Shimadzu tensile test machine AUTOGRAPH AG-250kNG (Shimazu Co., Japan). The strain up to fracture was measured with a mechanical extensometer SG50–100 (Shimazu Co.). To measure the Lankford coefficients, additional tests were conducted up to 10% nominal strain, and measurements were done with a high-resolution extensometer SG25–10 (Shimazu Co.). In 2020, additional tests were conducted at TUAT using the same equipment and testing procedure for samples cut at 0°, 45° and 90° orientations to RD, respectively. In 2020, at the University of Aveiro (UA), for the 0°, 45° and 90° orientations, uniaxial tensile tests were conducted at the same strain rate (10^−3^ s^−1^) on specimens of the same size (50 mm gauge length and 12.5 mm width) using a Shimadzu tensile test machine AUTOGRAPH AG-X100kN (Shimadzu Co., Japan). For the strain measurements, UA used a Digital Image Correlation (DIC) system and Aramis 5 M software of GOM (Germany) (see Fig. 2).

**Fig. 3:**
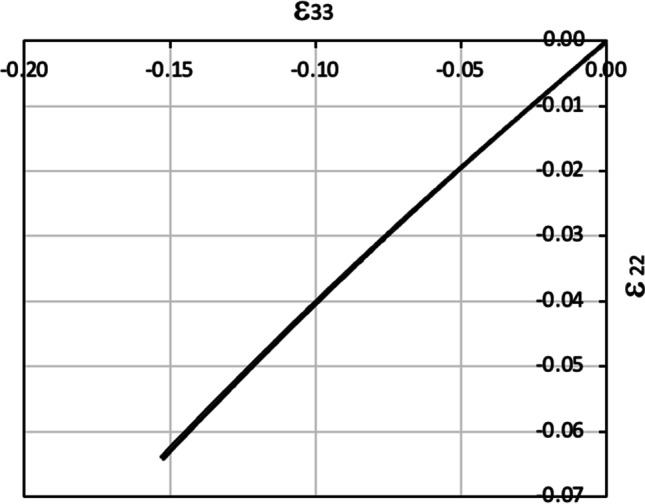
Width strain vs. thickness strain measured by DIC in a uniaxial tensile test along 90° direction

From the DIC data up to necking obtained at UA, the Lankford coefficient in any given orientation was estimated from the slope between the width strain (ε_22_), monitored during the whole test, and the thickness strain (ε_33_) based on volume conservation, as exemplified in Fig. 3 for a test at 90°. In addition, for the 0°, 45° and 90° orientations, the respective *r*-values were estimated from the slope of the width vs. thickness strains corresponding to the following plastic strain ranges: 5–10%, 10–15% and 15–20%. Note that r_0_ and r_90_ are practically constant while for r_45_ the variation with the axial strain is larger (see Fig. 5(c)).

**Fig. 4:**
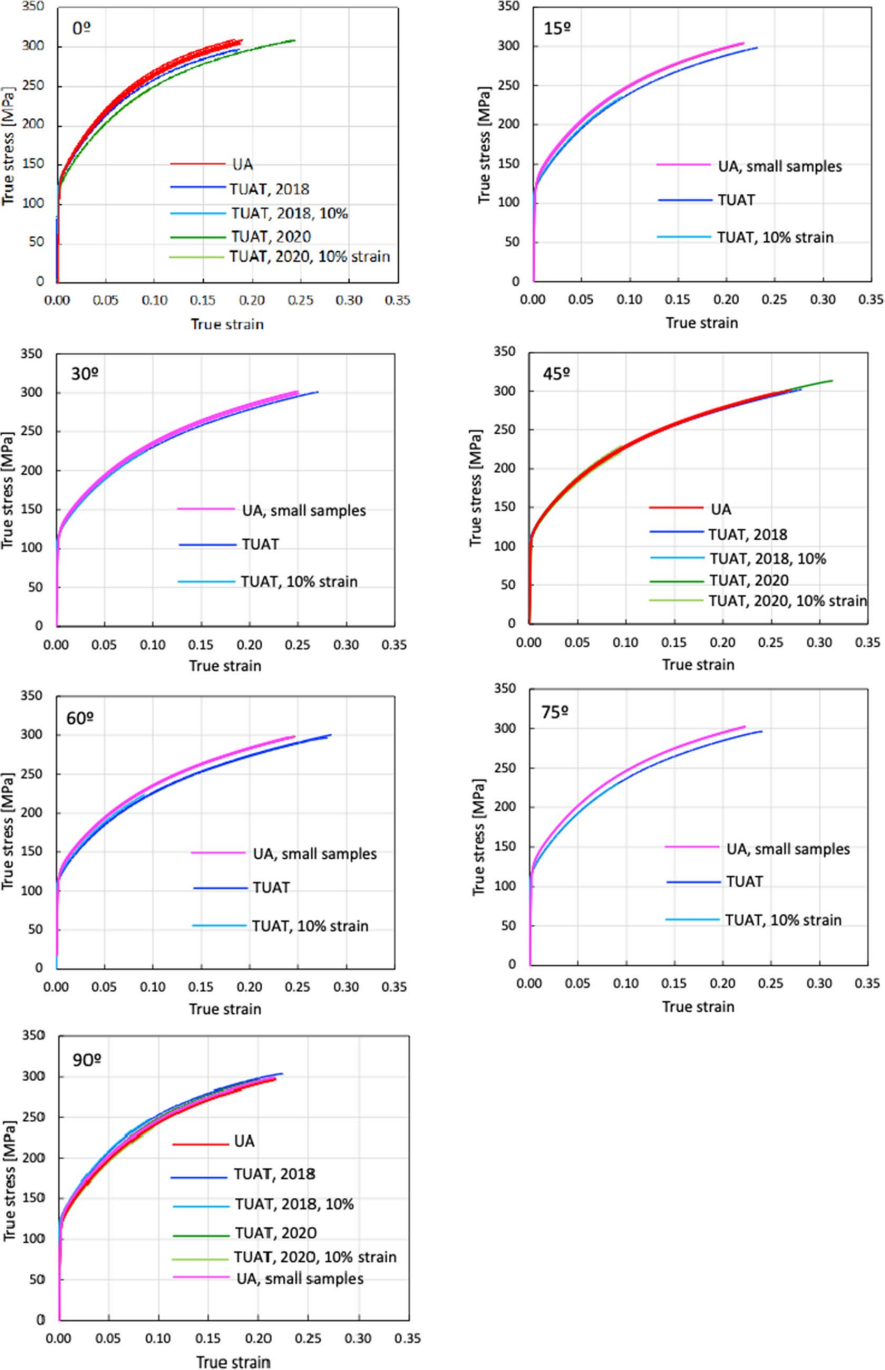
True stress - true strain curves of AA 6016-T4 in uniaxial tension. The curves in red and pink from UA correspond to standard and small specimens, respectively. The curves in blue and green from TUAT correspond to tests conducted in 2018 and 2020, respectively. The light blue and light green curves from TUAT were stopped at 10% nominal strain

To complement the characterization of the material anisotropy in the plane of the sheet, additional tests were performed at UA for the 15°, 30°, 60° and 75° orientations from the RD. Due to constraints related to material availability, these tests were conducted on smaller specimens with 25 mm gauge length and 9 mm width. It is worth mentioning that this geometry was verified in a previous work [101] and it was found that this specimen geometry does not affect the results. That conclusion can also be driven from the current results for the 6016-T4 material as checked for the 90° orientation (see Fig. 4).

To summarize, the uniaxial tensile tests conducted at TUAT and UA are listed in Table 3 while the stress-strain curves corresponding to each specimen orientation are given in Fig. 4. It is to be noted the very good reproducibility of the test results.

**Table 3:** Summary of the uniaxial tests and number of replicates for each orientation conducted in each laboratory

Angle from RD	TUAT (2018)	TUAT (2020)	UA (2020)	UA (2020) - small sample
0°	4	3	4	–
15°	3	–	–	3
30°	3	–	–	4
45°	3	3	4	–
60°	4	–	–	5
75°	3	–	–	3
90°	3	3	4	3

**Fig. 5:**
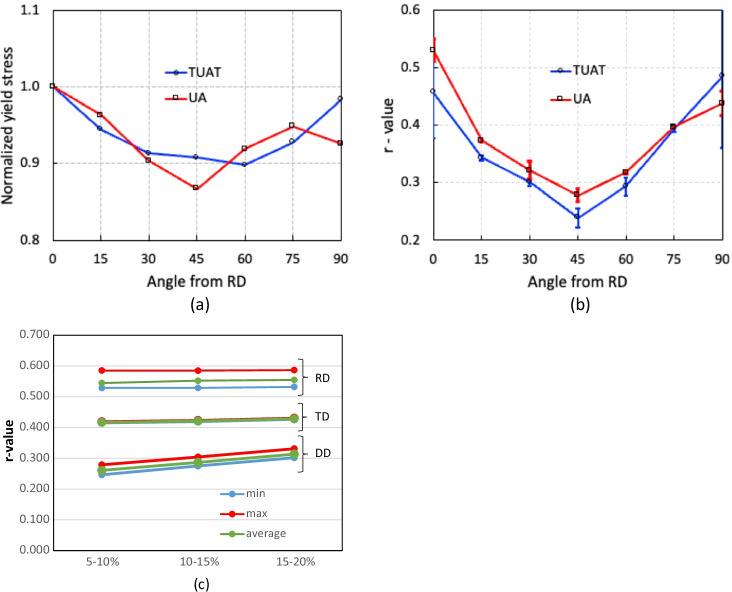
Anisotropy of the 6016-T4 sheet: **a** Experimental normalized yield stresses corresponding to $\varepsilon_0^{\mathrm{p}}$=0.08 at TUAT and $\varepsilon_0^{\mathrm{p}}$=0.002 at UA; **b** Experimental *r*-values obtained at UA from DIC data up to necking and at 10% of strain measured at TUAT in 2018 and 2020; **c** Experimental *r*-values based on UA tests for 3 plastic strain ranges

Figure 5 shows the experimental evolution of the *r*-values and yield stresses with the loading orientation. The corresponding numerical values and spread are given in Tables 4 and 5. The data indicate that the material displays a very little anisotropy in yield stresses and a pronounced anisotropy in *r*-values.

**Table 4:** Summary of the experimental *r*-values for AA 6016-T4 estimated from tests performed at UA and TUAT. For each orientation the average values from repeated tests (bold), the respective minimum and maximum values (in brackets) are given

Angle between RD and tensile direction	TUAT		UA
	2018	2020	2020
0°	**0.526** [0.499–0.553]	**0.384** [0.371–0.396]	**0.525** [0.505–0.554]
15°	**0.344** [0.339–0.348]		**0.359** [0.357–0.361]
30°	**0.301** [0.294–0.307]		**0.303** [0.277–0.320]
45°	**0.253** [0.253–0.254]	**0.229** [0.217–0.24]	**0.248** [0.236–0.267]
60°	**0.294** [0.278–0.309]		**0.297** [0.294–0.301]
75°	**0.393** [0.389–0.397]		**0.387** [0.384–0.390]
90°	**0.601** [0.580–0.621]	**0.368** [0.351–0.384]	**0.429** [0.404–0.454]

**Table 5:** Summary of the normalized experimental yield stresses for AA 6016-T4 obtained at UA and TUAT

Angle between RD and tensile test	0°	15°	30°	45°	60°	75°	90°
Normalized yield stress TUAT	1.000	0.944	0.913	0.908	0.898	0.928	0.983
Normalized yield stress UA	1.000	0.963	0.904	0.867	0.919	0.948	0.926

**Fig. 6:**
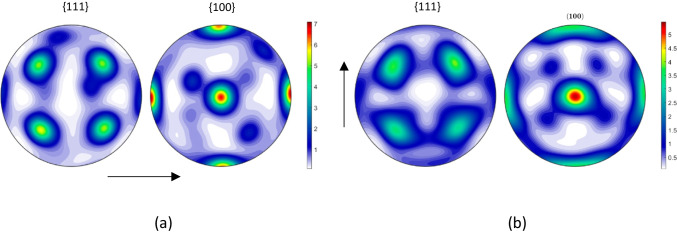
{111} and {100} pole figures of the material after uniaxial tensile tests conducted up to fracture: **a** along RD and **b** along TD. The arrows indicate the RD

To further examine whether there is evolution in the material anisotropy induced by uniaxial tension loading, EBSD measurements were performed on post-test samples from the RD and transversal direction (90° or TD) tests conducted at UA. The results are presented in Fig. 6. Note that no clear texture evolution occurred during uniaxial tension tests. There is only a small increase of the intensity value for the material stretched in the RD direction.

#### Biaxial tensile tests

**Fig. 7:**
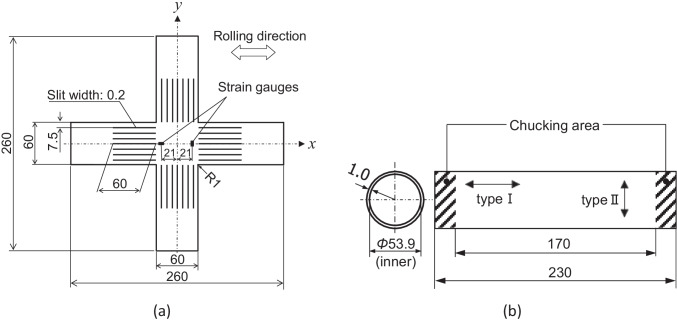
Geometry of cruciform specimen **a** and tubular specimen **b** used in biaxial tensile tests. Dimensions are in millimetres. The arrows indicate the RD

Figure 7(a) shows the geometry of the cruciform specimen used in the biaxial tensile tests. The specimen geometry and testing procedures have been established as an international standard: ISO 16842 [52]. A couple of strain gauges (YFLA-2, Tokyo Sokki Kenkyujo Co.) were mounted at ±21 mm from the centre along the maximum loading directions x and y (see Fig. 7) to measure the normal strain components *ε*_*x*_ and *ε*_*y*_. Using Finite Element analyses, Hanabusa et al. [43, 44] estimated that the stress measurement error is less than 2%, when the strain components are measured at the positions shown in Fig. 7(a). True stress increments were controlled and applied to the specimens so that the von Mises equivalent plastic strain rate became roughly constant at 5×10^−4^ s^−1^ for all stress paths.

Moreover, multiaxial tube expansion tests (MTETs) were performed to precisely measure the work hardening characteristics of the test samples for larger strain ranges than those obtainable from cruciform specimens. True stress increments were controlled and applied to the specimens so that the von Mises equivalent plastic strain rates became roughly constant at 5×10^−4^ s^−1^ for all stress paths. The details of the testing apparatus and procedures of the MTET are given in [58]. Figure 7(b) shows the geometry of the tubular specimens used in the MTETs. The as-received sheet samples were uniformly bent to form a cylinder and the sheet edges were welded using YAG laser. The inner diameter of the tubular specimen was 53.9 mm, and the gauge length (distance between the chucking areas at either end) was 170 mm. The maximum principal stress direction was always taken to be in the axial direction of the tubular specimens in the MTETs, as the strength of the heat-affected zone (HAZ) is lower than that of the base material. Therefore, two types of tubular specimens were made; the specimens of type I had the RD in the axial direction and were used for tests with *σ*_*x*_ > *σ*_*y*_, and the specimens of type II had the RD in the circumferential direction and were used for tests with *σ*_*x*_ < *σ*_*y*_.

Slight differences between the true stress vs. logarithmic plastic strain (${\sigma}-{\varepsilon}^{\mathrm{p}}$) curves obtained with the cruciform and tubular specimens were observed for all stress ratios, due to the influence of the prestrain applied to the sheet samples during tube fabrication. The prestrain, distributed linearly in the thickness direction, is equal to 0 at mid-thickness and takes the maximum and minimum values, ±0.018, at the outer and inner surfaces of the tube, respectively for the geometry shown in Fig. 7(b). The influence of the prestrain on the ${\sigma}-{\varepsilon}^{\mathrm{p}}$ curves measured using the MTETs was compensated using the procedures as described in [58].

Contours of plastic work in the stress space were measured to identify proper material models for the test samples subjected to biaxial tension. The true stress vs. logarithmic plastic strain curve (${\sigma}_0-{\varepsilon}_0^{\mathrm{p}}$) measurements for the RD were selected as reference data for work hardening; the plastic work per unit volume ${W}_0^{\mathrm{p}}$ associated with particular values of ${\varepsilon}_0^{\mathrm{p}}$ were determined. The stress point that gives the same plastic work as ${W}_0^{\mathrm{p}}$ on each linear stress path forms a contour of plastic work associated with ${\varepsilon}_0^{\mathrm{p}}$.

**Fig. 8:**
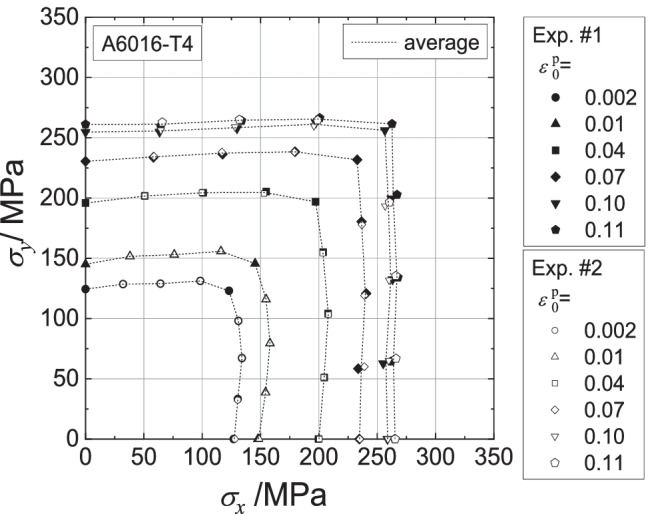
Stress points forming contours of plastic work at different levels of ${\boldsymbol{\varepsilon}}_{\mathbf{0}}^{\mathbf{p}}$

Figure 8 shows the stress points that form contours of plastic work. Two specimens were used per loading path. The maximum value of ${\varepsilon}_0^{\mathrm{p}}$ for which the work contour has a full set of stress points for nine linear stress paths was 0.11.

**Fig. 9:**
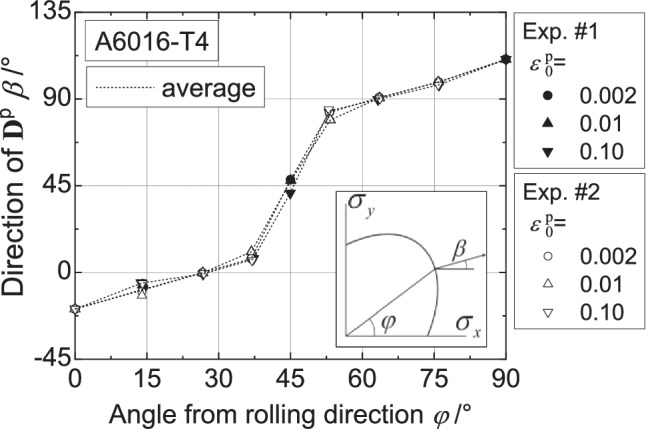
Direction of plastic strain rate at different levels of ${\boldsymbol{\varepsilon}}_{\mathbf{0}}^{\mathbf{p}}$

Figure 9 shows the directions of the plastic strain rates measured at different levels of ${\varepsilon}_0^{\mathrm{p}}$ and points that texture evolution during these biaxial tensile tests is either weak or does not affect the direction of the plastic strains. Let us remind that uniaxial tensile tests were characterized by a low texture evolution (see Fig. 6).

#### Monotonic and reverse simple shear tests

Shear test have been done in two laboratories, namely at the University of Liège (ULiege) and at the University of Aveiro (UA). Although the systems were very different, the common and favourable point is that the deformation volume is nearly identical, namely 30x3x1 mm^3^ in ULiege and 35x3x1 mm^3^ in UA.

**Fig. 10:**
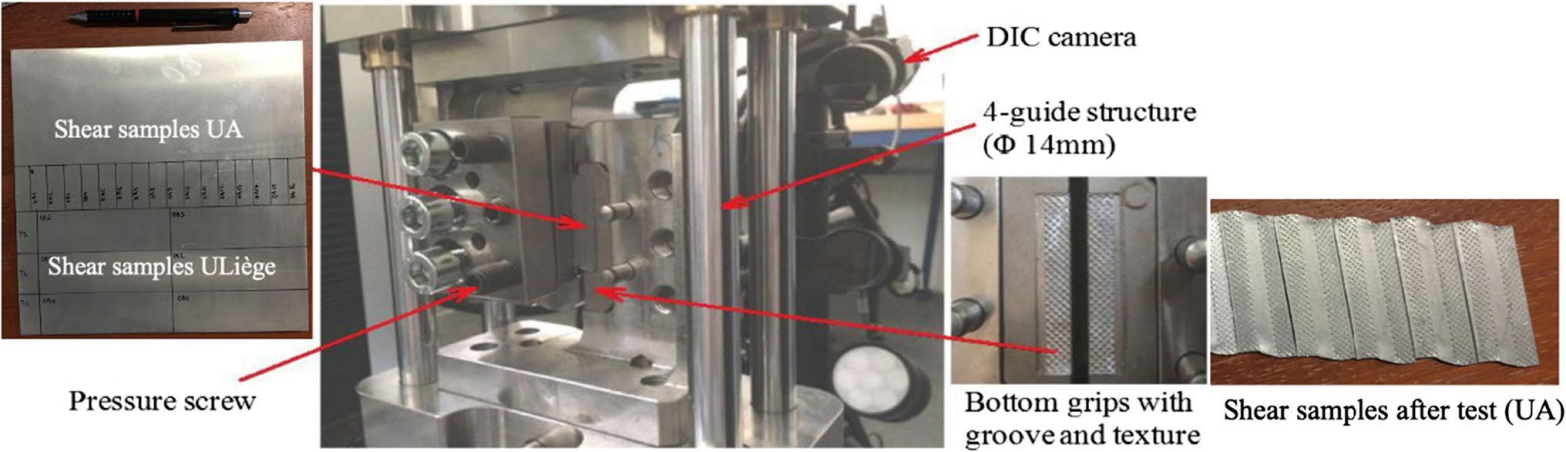
The dedicated shear device in UA as well as initial and deformed samples

To perform the simple shear tests in UA, the same equipment as in the case of the tensile test, namely AUTOGRAPH AG-X100kN (Shimazu Co., Japan) was used and a dedicated shear device was mounted. DIC coupled with Aramis 5 M software of GOM (Germany) was used to measure the strains. Details about the simple shear device can be found in [102] and in Fig. 10. The specimen size was 35x13x1 mm^3^ corresponding to length, width and thickness respectively.

**Fig. 11:**
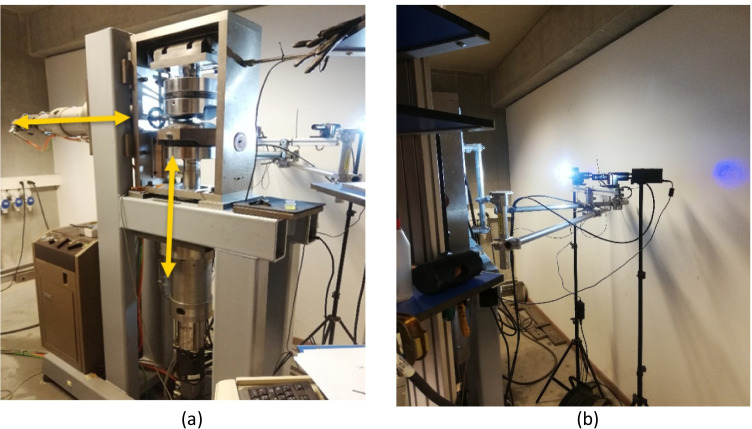
**a** Home-made biaxial machine and **b** optical system allowing to measure both the in-plane and out of plane displacement (ULiege)

The equipment used in ULiege is a biaxial in-house machine (Fig. 11). It has an optical system with 2 cameras to measure the strain, one computer to command the pistons connected to the software “Tema” and one computer for image acquisition from the optical system. The force and displacement acquisition from the biaxial machine are connected to the software VIC 3D. The size of the specimen used for the Benchmark was 100x30x1 mm^3^. The need for a large specimen is related to the hydraulic grips. The list of all the shear tests performed in both laboratories is given in Table 6.

**Table 6:** List of simple shear tests and number of replicates conducted in each laboratory

Angle from RD	Type of test	ULiege	UA
0°	Simple shear	3	5
0°	Reverse shear	2	3
45°	Simple shear	2	5
45°	Reverse shear	2	3

**Fig. 12:**
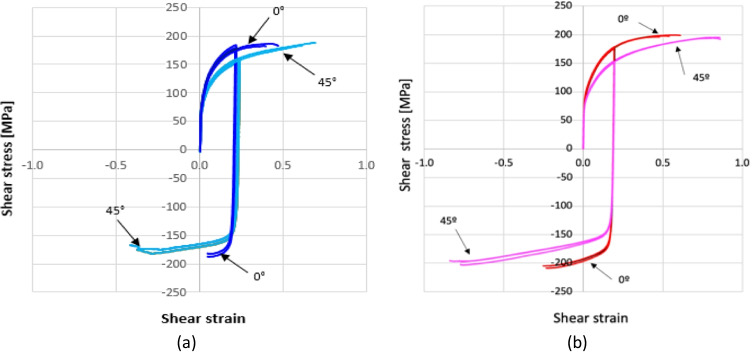
Shear stress - shear strain curves: **a** data from ULiege and **b** data from UA

**Fig. 13:**
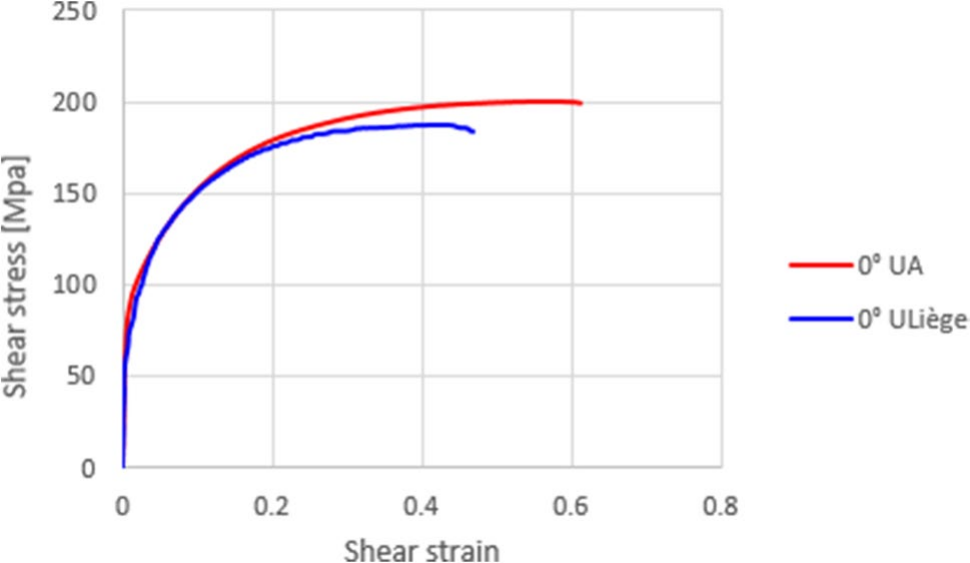
Comparison of shear test results from UA and ULiege

As shown in Fig. 12, the presence of a kinematic hardening is clear and the scattering of the shear tests in each laboratory is very low. The results of tests in 2 different directions enhance an anisotropic behaviour. Figure 13 compares the laboratory results, showing a reasonable agreement; the difference could be associated with the way each DIC system expresses the shear strain. This latter was calculated through the shear angle in Aramis software (used in UA), while VIC 3D software (used in ULiege) directly provided the Lagrange strain. Both measurements (shear angle and Lagrange strain) were calculated and an average was computed over the sheared area to generate the data shown in Fig. 12 (another source of potential differences).

#### EBSD measurements of the fully drawn cup of AA 6016-T4

**Fig. 14:**
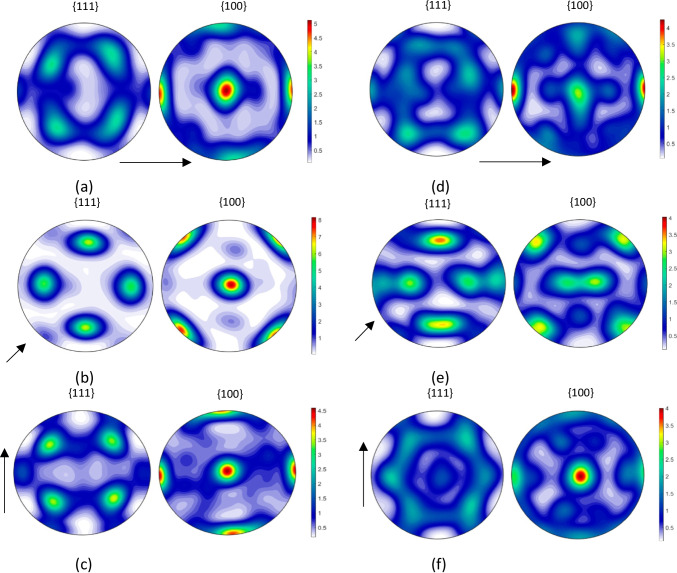
{111} and {100} pole figures obtained from the EBSD measurements performed on samples taken at the middle (left column) and at the top of fully draw cup (right column) respectively along (see **a** and **d**); 45º to RD (see **b** and **e**) and along TD (see **c** and **f**). The arrows indicate the RD direction

EBSD measurements were also performed after cup drawing. Samples were taken from two locations of the fully drawn cup, namely at the middle and the top of the cup along RD, 45° to RD and TD, respectively (see Fig. 14). As in the case of uniaxial tensile loading, no significant texture evolution is observed. Note that the maximum changes with respect to the initial texture were observed at the top of the cup.

## Cup forming and measurements

The proposed cylindrical cup drawing benchmark is used to investigate the anisotropic behaviour of an aluminium alloy AA 6016-T4 by measuring the earing profiles after cup forming. It will be the experimental reference result to compare with the numerical simulation predictions. Additional measurements are also considered, including punch force vs. punch stroke and the final thickness along the cylindrical perimeter from the sidewall of a drawn cylindrical cup for sections at different heights.

### Experimental forming tools and process conditions

**Fig. 15:**
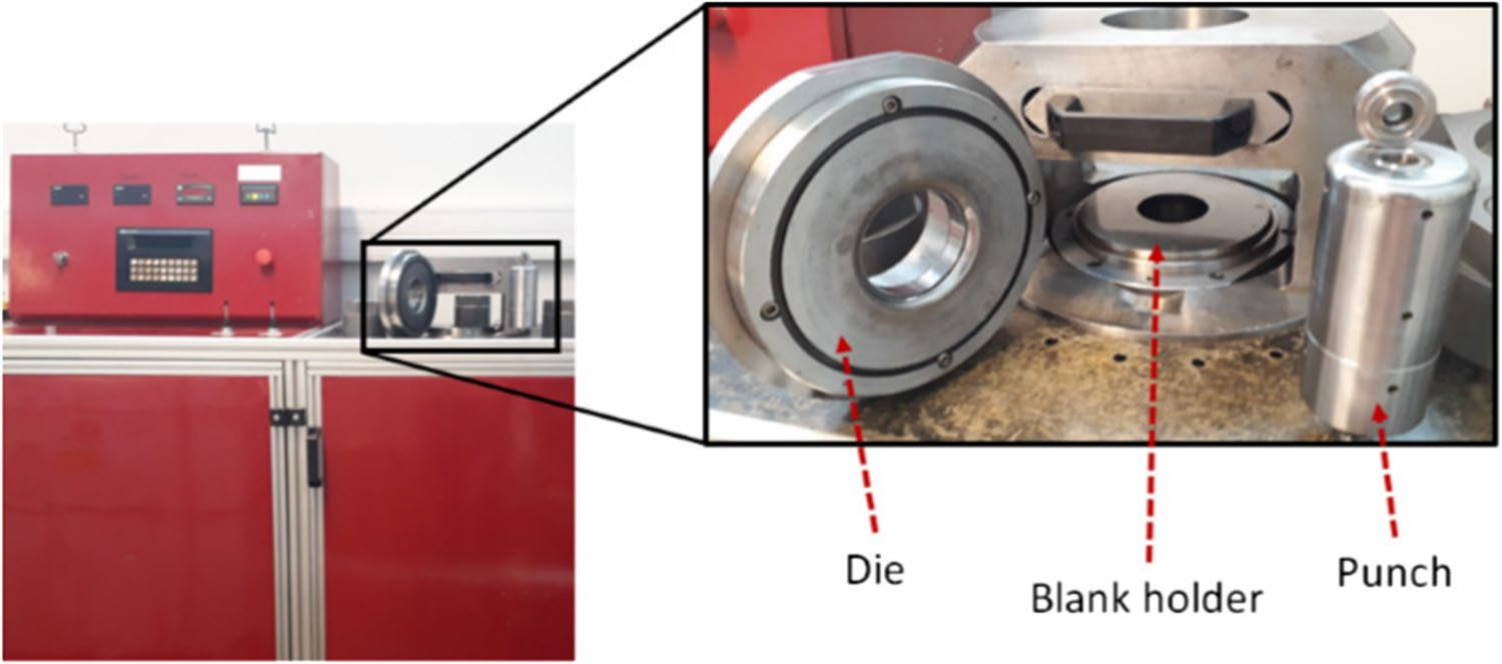
Universal testing machine and tools used for cylindrical cup benchmark

**Fig. 16:**
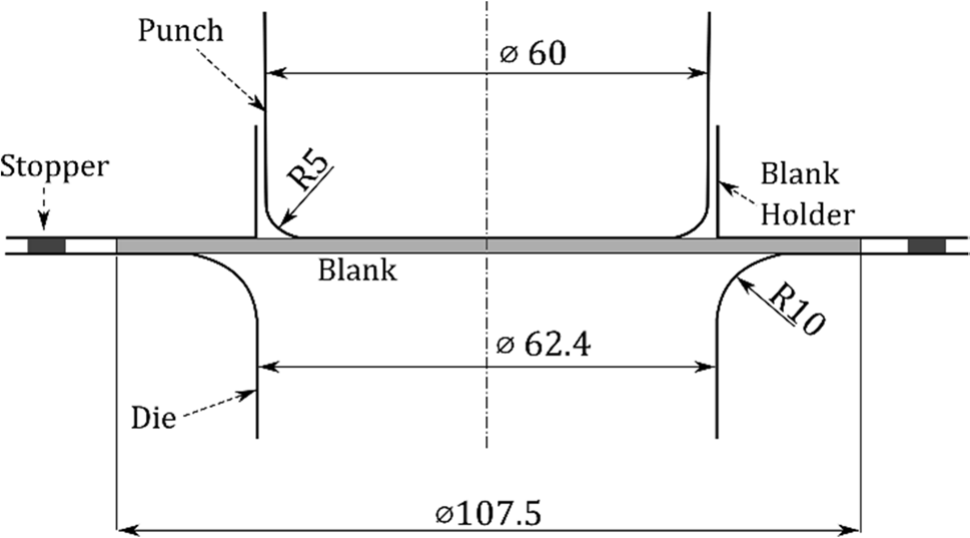
Cylindrical cup test schematic representation and tool dimensions

The cup test was performed in a hydraulic (250 bar) in house 300 kN Universal testing machine [88], as shown in Fig. 15. The tool configuration consisting of four parts: a die, a blank holder, a cylindrical punch and a stopper, which has the same thickness as the blank (see Fig. 16).

**Fig. 17:**
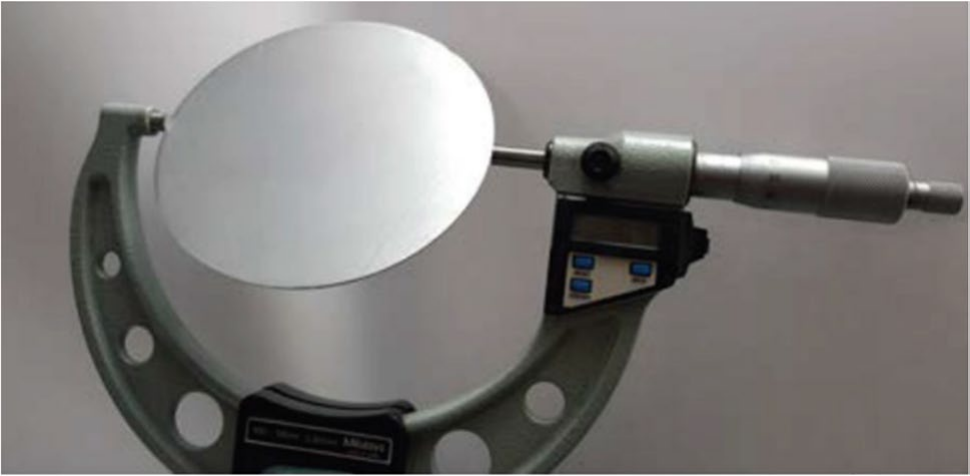
Methodology for measurement of blanks

The delivered material was a single sheet of AA 6016 with the size of 220x220x1 mm^3^, from which three circular blanks were extracted for testing, with a nominal diameter of 107.5 mm, thus giving a drawing ratio of 1.79 for cup drawing. The blank dimensions were measured (diameter and thickness) by using a micrometre, as shown in Fig. 17 and the corresponding results are presented in Table 7.

**Table 7:** Blank diameters and sheet thicknesses for AA 6016-T4 samples

Sample ID	Diameter [mm]	Thickness [mm]
1	107.54 ± 0.005	0.98 ± 0.004
2	107.54 ± 0.003	0.98 ± 0.004
3	107.53 ± 0.009	0.98 ± 0.006

Preliminary tests were performed also with AA 6016-T4 material of identical thickness but from a different supplier. Such trials were used to test and tune the experimental conditions, such as the drawing ratio to be used, blank holder conditions, output data and even the earing measurements and the lubrication conditions. Regarding the blank holder conditions, a stopper with the same thickness of the blank was used (see Fig. 16) and a constant force of 40 kN was selected in order to maintain the gap between the blank holder and the die.

### Punch force

**Fig. 18:**
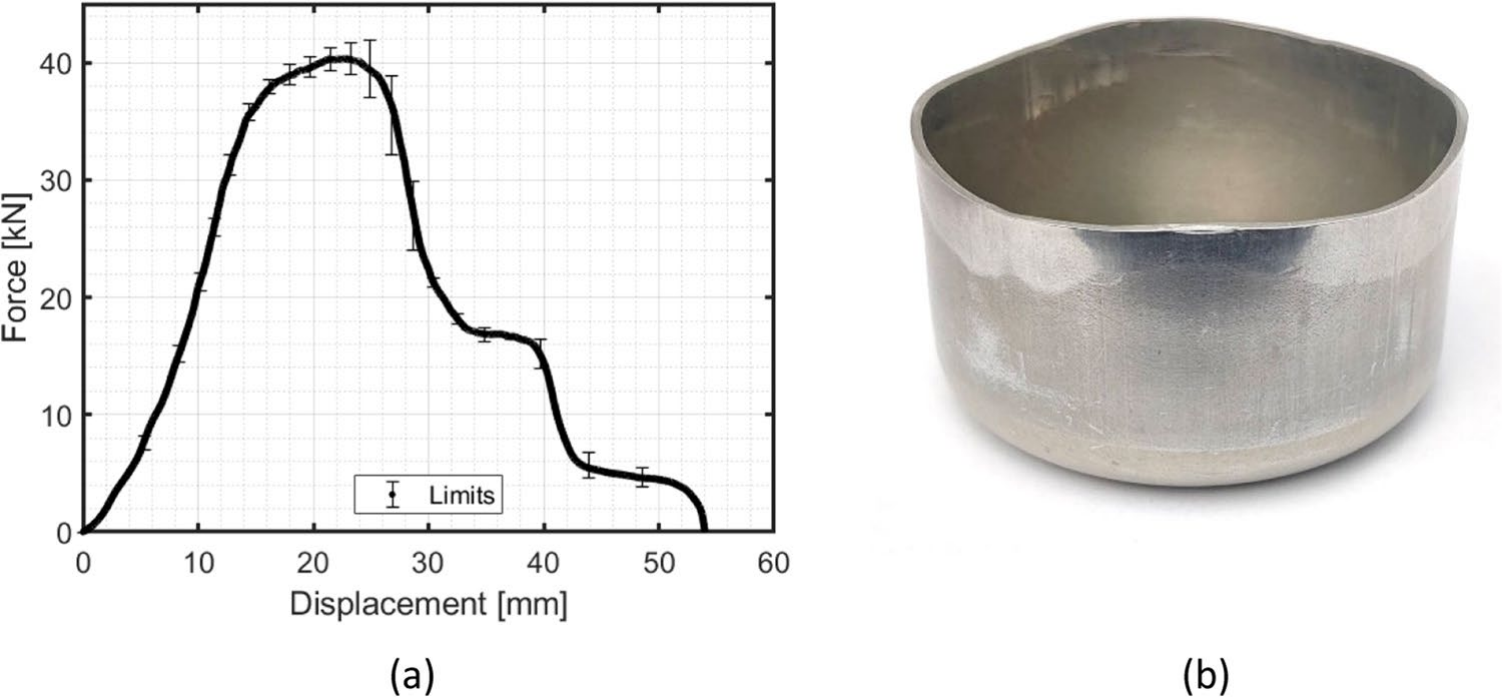
**a** Evolution of punch force vs. displacement and **b** final drawn cup

To fully draw the cup, a punch displacement of 54 mm is considered and a constant punch travel speed of 0.5 mm/s was defined. The punch force and the punch stroke are recorded during the test. The data acquisition was 50 Hz. Fig. 18(a) shows the resulting evolution of the punch force vs. displacement for the cup test. The final drawn cup is presented in Fig. 18(b). As observed in this figure, the upper part of the cylindrical cup shows some polished regions, thus denoting the occurrence of ironing, which is also related with the *plateau* observed in the punch force-displacement curve.

### Cup height and earing profile

**Fig. 19:**
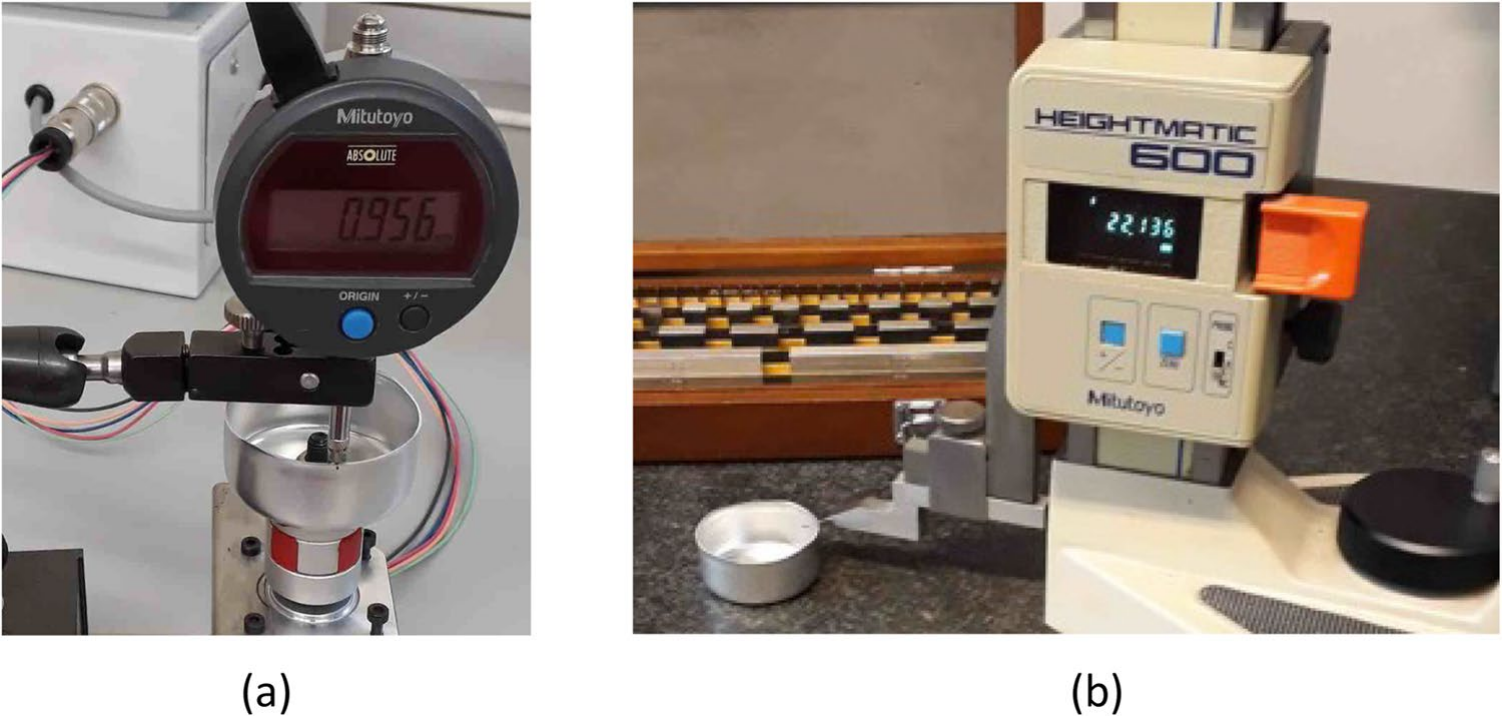
**a** Setup for measuring the earing height evolution **b** equipment to measure the total cup height

The samples of cylindrical cups were measured to obtain the earing profile along the perimeter of the top part of cup. Such measurement was performed using a Mitutoyo digital dial gauge micrometre with a resolution of 0.001 mm, as shown in Fig. 19(a). Rotation of cup was done by means of an electric motor. The measurements acquired with this digital dial gauge micrometre were relative measurements, with the zero-height defined for 0° to RD. The total cup height was performed with an additional setup using a high precision height gauge, Mitutoyo Heightmatic 600 mm, shown in Fig. 19(b).

**Fig. 20:**
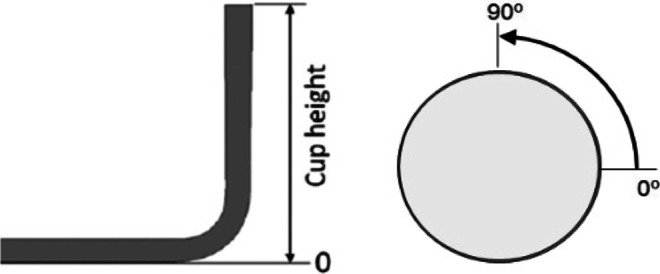
The earing profile evolution was measured around the cup circumference, starting from rolling direction (RD = 0°) and three anti-clockwise rotations (full 360°) were performed for each cylindrical cup

**Fig. 21:**
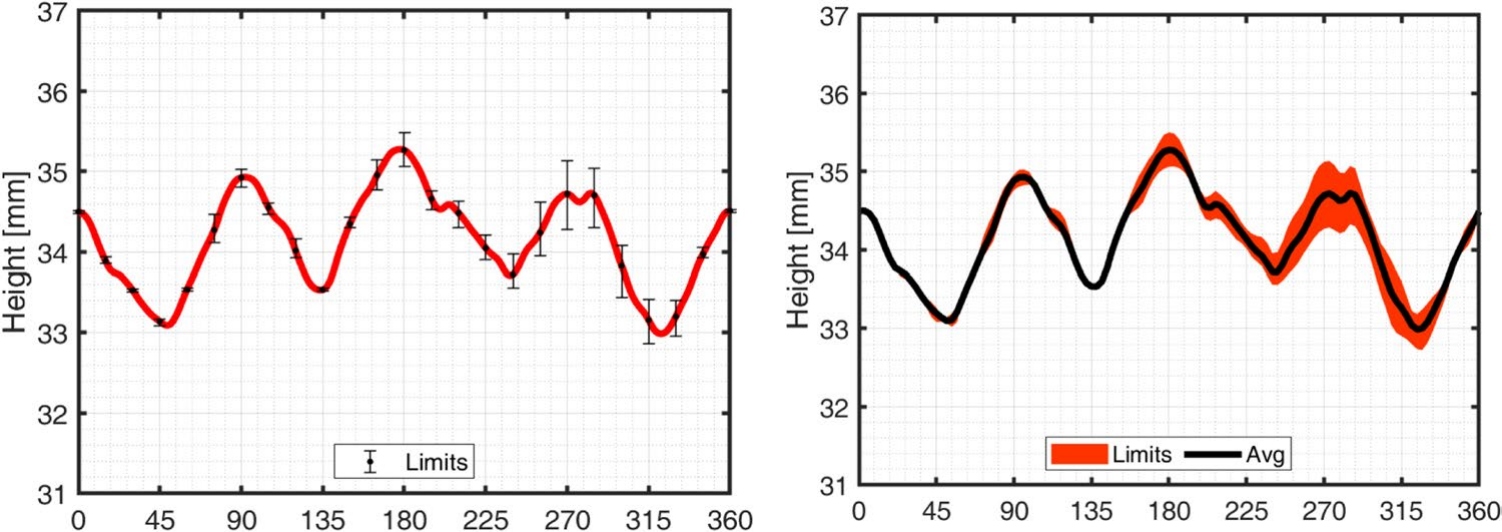
Evolution of average cup height for different angles

The direction of rotation for the motor and the sample was defined as clockwise rotation, as presented in Fig. 20. Therefore, the x-values on the results correspond to angle measurements, represented in Fig. 20, defined with anti-clockwise direction. Three complete rotations were considered for each sample, in order to assure repeatability. The average earing profile and the corresponding error limits are represented in Fig. 21.

### Cup thickness

**Fig. 22:**
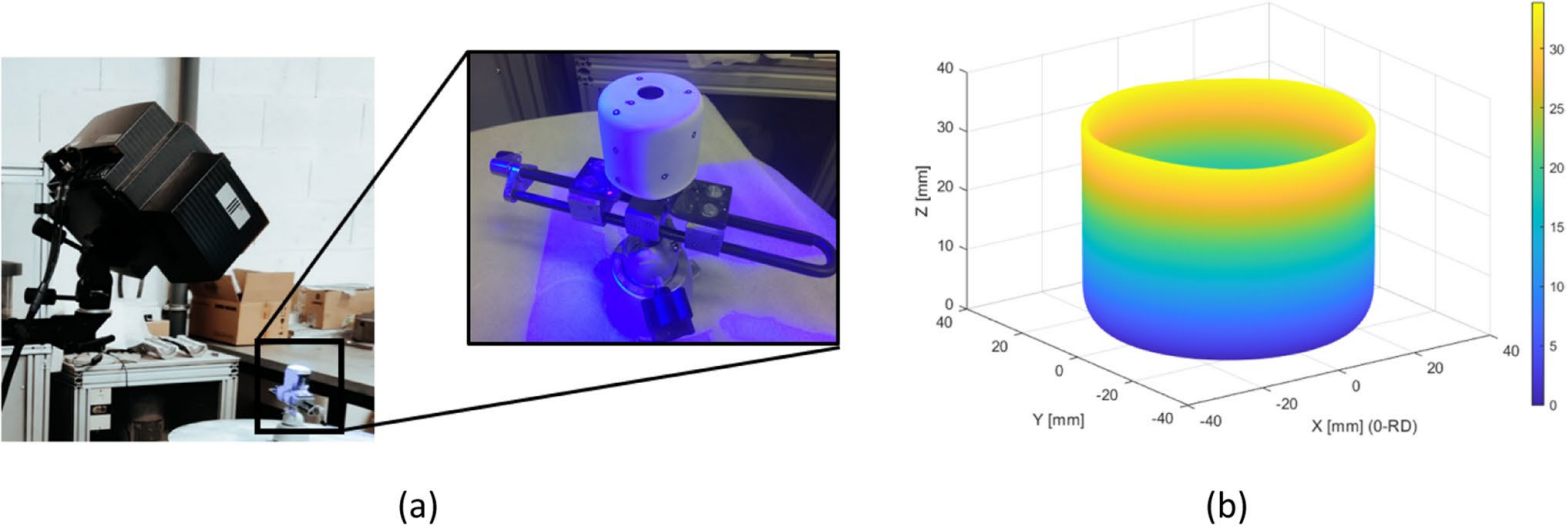
**a** Experimental **3**D measuring system **b** Cup geometry by point cloud

**Fig. 23:**
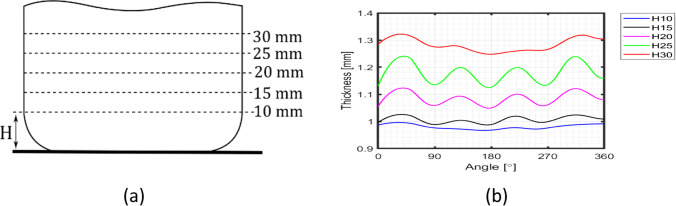
**a** Reference heights for the thickness measurements and **b** Evolution of thickness-angle for different cup heights

The thickness distribution was measured using an Industrial 3D Measuring System - ATOS Triple Scan as presented in Fig. 22. This equipment is a non-contact high-resolution optical digitizer, delivering three-dimensional data points. Figure 23 shows the evolution of thickness according to the cup perimeter considering different measurement heights.

It can be noticed that the measured thickness in the upper part of the cup is larger than the die-punch clearance of 1.2 mm (cf. Fig. 16). To confirm this finding, the tool dimensions have been re-measured with other techniques; all confirmed the dimensions in Fig. 16. Elastic deformations of the tools during the ironing stage could explain these observations.

### Measurement of tools and cup thickness by different methods

The results of thickness for the cup height of 30 mm, as seen in Fig. 23, gives rise to questions on tool dimensions (as defined in Fig. 16) and specially on the tool clearance between punch and die. As seen in Fig. 23, the average values of thickness for such cup height (30 mm) correspond to 1.3 mm, while the tool clearance is defined as 1.2 mm (Fig. 16).

It is clear that ironing occurs for this cup drawing, but the question follows: how can the sheet thickness be higher than the space between punch and die?

The first step to answer this question it is to verify and to confirm the measurements, for both tools and cup thickness. Accordingly, different strategies were used for new measurements:

- Tools were measured by two methods:
  - * Method T1* – measurement of Punch by using an outside micrometre (Mitutoyo Digimatic, 50–75 mm, 0.001 mm resolution); measurement of Die by using a digital 3-point internal micrometre (Mitutoyo, 0.001 mm resolution);
  - * Method T2* - 3D measuring system (ATOS Triple Scan e GOM Inspect) for both Punch and Die;
- Cup thickness was confirmed by three methods:
  - * Method C1* – ATOS Triple Scan, as already presented in “Cup thickness” Section;
  - * Method C2* – rotational system using an extensometer (Epsilon 3542), for data acquisition along the cup perimeter (height = 30 mm);
  - * Method C3* – discrete point measurements using an outside digital micrometre (Mitutoyo, 0.001 mm resolution) for points every 45°.

**Fig. 24:**
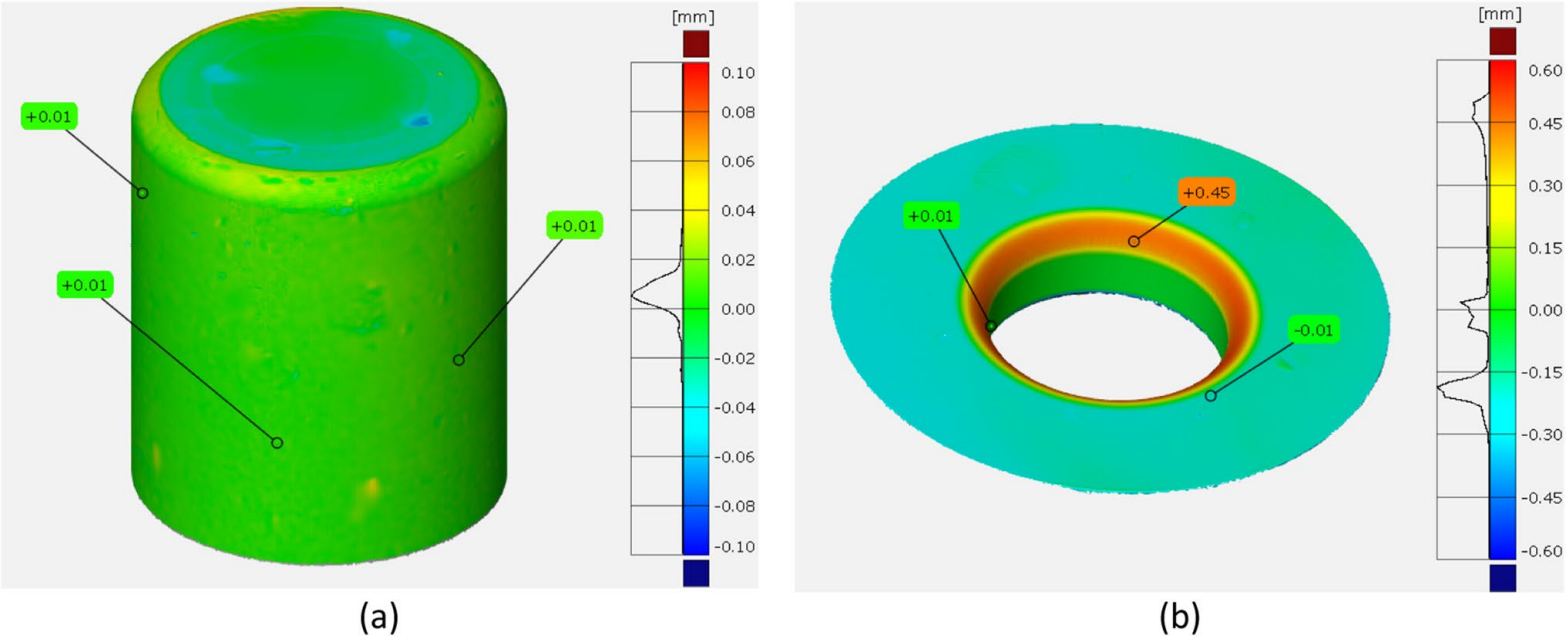
*Method T2* for tool measurements; results of differences to CAD data, using 3D measuring system (ATOS Triple Scan e GOM Inspect); **a** punch; **b** die

Results for tool measurements are presented in Table 8 and Fig. 24, respectively, for *Method T1* and *T2*. As seen, tool dimensions correspond to the defined dimensions, as presented in Fig. 16. Deviations on dimensions are in the order of ±0.001 mm, using *Method T1*, and ± 0.01 mm, using *Method T2*. There is an evident higher deviation only for die radius, which is detected when using *Method T2*, but this deviation is not affecting the current concern on tool clearance.

**Table 8:** Method T1 for tool measurements using a digital micrometre

	Calibration Reference [mm]	Measurement [mm]	Diameter [mm]
Punch	50.000	+ 10.002	60.00 ± 0.001
		+ 10.001	
		+ 10.000	
Die	59.998	+ 2.415	62.41 ± 0.01
		+ 2.415	
		+ 2.416

**Fig. 25:**
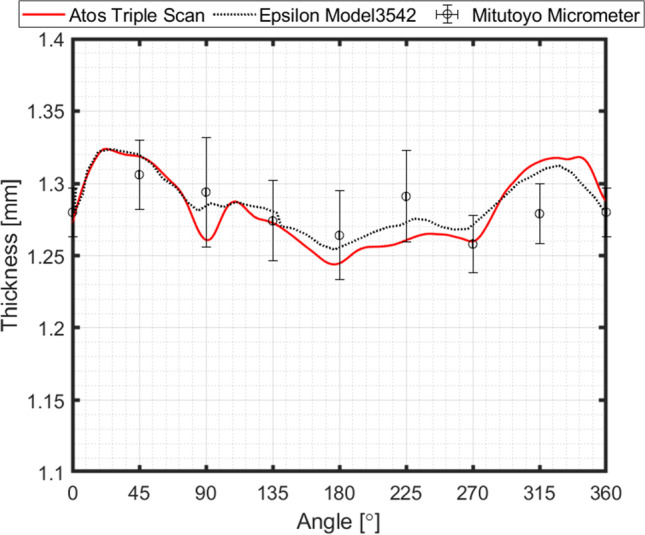
Thickness evolution for a cup height of 30 mm, using three methods

Results for cup thickness are presented in Fig. 25, were the previous measurements (Fig. 23, using ATOS Triple Scan) are compared with two additional methods (extensometer and micrometre). They are consistent among them, thus validating measurements by ATOS Triple Scan (Fig. 23).

These experimental results show that ironing occurs for cup drawing and it is indeed observed that the final sheet thickness is higher than the clearance between punch and die. These observations suggest that elastic deformation of the tools during the ironing stage can be a direction for an explanation of what our intuition would not tell us. Note that hereafter, the usual common approach to model tools as rigid bodies in FE drawing simulations is applied by all the Benchmark participants.

## Discussion about friction coefficient

In sheet metal forming processes, friction between the work piece and the tools is an important factor influencing the process behaviour such as material flow and forming forces, and further affecting the quality of formed products and tooling life [70]. In particular, for forming of aluminium alloys, the friction-induced galling phenomenon may commonly occur, leading to a significant impact on the quality of formed parts and the tool maintenance [38].

**Fig. 26:**
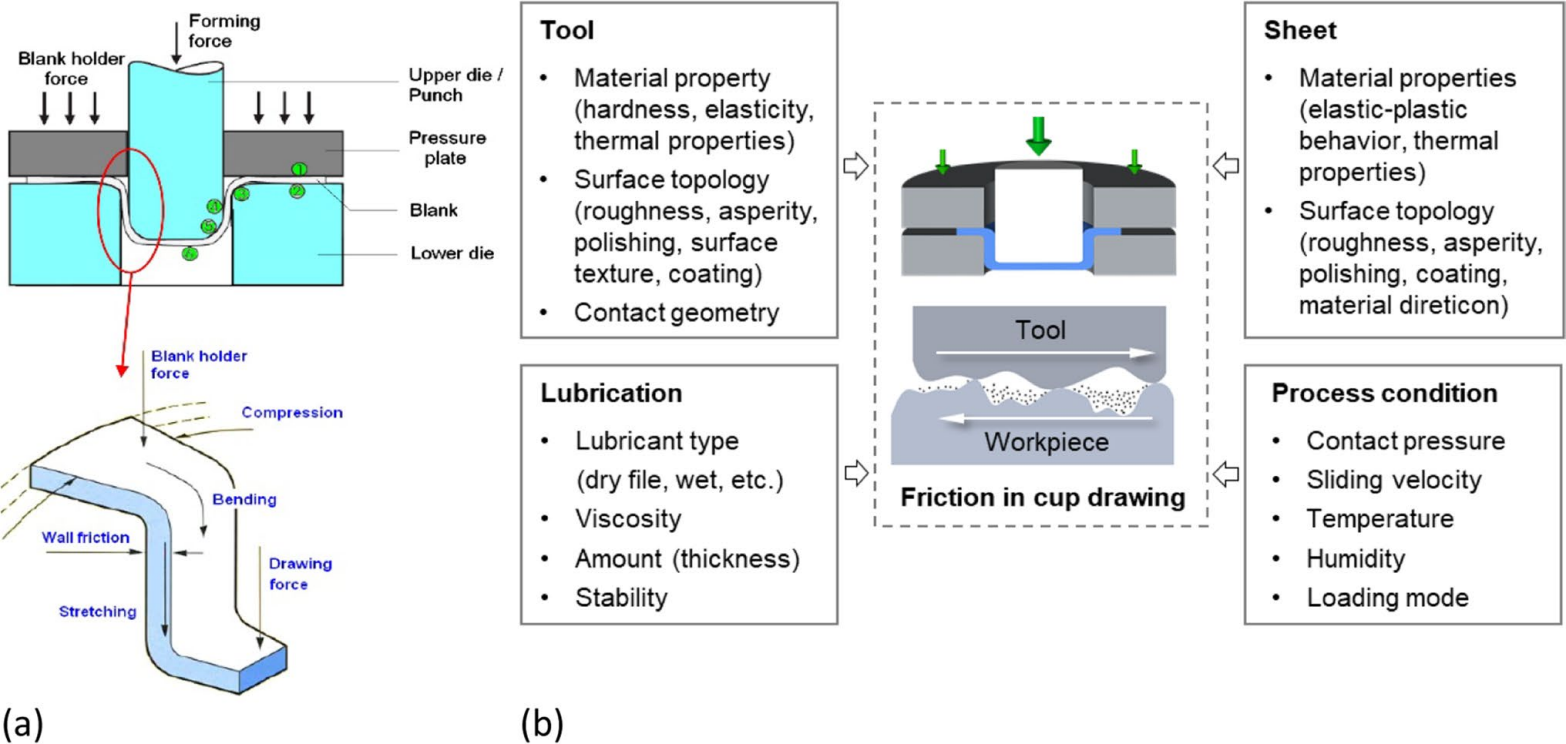
Friction in cup drawing process: **a** frictional characteristic [83]; **b** influential factors

The frictional behaviour varies with different metal forming operations due to various tooling/constraints, deformation characteristics, loading steps, etc. For cup drawing, as illustrated in Fig. 26(a), the friction occurring between the tools and the sheet has different characteristics depending on the respective zones, which can be summarized as follows:

- *Flange zone* (zone 1 and 2), in which a blank holder force is normally applied to prevent wrinkling. A relatively lower strain level is experienced. The severe friction in this zone is mainly characterized by abrasive and adhesive wear. The lubrication condition in the flange zone influences the thickness distribution in the sidewall of the formed parts as it affects the drawing force and stretching strain.
- *Corner zone of lower die* (zone 3), in which the sheet is forced to bend along the die radius and higher strain level is experienced. The contact stress in this zone is much higher and the wear pattern is similar to that of the flange zone; however, galling could possibly occur.
- *Cup wall zone* (zone 4), in which the sheet is stretched and punch-sheet contact is occurring. As the deformation progresses, the sheet further stretches and gradually sets apart from the interaction with the punch. However, if wrinkles form, a die-sheet contact and galling may occur in the wrinkling area.
- *Corner zone of punch* (zone 5), in which the interaction between sheet and punch corner exists, and wear characteristics are similar to those at the die corner zone.
- *Bottom zone* (zone 6), where the biaxial stress determines the stretch forming process. Under the action of the drawing force, the punch bottom contacts the sheet but with relatively small sliding.

Because of its complexity and the related difficulties associated with measurements of the friction coefficient, in FE simulation Coulomb’s law [24] with a constant coefficient is used [61]. However, different friction regimes (e.g. dry friction and mixed lubrication) may concurrently occur depending on the lubrication conditions and surface topography of the tools and sheet [47, 83]. Indeed, it has been reported that the lubrication affects the contact conditions at the sheet-tool interface [47, 61, 66, 83]. The different factors that affect the contact conditions and thus the frictional behaviour are depicted in Fig. 26(b) (see also [61]). Each contributing factor includes many variables that change during the forming process, so that the value of the friction coefficient may also evolve. For example in the case of lubricated deep drawing, it was reported in [74] that the friction coefficient is likely to vary in the range *μ* = 0.1 ~ 0.2. The material orthotropic behaviour also contributes to an uneven distribution of the contact pressure between the blank and the tools [8]. To account for this, some authors use a different value of the friction coefficient in RD and TD and in this way manage to improve the agreement between FE predictions and experimental earing profile [95]. Friction tests, with particular regard to influence of process parameters such as lubrication, e.g. [34, 85] or normal pressure, e.g. [31, 73] can provide a better compliance between experiments and simulations, however the influence of evolving friction coefficient was not studied in this benchmark.

**Fig. 27:**
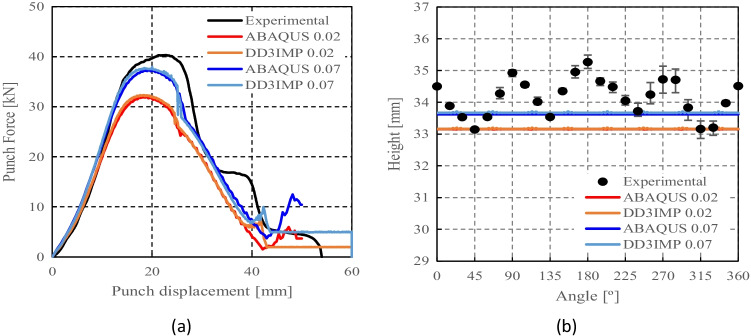
Effect of the value of the friction coefficient in cup drawing simulations obtained with FE codes DD3IMP and ABAQUS using von Mises yield criterion in comparison with experimental results for 6016-T4, **a **punch force evolution and **b** earing profile

Numerical simulations of the present benchmark were performed, using the von Mises yield criterion and two constant values for the friction coefficient: 0.02 and 0.07. As shown in Fig. 27(a), the value considered for the friction coefficient affects the predicted punch force throughout the cup drawing process. Setting a higher friction coefficient (from 0.02 to 0.07) leads to a predicted maximum punch force roughly 20% higher and a very slight increase of the corresponding punch displacement. As shown in Fig. 27(b), this is related with the increase in the predicted overall cup height of 0.5 mm, i.e. 1.5%. In fact, for the higher value of friction coefficient the predicted height is 33.8 mm against the experimental average of 34.1 mm (1.3% difference). These FE results show that for this material the von Mises yield criterion enables a good prediction of the average cup height, and that the prediction can be further “tuned” by changing the value of the friction coefficient. Nevertheless, with the von Mises criterion the thickening of the cup wall is underestimated. Indeed, the results in Fig. 27(a) indicate that irrespective of the value considered for the friction coefficient, a lower value is predicted for the ironing force as compared to the experimental data. Note that in Fig. 27 are presented results obtained with two FE codes: ABAQUS standard (conducted by REEF) and DD3IMP (performed by UCoimbra). Further details about the FE modelling (e.g. contact algorithm, type of elements) are presented in “Summary of features of FEM simulations” Section. Irrespective of the code adopted, the punch force and the cup height increase if a higher value of the friction coefficient is considered; the differences between the predictions obtained with the two codes being negligible. For more examples and further discussion on the influence of the modelling strategies, including the algorithms used for the treatment of the contact conditions in different FE codes, the reader is referred to “Cup drawing simulations, analysis and discussion” Section.

## Summary of features of FEM simulations

A total of 11 teams contributed with results to the benchmark, as summarized in Table 9. Some teams provided more than one result, to enrich the discussion. Table 9 presents the codes adopted, highlighting that the majority adopted ABAQUS code, which is connected with the possibility to integrate user-defined material subroutines. In this context, two teams used their own in-house codes (ULiege with Lagamine and UCoimbra with DD3IMP). Regarding the time integration scheme, ABAQUS, and MSC.MARC allow the selection of either the implicit or the explicit strategy. As shown in Table 9, 5 teams adopted the implicit scheme, while 5 used the explicit one. One team selected the approach in function of the chosen code. This table also presents the selected element type, often solid. Only one team opted for continuum-shell elements and another for shell. USakarya and USiegen contributed with results using shell and solid elements. Their choice reflects the difficulties in predicting the ironing of the cup wall (see “Hill48 with associated flow rule” Section) with shell elements.

**Table 9:** Choice(s) of FE code(s) and element type(s) by each team. Total number of finite elements used to discretise the blank (for solid elements, the number of through-thickness layers is shown in brackets, while for shell or continuum-shell elements the number of integration points through the thickness is between brackets)

Acronym	Code	Element type	Total FE
KUL	ABAQUS (explicit) 2019	Continuum Shell (SC8R)	9743 (5)
NTNU	ABAQUS (implicit) 6.14	Solid (C3D8R)	7401 (3)
POSTECH	ABAQUS (explicit) 6.13	Solid (C3D8R)	1344 (2)
REEF	ABAQUS (implicit) 6.14	Solid (C3D8R)	10,900 (3)
			15,660 (3)
UAalto	ABAQUS (explicit) 2020	Solid (C3D8R)	40,143 (2)
UCoimbra	DD3IMP (implicit)	Solid (Selective Reduced Integration)	15,982 (2)
UGent	ABAQUS (implicit) 6.13	Shell (S4R)	11,027 (5)
ULiege	Lagamine (implicit)	Solid (BWD3D one integration point)	2904
UPorto	ABAQUS (explicit)	Solid (C3D8R)	2128 (2)
USakarya	LS-DYNA R5.1 (explicit)	Quadrilateral shell (elform = 16), Fully integrated	3072 (7)
	MSC.MARC (implicit)	Solid Hexahedral, Fully integrated	3072 (1)
USiegen	LS-DYNA R10/R12 (explicit)	Quadrilateral shell based on Hu-Washizu Three Field Principle, Fully integrated	11,112
		Hexahedral solid (elform = −2), Fully integrated	55,560 (5)
	PAMSTAMP 2020 (explicit)	Belytschko-Tsai quadrilateral shell, Fully integrated	9558
		Flanagan-Belytschko hexahedral solid, Fully integrated	55,560 (5)

**Fig. 28:**
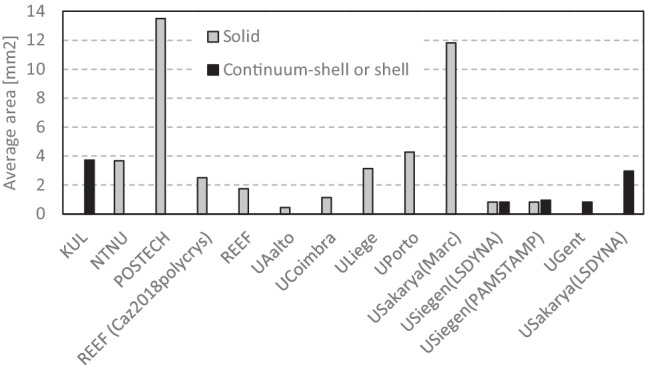
FE average in-plane size for the models adopted by each team

Only two teams adopted a full mesh to model the 360° geometry: KUL and USakarya, but the latter used this approach only with MSC.MARC code. KUL used the full model to impose some deviation to the initial blank positioning of the blank on the tools, in order to improve the correlation with the experimental results. In Table 9, the mesh refinement is described: the total number of elements used to discretise the blank and the number of layers (number in brackets) through the thickness, in case of solid elements. In case of shell or continuum-shell elements, it is presented the number of integration points through the thickness (in brackets). The dispersion in the total number of elements is very high, reflecting different options concerning the average in-plane finite element size. In fact, the full models are not the ones using the highest number of elements in the sheet plane. The average in-plane element area was determined based on the initial area of the blank and the total number of elements used in the sheet plane. The results shown in Fig. 28 highlight that the average value is typically lower (~1.9 mm^2^) for shell elements than for solid element (~4.0 mm^2^).

All teams considered the tools as rigid. Since their geometry is quite simple, the analytical description of the tools was adopted by UGent, UPorto and USakarya (when using LS-DYNA). Remaining teams adopted a description with rigid elements, except UCoimbra that uses Nagata surfaces. In terms of contact search algorithm, most teams resort to the surface-to-surface approach. In terms of method to enforce the contact constraints, most of the teams used the penalty method. All teams adopted the Coulomb friction model, with a constant value for the friction coefficient, indicated in Table 10. USakarya also considered a shear stress limit of 70 MPa (when using MSC.MARC). In general, the teams selected the constant friction coefficient based on the prediction of the punch force, during the drawing stage. This resulted in a value in the range of 0.075–0.1. Nevertheless, as previously mentioned in “Discussion about friction coefficient” Section, this leads to an overestimation of the force in the ironing stage, which explains the use of a lower value by other teams. Moreover, in order to avoid the ironing effect, USakarya altered the clearance between the punch and the die to 1.4 mm.

**Table 10:** FE code, contact algorithm and friction coefficient adopted in the Coulomb law

Acronym	Code	Contact algorithm	Friction coefficient
KUL	ABAQUS	Surface-to-surface, Penalty contact method, Tangential (friction: penalty), Normal (hard contact)	0.09
NTNU	ABAQUS	Finite sliding	0.09
POSTECH	ABAQUS	Penalty contact	0.1/0.07
REEF	ABAQUS	ABAQUS built-in contact pressure-overclosure (linear)	0.02
UAalto	ABAQUS	Penalty contact	0.01
UCoimbra	DD3IMP	Node-to-surface; Augmented Lagragian	0.02/0.07/0.10
UGent	ABAQUS	Surface-to-surface, Tangential (penalty) and hard contact	0.07
ULiege	Lagamine	Penalty contact method at integration points with a surface layer of elements (CFI3D)	0.082
UPorto	ABAQUS	Penalty contact	0.05
USakarya	LS-DYNA	Forming one way surface-to-surface	0.10
	MSC.MARC	Segment-to-segment	
USiegen	LS-DYNA	Forming one way surface-to-surface	0.075
	PAMSTAMP	Surface-to-surface	

The teams reported different strategies to control the blank holder, in order to mimic the presence of the stopper used in the experimental setup (see Fig. 16). In some cases, this involved the modelling of a rigid body stopper. UPorto considered a constant distance of 1.2 mm between the die and the blank holder.

The list of the yield criteria selected by the teams is presented in Table 11, confirming the enormous variety of options. Some yield criteria appear more frequently, because their anisotropy parameters were supplied by the benchmark committee. This is not the case of the Yld2000-2D and the Yld2004–18p, which were also selected by two of the teams using crystal plasticity models: UGent and NTNU, respectively. Regarding the hardening law, the summary is presented in Table 12. Only one team modelled the kinematic component of the hardening behaviour, USakarya (when using the MSC.MARC). Many teams adopted the Swift isotropic hardening law since its parameters were supplied by the benchmark committee. Note that ULiege team chose Voce isotropic hardening law coupled with Hill48 criterion and Swift hardening law coupled with their Crystal plasticity law Minty.

**Table 11:** FE code and yield criterion used by each team, with (A) for an associated law, (NA) for a non-associated choice. For polycrystalline model, their use for identification (I) or forming simulations (F) is noted in last column. The source of the set of anisotropy parameters used (benchmark committee, own identification) is also indicated

Acronym	Code	Yield criterion	Benchmark set	Own set	CP model
KUL	ABAQUS	Facet-3D		X	ALAMEL (I)
NTNU	ABAQUS	Yld2004–18p (A)		X	DAMASK (I)
		Yld2004–18p (A)		X	
POSTECH	ABAQUS	Yld2004–18p (A)	X		
		Caz2018singlecrys		X	
REEF	ABAQUS	Hill48 (A)	X		
		Caz2018-Orth (A)	X		
		Caz2018singlecrys		X	
		Caz2018polycrys		X	Taylor (F)
UAalto	ABAQUS	Hill48 (NA)		X	
UCoimbra	DD3IMP	Hill48 (A)	X		
		CB2001 (A)		X	
		CPB06ex2 (A)		X	
		Caz2018-Orth (A)	X		
UGent	ABAQUS	Yld2000-2D (A)		X	VPSC (I)
ULiege	Lagamine	Hill48 (A)		X	
		Minty		X	Taylor (F)
UPorto	ABAQUS	Hill48 (A)	X		
		Hill48 (NA)		X	
USakarya	LS-DYNA	Yld2000-2D (A)	X		
		Yld89 (A)		X	
		HomPol4 (A)		X	
		HomPol6 (A)		X	
	MSC.MARC	HomPol4 (A)		X	
		HomPol6 (A)		X	
USiegen	LS-DYNA	Hill48 (A)	X		
		Yld2000-2D (A)	X		
		Yld2004–18p (A)	X		
	PAMSTAMP	Hill48 (A)	X		
		Hill48 (NA)	X		
		Yld2000-2D (A)	X

**Table 12:** For each team, choice of FE code and hardening law used; the source of the set of parameters (benchmark committee, own identification) is also indicated

Acronym	Code	Hardening law	Benchmark set	Own set
KUL	ABAQUS	Swift, isotropic		X
NTNU	ABAQUS	Voce, isotropic		X
POSTECH	ABAQUS	Voce, isotropic		X
REEF	ABAQUS	Swift, isotropic	X	
UAalto	ABAQUS	Voce, isotropic		X
UCoimbra	DD3IMP	Swift, isotropic	X	
		Voce, isotropic		X
UGent	ABAQUS	Voce, isotropic		X
ULiege	Lagamine	Voce, for Hill48 law		X
		Swift, for Minty law		X
UPorto	ABAQUS	Swift, isotropic	X	
USakarya	LS-DYNA	Swift, isotropic	X	
	MSC.MARC	Swift, isotropic		X
		Swift, isotropic + Chaboche, kinematic		X
USiegen	LS-DYNA	Swift, isotropic	X	
	PAMSTAMP	Swift, isotropic	X	

## Main features of constitutive laws

Hereafter all the constitutive laws used by the Benchmark participants are presented.

To account for the observed anisotropy in the plastic deformation of the AA 6016-T4 polycrystalline sheet alloy, both polycrystalline models and macroscopic phenomenological elastoplastic models were adopted. Crystal plasticity models and Fast Fourier Transformation approach (FFT) were for instance used to generate virtual tests or material features. The anisotropic yield functions and plastic potentials involved in the elastoplastic models were identified either using only mechanical data (POSTECH, UCoimbra, USiegen, USakarya) or using a combination of mechanical data and results of polycrystalline simulations (KUL, UGent, NTNU, ULiege, REEF).

For simulation of the cup forming process, most of the participating teams have used phenomenological elastoplastic models. However, some teams also directly exploited crystal plasticity simulations. UGent applied a Visco-Plastic-Self-Consistent (VPSC) model, ULiege an interpolation model based on the yield locus concept, where points of this surface are computed by a crystal plasticity approach. In addition, direct FE simulations of cup drawing using a single crystal plasticity law were performed by POSTECH, while a polycrystalline model based on the same single crystal plasticity law was reported by REEF.

We begin with the presentation of the general form of the governing equations for modelling rate-independent elastoplastic deformation. Next, in “Orthotropic yield functions” Section, are presented the 2-D and 3-D macroscopic orthotropic yield functions (phenomenological laws identified by classical mechanical tests), while “Crystal plasticity based constitutive models”  Section describes the polycrystalline models. They rely for their identification either on crystal plasticity models, like FACET-3D yield surface, associated with ALAMEL crystal plasticity model (see “Facet-3D model linked to ALAMEL crystal plasticity model” Section) or directly use crystal plasticity models within the constitutive law as the elasto visco-plastic-self-consistent approach implemented by UGent (see “Viscoplastic self consistent model (VPSC)” Section).

The set of equations governing elastoplastic behaviour are:

1 $$\left\{\begin{array}{l}\mathbf{D}={\mathbf{D}}^\mathrm{e}+{\mathbf{D}}^\mathrm{p}\\ {}\dot{\boldsymbol{\sigma}}={\mathbf{C}}^\mathrm{e}:{\mathbf{D}}^\mathrm{e}\\ {}f\left(\boldsymbol{\upsigma}, \mathbf{X},\xi \right)=\overline{\sigma}\left(\mathbf{s}-\mathbf{X}\right)-Y\left(\xi \right)\le 0\\ {}{\mathbf{D}}^\mathrm{p}=\dot{\lambda}\frac{\partial g}{\partial \boldsymbol{\upsigma}}\\ {}\dot{\lambda}\ge 0\;\mathrm{and}\;f\dot{\lambda}=0\end{array}\right.$$

where **s** is the Cauchy stress deviator ***s*** = ***σ*** − *σ*_*m*_**I** with ${\sigma}_m=\frac{1}{3}\mathrm{tr}\left(\boldsymbol{\sigma} \right)$, where **I** is the second-order identity tensor while “tr” denotes the trace operator, **D** is the strain-rate tensor with **D**^e^ and **D**^p^ being its elastic and plastic part, respectively.

Eq. (1)_2_ is the hypo-elastic law defining the stress rate with respect to the elastic strain rate, **C**^e^ is the elastic fourth-order stiffness tensor while “:” denotes the double contracted product between the two tensors. Assuming linear isotropic elastic response, with respect to any coordinate system, **C**^e^ is given as:

2 $${C}_{\boldsymbol{ijkl}}^e=G\left({\delta}_{ik}{\delta}_{jl}+{\delta}_{il}{\delta}_{jk}\right)+\left(K-\frac{2}{3}G\right){\delta}_{ij}{\delta}_{kl}$$

with *i*, *j*, *k*, *l* = 1...3, *δ*_*ij*_ is the Kronecker delta, while *G* and *K* are the shear and bulk modulus, respectively. Eq. (1)_3_ defines the elastic domain with *f* denoting the yield function, $\overline{\sigma}$ the effective stress which depends on the Cauchy stress deviator ***s*** and hardening variables which can be scalar or tensorial (e.g. the back-stress **X**). Eq. (1)_4_ defines the direction of the plastic flow with $\dot{\lambda}\ge 0$ being the plastic multiplier and *g* denotes the plastic potential. For associated flow rule, the yield function and the plastic potential are equal (*g* = *f)*.

Isotropic hardening (i.e. a single scalar hardening variable) is widely used for description of the plastic behaviour under monotonic loadings or for modelling the plastic behaviour for processes that do not involve cyclic loading/unloading conditions. All participants assuming isotropic hardening, described it either by the Swift law [90], i.e.

3 $$Y\left({\overline{\varepsilon}}^\mathrm{p}\right)={K}_0{\left({\varepsilon}_0+{\overline{\varepsilon}}^\mathrm{p}\right)}^n$$

where *K*_0_, *ε*_0_ and *n* are material parameters, or by the Voce law (1948) [104], given by,

4 $$Y={Y}_0- Bexp\left(-n{\overline{\varepsilon}}^\mathrm{p}\right)$$

where *Y*_0_, *B* and *n* are parameters; ${\overline{\varepsilon}}^\mathrm{p}$ is the effective plastic strain being the work-conjugate of the effective stress. In addition, the team from USakarya used a Chaboche type hardening law (also called Armstrong-Frederick law) [17, 20, 21] involving both a scalar variable and a second-order tensorial variable **X** (combined isotropic and kinematic hardening law) for which the evolution law is given as:

5 $$\dot{\mathbf{X}}=C\left(2/3\right){\mathbf{D}}^\mathrm{p}-\gamma \mathbf{X}{\dot{\overline{\varepsilon}}}^\mathrm{p}$$

where *γ* and *C* are parameters that were determined from the cyclic shear tests.

Most participants assumed an associated flow rule, *f* = *g* in Eq. (1)_4_).

UAalto, UPorto, USiegen (see Table 11) used in conjunction with Hill’s yield function (see Eq. 12) a non-associated flow rule. As in [89], it was assumed that the flow potential *g* has the same mathematical form as Eq. (12), but it is characterized by different values for the anisotropy coefficients. In this manner, in the elastoplastic model, there are six anisotropy coefficients denoted as *F*_*σ*_, *G*_*σ*_, *H*_*σ*_, *L*_*σ*_, *M*_*σ*_, *N*_*σ*_ associated with the yield function and six additional anisotropy coefficients denoted as *F*_*r*_, *G*_*r*_, *H*_*r*_, *L*_*r*_, *M*_*r*_, *N*_*r*_, associated with the flow potential (see [89]). Evolution of the anisotropy coefficients involved in either yield function or potential flow with the equivalent plastic strain can also be also considered (see for example, [63] and “Hill48 and non-associated flow rule” Section).

### Orthotropic yield functions

Both 2-D orthotropic yield functions and general 3-D orthotropic yield functions that are applicable to any stress state were considered. The expressions of these yield functions are presented in “2-D orthotropic yield functions (Yld89, Yld2000-2D, HomPol4, HomPol6” and “3-D orthotropic yield functions (Hill48, CB2001, Yld2004-18p, CPB06ex2, Caz2018-Orth)” Sections, respectively, while details concerning the identification of these yield functions for the AA 6014-T4 are given in “Parameter identification and validation of phenomenological models” Section. In the following, (x, y, z) denotes the Cartesian coordinate system associated with the orthotropy axes; for a rolled sheet such as the material studied in this benchmark, x coincides with RD, y with TD and z is the normal direction to the sheet plane.

#### 2-D orthotropic yield functions (Yld89, Yld2000-2D, HomPol4, HomPol6)

The 2-D yield functions Yld89 and Yld2000-2D proposed by [4, 5] are extensions to orthotropy of the isotropic Hershey-Hosford yield function (see [45, 110]) which is expressed as:

6 $${\phi}_{H-H}\left({s}_1,{s}_2,{s}_3\right)={\left|{s}_1-{s}_2\right|}^m+{\left|{s}_2-{s}_3\right|}^m+{\left|{s}_3-{s}_1\right|}^m$$

where s_1_, s_2_, s_3_ denote the principal values of the stress deviator, and *m* is an exponent.

Specifically, Yld89 is a non-quadratic plane stress yield function containing a shear stress term,

7 $$a{\left|{k}_1+{k}_2\right|}^m+a{\left|{k}_1-{k}_2\right|}^m+\left(2-a\right){\left|2{k}_2\right|}^m=2{\overline{\sigma}}^m$$

and *k*_1_ = (*σ*_*xx*_ + *hσ*_*yy*_)/2 and ${k}_2=\sqrt{{\left({\sigma}_{xx}-h{\sigma}_{yy}\right)}^2/4+{p}^2{\sigma}_{xy}^2}$.

In the above equation, *a*, *h*, *p* are material parameters; the recommended values for the exponent *m* = 6 for body centred cubic (BCC) materials and *m* = 8 for face centred cubic (FCC) materials. In [4], in Eq. (7), a coefficient *c*=(2 − *a*) is sometimes introduced. In the identification of the model parameters by POSTECH, “…the anisotropy coefficients are indeed *a, h, p, m *(see Table 25 in “Non quadratic plane-stress yield locus Barlat (Yld89)” Section).

The Yld2000-2D yield function was defined as:

8 $${\left|{X}_1^{\prime }-{X}_2^{\prime}\right|}^m+{\left|2{X}_2^{{\prime\prime} }+{X}_1^{{\prime\prime}}\right|}^m+{\left|2{X}_1^{{\prime\prime} }+{X}_2^{{\prime\prime}}\right|}^m=2{\overline{\sigma}}^m$$

with ${X}_{1,2}^{\prime }=\frac{1}{2}\left({X}_{xx}^{\prime }+{X}_{yy}^{\prime}\pm \sqrt{{\left({X}_{xx}^{\prime }-{X}_{yy}^{\prime}\right)}^2+4{X^{\prime}}_{xy}^2}\right)$ and similar expression with the appropriate double prime indices for ${X}_{1,2}^{{\prime\prime} }$. The relations giving the components of **X**^′^ and **X**^″^in terms of the in-plane components of the Cauchy stress deviator are:

9 $$\left[\begin{array}{c}{X}_{xx}^{\prime}\\ {}{X}_{yy}^{\prime}\\ {}{X}_{xy}^{\prime}\end{array}\right]=\left[\begin{array}{ccc}{\alpha}_1& 0& 0\\ {}0& {\alpha}_2& 0\\ {}0& 0& {\alpha}_7\end{array}\right]\left[\begin{array}{c}{s}_{xx}\\ {}{s}_{yy}\\ {}{s}_{xy}\end{array}\right]\kern0.5em \mathrm{and}\kern0.75em \left[\begin{array}{c}{X}_{xx}^{{\prime\prime}}\\ {}{X}_{yy}^{{\prime\prime}}\\ {}{X}_{xy}^{{\prime\prime}}\end{array}\right]=\frac{1}{3}\left[\begin{array}{ccc}4{\alpha}_5-{\alpha}_3& 2{\alpha}_6-{\alpha}_4& 0\\ {}2{\alpha}_3-2{\alpha}_5& 4{\alpha}_4-{\alpha}_6& 0\\ {}0& 0& 3{\alpha}_8\end{array}\right]\left[\begin{array}{c}{s}_{xx}\\ {}{s}_{yy}\\ {}{s}_{xy}\end{array}\right]$$

where *α*_*j*_, j = 1…8 denote the independent anisotropy coefficients involved in the formulation of Yld2000-2D (see [5]).

The polynomial plane stress yield functions developed by [86] and called hereafter HomPol4 and HomPol6, respectively, are expressed as:

10 $${\displaystyle \begin{array}{c}\varphi ={a}_1{\sigma}_{xx}^4+{a}_2{\sigma}_{xx}^3{\sigma}_{yy}+{a}_3{\sigma}_{xx}^2{\sigma}_{yy}^2+{a}_4{\sigma}_{xx}{\sigma}_{yy}^3\\ {}+{a}_5{\sigma}_{yy}^4+\left({a}_6{\sigma}_{xx}^2+{a}_7{\sigma}_{xx}{\sigma}_{yy}+{a}_8{\sigma}_{yy}^2\right){\sigma}_{xy}^2\\ {}+{a}_9{\sigma}_{xy}^4\end{array}}$$

11 $${\displaystyle \begin{array}{c}\phi ={a}_1{\sigma}_{xx}^6+{a}_2{\sigma}_{xx}^5{\sigma}_{yy}+{a}_3{\sigma}_{xx}^4{\sigma}_{yy}^2+{a}_4{\sigma}_{xx}^3{\sigma}_{yy}^3\\ {}+{a}_5{\sigma}_{xx}^2{\sigma}_{yy}^4+{a}_6{\sigma}_{xx}{\sigma}_{yy}^5+{a}_7{\sigma}_{yy}^6+\\ {}\left({a}_8{\sigma}_{xx}^4+{a}_9{\sigma}_{xx}^3{\sigma}_{yy}+{a}_{10}{\sigma}_{xx}^2{\sigma}_{yy}^2+{a}_{11}{\sigma}_{xx}{\sigma}_{yy}^3+{a}_{12}{\sigma}_{yy}^4\right){\sigma}_{xy}^2\\ {}+\left({a}_{13}{\sigma}_{xx}^2+{a}_{14}{\sigma}_{xx}{\sigma}_{yy}+{a}_{15}{\upsigma}_{yy}^2\right){\sigma}_{xy}^4+{a}_{16}{\sigma}_{xy}^6\end{array}}$$

with *a*_*i*_ denoting anisotropy coefficients.

#### 3-D orthotropic yield functions (Hill48, CB2001, Yld2004–18p, CPB06ex2, Caz2018-Orth)

The orthotropic extension of the isotropic von Mises yield criterion was introduced by Hill [46]. The effective stress associated to Hill’s criterion is given by:

12 $$F{\left({\sigma}_{yy}-{\sigma}_{zz}\right)}^2+G{\left({\sigma}_{zz}-{\sigma}_{xx}\right)}^2+H{\left({\sigma}_{xx}-{\sigma}_{yy}\right)}^2+2\left(L{\sigma}_{yz}^2+M{\sigma}_{zx}^2+N{\sigma}_{xy}^2\right)={\overline{\sigma}}^2$$

where *F, G, H, L, M* and *N* are parameters describing the material anisotropy.

The orthotropic yield functions developed by [11, 12] are expressed in terms of the orthotropic invariants ${J}_2^0,{J}_3^0$. In this manner it is ensured that these formulations automatically satisfy the orthotropy requirements (i.e. correct combinations of stress components). The condition of independence of yielding on hydrostatic pressure is fulfilled and the anisotropy coefficients are independent. Specifically, the expressions of the orthotropic invariants were developed using rigorous representation theorems for tensor functions, imposing that they are respectively homogeneous polynomials of degree two, and three in stresses, pressure-insensitive, and for isotropy reduce to the isotropic invariants *J*_2_ and *J*_3_, respectively. In the coordinate system (*x*, *y*, *z*) associated with the orthotropy axes (i.e. RD, TD, ND), these orthotropic invariants are expressed as follows:

13 $${J}_2^o=\frac{a_1}{6}{\left({\sigma}_{xx}-{\sigma}_{yy}\right)}^2+\frac{a_2}{6}{\left({\sigma}_{yy}-{\sigma}_{zz}\right)}^2+\frac{a_3}{6}{\left({\sigma}_{xx}-{\sigma}_{zz}\right)}^2+{a}_4{\sigma}_{xy}^2+{a}_5{\sigma}_{xz}^2+{a}_6{\sigma}_{yz}^2$$

14 $${\displaystyle \begin{array}{l}{J}_3^o=\frac{1}{27}\left({b}_1+{b}_2\right){\sigma}_{xx}^3+\frac{1}{27}\kern0.24em \left({b}_3+{b}_4\right){\sigma}_{yy}^3\\\kern2em+\frac{1}{27}\kern0.24em \left[2\left({b}_1+{b}_4\right)-{b}_2-{b}_3\right]{\sigma}_{zz}^3\\ {}\;\kern1.5em -\frac{1}{9}\;\left({b}_1\kern0.1em {\sigma}_{yy}+{b}_2\;{\sigma}_{zz}\right){\sigma}_{xx}^2\\\kern2em-\frac{1}{9}\;\left({b}_3\kern0.20em {\sigma}_{zz}+{b}_4\;{\sigma}_{xx}\right){\sigma}_{yy}^2\\ {}\begin{array}{l}\kern1.85em -\frac{1}{9}\;\left[\left({b}_1-{b}_2+{b}_4\right)\kern0.1em {\sigma}_{xx}+\left({b}_1-{b}_3+{b}_4\right)\;{\sigma}_{yy}\right]{\sigma}_{zz}^2\\ {}\kern1.6em +\frac{2}{9}\left({b}_1+{b}_4\right)\kern0.1em \;{\sigma}_{xx}\;{\sigma}_{zz}{\sigma}_{yy}\\\kern1.8em-\frac{\sigma_{xz}^2}{3}\kern0.24em \left[2\kern0.20em {b}_9\;{\sigma}_{yy}-{b}_8\;{\sigma}_{zz}-\left(2\kern0.1em {b}_9-{b}_8\right)\;{\sigma}_{xx}\right]\\ {}\begin{array}{l}\kern1.7em -\frac{\sigma_{xy}^2}{3}\;\left[2\kern0.1em \;{b}_{10}\;{\sigma}_{zz}-{b}_5\kern0.1em {\sigma}_{yy}-\left(2\kern0.1em {b}_{10}-{b}_5\right)\kern0.1em {\sigma}_{xx}\;\right]\\\kern1.7em-\frac{\sigma_{y\kern0.1em z}^2}{3}\kern0.24em \left[\left({b}_6+{b}_7\right)\;{\sigma}_{xx}-{b}_6{\sigma}_{yy}-{b}_7{\sigma}_{zz}\;\right]\\ {}\kern1.5em +2\kern0.20em {b}_{11}{\sigma}_{xy}{\sigma}_{xz}{\sigma}_{yz}\end{array}\end{array}\end{array}}$$

where *a*_*k*_ (*k* = 1…6) and *b*_*j*_ (*j* = 1…11) are anisotropy coefficients (see book [16]).

The yield function [12], denoted hereafter as CB2001 is of the form:

15 $$f={\left({J}_2^o\right)}^3-c{\left({J}_3^o\right)}^2$$

where *c* is a parameter. For isotropic conditions (i.e. all anisotropy coefficients set equal to unity), the isotropic yield function proposed by Drucker [28] is recovered.

The effective stress according to the orthotropic yield function of [11], called hereafter Cazacu2018-Orth is expressed as:

16 $$\overline{\sigma}=B{\left[{\left({J}_2^o\right)}^4-\alpha \left({J}_2^o\right){\left({J}_3^o\right)}^2\right]}^{1/8}$$

with *α* being a parameter and *B* defined such as for uniaxial tension in the *x*-direction the effective stress reduces to the yield stress, i.e.:

17 $$B=\frac{3\sqrt{2}}{{\left\{\left[27{\left({a}_1+{a}_3\right)}^3-8\alpha {\left({b}_1+{b}_2\right)}^2\right]\left(3{a}_1+3{a}_3\right)\right\}}^{1/8}}$$

A 3-D orthotropic extension of Hershey-Hosford isotropic yield function given by Eq. (6) is the yield function Yld2004–18p proposed in [7]:

18 $$\phi \left({s}_{\alpha \beta}\right)=\Phi \left(\tilde{s}_{i}^{\prime },\tilde{s}_{j}^{{\prime\prime}}\right)=\sum_{i,j}^{1,3}{\left|\tilde{s}_{i}^{\prime }-\tilde{s}_{j}^{{\prime\prime}}\right|}^m=4{\overline{\sigma}}^m$$

In the above equation $\tilde{s}_{i}^{\prime }-\tilde{s}_{j}^{{\prime\prime}}$ being the principal values of two transformed stress deviators $\tilde{\mathbf{s}}^{\prime }={\mathbf{C}}^{\prime}\mathbf{s}$ and $\tilde{\mathbf{s}}^{{\prime\prime}}={\mathbf{C}}^{{\prime\prime}}\mathbf{s}$. These transformed tensors are written in a matrix form as:

19 $$\widetilde s\equiv\begin{bmatrix}{\widetilde s}_{xx}\\{\widetilde s}_{yy}\\{\widetilde s}_{zz}\\{\widetilde s}_{yz}\\{\widetilde s}_{zx}\\{\widetilde s}_{xy}\end{bmatrix}\begin{bmatrix}0&-c_{12}&-c_{13}&0&0&0\\-c_{21}&0&-c_{23}&0&0&0\\-c_{31}&-c_{32}&0&0&0&0\\0&0&0&c_{44}&0&0\\0&0&0&0&c_{55}&0\\0&0&0&0&0&c_{66}\end{bmatrix}\begin{bmatrix}s_{xx}\\s_{yy}\\s_{zz}\\s_{yz}\\s_{zx}\\s_{xy}\end{bmatrix}$$

with the appropriate symbols (prime and double prime) for each transformation, i.e., ${C}_{ij}^{\prime }$ for $\tilde{s}^{{\prime}}$ and ${C}_{ij}^{{\prime\prime} }$ for $\tilde{s}^{{\prime\prime}}$ (for more details, see [7]). Note that the two tensors $C^{\prime}$, and ${C}^{\prime\prime}$  are not symmetric (${C}_{ij}^{\prime}\ne {C}_{ji}^{\prime }$ and ${C}_{ij}^{{\prime\prime}}\ne {C}_{ji}^{{\prime\prime} }$).

To account for yielding asymmetry between tension and compression associated either with deformation twinning or non-Schmid effects at single crystal level, in [14] it was proposed an isotropic criterion of the form:

20 $$G\left({s}_1,{s}_2,{s}_3,k,a\right)={\left(\left|{s}_1\right|-{ks}_1\right)}^a+{\left(\left|{s}_2\right|-{ks}_2\right)}^a+{\left(\left|{s}_3\right|-{ks}_3\right)}^a$$

where *k* is a parameter. Furthermore, this isotropic yield criterion was further extended to orthotropy by applying a fourth-order symmetric and orthotropic tensor **C** on the stress deviator **s**, i.e. in Eq. (20), *s*_1_, *s*_2_, and *s*_3_ were substituted by the principal values of the transformed tensor **Σ = Cs**. Thus, the resulting anisotropic yield criterion, denoted, CPB06 is of the form:

21 $${F}_1={\left(\left|{\Sigma}_1\right|-k{\Sigma}_1\right)}^a+{\left(\left|{\Sigma}_2\right|-k{\Sigma}_2\right)}^a+{\left(\left|{\Sigma}_3\right|-k{\Sigma}_3\right)}^a={\overline{\sigma}}^a$$

where Σ_*i*_ are the principal values of and $\overline{\sigma}$ is the effective stress associated with this criterion. If two linear transformations operating on the Cauchy stress deviator **s** are considered, namely **Σ = Cs** and Σ^′^ **= C**^′^**s** with **C **and **C**^′^ being symmetric and orthotropic, the general form of the orthotropic criterion, called CPB06ex2, is:

22 $${F}_2=G\left({\Sigma}_1,\ {\Sigma}_2,\ {\Sigma}_3,\ k,\ a\right)+G\left({\Sigma}_1^{\prime },\ {\Sigma}_2^{\prime },\ {\Sigma}_3^{\prime },\ {k}^{\prime },\ a\right)={\overline{\sigma}}^a$$

where *k*, *k*^′^ are parameters and Σ_*i*_ and Σ_*i*_^′ ^the principal values of the respective transformed tensors (for more details, see [72]).

### Crystal plasticity based constitutive models

To describe the macroscopic plastic anisotropy of a polycrystalline metallic material, in crystal plasticity based constitutive models, the deformation of the constituent crystals is explicitly simulated. Generally, for FCC materials, the plastic deformation of the crystals is modelled with the Schmid law, i.e. it is assumed that a critical value of the resolved shear stress is required for the initiation of the slip. Furthermore, the same critical resolved shear stress is considered for all twelve slip systems (e.g. see review of [97]). The slip rate on each slip system is generally described by a power-law (e.g. see Eq. 35). Recently, a new single-crystal law [15] called hereafter “Caz2018singlecrys” that is defined for any stress-state was developed (see Eq. 28–30).

To obtain the macroscopic stress-strain response of the polycrystal from the response of the individual constituent crystals, different assumptions are made among the Benchmark participants:

- the homogeneous strain assumption of Taylor [91] referred hereafter as the Full Constraints Taylor model is used in the models developed by REEF (“Caz2018polycrys, a polycrystalline model based on Cazacu single crystal law Caz2018singlecrys” Section), ULiege (“Minty model, an interpolation approach” Section), NTNU (“Crystal plasticity model used in DAMASK solver” Section);
- more relaxed grain interaction constraints, such in the ALAMEL model [98] is applied by KUL (see “Facet-3D model linked to ALAMEL crystal plasticity model” Section);
- a self-consistent homogenization method (see “Viscoplastic self consistent model (VPSC)” Section) is used by UGent.

Finally, the simplified approach of POSTECH is to assume that only the cubic component is present in the texture of the AA 6016-T4 material (i.e. 100% of the crystals are oriented along the <100> crystallographic directions) and use the cubic single-crystal law of Cazacu [15] to model the yielding behaviour of the polycrystalline material.

#### Facet-3D model linked to ALAMEL crystal plasticity model

The FACET approach presented in [99] defines a phenomenological yield surface in the stress space based on rates of plastic work per unit volume, obtained with the ALAMEL model. The Facet expression for this plane-stress yield surface in stress space is given by [99]:

23 $$\phi \left(\boldsymbol{s}\right)={\left(\sum\textstyle_{k=1}^m{c}_k{\left(\boldsymbol{s}.{\boldsymbol{a}}_k\right)}^n\right)}^{\frac{1}{n}}=1$$

where for Facet-3D, which is a generalized plane stress model, ***s*** is a 3D stress vector such that $$\boldsymbol{s}={\left[\begin{array}{ccc}{s}_1& {s}_2& {s}_3\end{array}\right]}^{\mathrm{T}}$$ in which ${s}_1=\frac{1}{\sqrt{2}}\left({s}_x-{s}_y\right)$, ${s}_2=\sqrt{\frac{3}{2}}\left({s}_x+{s}_y\right)$, and ${s}_3=\sqrt{2}{s}_{xy}$, with *s*_*x*_, *s*_*y*_, and *s*_*xy*_ being the components of stress deviator. *m* represents the number of Facet terms, *n* is the order of the Facet expression, *c*_*k*_ are coefficients to be determined during the fitting procedure, and ***a***_*k*_ are normal vectors to hyperplanes or “facets” in 3D stress space to ensure convexity of the yield surface. The order *n* should be an even number, and the coefficients *c*_*k*_ must be non-negative.

The 3D plastic strain rate vector $${\dot{\boldsymbol{e}}}^{\mathrm{P}}={\left[\begin{array}{ccc}{\dot{e}}_1^{\mathrm{P}}& {\dot{e}}_2^{\mathrm{P}}& {\dot{e}}_3^{\mathrm{P}}\end{array}\right]}^{\mathrm{T}}$$ that corresponds to a given yield stress is calculated by:

24 $${\dot{\boldsymbol{e}}}^{\mathrm{P}}=\dot{\lambda}\frac{\partial \phi \left(\boldsymbol{s}\right)}{\partial \boldsymbol{s}}=\dot{\lambda}{\left(\sum\textstyle_{k=1}^m{c}_k{\left(\boldsymbol{s}.{\boldsymbol{a}}_k\right)}^n\right)}^{\frac{1}{n}-1}\ \sum\textstyle_{k=1}^m{c}_k{\left(\boldsymbol{s}.{\boldsymbol{a}}_k\right)}^{n-1}{\boldsymbol{a}}_k$$

in which $\dot{\lambda}$ is an arbitrary non-negative number, called the plastic multiplier. Note that rate-insensitivity is assumed. The components of the plastic strain rate vector are then calculated by:

25 $${\displaystyle \begin{array}{c}{\dot{e}}_x^{\mathrm{P}}=\frac{1}{\sqrt{2}}{\dot{e}}_1^{\mathrm{P}}+\frac{1}{\sqrt{6}}{\dot{e}}_2^{\mathrm{P}}\\ {}{\dot{e}}_y^{\mathrm{P}}=-\frac{1}{\sqrt{2}}{\dot{e}}_1^{\mathrm{P}}+\frac{1}{\sqrt{6}}{\dot{e}}_2^{\mathrm{P}}\\ {}\begin{array}{c}{\dot{e}}_z^{\mathrm{P}}=-{\dot{e}}_x^{\mathrm{P}}-{\dot{e}}_y^{\mathrm{P}}\\ {}{\dot{e}}_{xy}^{\mathrm{P}}=\frac{1}{\sqrt{2}}{\dot{e}}_3^{\mathrm{P}}\end{array}\end{array}}$$

The Facet expression is further used within a VUMAT user material routine of ABAQUS/Explicit [35] for the cup drawing simulations. As already mentioned, and further explained in “Facet-3D model” Section, for this benchmark the ALAMEL model was used to identify the Facet-3D yield locus. ALAMEL [98] is a statistical crystal plasticity model that accounts for short-range interaction of the grains. In ALAMEL model, pairs of grains are considered as a cluster with a common boundary plane of a certain orientation. While in the full constraints Taylor model all the grains are assumed to undergo the same velocity gradient as that imposed on the aggregate of crystals (a.k.a. the macroscopic deformation), in ALAMEL certain deviations from the macroscopic deformation are allowed for each pair of grains.

In particular, two shear strain components parallel to the boundary plane of each pair of grains are relaxed from the full constrains Taylor theory. If the macroscopic velocity gradient is given by ***L***, then the local velocity gradient ***l*** is calculated by:

26 $${\boldsymbol{l}}_i=\boldsymbol{L}+{\left(-1\right)}^i\sum_{j=1}^2{\boldsymbol{T}}_j^{\mathrm{R}}{\dot{\gamma}}_j^{\mathrm{R}}$$

where *i* = 1, 2 represents the number of grain in the cluster, ${\dot{\gamma}}^{\mathrm{R}}$ is a “cooperative” shear deformation which is equal for both grains and operates on “pseudo slip systems”, and ***T***^R^ are relaxation matrices which in the local coordinate system with a normal in *z* direction are defined as:

27 $${\displaystyle \begin{array}{c}{\boldsymbol{T}}_1^{\mathrm{R}}=\left[\begin{array}{ccc}0& 0& 1\\ {}0& 0& 0\\ {}0& 0& 0\end{array}\right]\\ {}{\boldsymbol{T}}_2^{\mathrm{R}}=\left[\begin{array}{ccc}0& 0& 0\\ {}0& 0& 1\\ {}0& 0& 0\end{array}\right]\end{array}}$$

More details about ALAMEL and its comparison with Taylor and other models can be found in [98]. For the virtual experiments with ALAMEL, the latent hardening of the grains was neglected. Therefore, only the texture data was necessary to generate 10,000 orientations to assign to the grains. These grains were then randomly grouped into 5000 clusters to make the input for ALAMEL. In the present work, the virtual tests are performed with the initial texture, assuming identical critical resolved shear stresses for all slip systems in all grains. No hardening is considered at the microscopic scale. The virtual yield stresses define the shape of the yield locus. This is normalized to yield stress of one for the tensile test in the rolling direction. Further scaling of the yield locus to account for hardening is done at the macroscopic level (i.e. the FE simulations), where isotropic hardening is assumed using the Swift hardening law described in “Identification of hardening laws and data used” Section (see Table 32).

#### Caz2018polycrys, a polycrystalline model based on Cazacu single crystal law Caz2018singlecrys

The polycrystal is represented by a finite set of grains characterized by orientation and volume fraction to reproduce the material texture. Elastic deformations are modelled using Hooke’s law for the type of symmetry shown by cubic crystals.

With Caz2018polycrys law, the plastic behaviour of the constituent crystals is modelled using the single crystal law of [15], called Caz2018singlecrys, normality rule, and isotropic hardening described by a Swift-type law. The single-crystal law is defined for any stress-state. It is written in terms of cubic stress-invariants that were deduced using rigorous theorems of representation of tensor functions (see Eq. 28–30). Consequently, the exact number of anisotropy coefficients that ought to be involved in the formulation, in order to satisfy the symmetries of the cubic lattice and the condition of insensitivity of plastic deformation to hydrostatic pressure are satisfied (for full mathematical proofs and further details, see [15] and book [16]).

Relative to the Cartesian coordinate system O*x*_1_*x*_2_*x*_3_ associated with the crystal axes (i.e., the <100> crystal directions), the expression of the cubic invariants are:

28 $${\displaystyle \begin{array}{l}{J}_2^\mathrm{c}=\frac{1}{2}{m}_1\left({s}_{11}^2+{s}_{22}^2+{s}_{33}^2\right)+{m}_2\left({s}_{12}^2+{s}_{13}^2+{s}_{23}^2\right)\\ {}{J}_3^\mathrm{c}={n}_1{s}_{11}{s}_{22}{s}_{33}-{n}_3\left({s}_{33}{s}_{12}^2+{s}_{11}{s}_{23}^2+{s}_{22}{s}_{13}^2\right)+2{n}_4{s}_{12}{s}_{13}{s}_{23}\end{array}}$$

The single crystal law writes,

29 $${\left({J}_2^\mathrm{c}\right)}^3-c{\left({J}_3^\mathrm{c}\right)}^2={k}^6$$

the effective stress of the crystal, ${\overline{\sigma}}_{\text{grain}}$, being given by:

30 $${\overline{\sigma}}_\text{grain}=\frac{3}{{\left(27-4c{n}_1^2\right)}^{1/6}}{\left[{\left({J}_2^{\mathrm{c}}\right)}^3-c{\left({J}_3^\mathrm{c}\right)}^2\right]}^{1/6}$$

where ***s*** denotes the deviator of the applied Cauchy stress, *k* is the yield limit in simple shear *m*_1_, *m*_2_, *n*_1_, *n*_3_, *n*_4_ are anisotropy coefficients and *c* is a parameter that describes the relative importance of the second-order and third-order cubic stress-invariants on yielding. The plastic strain-rate of each crystal ${\mathbf{d}}_{\text{grain}}^{\mathrm{p}}$ is uniquely defined for any stress state and can be easily calculated as:

31 $$\mathbf{d}^{\mathrm{p}}=\dot{\lambda}\frac{\partial {\overline{\sigma}}_{\text{grain}}}{\partial {\boldsymbol{\sigma}}}$$

where $\dot{\lambda}$ is the plastic multiplier, and ${\overline{\sigma}}_{\text{grain}}$ is given by Eq. (30).

While all the simulations presented hereafter were done using Caz2018polycrys polycrystalline model implemented in the commercial FE solver ABAQUS Standard (implicit solver), the same approach can be used to do calculations in any FE solver. The model is called Caz2018polycrys, as already introduced in Table 11, to have a clear distinction with Caz2018singlecrys.

A polycrystalline aggregate is associated with each FE integration point. The FE code provides the deformation gradient at the integration point. The elasto-plasticity problem is solved in each grain (crystallite level). The orientation, the equivalent plastic strain and stress of the individual grains are updated depending on the deformation of the element, and the calculated individual crystallite stresses are homogenized to give the stress at the integration point, for use in the solution of the continuum equilibrium equations. The isostrain homogenization scheme is used (the total strain-rate of each grain is equal to the overall strain-rate **D**).

It is considered that the total strain-rate of each grain belonging to a given element is equal to the overall strain-rate **D**. At the time increment *(n)*, the stress in each grain is computed by solving the governing equations, namely:

32 $${\displaystyle \begin{array}{l}\mathbf{D}_{\text{grain}}^{\left(n\right)}={\left(\mathbf{R}^{\left(n\right)}\right)}^{\mathrm{T}}\mathbf{D}^{\left(n\right)}\mathbf{R}^{\left(n\right)}\\ {}\mathbf{D}_{\text{grain}}^{\left(n\right)}=\mathbf{D}_{\text{grain}}^{\mathrm{e}\left(n\right)}+\mathbf{D}_{\text{grain}}^{\mathrm{p}\left(n\right)}\\ {}{\boldsymbol{\sigma}}_{\text{grain}}^{(n)}={\boldsymbol{\sigma}}_{\text{grain}}^{\left(n-1\right)}+\left(\mathbf{C}^{\mathrm{e}}{\text{:}}\ \mathbf{D}_{\text{grain}}^{\mathrm{e}\left(n\right)}\right)\mathrm{d}t\\ {}{\overline{\sigma}}_{\text{grain}}^{(n)}-Y\left({\overline{\varepsilon}}_{\text{grain}}^{\mathrm{p}\left(n\right)}\right)\le 0\\ {}\mathbf{D}_{\text{grain}}^{\mathrm{p}\left(n\right)}={\dot{\lambda}}_{\text{grain}}\frac{\partial {\overline{\sigma}}_{\text{grain}}^{(n)}}{\partial{\boldsymbol{\sigma}}}\end{array}}$$

where ${\mathbf{D}}_{\text{grain}}^{\left(n\right)}$, ${\mathbf{D}}_{\text{grain}}^{\mathrm{p}\left(n\right)}$ and ${\mathbf{D}}_{\text{grain}}^{\mathrm{e}\left(n\right)}$ are respectively the total strain-rate, the plastic and elastic strain-rate, ${\boldsymbol{\upsigma}}_{\text{grain}}^{\left(n-1\right)}$ and ${\boldsymbol{\upsigma}}_{\text{grain}}^{(n)}$ are the stress tensors at the beginning and end of the increment, respectively, $Y\left({\overline{\varepsilon}}_{\text{grain}}^{p\left({n}\right)}\right)$ is the hardening law while ${\overline{\varepsilon}}_{\text{grain}}^{p\left({n}\right)}$ is the equivalent plastic strain in the given grain.

The stress of the polycrystal at the end of the increment is given by:

33 $$\boldsymbol{\sigma}^{(n)}=\left(\sum_{\mathrm{i}}{w}_{\mathrm{i}}\mathbf{R}_{\mathrm{i}}^{\left(\mathrm{n}\right)}{\left({\boldsymbol{\sigma}}_\text{grain}^{(n)}\right)}_{\mathrm{i}}{\left({\mathbf{R}}_{\mathrm{i}}^{\left(\mathrm{n}\right)}\right)}^\mathrm{T}\right)/\left({\sum}_{\mathrm{i}}{w}_{\mathrm{i}}\right)$$

where ${\left({\boldsymbol{\upsigma}}_{\text{grain}}^{(n)}\right)}_{\mathrm{i}}$ is the stress tensor of grain *i*, and ${\mathbf{R}}_{\mathrm{i}}^{\left(\mathrm{n}\right)}$ is the transformation matrix for passage from the crystal axes of grain *i* to the loading frame axes, while *w*_i_ is the weight of the grain *i*. Note that to increase readability, in Eqs. (32) the index ‘*i*’, designating the local field variables associated to the grain *i,* has been dropped.

#### Minty model, an interpolation approach

The main specificity of the Minty model is that it uses a local yield locus approach based on a direct stress-strain interpolation method. In this respect, it does not use a classical yield locus formulation neither for the interpolation nor in the stress integration scheme. Instead, a linear stress-strain interpolation is employed:

34 $$\boldsymbol{\sigma} =\tau \boldsymbol{Cu}$$

In the above equation, ***σ ***is a 5-D vector containing the deviatoric part of the stress, its hydrostatic part being elastically computed according to Hooke’s law. The 5-D vector ***u*** is the deviatoric plastic strain rate direction; it is a unit vector. *τ* is the critical resolved shear stress describing the work hardening according to the Swift type exponential relationship of Eq. (35), where the strength coefficient *K*, the offset *Г*^0^ and the hardening exponent *n* are material parameters, fitted to experimental data (further explanations in “Minty law” Section) and *Г* is the accumulated polycrystal induced slip.

35 $$\tau =K{\left({\varGamma}^0+\varGamma \right)}^n$$

The stress-strain interpolation is included in the matrix ***C*** of Eq. (34). For its construction, 5 directions: u_*i*_ (*i* = 1…5) in the deviatoric strain rate space are advisedly chosen. The associated deviatoric stresses: s_*i*_ (*i* = 1…5) are computed by a full constraints Taylor’s model. These stress vectors lie on the yield surface according to Taylor’s model. These points define the interpolation domain; they are located at the vertices of the domain and are called ‘stress nodes’. The mathematical details about the construction of the ***C*** matrix from the 5 stress nodes can be found in [40, 41].

With this method, only a small part of the yield locus is known. As long as the interpolation is achieved in the domain delimited by the 5 stress nodes, the interpolation matrix ***C*** is valid. When the stress direction explored during the finite element computation falls out of the domain, updating of the stress nodes must take place; a new interpolation matrix is computed. The classical updating method consists in finding 5 new stress nodes defining a new domain containing the current stress direction. An enhanced updating method makes use of the adjacent domain. Therefore, only 1 new stress node is computed with Taylor’s model and 4 of the 5 old stress nodes are kept for the interpolation. The main advantages of this method are that updating requires only 1 (instead of 5) call to Taylor’s model and that it improves the continuity of the resulting yield locus and the continuity of its normal.

The texture of the material is represented at each integration point of the finite element mesh by a set of crystallographic orientations. Texture evolution can be computed at each integration point on the basis of the strain history using Taylor’s model. To include its effects in the computations, the interpolation matrix must be updated when texture evolution took place.

#### Crystal plasticity model used in DAMASK solver

The usual constitutive assumptions are considered. Namely, it is assumed a local multiplicative decomposition of the deformation gradient **F** = **F**_**e**_**F**_**p**_, where **F**_**e**_ and **F**_**p**_ are elastic and plastic component, respectively. The evolution of deformation gradients is obtained from **L** = **L**_**e**_ + **F**_**e**_**L**_**p**_(**F**_**e**_)^−**1**^. It is assumed that plastic deformation is due to slip over N slip systems. Each deformation system (α) is characterized by the unit vectors **n**^*α*^ (normal to the slip plane) and a vector **b**^*α*^ (shear direction), their dyadic product defining the Schmid tensor of the system, ${\mathbf{S}}_0^{\alpha }$. It is considered that the plastic velocity gradient **L**_**p**_ is expressed as:

36 $${\mathbf{L}}_{\mathbf{p}}={\dot{\mathbf{F}}}_{\mathbf{p}}{\mathbf{F}}_{\mathbf{p}}^{-\mathbf{1}}=\sum_{\alpha}^N{\dot{\gamma}}^{\alpha }{\mathbf{S}}_0^{\alpha }$$

where ${\dot{\gamma}}^{\alpha }$ is the shear on the slip system (α).

The usual power-law [71, 87] is used to define the plastic shear rate ${\dot{\gamma}}^{\alpha }$:

37 $${\dot{\gamma}}^{\alpha }={\dot{\gamma}}^0{\left|\frac{\tau_{\alpha }}{g^{\alpha }}\right|}^{1/m}\operatorname{sign}\left({\tau}_{\alpha}\right)$$

where *τ*_*α*_ is the applied shear stress, ${\dot{\gamma}}^0$ is a reference shear strain rate and the exponent *m* defines the rate sensitivity of slip system, *g*^*α*^ is the slip resistance which accounts for the strain hardened state of the crystal in the current configuration [9]. The evolution of *g*^*α*^ is governed by:

38 $${\dot{g}}^{\alpha }=\sum_{\beta}^N{h}_{\alpha \beta}{\dot{\gamma}}^{\beta }{g}^{\alpha }$$

where *g*^*α*^(0) = *τ*_0_ is the initial hardness, assumed the same for each slip system and *h*_*αβ*_ is the instantaneous strain hardening matrix, which is given by:

39 $${h}_{\alpha \beta}={h}_0\left[q+\left(1-q\right){\delta}_{\alpha \beta}\right]\left|1-\frac{g^{\beta }}{g_{\infty }}\right|\mathit{\operatorname{sign}}\left(1-\frac{g^{\beta }}{g_{\infty }}\right)$$

where *q* is a latent hardening parameter. For a coplanar slip system *q*=1, and for a non-coplanar slip system *q*=1.4, where *h*_0_, α and *g*_∞_ are hardening parameters.

The applied shear stress *τ*_*α*_ is related to the Cauchy stress tensor **σ** as [75]:

40 $${\tau}_{\alpha }=\boldsymbol{\sigma} :\left({\mathbf{F}}_{\mathbf{e}}{\mathbf{S}}_0^{\alpha }{\mathbf{F}}_{\mathbf{p}}^{-\mathbf{1}}\right)=\left({\mathbf{F}}_{\mathbf{e}}^{\mathrm{T}}{\mathbf{F}}_{\mathbf{e}}\mathbf{S}\right):{\mathbf{S}}_0^{\alpha }$$

where **S** is the second-order Piola-Kirchhoff stress calculated using the small elastic strain assumption:

41 $$\mathbf{S}=\mathbf{C}:{\mathbf{E}}_e\ \mathrm{with}\ {\mathbf{E}}_e=\left({\mathbf{F}}_{\mathbf{e}}^{\mathrm{T}}{\mathbf{F}}_{\mathbf{e}}-\mathbf{I}\right)/\mathbf{2}$$

where **C** is the elasticity matrix in the sample coordinate system. With respect to the crystal axes, this matrix for the case of a cubic crystal is fully specified by three material constants, C11, C12 and C44.

This model has been implemented into the open-source spectral method solver DAMASK [76]. Details on the numerical spectral method using the Fast Fourier transformation (CPFFT) can be found in Shanthraj et al. [81]. For this benchmark, DAMASK is used to predict yield stress points in both in-plane and out-of-plane uniaxial loadings and other multi-axial loadings.

#### Viscoplastic self consistent model (VPSC)

The viscoplastic self-consistent (VPSC) model, developed by Lebensohn and Tomé [60], uses a mean-field approach for the simulation of plastic deformation of polycrystals. Each grain is treated as an anisotropic, viscoplastic, ellipsoidal inclusion embedded in a uniform matrix having the unknown properties (to be determined) of the polycrystal. In this work, the VPSC90 implementation [32] is employed. VPSC90 differs from the original VPSC version [60] in that it also considers elastic deformation and uses a different algorithm to find the self-consistent solution. The main assumptions are as follows:

1. Grain constitutive equation: the plastic strain rate of each grain is calculated using the strain rate sensitivity approach of Asaro and Needleman [2], which correlates the plastic strain rate of the grain, ${\dot{\varepsilon}}_g$, with its stress, *σ*_grain_, through the relationship:

42 $$\mathbf{D}_{\text{grain}}^{\mathrm{p}}=\sum_s{\mathbf{m}}^s\;{\dot{\gamma}}^s={\dot{\gamma}}_o\;\sum_s{\mathbf{m}}^s{\left(\frac{\mid {\mathbf{m}}^s:{\boldsymbol{\sigma}}_\text{grain}\mid }{\tau_\mathrm{c}^s}\right)}^{n_s}\kern0.24em \operatorname{sgn}\left({\mathbf{m}}^s:{\boldsymbol{\sigma}}_\text{grain}\right)$$

where ${\dot{\upgamma}}^{\mathrm{s}}$, **m**^s^, and *n*_*s*_ are respectively, the shear rate, the symmetric part of the Schmid tensor, the strain-rate sensitivity exponent of the slip system (s), and ${\dot{\gamma}}_0$ is a normalization constant (usually taken equal to unity). The critically resolved shear stress of the slip system (*s*), ${\tau}_c^s$, is calculated according to an extended Voce-type hardening law [93],

43 $${\tau}_c^s\left(\Gamma \right)={\tau}_0^s+\left({\tau}_1^s+{\theta}_1^{\mathrm{s}}\ \Gamma \right)\left(1-\exp \left(-\Gamma \left|\frac{\theta_0^s}{\tau_1^s}\right|\right)\right)$$

where Γ is the accumulated shear in all slip systems and *τ*_0_, *τ*_1_, *θ*_0_ and *θ*_1_ are parameters.

2) Micro-macro relationships: the overall stress and strain are calculated as the weighted averages over all the constituent grains:

44 $$\mathbf{D}=\sum_g{w}_g{\mathbf{D}}_\text{grain}^\mathrm{p}$$

45 $$\boldsymbol{\upsigma} =\sum_g{w}_g{\boldsymbol{\upsigma}}_\text{grain}$$

with *w*_*g*_ the volumetric fraction of grain *g*.

3) Interaction equation: the Eshelby inclusion problem [30, 60] is solved and the grain interaction tensor ${\tilde{M}}_g$ is found. This tensor is used to correlate the overall (polycrystal) and grain level stresses and strains:

46 $$\mathbf{D}- {\mathbf{D}}_{\text{grain}}^\mathrm{p}=-{\tilde{\mathbf{M}}}_g\left(\boldsymbol{\upsigma} -{\boldsymbol{\upsigma}}_{\text{grain}}\right)$$

In addition, the obtained solution must fulfil the boundary conditions of the problem. At the macroscopic level, it is imposed that certain components of the stress or total strain (adding the elastic contribution) reach predefined values. The solution is found using an iterative algorithm, which is based on the gradient descent method in VPSC90. Namely, the total deformation of the polycrystal is obtained by imposing successive strain increments and calculating the resulting shears in the active deformation systems in the grains. The final texture is given by the grain reorientations associated with these shears.

## Parameter identification and validation of constitutive models

Since all the plasticity anisotropic constitutive laws are non-linear, the determination of the parameters involved in the models often needs to be done in an iterative way and in general, multiple sets of parameters (i.e. solutions) can be obtained for the same law. The identification methodology involves minimizing the error between theoretical predictions and experimental data. To eliminate parametrizations that are not valid, additional constraints can be imposed to ensure that the yield surface is well-defined (e.g. the transformations tensors should not be singular) and convex.

The benchmark committee has provided a set of parameters for several orthotropic yield criteria. Specifically, POSTECH organizer supplied the values of the parameters for Yld2000-2D and Yld2004–18p (see Table 27 and 28); REEF supplied the values of the Caz2018-Orth yield criterion (see Table 30); UCoimbra supplied the parameters values for Hill 48 yield criterion (stress- *r*-based identification, see Table 22). The benchmark committee has also supplied the parameter values for the Swift and Voce isotropic hardening law (Table 32). Participants could use these material parameters or chose to perform their own identification based on experimental data of “Material experimental characterization” Section. Some participants selected other 2-D and 3-D yield criteria and/or models (see Table 11).

In this section, the ESAFORM benchmark organizers and participants have provided the information about their parameter identification methodology. In their procedure, they used mechanical tests of “Material experimental characterization” Section or virtual ones, simulated with their crystal plasticity laws. The parameter values provided in this section are referenced when presenting and discussing the FE cup drawing results of “Cup drawing simulations, analysis and discussion” Section.

Note that each participant had to provide the set of parameters used and to show the predictions of the Lankford coefficients, elastic limits (yield stresses), and the yield locus cross-section in the (RD-TD) plane. In this manner, the accuracy of the parameterization and the capacity of the models to describe the basic features of the plastic behaviour of the material can be evaluated. For the yield functions that have analytic expressions, the variation in yield stresses, Lankford coefficients and respectively yield surfaces was simply done by using the respective equations presented in “Orthotropic yield functions” Section. For the polycrystalline multi-scale models, these properties can be calculated only numerically.

Furthermore, to verify the implementation of the various orthotropic yield functions in the respective FE codes, participants were asked to provide the Lankford coefficients calculated by performing uniaxial tensile tests, and to check the numerical *r*-values vs. the analytical ones.

Finally by calculating with the same code both tensile tests and cup drawing tests, it was possible to ascertain whether the respective models can capture the experimentally observed correlations between the anisotropy in Lankford coefficients and the earing profile. Moreover, this approach helps to differentiate between the effects on the predictions associated with the choice of FE elements and FE mesh and those associated with the choice of the constitutive model and parametrization used.

“Applied identification and validation methodology for crystal plasticity models” Section presents the methodology used for identification and verification of polycrystalline models. “Parameter identification and validation of phenomenological models” Section details the procedures used to identify the orthotropic yield functions. The approach adopted to identify the hardening law is described in “Identification of hardening laws and data used” Section while “Discussion on the methodology for identification of the models” Section points the effort required in all these identification steps.

### Applied identification and validation methodology for crystal plasticity models

For any polycrystalline plasticity model, the texture data is an input. As underlined hereafter, the description of the texture derived from the same EBSD data (“Material and initial texture” Section) can already present significant variations. So each team describes in “Representative textures and microstructures” Section, its methodology to obtain the representative texture. “Parameter identification, validation and/or use of crystal plasticity models” Section provides the parameter identification method and verifies the parameterizations.

#### Representative textures and microstructures

##### Texture description for ALAMEL

KUL team uses the so-called ALAMEL crystal plasticity model to generate data exploited to identify the material parameter set of their Facet-3D yield locus (see “Facet-3D model linked to ALAMEL crystal plasticity model” Section). First a continuous texture was generated in the form of spherical harmonic C-coefficients (with a certain maximum rank L_max_) from the raw EBSD texture [10]. This was done by assuming a Gaussian distribution (with a certain spread *φ*_0_) on each of the 471,411 indexed pixels in the raw EBSD data. The continuous texture was then re-discretized into 10,000 orientations with the statistical method described in [94].

**Fig. 29:**
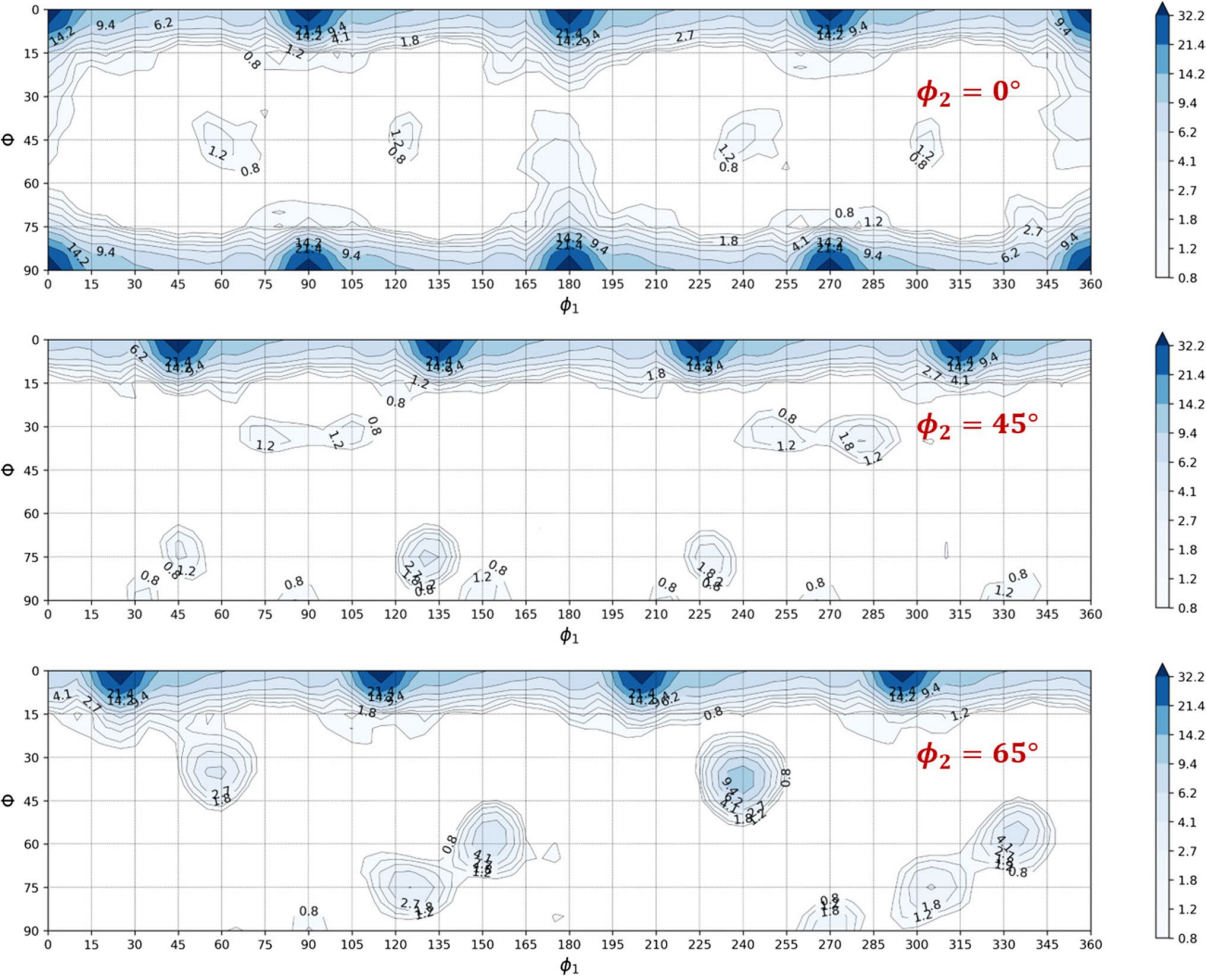
The ODF maps of the discrete texture used as the input for virtual experiments with ALAMEL crystal plasticity model

Figure 29 shows the orientation distribution function (ODF) maps of the discrete texture at three different sections, which compare well with the measured textures with the main components preserved in the discrete texture. For this visualization, the discrete texture was made continuous again with the same *φ*_0_ and *L*_max_ values as those used for converting the initial EBSD orientations into a continuous ODF.

**Fig. 30:**
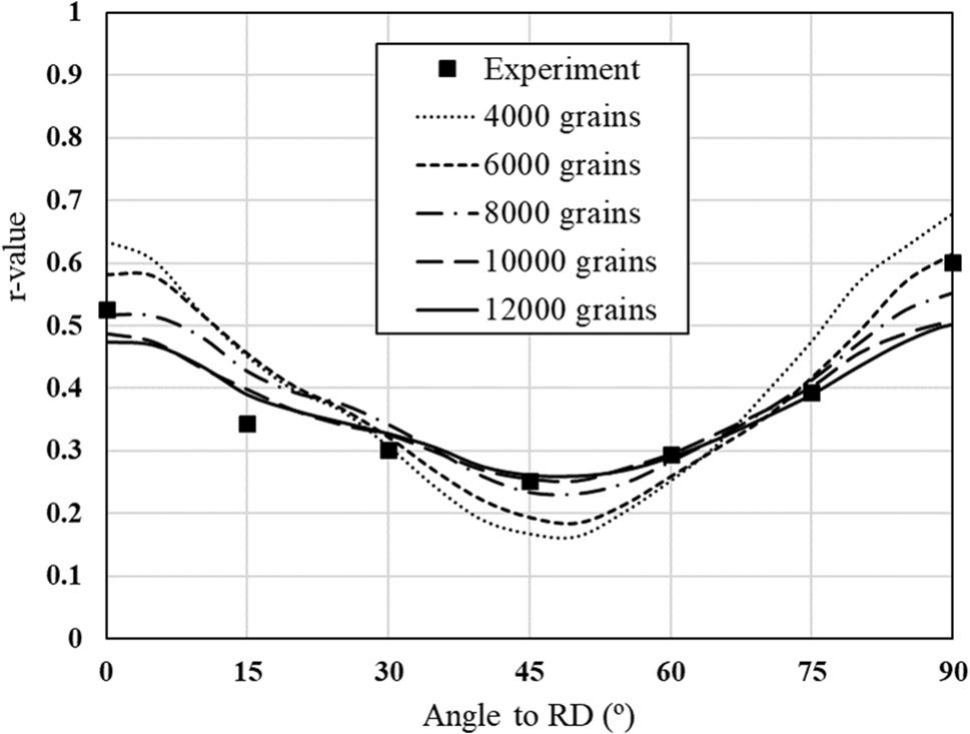
Effect of number of grains in the discrete texture on the *r*-values obtained by ALAMEL. This texture is generated by making the measured EBSD texture continuous with *φ*_0_ *=* 3^°^ and *L*_max_ = 22 followed by a statistical discretization procedure described in [94]

It was observed that the Gaussian spread width, *φ*_0_, and the maximum rank for the generalized spherical harmonics, *L*_max_, are important parameters that affect the results of the virtual experiments. When *φ*_0_ is too large (in practice above 6^°^), the virtual experimentation leads to a larger ∆*r* = *r*_0_ − 2*r*_45_ + *r*_90_ than that in the physical experiments, and the results are almost insensitive to the number of grains, *n*_g_, that are considered for the ALAMEL virtual tests. On the other hand, when *φ*_0_ is 6^°^ or smaller, the ∆*r* value decreased and converged to the experimental value when *n*_g_ was increased (see Fig. 30). For the aluminium alloy in the present study, a combination of *φ*_0_ = 3^°^, *L*_max_ = 22, and *n*_g_ = 10,000 was found to be sufficient for reproducing the *r*-values with the virtual experiments, as will be shown in “Facet-3D model” Section (see Fig. 32). As shown by Hutchinson et al. [49], the Gaussian spread of *φ*_0_ = 3^°^ offers the best possible correspondence between XRD and EBSD derived ODFs.

##### Texture description for Caz2018polycrys

As mentioned in “Material and initial texture” Section, the organizers at UA provided the measured ODF of the AA 6016-T4 aluminium alloy (see Fig. 1 and Table 2). This ODF was first symmetrized to impose orthotropy and the MTEX Matlab Toolbox [3] software was used to generate 250 orientations representative of the material texture, which were further used as input in all the FE simulations with Caz2018polycrys, which relies on Caz2018singlecrys yield criterion for the description of the plastic behaviour of the constituent crystallites.

##### Texture description for Minty

For Minty law, the texture data provided by the benchmark committee was also used (see “Material and initial texture” Section). From EBSD measurements, a set of 1000 crystal orientations in the form of Euler angles and weights was made available by the organizers. Such format can be directly used by Minty. It is worth to note that the orthotropic symmetry of the material (as shown in Fig. 1(b)) is considered in the set of 1000 grains.

##### Texture description for Damask

**Fig. 31:**
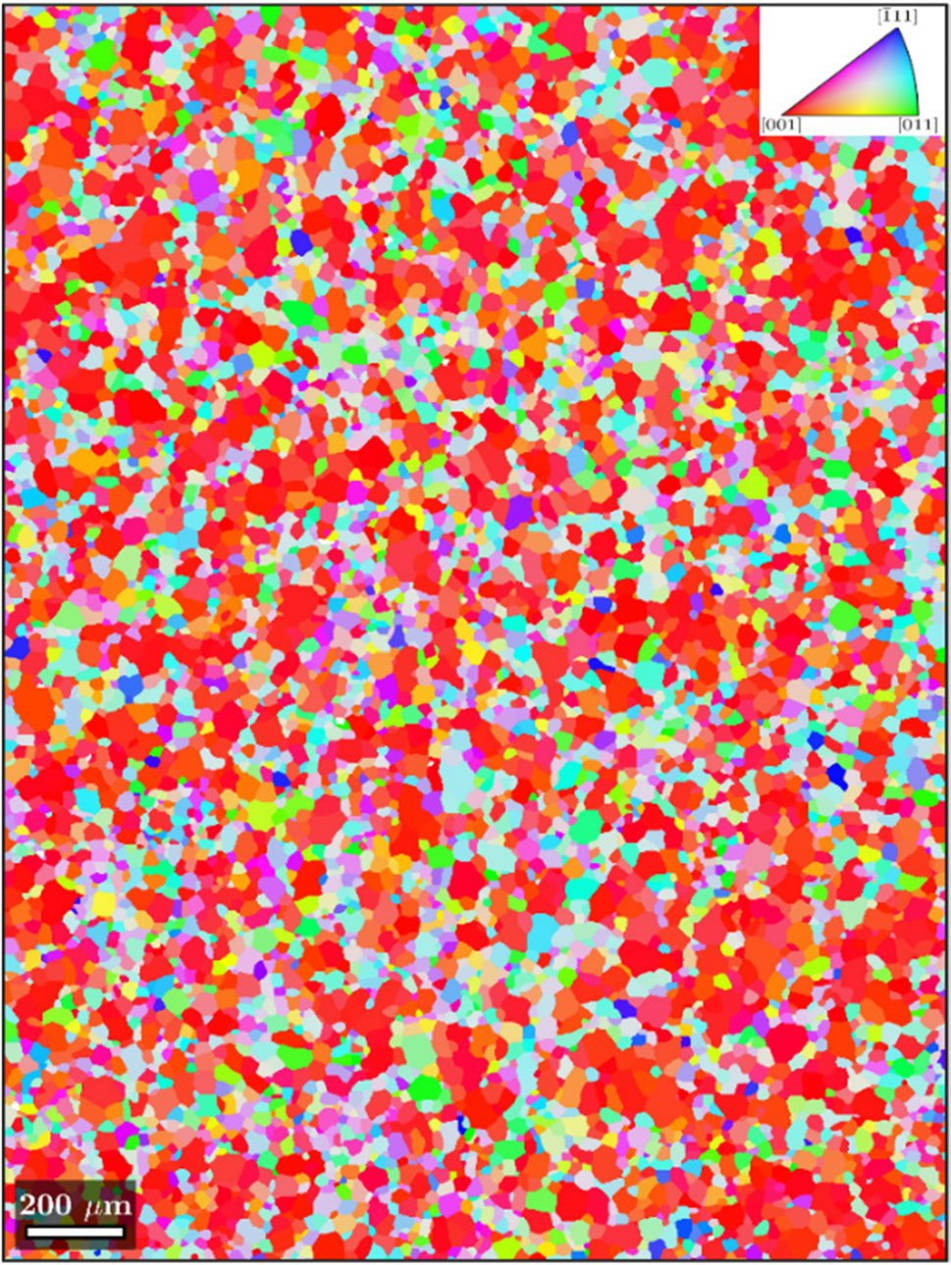
The EBSD map including the 7509 grains selected as input for the 3D RVE of AA 6016-T4 aluminium alloy sheet

The 3D RVE crystal plasticity modelling used to predict the mechanical anisotropy of AA 6016-T4 needs texture data. First, the raw EBSD data in “Material and initial texture” Section were post-processed to remove the non-indexed points by their neighbouring orientations using the open-source MATLAB package, MTEX 5.6.0. As illustrated in Fig. 31, 7509 grains in total were obtained from the post-processed EBSD map, among which the majority of the grains are aligned with cube orientation. Then, the orientations of the 7509 grains were assigned in the 3D RVE by using the open-source polycrystal generation software Neper, based upon the Voronoi tessellation method.

##### Texture description for VPSC model

The microstructural information required by the VPSC model is the grain shape, expressed as the aspect ratios between the axes of the equivalent ellipsoid and a set of Euler angles indicating its spatial orientation (an average shape is commonly used for all grains in a phase), and a discrete crystallographic texture. Moreover, the model is used here in a particular way in order to take into account the influence of grain sizes. The capacity of VPSC to simulate multiphase materials is exploited to consider grains of different sizes as if they were different phases. The adopted approach by UGent team is similar to the one described in [33].

The microstructural data required by the model are extracted from the EBSD experimental data described in “Material and initial texture” Section. First, the raw data are processed with the help of the Dream3D software [39]. The data points with lower confidence index and image quality are removed, and the corresponding areas are filled extrapolating from adjacent grains. Then, using a grain boundary misorientation threshold of 5 degrees, the data is partitioned into different grains, and a grain list is generated with the grain area and the orientation obtained by averaging over all the data points of the grain. The list is then ordered by grain area and divided into three different sections, each corresponding to a different size range. For each of these sections, a different crystallographic texture and average grain shape are calculated. The different sections will be considered as different phases in the model, but, in fact, they correspond to the same material phase. The equivalent ellipsoids of each grain are calculated using Dream3D, and then the average lengths are calculated using the geometric average, while a discrete texture is calculated using the MTM-FHM software [96]. First, an ODF is calculated (with triclinic symmetry, a Gaussian spread of 7 degrees, and *L*_*max*_ = 22), and then a discrete texture of 500 orientations is extracted. Thus, in total, for the three phases in the model, 1500 grains are considered in the simulation of “Cup drawing simulations, analysis and discussion” Section.

##### About the representative number of grains used to model the texture

Let us already point the large discrepancy in the number of grains selected as representative of the texture (see Table 13). Each team made the choice based on their experience and focus on capturing the tensile test behaviour (yield stress variation and Lankford prediction in different direction from RD) and, sometimes, the shear test behaviour (see “Parameter identification, validation and/or use of crystal plasticity models” Section), before modelling the deep drawing process. The material chosen does not present an unusual earing profile (4 ears is a classical pattern). However, its strong cube texture with grains with different sizes already present some challenges. Therefore, a blind choice to model any material with a set of representative crystals, without any comparison with experiments, seems out of the scope. Nowadays, forgetting experiments and relying only on texture measurements and digital tools is still unreliable.

**Table 13:** Number of crystals used to model the texture and target model for each team

Team	Target constitutive law	Number of grains
KUL	ALAMEL model	10,000
REEF	Caz2018polycrys	250
ULiege	Minty law	1000
NTNU	3D RVE for FFT (DAMASK)	7509
UGent	VPSC (own implementation)	1500

#### Parameter identification, validation and/or use of crystal plasticity models

##### Facet-3D model

The Facet-3D yield function was constructed based on 200 yield stresses computed by ALAMEL and the corresponding plastic strain rate directions obtained by virtual experiments on an equi-distant grid in 3D stress space. This grid was generated using the Spiral method [78] by evenly distributing 200 points on a sphere with a radius 1 and centred at the origin in a Cartesian coordinate system. Here, the *x*, *y*, and *z* coordinates of each point were assumed to define the components of a stress mode $$\boldsymbol{u}={\left[\begin{array}{ccc}x& y& z\end{array}\right]}^{\mathrm{T}}$$, and the yield stress *s*^y^, corresponding to this vector, was obtained from virtual experiment. Then, the coefficients of Facet-3D expression as defined in Eq. (24) were found by a non-negative least square (NNLS) fitting [35] to make sure that all of them are positive.

In the present study, the Facet order was 8, and the fitted Facet-3D expression included 38 terms with the coefficients and normals to the facets given in Table 14.

**Table 14:** The coefficients and normal vectors to facets for the fitted Facet-3D with order 8

Facet number *k*	Coefficient *c*_*k*_	Normal vector to the facet (***a***_*k*_)		
		${a}_k^1$	${a}_k^2$	${a}_k^3$
1	0.383	0.115	−0.003	0.403
2	0.056	−0.009	−0.381	0.304
3	0.227	0.267	−0.015	0.341
4	0.291	−0.294	0.001	0.308
5	0.025	0.336	0.015	0.279
6	0.283	0.186	0.393	0.239
7	0.266	−0.176	0.407	0.231
8	0.082	−0.193	−0.410	0.212
9	0.051	0.185	−0.418	0.207
10	0.554	0.245	−0.386	0.187
11	0.464	0.245	0.399	0.169
12	0.223	−0.274	0.383	0.140
13	0.141	−0.432	−0.028	0.086
14	0.578	0.444	0.006	0.043
15	0.147	0.332	0.338	0.042
16	0.264	−0.329	0.347	−0.013
17	0.057	−0.445	0.027	−0.021
18	0.003	−0.298	−0.384	−0.047
19	0.073	0.435	−0.060	−0.064
20	0.275	0.296	0.370	−0.108
21	0.056	−0.231	0.414	−0.157
22	0.001	−0.282	0.373	−0.145
23	0.058	−0.277	−0.366	−0.165
24	0.264	0.220	−0.402	−0.196
25	0.033	0.273	−0.349	−0.190
26	0.291	0.386	−0.028	−0.194
27	0.482	0.228	0.385	−0.208
28	0.303	−0.349	0.029	−0.262
29	0.081	0.139	−0.402	−0.258
30	0.034	0.117	0.397	−0.273
31	0.317	0.029	0.408	−0.283
32	0.287	−0.065	0.401	−0.284
33	0.210	−0.279	−0.013	−0.332
34	0.463	−0.013	−0.385	−0.300
35	0.228	0.233	−0.016	−0.353
36	0.259	0.152	−0.003	−0.390
37	0.102	0.055	−0.035	−0.409
38	0.166	0.016	0.031	−0.412

**Fig. 32:**
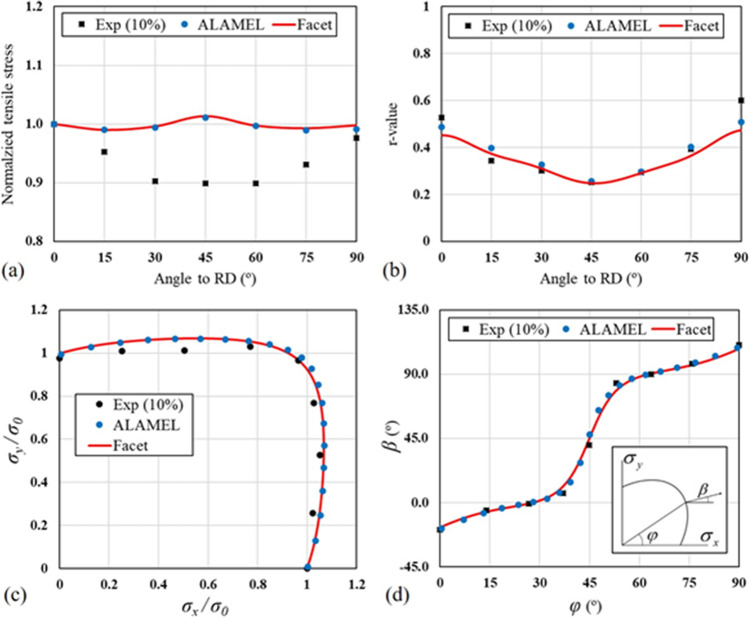
Comparison of **a** normalized tensile stresses, **b** *r*-values, **c** yield loci, and **d** directions of the plastic strain rates from experiment (TUAT values) virtual experiment (ALAMEL), and Facet-3D

Figure 32 compares the experimental *r*-values, normalized tensile stresses, yield loci, and directions of the plastic strain rates with those obtained from the virtual experiments and the fitted Facet-3D.

The maximum difference between the *r*-values predicted by ALAMEL virtual tests and the experimental ones is around 0.078; and between the Facet-3D approximation and the experiments it is around 0.064 (Fig. 32(b)). The experimental normalized tensile yield stress values with a minimum at 45° is not predicted well by ALAMEL and Facet (Fig. 32(a)). It needs to be taken into account that the yield stress anisotropy is not an exclusive function of the crystallographic texture, but may also be affected by spatial anisotropy of other microstructural elements, such as the morphology of grains and/or particles and the anisotropic distribution of particles. The ALAMEL yield surface (and the Facet approximation) is more rounded than the experimental one, with yield stresses that are up to 7% larger than the experimental values (Fig. 32(c)). Whereas the ALAMEL normals to the yield surface pass through the experimental ones, the Facet-3D approximation shows deviations up to 14.5° (Fig. 32(d)). Together with the previously mentioned computational efficiency, the resulting accuracy in reproducing the anisotropic plastic material behaviour makes the combination of ALAMEL and Facet highly suitable for multi-scale analysis of metal forming processes. A hierarchic framework for such a multi-scale analysis including full-field calculations of the evolution of texture and the associated anisotropy is described in [35].

##### Caz2018polycrys

It is to be noted that the polycrystalline model Caz2018polycrys involves seven independent parameters which are associated with the description of the plastic behaviour of the crystals, namely the four independent parameters of the cubic Cazacu single-crystal law [11], called here Caz2018singlecrys, which describes the yielding behaviour and respectively the three parameters associated with isotropic hardening (see “Caz2018polycrys, a polycrystalline model based on Cazacu single crystal law Caz2018singlecrys” Section). Since the determination of these parameters is done based on macroscopic plastic properties, only an inverse method using results of polycrystalline simulations and macroscopic mechanical test data can be used for identification. The multi-step identification procedure used is outlined in the following. Given that this procedure does not require a very large number of FE simulations, the proposed approach enables a rapid/time efficient determination of the model parameters. While the computational approach was presented in “Caz2018polycrys, a polycrystalline model based on Cazacu single crystal law Caz2018singlecrys” Section, here we recall that in the FE simulations, a polycrystalline aggregate is associated with each FE integration point. The FE code imposes the computed macroscopic velocity gradient on the polycrystal. The orientation and the hardening of the individual grains depending on the deformation history of the element are updated, and the homogenization formula (Eq. 33) predicts the macroscopic stress for use in the solution of the continuum equilibrium equations. The advantage of this approach is that it accurately accounts for material anisotropy and its evolution with texture development.

**Fig. 33:**
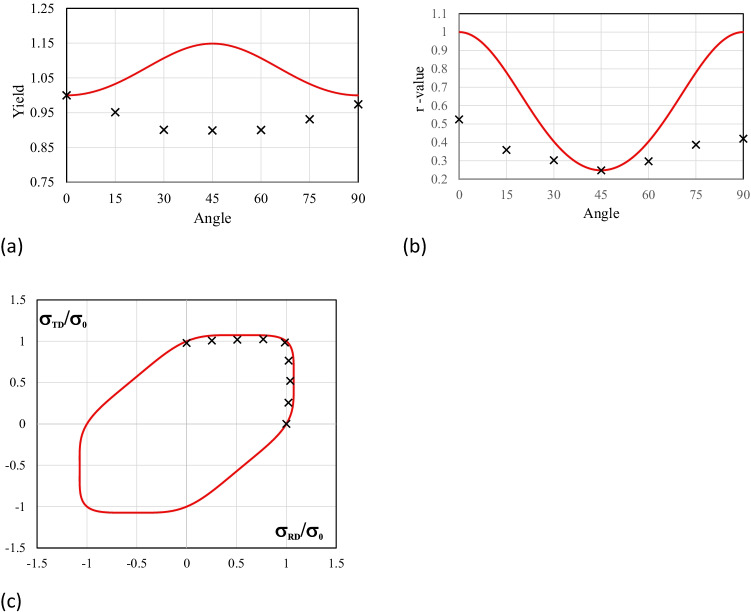
Predicted anisotropy for the AA 6016-T4 alloy assuming ideal cube orientation [100] using Eq. (28–30) (parameters values Table 15-line 1): **a** Uniaxial tensile flow stresses; **b** *r*-values; **c** Predicted yield surface section in the biaxial plane (*σ*_RD_, *σ*_TD_) (no shear). Data from UA (uniaxial data) and TUAT (biaxial data) are represented by cross symbols. All stresses are normalized by the yield stress along the rolling direction, σ_0_

In general, the initial guess values of anisotropy coefficients (Lankford coefficient) are taken equal to unity (i.e. equal to the isotropic values). However, as within 30 degrees misorientation, the benchmark AA 6016-T4 material has almost 52% of the grains along the cube component (see Table 2); the first step of the applied identification procedure consists in considering that only the cube component is present in the texture, i.e. all the grains are oriented along the <100> directions. Each constituent crystal has the same response under the applied loadings, so the polycrystalline response is the same as the response of each single crystal. Only under this assumption that 100% of the grains have the [100] orientation, one can use a least square fit of the parameters of the cubic single crystal law of Eqs. (28–30) to predict mechanical data in uniaxial and biaxial tension, by comparison with the experimental results reported by UA and TUAT (see “Uniaxial tensile tests” and “Biaxial tensile tests” Sections). The identified parameters by REEF of Eqs. (28–30) of this first step are defined in Table 15, line 1. With the assumption of ideal cube orientation [100], the variation obtained for the yield stress and *r*-values with respect to the angle from RD is given in Fig. 33(a)-(b) while Fig. 33(c) shows the projection of the yield surface in the biaxial plane (*σ*_RD_, *σ*_TD_).

**Table 15:** REEF identified parameters for Caz2018singlecrys (Eqs. (28–30) of “Caz2018polycrys, a polycrystalline model based on Cazacu single crystal law Caz2018singlecrys” Section) at different steps of the identification method using the UA tensile test results (see “Uniaxial tensile tests” Section)

Assumption	m_1_	m_2_	n_1_	n_3_	n_4_	*c*
Only cube texture component	0.386	0.256	0.239	0.159	0.129	2.4
Polycrystal model (for minimization see (Eq. 47))	0.355	0.0973	0.341	0.103	0.0571	0.8

It is worth emphasizing that the assumption that all the grains have the [100] orientation implies that *r*_0_ = *r*_90_ = 1. Thus, for the AA 6016-T4 material which has an average *r*_0_ = 0.525 and *r*_90_ = 0.429 (see Table 4, results UA 2020), the assumption of ideal cube texture is only justifiable for determining an initial guess of the parameters. In the second step of the identification procedure, we use 250 grains to represent the texture of the AA 6016-T4 material (see “Texture description for Caz2018polycrys” Section). Specifically, we determine the model parameters by numerical minimization of an error function of the form:

47 $$E=\displaystyle\sum_{k=1}^7{\left[\frac{r_k^{\mathrm{th}}}{r_k^{\mathrm{exp}}}-1\right]}^2$$

In the above equation, *k* represents the number of available experimental *r*-ratios (k = 7; data from UA) while the superscript indicates whether the corresponding value is experimental or theoretical. The theoretical *r*-value at an angle *θ* from the RD of the polycrystalline material (a single aggregate of 250 grains) is calculated as:

48 $${r}_{\theta }=-\frac{D_{xx}^\mathrm{p}{\mathit{\sin}}^2\theta -{D}_{xy}^\mathrm{p}\mathit{\sin}\left(2\theta \right)+{D}_{yy}^\mathrm{p}{\mathit{\cos}}^2\theta }{D_{xx}^\mathrm{p}+{D}_{yy}^\mathrm{p}}$$

with the plastic strain-rate **D**^p^ expressed in the loading frame, given as

49 $${\mathbf{D}}^\mathrm{p}=\frac{\dot{\lambda}}{N}\displaystyle\sum_{i=1}^N\frac{\partial {\overline{\sigma}}_{\text{grain}}^i\left({\mathbf{R}}_i^\mathrm{T}{\boldsymbol{\upsigma} \mathbf{R}}_i\right)}{\partial \left({\mathbf{R}}_i^\mathrm{T}{\boldsymbol{\upsigma} \mathbf{R}}_i\right)}{\mathbf{R}}_i^\mathrm{T}$$

where $\dot{\lambda}$ is the plastic multiplier, **σ** denotes the applied stress tensor, *N* is the number of grains considered in the input texture, ${\overline{\sigma}}_{\text{grain}}^i$ is the effective stress of grain *i* according to Eq. (30) and **R**_*i*_ is the transformation matrix for passage from the crystal axes of grain *i* to the loading frame axes.

Note that in this second step of the identification procedure in the determination of the set of parameters, we took into consideration the main sources of the anisotropic response of the AA 6016-T4 material under uniaxial tension (measured Lankford coefficient and initial texture).

**Fig. 34:**
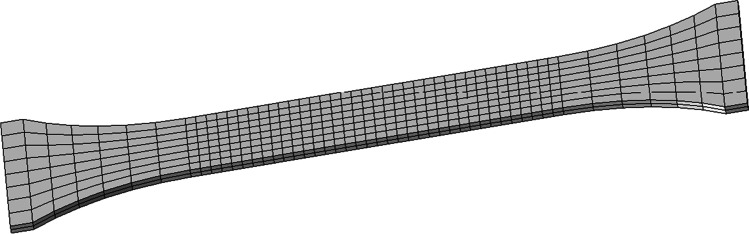
FE mesh used in the simulations of the uniaxial tension tests performed with the FE polycrystalline model

Finally, the last step of the identification procedure consists in fine-tuning the parameters determined in the previous step, to further improve the predictions of the anisotropy in *r*-values for this material. For this purpose, only a few FE simulations using the polycrystalline model described by Eqs. (32)–(33) in “Caz2018polycrys, a polycrystalline model based on Cazacu single crystal law Caz2018singlecrys” Section were performed on uniaxial tension tests at *θ*=0°, 15°, 30°, 45°, 60°, 75° and 90°. In the FE simulations of any uniaxial tension test, the specimen is meshed with 570 ABAQUS C3D8R reduced integration elements (see Fig. 34 for the FE mesh). As previously mentioned, a polycrystalline aggregate is associated with each FE integration point. The numerical *r*-value is extracted from the slope of the curve *ε*_*w*_ vs. *ε*_*t*_, where *ε*_*w*_ =  *ln* (*w*/*w*_0_) and *ε*_*t*_ =  *ln* (*t*/*t*_0_) with *w*_*0*_ and *w* being respectively the initial and current width, and *t*_*0*_ and *t* being the initial and current thickness.

**Fig. 35:**
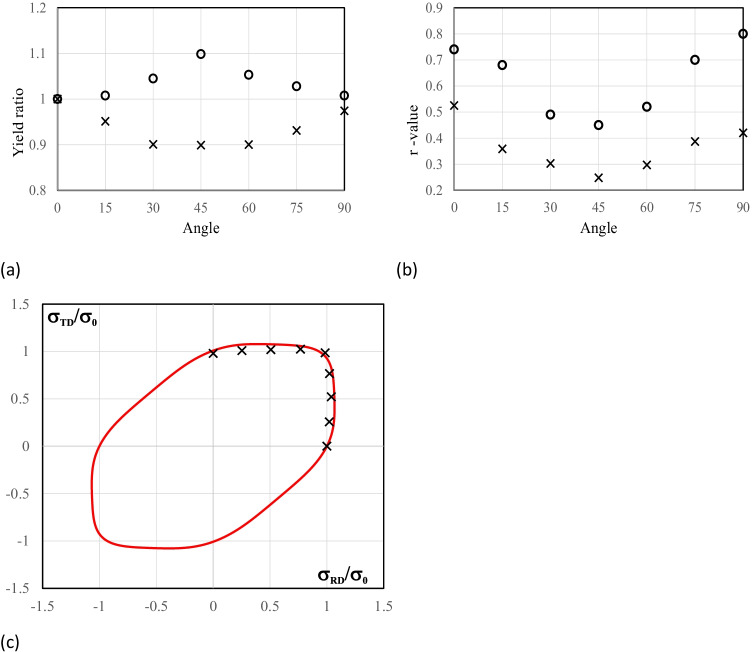
Predicted anisotropy using the polycrystalline FE model taking into account the texture of the AA 6016-T4 alloy (parameters values Table 15-line 2): **a** Uniaxial tensile flow stresses; **b** r-values; **c** Predicted yield surface section in the biaxial plane (***σ***_RD_, ***σ***_TD_) (no shear). Data (UA) are represented by cross symbols. All stresses are normalized by the yield stress along the rolling direction, σ_0_

The final values of the identified parameters for the AA 6016-T4 polycrystalline material are provided in Table 15, line 2, and were further used to conduct the FE simulations with Caz2018polycrys model of cup drawing presented in “Cup drawing simulations, analysis and discussion” Section. Figure 35(a) and (b) show the comparisons of the FE polycrystalline simulation results (circles) with the experimental data for the uniaxial yield stresses and *r*-values (cross) for the seven orientations in the plane of the sheet. The FE predicted yield surface cross-section in the RD, TD plane is shown in Fig. 35(c).

Finally, for Caz2018polycrys an isotropic Swift hardening law (see Eq. (3)) was considered at crystal plasticity level, the respective parameters being determined from the experimental stress-strain curve in uniaxial tension along *θ*=0° reported by UA (see Table 16).

**Table 16:** Parameters of the hardening law at crystal plasticity level in the polycrystalline model Caz2018polycrys

Hardening model	Set of parameters (REEF identification)		
	*K*_*0*_ (MPa)	*ε*_0_ (−)	*n (−)*
Swift	277	0.0089	0.285

**Fig. 36:**
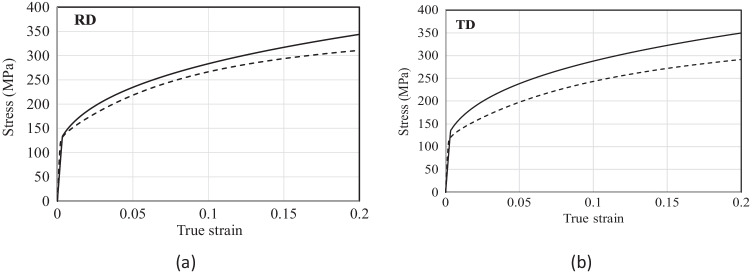
Comparison between experimental stress-strain curves (dashed line) in uniaxial tension and FE predictions obtained with the polycrystalline model (Eqs. (32)–(33), data set Table 15-line 2 and Table 16): **a** RD direction; **b** TD direction

**Fig. 37:**
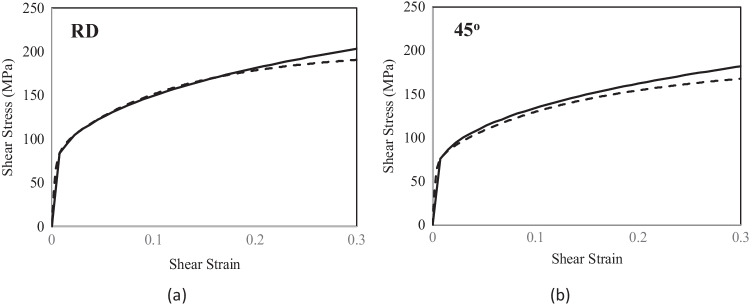
Comparison between experimental stress-strain curves (dashed line) in shear and FE predictions obtained with the polycrystalline model (Eqs. (32)–(33), data set Table 15-line 2 and Table 16): **a** RD direction; **b** at 45° to the RD direction

We conclude the verification of the validity of the parametrization of the Caz2018polycrys model with a comparison between experimental stress-strain curves and polycrystalline predictions for uniaxial tension at RD and TD in Fig. 36. Furthermore, illustrative examples of the predictive capabilities of the new FE polycrystalline model is done through comparison with shear tests at RD and 45° to RD (data obtained at UA) in Fig. 37. It is worth noting that shear tests data were not used in the identification procedure and as such, the results serve for validation of the polycrystalline model.

Note that the impact of the hardening functions and parameters at the level of crystal plasticity models cannot be neglected as shown by [50], where 3 single slip hardening laws were tested for an AA 5754 aluminium sheet. A unit cell model representing 221 grains/orientations and meshed by 2484 finite elements predicted small differences within the virtual tensile stress-strain curves, but a clear effect on the predictions of *r*-values.

##### Minty law

In Minty law, a type of interpolation law, the anisotropy of the material behaviour is solely determined from its texture description, as it uses a Taylor assumption where a set of crystals at one integration point has a single macroscopic strain value. For the present study, the texture was represented by the 1000 crystal orientations extracted from EBSD measurements as explained in “Texture description for Minty” Section.

The isotropic elastic behaviour is characterized by Young’s modulus (E = 70,000 MPa) and Poisson’s ratio 0.33. As mentioned in “Minty model, an interpolation approach” Section, the isotropic hardening is modelled by a Swift type equation. However, the classical formulation of Swift hardening expressed as the yield stress as a function of the equivalent plastic strain (Eq. 3) must be converted in the critical resolved shear stress *τ* vs. the accumulated polycrystalline induced slip Γ (Eq. 35). As detailed in Eq. (50), adapted from [29], this conversion requires the computation of Taylor’s factor $\overline{M}$ whose value is 2.8054 for the studied material.

50 $$\left\{\begin{array}{c}K=\frac{K_0}{{\overline{M}}^{n+1}}\\ {}{\varGamma}^0={\varepsilon}_0\cdot \overline{M}\end{array}\right.$$

In this equation *n* is unchanged compared with Eq. (35). Finally, the parameters involved in the isotropic Swift hardening law (see Eq. 3) were determined by the benchmark committee, based on the experimental stress-strain curve in uniaxial tension along RD, and Eq. (50) allows to identify the hardening at the crystal level (see Table 17).

**Table 17:** Set of parameters for hardening Minty law

Hardening model	Set of parameters		
	*K*_0_ *(*MPa*)*	*ε*_0_ (−)	*n* (−)
Macroscopic Swift law Eq. (3) from UA	498.8	0.0089	0.285
	*K (*MPa*)*	*Г*^0^ (−)	*n* (−)
At the crystal plasticity level Eq. (35)	132.51	0.025	0.285

An important numerical parameter in the stress-strain interpolation of Minty law is the size of the interpolation domain, expressed as the angular gap between adjacent stress nodes in 5-D space (see “Minty model, an interpolation approach” Section). A small domain permits an accurate interpolation of the stress; however, it requires frequent updates of the domain leading to a larger computation time. Conversely, a large domain will remain valid longer, but is expected to provide less accuracy. As shown in [41], an angular size of 5° is a good compromise between accuracy and computation time; it is the value used in this study.

**Fig. 38:**
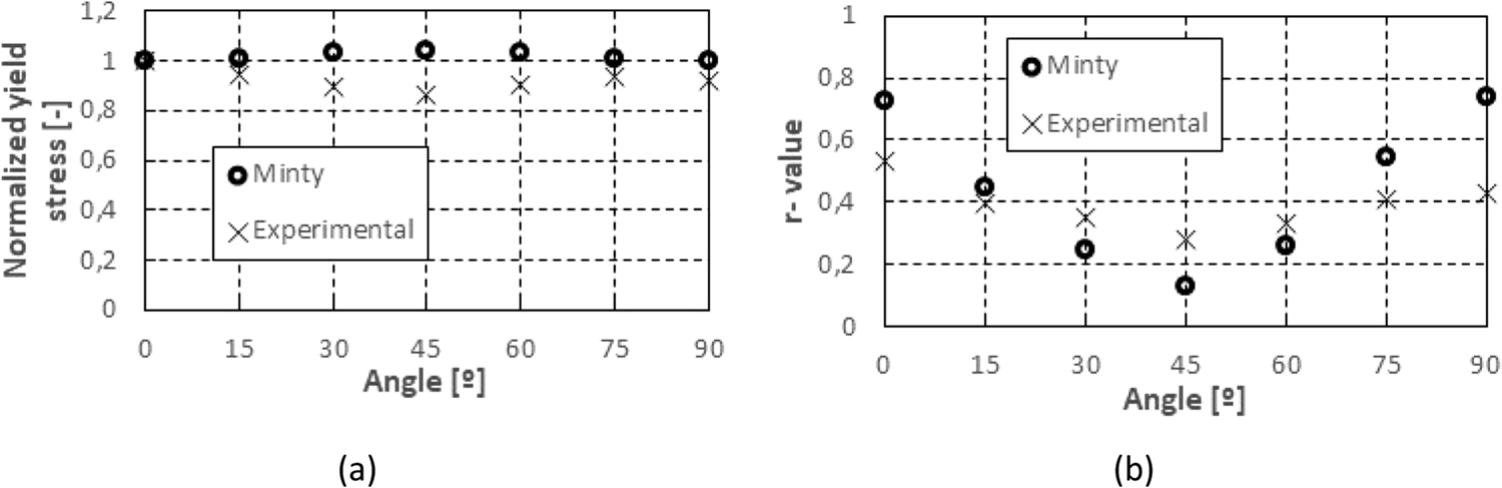
Comparison of normalized yield stress **a** and Lankford coefficients **b** predicted by Minty with experimental values obtained from tensile tests of UA

Figure 38 presents the mechanical behaviour of AA 6016-T4 aluminium alloy as predicted by Minty law. As already observed in Fig. 30, the Lankford coefficient curve (Fig. 38(b)) has a similar shape as the experimental results, but with an overestimated amplitude. Conversely, the yield strength curve (Fig. 38(a)) has an opposite trend to the experimental results with a low amplitude.

##### RVE Damask simulations

**Fig. 39:**
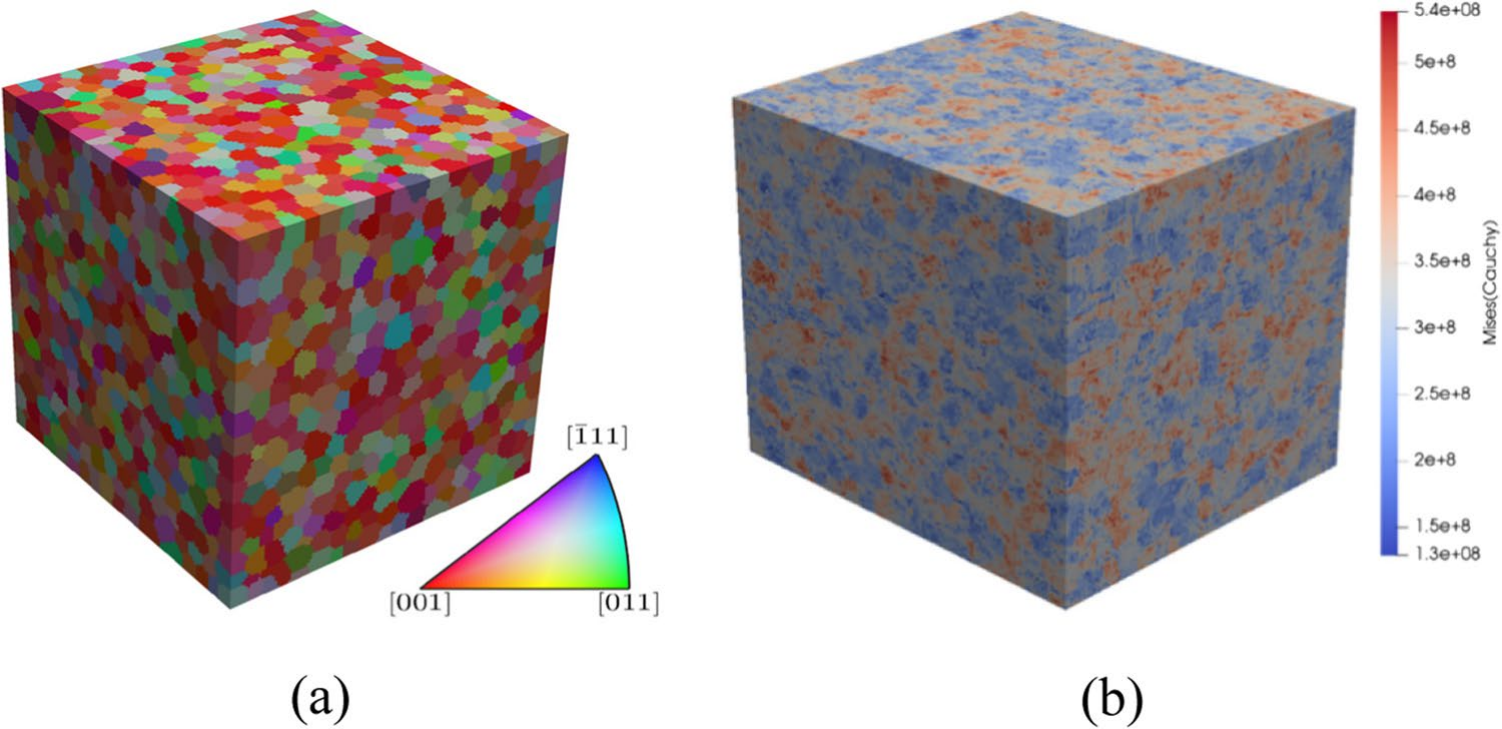
**a** 3D Representative Volume Element (RVE) with 7509 equiaxial grains generated by a Voronoi tessellation; **b** Distribution of equivalent Mises stress (Pa) of RVE in uniaxial tension simulation at RD direction for a macroscopic strain of 0.1

**Fig. 40:**
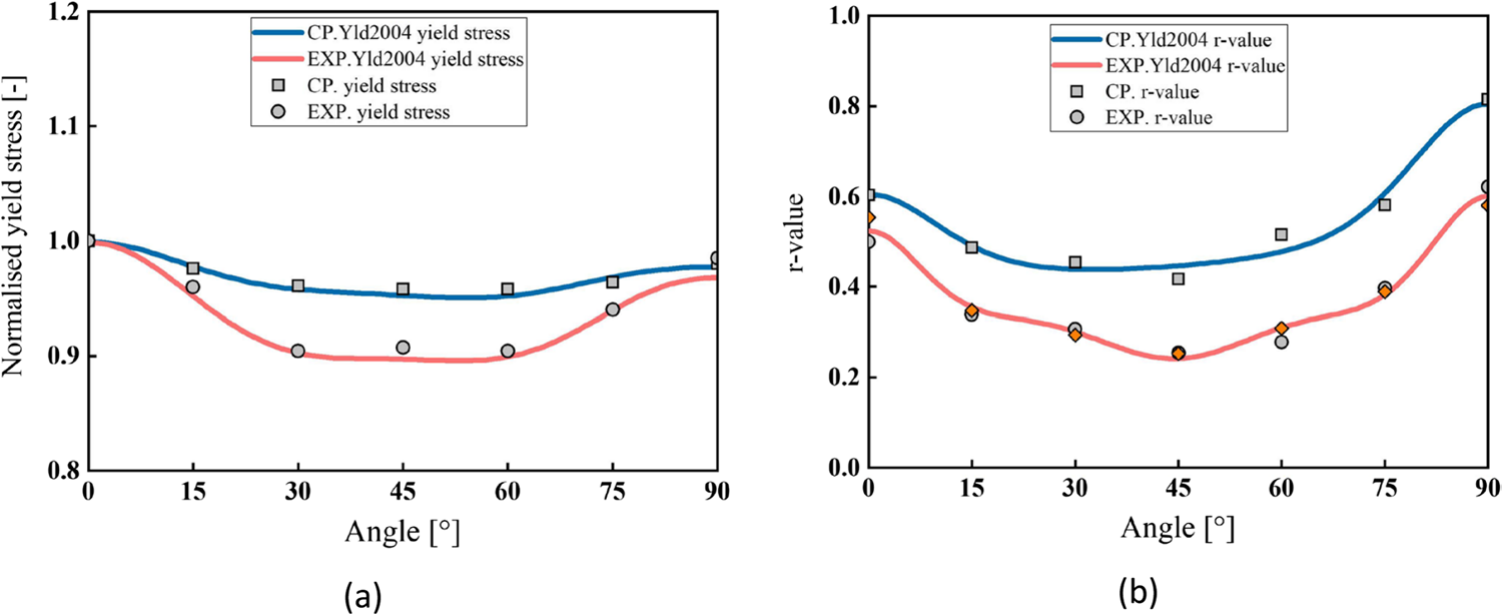
Comparison of mechanical anisotropy of AA 6016-T4 aluminium alloy sheet obtained from experiments, Damask simulations (CP) and Yld2004–18 yield function identified either on physical experiments or from CP results: **a** The directional normalized yield stresses; **b** r-values

Figure 39 shows the established periodic cubic RVE that consists of 7509 equiaxial grains, with a high grid resolution of 120 × 120 × 120 Fourier points. This RVE was used to perform the virtual experiments [64], in the full-field crystal plasticity modelling kit DAMASK—an open-source spectral method by solver [76], as described in “Crystal plasticity model used in DAMASK solver” Section. Twelve slip systems in FCC crystal structure including <01$\overline{1}$>{111} slip system and its all symmetrically equivalent ones were taken into account in the polycrystal modelling. The phenomenological power law was used to describe the hardening behaviour. The hardening parameters *h*_*0*_*, τ*_*0*_*, τ*_*s*_*,* and *a* for the slip system described in “Crystal plasticity model used in DAMASK solver” Section were determined by inverse modelling, focused on sets of stress-strain curves obtained from physical uniaxial tensile, pure shear, and plane-strain tensile tests in RD. The value of strain rate sensitivity, m = 0.02 resulted in good agreement between the experimental (of TUAT 2018) and predicted uniaxial stress-strain curve and it was adopted in this work. The slip system references shear rate ${\dot{\gamma}}^0$, latent hardening parameter *q* and the cubic crystal elastic constants *C*_*11*_*, C*_*12*_*,* and *C*_*44*_ are typical values for cold-rolled FCC aluminium alloy [64]. The parameters of the crystal plasticity model are summarized in Table 18. By Damask simulations, the mechanical responding of the AA 6016-T4 RVE during the virtual uniaxial tensile tests at RD direction can be obtained and is illustrated in Fig. 40.

**Table 18:** Damask crystal plasticity model parameters for the AA 6016-T4 aluminium sheet

*C*_11_	*C*_12_	*C*_44_	${\dot{\gamma}}_0$	*h*_0_	*τ*_0_	*τ*_*s*_	*m*	*a*	*q*
82 GPa	62 GPa	29 GPa	0.001*s*^−1^	1140 MPa	48 MPa	140 MPa	0.02	2.0	1.4

Using the above RVE and Damask simulations, virtual experiments were performed for tensile and biaxial loading conditions. Figure 40(a) and (b), respectively, show the yield stresses and *r*-values of virtual and physical uniaxial tensile tests at 0, 15, 30, 45, 60, 75, and 90 degree with respect to RD at the nominal strain of 10%. Both virtual and physical experiments indicate that the *r*-value is relatively higher at 0 and 90 degree but the lowest at 45 degree. In addition, the lowest normalized yield stress is also observed at 45 degree which is about 0.90 and 0.95 obtained from physical and virtual experiments, respectively.

**Fig. 41:**
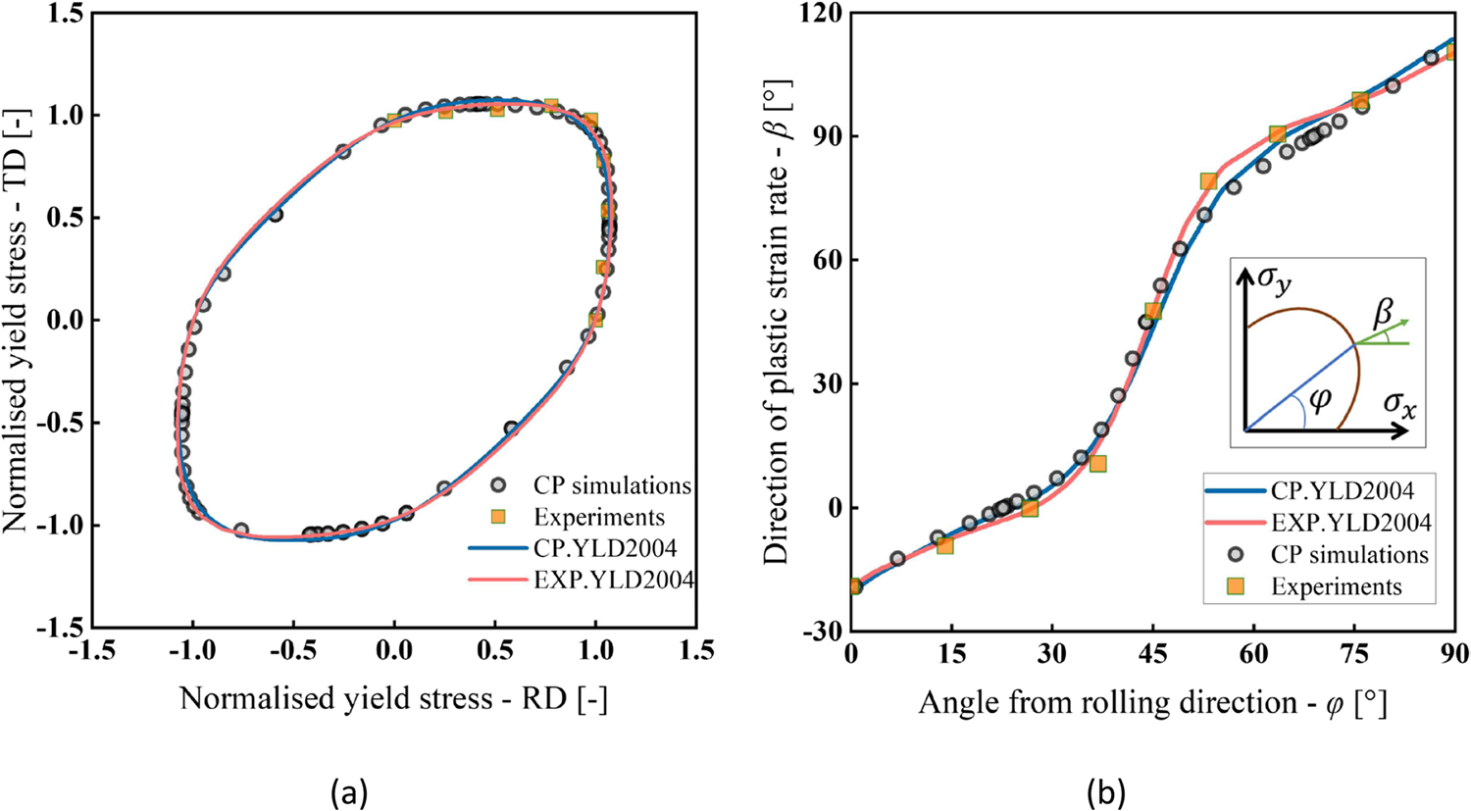
Measured experimental points, CP modelling results and Yld2004–18p yield function cut as well as plastic strain directions: **a** Yield surface of biaxial stress state in RD-TD plane; **b** Direction of plastic strain rate in biaxial tension

Figures 41(a) and (b) show biaxial yield surfaces and directions of plastic strain rate, respectively, obtained from CP modelling and experiments. The plots show a good agreement between the CP predictions and experiment results.

Note that both experimental and CP predicted uniaxial and biaxial material sampling points were used to calibrate the material parameters of Yld2004–18p yield function. The Matlab own least-squares function so-called ‘lsqnonlin’ was used to identify the parameters of Yld2001-18p. The identified parameters values are given in “Yld2000-2D and Yld 2004-18p” Section with the other phenomenological identifications of this yield locus to allow easy comparison of anisotropic parameter values (see Table 28).

##### VPSC model

**Fig. 42:**
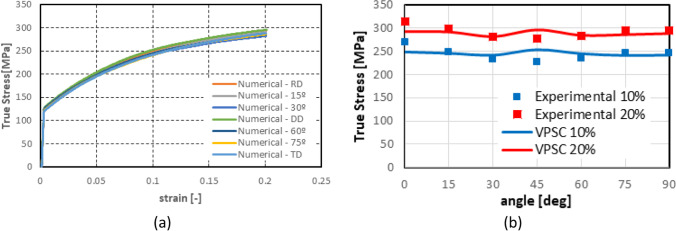
**a** Final predictions of the fitting procedure of the VPSC model parameters; **b** Comparison of the model predictions (continuous line) with experimental values (squares) at specific strain levels

The simulations of the cup drawing test are performed considering three distinct phases within the set of crystals, as explained in “Texture description for VPSC model” Section. The model phases correspond to the same material phase and are defined based on grain size. For each model phase associated to a different grain size, different crystallographic textures and grain shapes are considered. The same strain rate sensitivity exponent and hardening parameters are employed for the three phases, but the *τ*_0_ parameter is modified according to the Hall-Petch relationship (i.e. an additional term is added, proportional to the inverse of the grain diameter square root and a constant *k*_*HP*_). In total, six parameters are fitted: the strain rate sensitivity exponent *n*_*s*_, the Voce law parameters *τ*_0_ (for the average grain size), *τ*_1_, *θ*_0_ and *θ*_1_, (see Eq. 43) and the Hall-Petch constant *k*_*HP*_. The Levenberg-Marquadt algorithm is used for the fitting [62]. Having an initial estimation, a gradient value is calculated from simulations in which a small perturbation is applied to each parameter, and this gradient is then used to find an improved solution. The goal is to reproduce the uniform strain region of the tensile experiments in different directions described in “Uniaxial tensile tests” Section (TUAT tests in 0°, 15°, 30°, 45°, 60°, 70° and 90° directions). After several iterations, the method converges to the parameters shown in Table 19. Figure 42 compares the experimental and simulation results.

**Table 19:** VPSC fitted parameters

*n*_*s*_ (−)	*τ*_0_ (MPa)	*τ*_1_(MPa)	*θ*_0_ (−)	*θ*_1_ (−)	${k}_{HP}\ \left(\mathrm{MPa}\sqrt{\mathrm{m}}\right)$
44	54.2	33.2	171.0	0.12	14.0

**Fig. 43:**
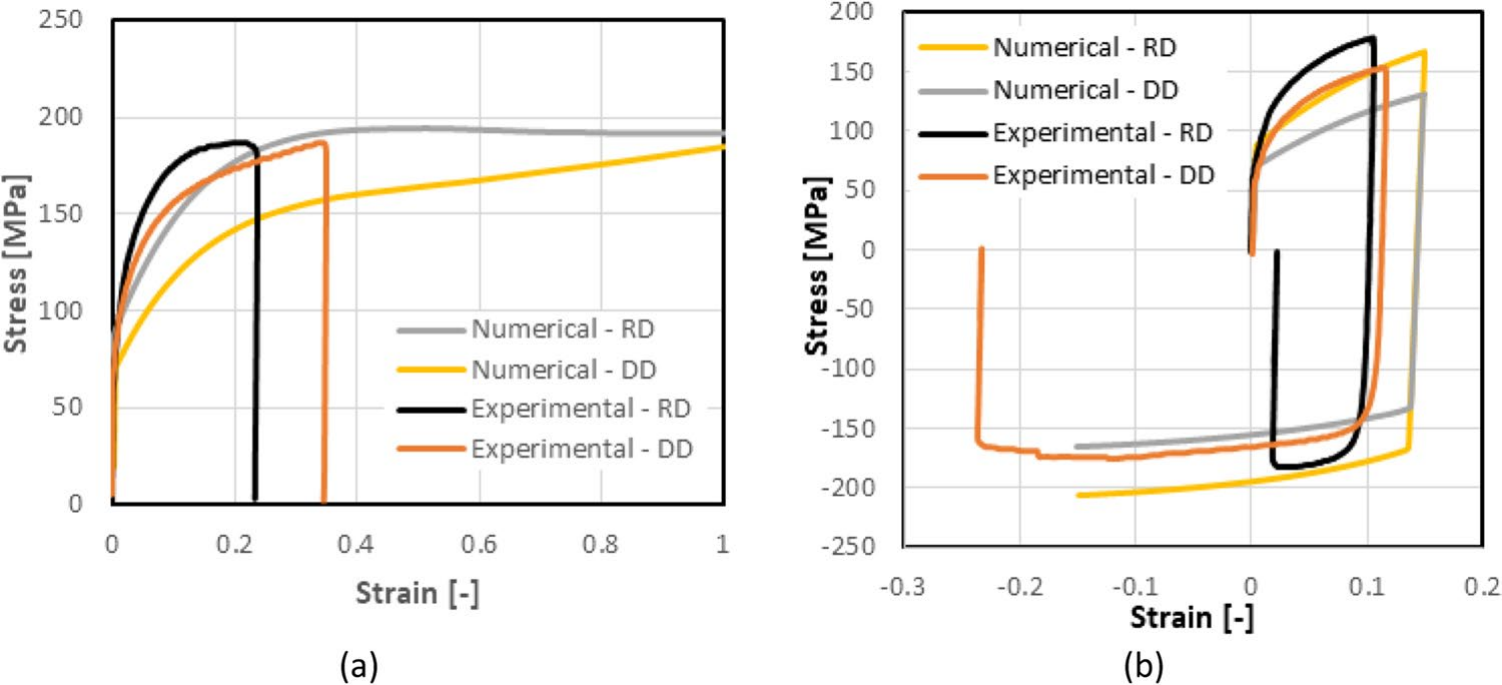
VPSC simulations compared with the: **a** monotonic and **b** reversed shear tests of ULiege, used for the validation of the model

Once the parameters are fitted, the VPSC model and its data set are validated using the monotonic and reverse shear tests of “Monotonic and reverse simple shear tests” Section not exploited in the identification method, as shown in Fig. 43.

**Fig. 44:**
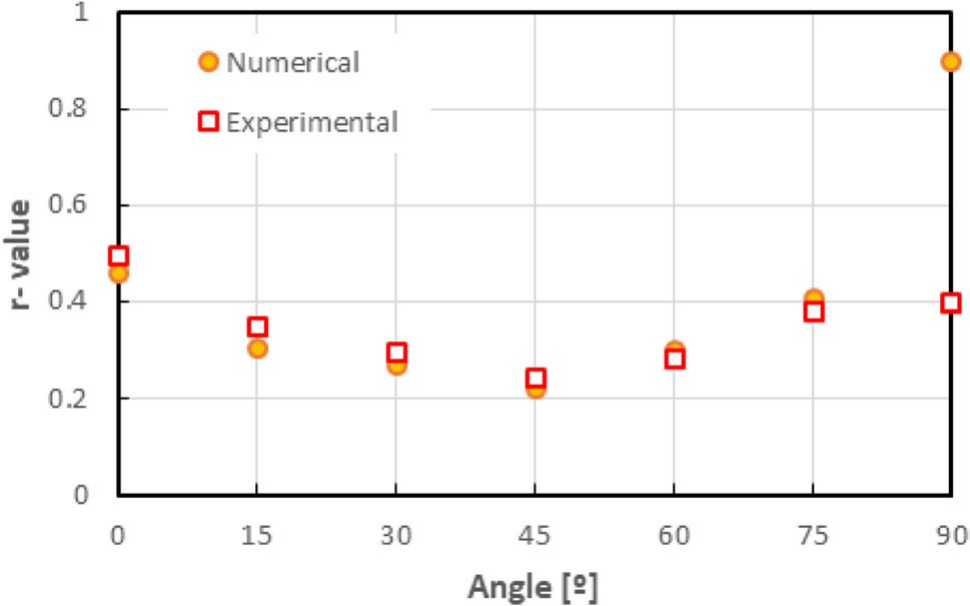
Comparison of experimental (UA) and simulated (VPSC) *r*-values

Note that virtual uniaxial and biaxial tensile experiments performed by means of VPSC are used to calibrate the phenomenological yield criteria Yld2000-2D. From tensile tests in the 0°, 45° and 90° directions, the respective yield strengths *σ*_0_, *σ*_45_, *σ*_90_ and Lankford coefficients *r*_0_, *r*_45_, *r*_90_ are calculated. From an equibiaxial tension test, the yield strength *σ*_*b*_ and Lankford coefficient *r*_*b*_ are calculated. All values are computed at a fixed level of effective plastic strain of 10%. Figure 44 presents the comparison of experimental (UA) and simulated (VPSC) *r*-values.

**Fig. 45:**
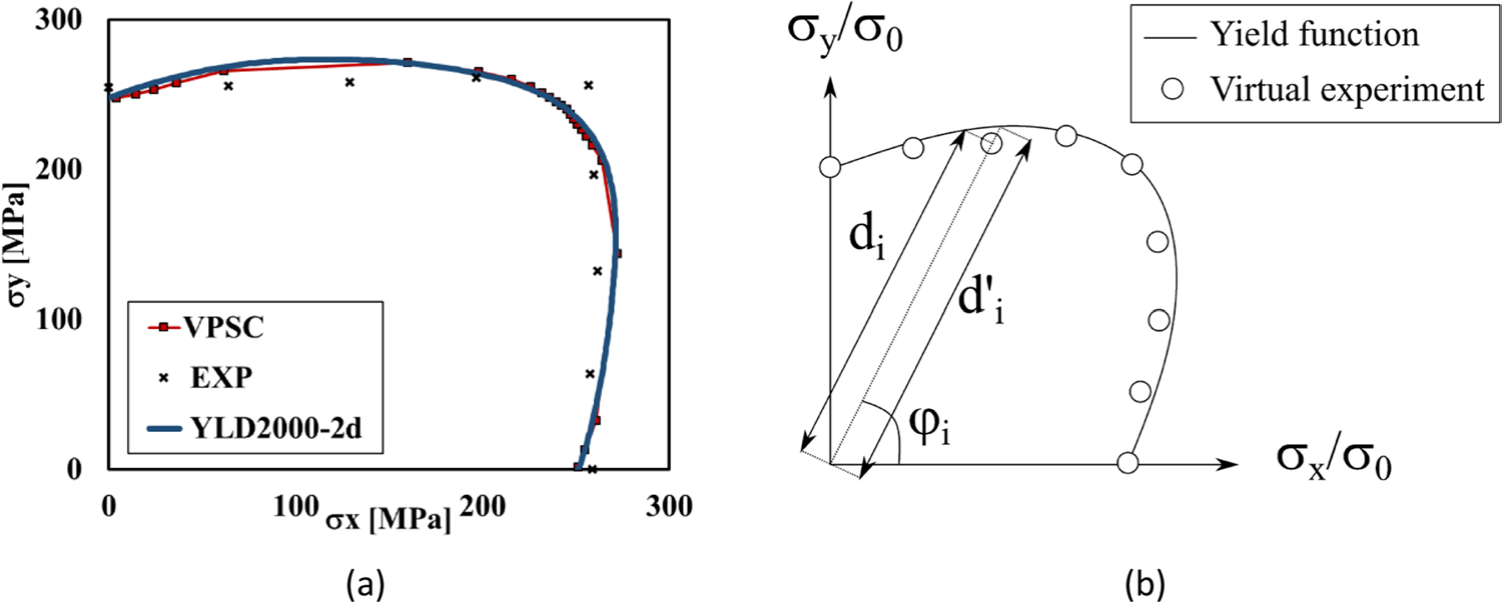
Identification method and validation of VPSC and Yld2000-2D predictions vs. experiments: **a** Experimental, virtual and predicted work contours at 10% of effective plastic strain and **b** Scheme of calibration procedure for *m*

Using the VPSC yield strengths and Lankford coefficients, the Yld2000-2D model parameters *α*_*i*_ (i = 1–8) are identified following the procedure described in [23, 59]. The identification of the anisotropic parameters is performed giving more weight to the *r*-values rather than to the stress amplitudes. This choice is made to enhance the capability of the model to predict the experimental earing profile. The identification of the exponent *m* is performed by a virtual work contour, the value 6.11 will be used. Data points in the *σ*_*x*_ − *σ*_*y*_ principal plane are obtained carrying out virtual biaxial experiments with different, linear stress paths. The VPSC data points used are presented in Fig. 45(a). The values are calculated at the moment when the same level of plastic work per unit volume is applied by *σ*_*x*_ and *σ*_*y*_ as in the tensile experiment along the RD at 10% of effective plastic strain. *m* is found minimizing the root mean square error *δ*_*d*_ between the virtual work contour and the numerical one predicted by Yld2000-2D [23, 59]:

51 $${\delta}_d=\sqrt{\frac{\sum_i^N{\left({d}_i^{\prime}\left({\varphi}_i\right)-{d}_i\left({\varphi}_i\right)\right)}^2}{N}}$$

where *d*_*i*_ and ${d}_i^{\prime }$ are the distances between the origin of the principal stress space and the i-th VPSC data point and Yld2000-2D yield locus, respectively. The slope of the linear stress path is identified by the angle *φ*_*i*_. N (=9) is the total number of data points considered for the minimization procedure. The following stress paths are considered *σ*_*x*_ : *σ*_*y*_ = 1 : 0, 4 : 1, 2 : 1, 4 : 3, 1 : 1, 3 : 4, 1 : 2, 1 : 4, 0 : 1. Figure 45(b) gives a schematic representation of the methodology used for the calculation of *δ*_*d*_.

The model constants obtained from the identification procedure of Yld2000-2D are summarized in Table 27 of “Yld2000-2D and Yld 2004-18p” Section to allow an easy comparison of the yield loci identified by classical tests or virtual ones. Finally, the Voce law parameters obtained by fitting the hardening curve for the tensile experiment performed along RD by UA are provided in “Identification of hardening laws and data used” Section (see Table 32).

##### Caz2018singlecrys

**Fig. 46:**
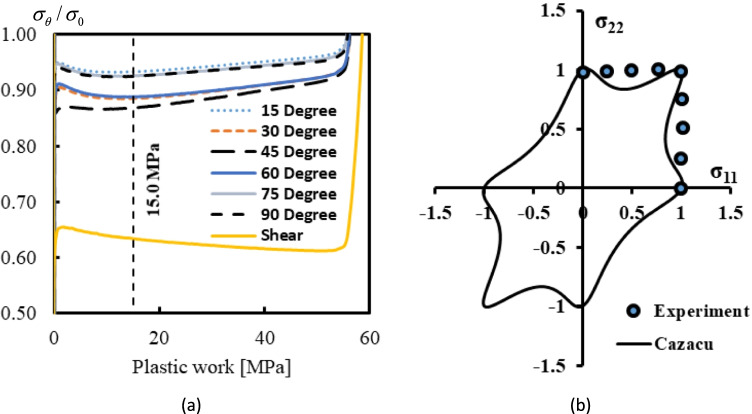
**a** Plastic work per unit volume vs. normalized flow stress in tests along different directions and in shear test in RD direction (UA tests) and **b** predicted Cazacu Single Crystal shape with the data set of Table 20 and **b** experimental yield locus (UA and TUAT)

**Fig. 47:**
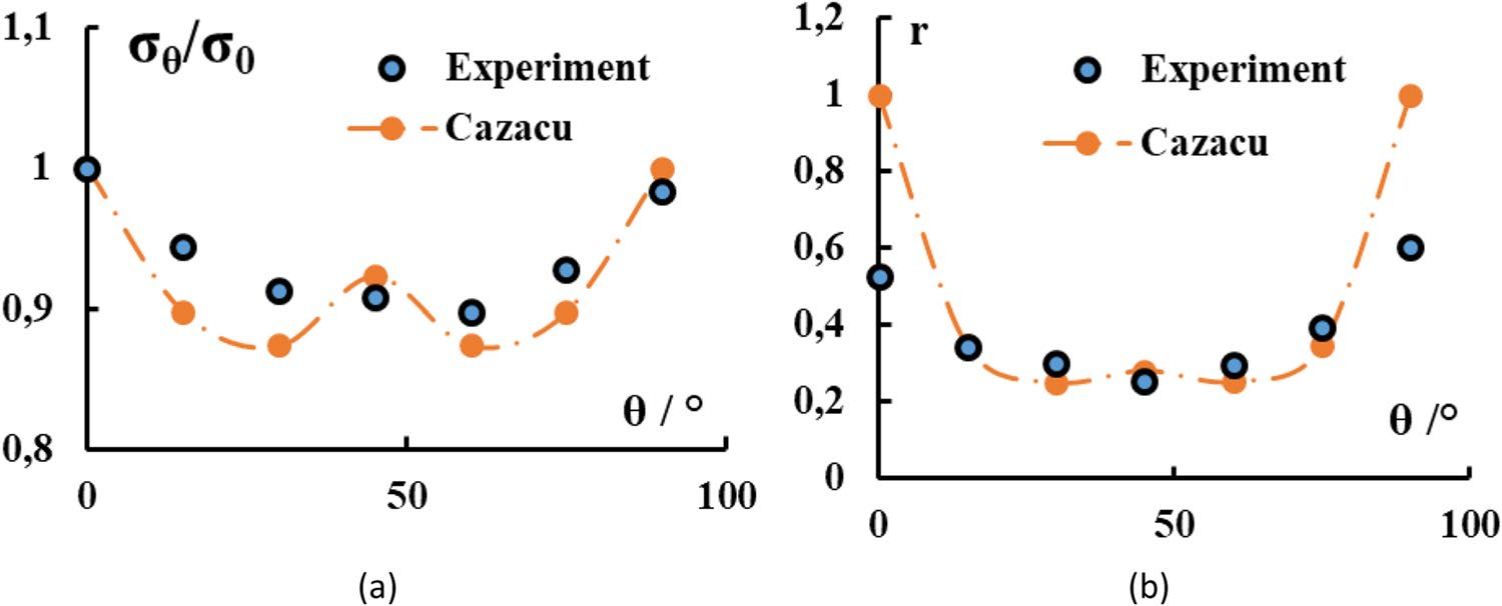
Comparison of **a** normalized flow stress and **b** *r*-values measured by UA tensile tests or predicted by Cazacu single crystal yield criterion identified by POSTECH

If the texture is assumed to be a pure cube texture (simplified assumption based on Table 2), the Cazacu single crystal yield criterion could directly be applied. It was an interesting test performed by POSTECH team. The yield locus coefficients were determined with an optimization algorithm based on the Nelder-Mead Simplex algorithm, using the normalized flow stresses for different stress states obtained from UA test data (tensile and shear). An additional constraint about mainly plane stress assumption, explains that *n*_*4*_ parameter can be seen as irrelevant. The normalized flow stresses are taken at 15 MPa plastic work per unit volume corresponding to 0.07 plastic strain in the RD tensile test (Fig. 46(a)). Table 20 gives the identified coefficients for Cazacu single crystal yield criterion. The predicted yield locus (Fig. 46(b)), normalized flow stress (Fig. 47(a)) and *r*-values (Fig. 47(b)) show considerable deviating from the experimental values, illustrating that the assumptions are too strong: the polycrystalline behaviour should take into account other components than the cube one and the identification method should include out of plane experiments allowing a correct *n*_*4*_ calibration. The effect on deep drawing simulations is presented in “Cup drawing simulations, analysis and discussion” Section.

**Table 20:** POSTECH identified set of parameters of single crystal yield locus Caz2018singlecrys (see Eqs. (28–30) of “Caz2018polycrys, a polycrystalline model based on Cazacu single crystal law Caz2018singlecrys” Section

Assumption	*m*_*1*_	*m*_*2*_	*n*_*1*_	*n*_*3*_	*n*_*4*_	*c*
Pure cube texture component	1	0.489	1.5	0.1	irrelevant	2.568

Note that the convexity issue of the yield locus for this set of parameter values is not a surprise. It happens for this function if *c* and *n*_*1*_ are high, and *n*_*3*_ is low. The same issue can be observed in Drucker’s isotropic yield condition where a high coefficient *c* is assumed for the factor of the third stress invariant J_3_ square. If an implicit FE solver is used for the FE simulations of “Cup drawing simulations, analysis and discussion” Section, the identified yield locus of Fig. 46(b) will be an issue, which explains why POSTECH has chosen an FE explicit solver.

### Parameter identification and validation of phenomenological models

#### Hill48 yield locus (associated law)

The determination of the anisotropic parameters associated to Hill48 model can be done analytically using as input either yield stresses corresponding to uniaxial loading along 0°, 45°, 90° orientations and equibiaxial yield stress or Lankford coefficients for 0°, 45°, 90° directions in the plane of the sheet. If the equivalent stress according to Hill48 yield criterion is given as in Eq. (12) of “3-D orthotropic yield functions (Hill48, CB2001, Yld2004-18p, CPB06ex2, Caz2018-Orth)” Section, the theoretical Hill stress is imposed to coincide with the yield stress along RD. If the assumption of associated flow rule is considered, the respective analytical formulae are given in Table 21 (see for example [16, 56]).

**Table 21:** Analytical determination of the anisotropy parameters in Hill48 model with effective stress given by Eq. (12)

Method	Input parameters	Equations	
Stress based	*σ*_0_, *σ*_45_, *σ*_90_, *σ*_b_	$2F=\frac{\sigma_0^2}{\sigma_{90}^2}-1+\frac{\sigma_0^2}{\sigma_{\mathrm{b}}^2}$	$2H=1+\frac{\sigma_0^2}{\sigma_{90}^2}-\frac{\sigma_0^2}{\sigma_{\mathrm{b}}^2}$
		$2\ G=1-\frac{\sigma_0^2}{\sigma_{90}^2}+\frac{\sigma_0^2}{\sigma_{\mathrm{b}}^2}$	$2N=\frac{4\bullet {\sigma}_0^2}{\sigma_{45}^2}-\frac{\sigma_0^2}{\sigma_{\mathrm{b}}^2}$
*r*-value based	*r*_0_, *r*_45_, *r*_90_	$F=\frac{r_0}{r_{90}\bullet \left(1+{r}_0\right)}$	$H=\frac{r_0}{1+{r}_0}$
		$G=\frac{1}{1+{r}_0}$	$N=\frac{\left({r}_0+{r}_{90}\right)\bullet \left({r}_{45}+0.5\right)}{r_{90}\bullet \left(1+{r}_0\right)}$

The shear coefficients in the planes RD-ND, TD-ND (where ND is the thickness direction) have analytical formulae in terms of the yield stresses in shear in these planes (see [46]). Due to the difficulty of measuring the through thickness shear, generally it is assumed that L = M = N (same shear behaviour in the planes of orthotropy). If for isotropic behaviour, this assumption is correct, for an anisotropic sheet severely deformed, like in incremental forming for instance, this assumption could be inadequate.

Depending on the input data used to identify the Hill48 yield locus, the predictions using Hill criterion are markedly different (for examples see [13, 57]). Another methodology is to determine the parameters by numerical methods using both the experimental *r*-values and yield stresses in more than 3 orientations in conjunction with an objective function of the form:

52 $$E=\sum_i{\eta}_i{\left[\frac{{\left({\sigma}_{\theta}\right)}_i^{\mathrm{th}}}{{\left({\sigma}_{\theta}\right)}_i^{\mathrm{exp}}}-1\right]}^2+\sum_k{\eta}_k{\left[\frac{r_k^{\mathrm{th}}}{r_k^{\mathrm{exp}}}-1\right]}^2$$

In the above equation, *i* and *k* represent the number of available experimental normalized tensile stresses and *r*-ratios respectively while the superscript indicates whether the corresponding value is experimental or predicted. As pointed in “CPB06 criterion by Cazacu, Plunket and Barlat (2006)” Section, the input experimental yield stresses can be extended such as to take into account shear and biaxial elastic limits. Most of the teams just look at one point per curve: elastic limit or yield value for a defined plastic work per unit volume or strain, while another possibility is to identify in a single optimization both hardening and yield locus shape by using the total stress-strain curves like again done in “CPB06 criterion by Cazacu, Plunket and Barlat (2006)” Section. In Eq. (52), the constants *η*_*i*_ and *η*_*k*_ are weight factors. Table 22 provides the parameter values either identified by analytical formulae or by numerical optimization based on tensile data.

**Table 22:** Parameters of Hill [46] yield function obtained with different identification procedures

Method to compute Hill parameters	F	G	H	N
Provided by Organizers (UCoimbra)				
Analytical formulae of Table 21 using r_0_, r_45_, r_90_ measured by UA	0.821	0.655	0.345	1.104
ULiege Set 1:				
Based on *r*-values at 0, 15, 30, 45, 75, 90° (UA data) η_i_ = 0, η_k_ = 1 in Eq. (52)	0.756	0.669	0.330	1.119
ULiege Set 2:				
(used for deep drawing simulations)				
Based on UA tensile tests (0, 15, 30, 45, 75, 90°) and both yield stresses and r values; η_i_ = η_k_ = 0.5 in Eq. (52)	0.815	0.666	0.334	1.184
ULiege Set 3:				
Based on yield stresses (0, 15, 30, 45, 75, 90°) η_i_ = 1, η_k_ = 0 in Eq. (52)	1.045	0.914	0.086	1.661

**Fig. 48:**
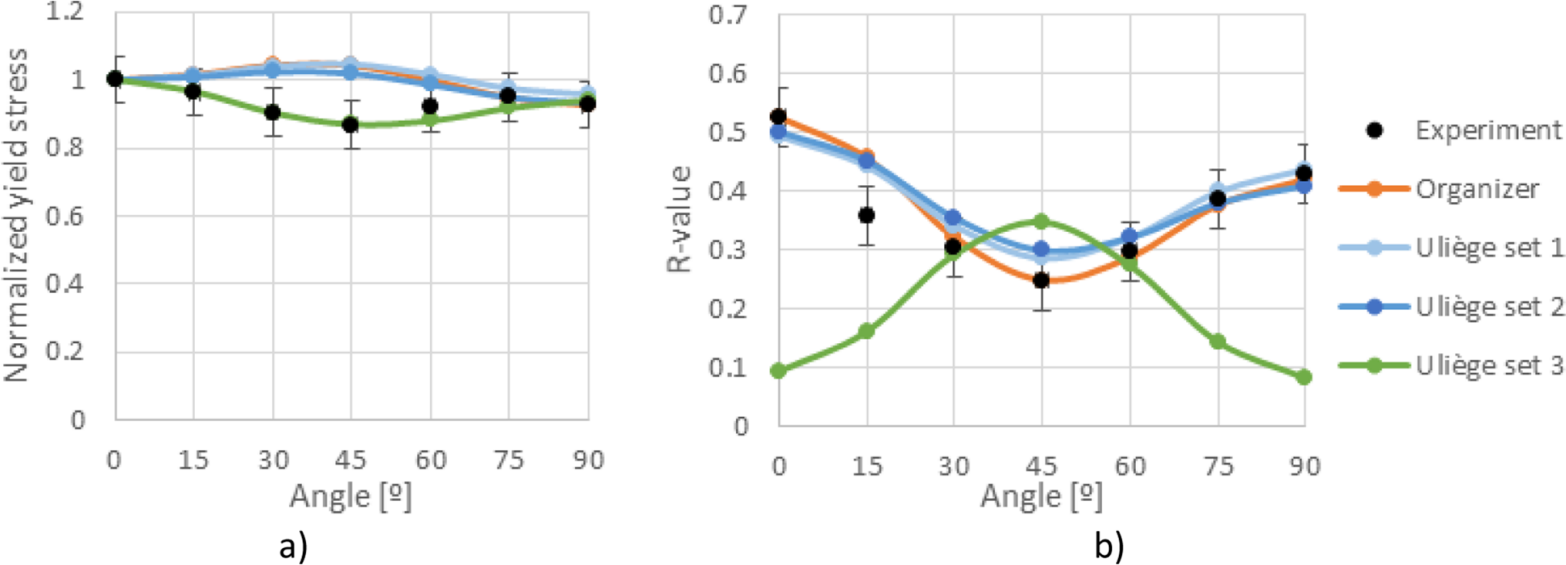
Experimental and predicted **a** directional normalized yield stresses, **b** *r*-values in uniaxial tensions (experimental data of UA) for chosen sets of parameters (in Table 22). Stresses are normalized by the yield stress along RD, *σ*_0_

**Fig. 49:**
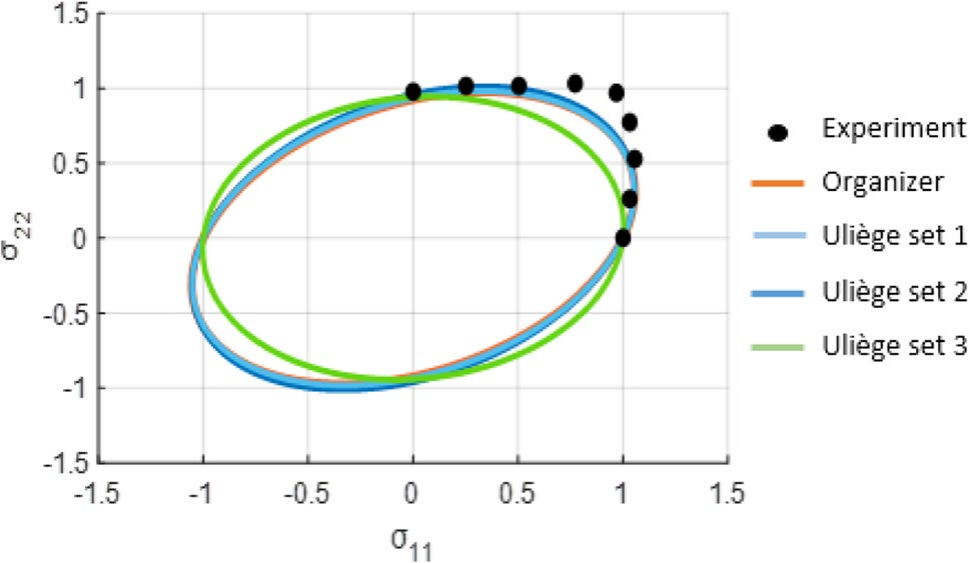
Hill yield locus cut in RD -TD orthotropic plane stress based on different data sets (details in Table 22) and experimental points (work contour at 0.2% from TUAT). Stresses are normalized by the yield stress along RD, *σ*_0_

Figures 48(a) and (b) show the strong influence of the data used for the identification on the predictions of yield stresses and Lankford coefficients. Figure 48(b) serves to illustrate the reason why the identification based on Lankford coefficients is recommended for deep drawing simulations. The Lankford coefficient is representative of the sheet thinning during the forming process and if the data set identification is focused on the stress, one can generate Lankford prediction values very far from the experimental ones. Finally, the yield locus shape (Fig. 49) reminds why more advanced anisotropic yield loci were defined. With Hill48 approach, one cannot model both the behaviour of uniaxial tensile tests and the biaxial states measured by TUAT.

#### 4^th^ order polynomial model (HomPol4)

In order to calibrate the anisotropic parameters of HomPol4 yield locus described by Eq. (10) in “2-D orthotropic yield functions (Yld89, Yld2000-2D, HomPol4, HomPol6)” Section, the mechanical test data obtained by TUAT were used. The identified parameters of HomPol4 are listed in the Table 23.

**Table 23:** Anisotropy material parameters of HomPol4

*a﻿*_1_	*a*_2_	*a*_3_	*a*_4_	*a*_5_	*a*_6_	*a*_7_	*a*_8_	*a*_9_
1	−1.379	1.953	−1.608	1.071	6.865	2.966	6.899	5.790

Here, *a*_1–9_ are the parameters related to the anisotropy of the material. The parameters *a*_1–5_ are calibrated with yield stresses and *r*-values in rolling and transverse axes in uniaxial stress state, as well as biaxial yield stress [86]. Parameters *a*_1–5_ are determined with the equations given below:

53 $${a}_1=1$$

54 $${a}_2=-\frac{4{r}_0}{\left(1+{r}_0\right)}$$

55 $${a}_3=\left(\frac{1}{{\overline{\upsigma}}_{\mathrm{b}}^4}\right)-\left({a}_1+{a}_2+{a}_4+{a}_5\right)$$

56 $${a}_4=-\frac{4{a}_5{r}_{90}}{\left(1+{r}_{90}\right)}$$

57 $${a}_5=\frac{1}{{\overline{\upsigma}}_{90}^4}$$

An optimization method was applied to determine the coefficients *a*_6_ and *a*_8_ with an objective function similar to Eq. (52), based on the error between predicted stress ratios and *r*-values and the corresponding the tensile experimental points of TUAT [80] at 15°, 30°,45°,60° and 75°. An equal weight was chosen for Lankford coefficients and yield limits. In addition, *a*_6_ and *a*_8_ have to satisfy the following inequalities to ensure the convexity conditions [86].

58 $$0\le {a}_6\le 6\sqrt{{a}_1{a}_9}$$

59 $$0\le {a}_8\le 6\sqrt{{a}_5{a}_9}$$

The parameter *a*_7_ is calculated based on *a*_6_ and *a*_8_ by using following equation:

60 $${a}_7=\left(\frac{2}{\left(1+{r}_{45}\right){\overline{\upsigma}}_{45}^4}-\frac{2}{{\overline{\upsigma}}_{\mathrm{b}}^4}\right)-\left({a}_6+{a}_8\right)$$

Regarding the convexity and the positivity measures, *a*_9_ can be determined by:

61 $${a}_9=\frac{{\left(\frac{2}{{\overline{\upsigma}}_{45}}\right)}^4{r}_{45}}{1+{r}_{45}}+{\mathrm{B}}_1>0$$

where,

62 $${\mathrm{B}}_1={a}_1+{a}_2+{a}_3+{a}_4+{a}_5={\left(\frac{1}{{\overline{\upsigma}}_{\mathrm{b}}}\right)}^4$$

Here, *r*_45_ should also ensure the following condition [86]:

63 $$\begin{aligned}&\frac{-\left[5\ {\left(2\ \frac{{\overline{\sigma}}_b}{{\overline{\sigma}}_{45}}\right)}^4+8\right]+\left(\frac{3}{2}\right){\left(2\ \frac{{\overline{\sigma}}_b}{{\overline{\sigma}}_{45}}\right)}^4{\left[{\left(2\ \frac{{\overline{\sigma}}_b}{{\overline{\sigma}}_{45}}\right)}^4+8\right]}^{1/2}}{\left[9\ {\left(2\ \frac{{\overline{\sigma}}_b}{{\overline{\sigma}}_{45}}\right)}^4+8\right]}\le {r}_{45}\\&{r}_{45}\le \frac{{\left(2\ \frac{{\overline{\sigma}}_b}{{\overline{\sigma}}_{45}}\right)}^4}{2}-1\end{aligned}$$

**Fig. 50:**
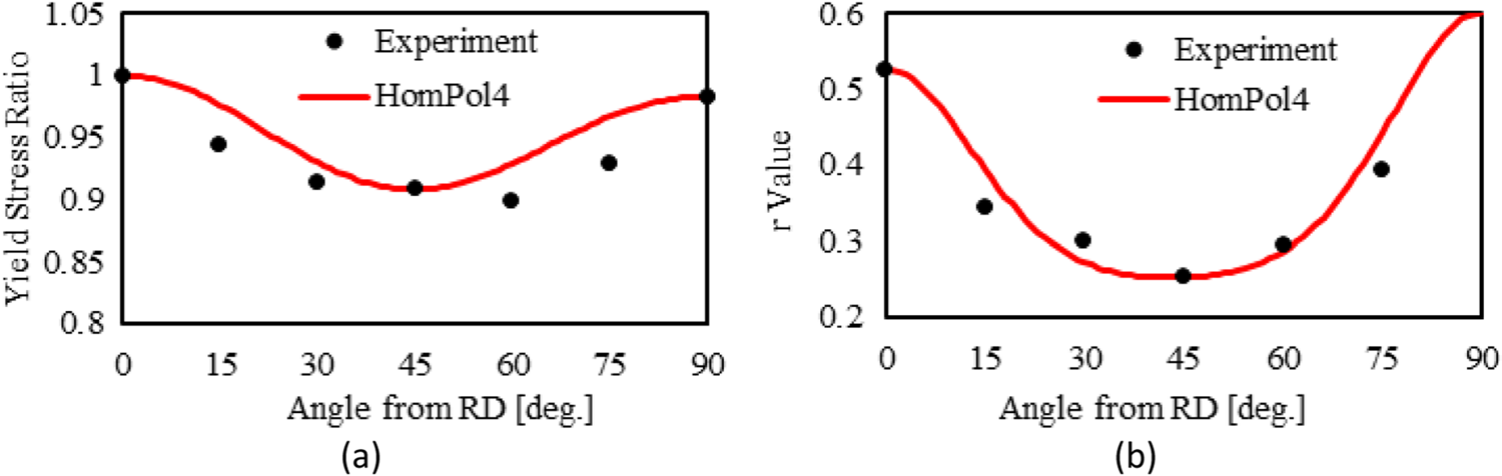
Predictions of **a** yield stress ratio and **b** Lankford coefficient values by HomPol4 with the data set of Table 23 compared with TUAT experimental values

**Fig. 51:**
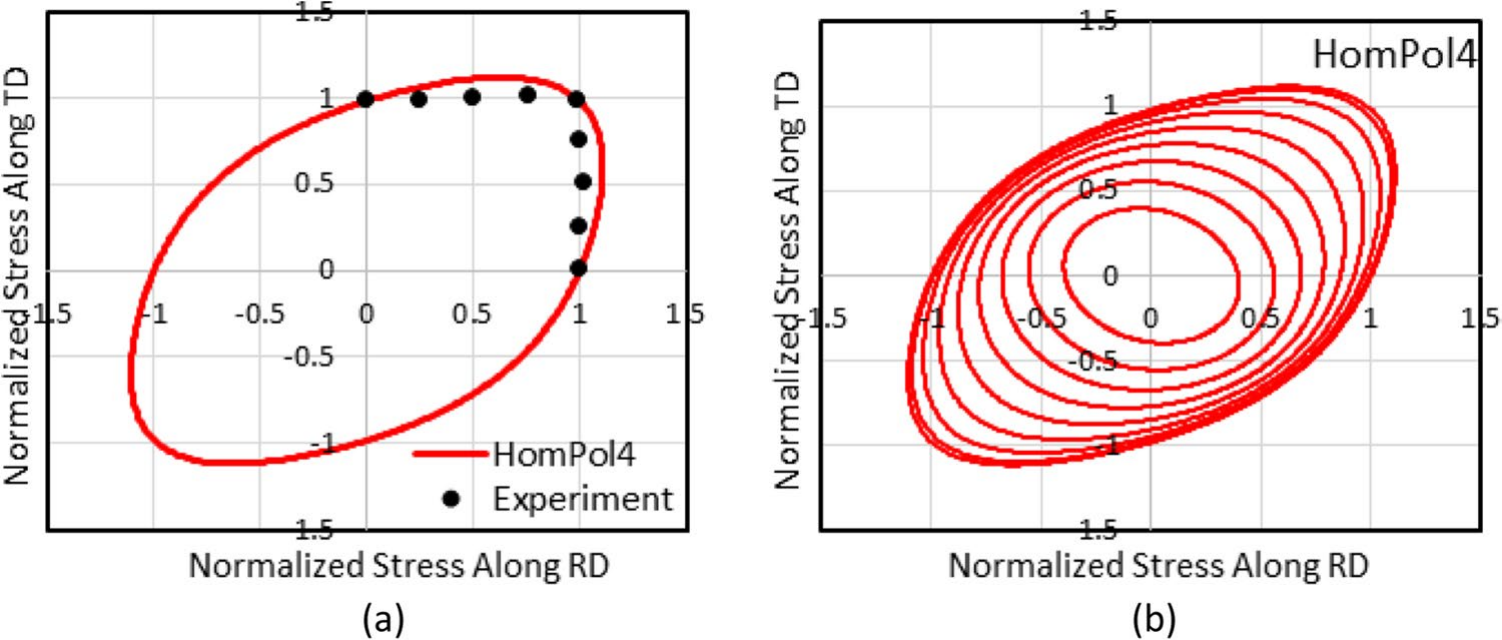
**a** Yield locus predicted by HomPol4 in principal stress space; **b** Yield loci contours predicted by HomPol4 for successive shear values of 0.1

The *r*-values and the initial yield stress in different tensile orientations are presented in Fig. 50 together with experimental results, while the yield locus cuts obtained by HomPol4 criterion for different shear stress ratios are shown in Fig. 51.

#### 6^th^ order polynomial model (HomPol6)

This yield locus described by Eq. (11) of “2-D orthotropic yield functions (Yld89, Yld2000-2D, HomPol4, HomPol6)” Section has 16 material parameters. In the identification procedure, the coefficients *a*_1_, *a*_2_, *a*_6_ and *a*_7_ are determined with the explicit formulae given in below:

64 $${r}_1=1$$

65 $${a}_2=-6{r}_0/\left(1+{r}_0\right)$$

66 $${a}_7={\left({\upsigma}_0/{\upsigma}_{90}\right)}^6$$

67 $${a}_6=-6{r}_{90}{a}_7/\left(1+{r}_{90}\right)$$

The other coefficients of the homogeneous polynomial model were determined by the minimization of an error function similar to Eq. (52). Experimental data of TUAT were used to identify the coefficients of HomPol6 for AA 6016-T4 alloy, which are given in Table 24.

**Table 24:** HomPol6 coefficients for AA 6016-T4

*a*_1_	*a*_2_	*a*_3_	*a*_4_	*a*_5_	*a*_6_	*a*_7_	*a*_8_
1.00000	−2.06815	3.53177	−3.93091	3.91107	−2.49639	1.10835	16.41230
*a*_9_	*a*_10_	*a*_11_	*a*_12_	*a*_13_	*a*_14_	*a*_15_	*a*_16_
−7.21657	11.92569	−5.02031	18.22988	22.46969	17.98091	24.43169	13.93065

**Fig. 52:**
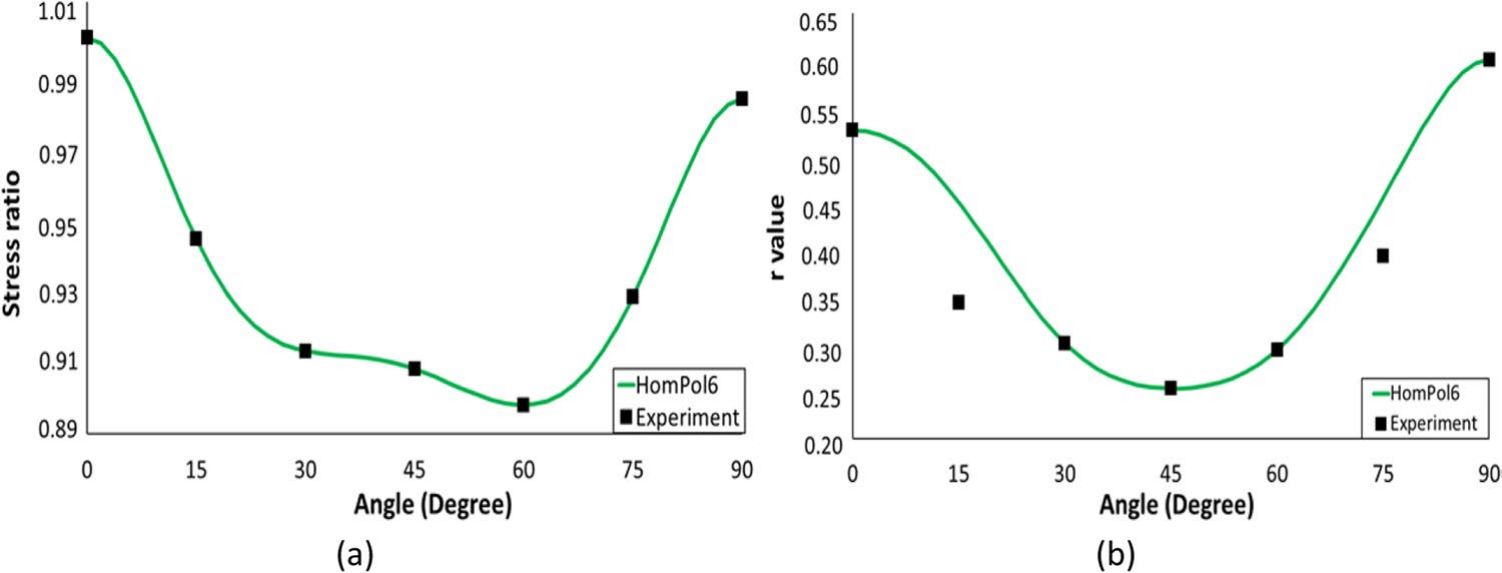
**a** Predictions of stress ratios **-** and **b** Lankford coefficients for tensile tests in different directions based on HomPol6 yield locus and TUAT experimental results (angles measured from Rolling Direction)

**Fig. 53:**
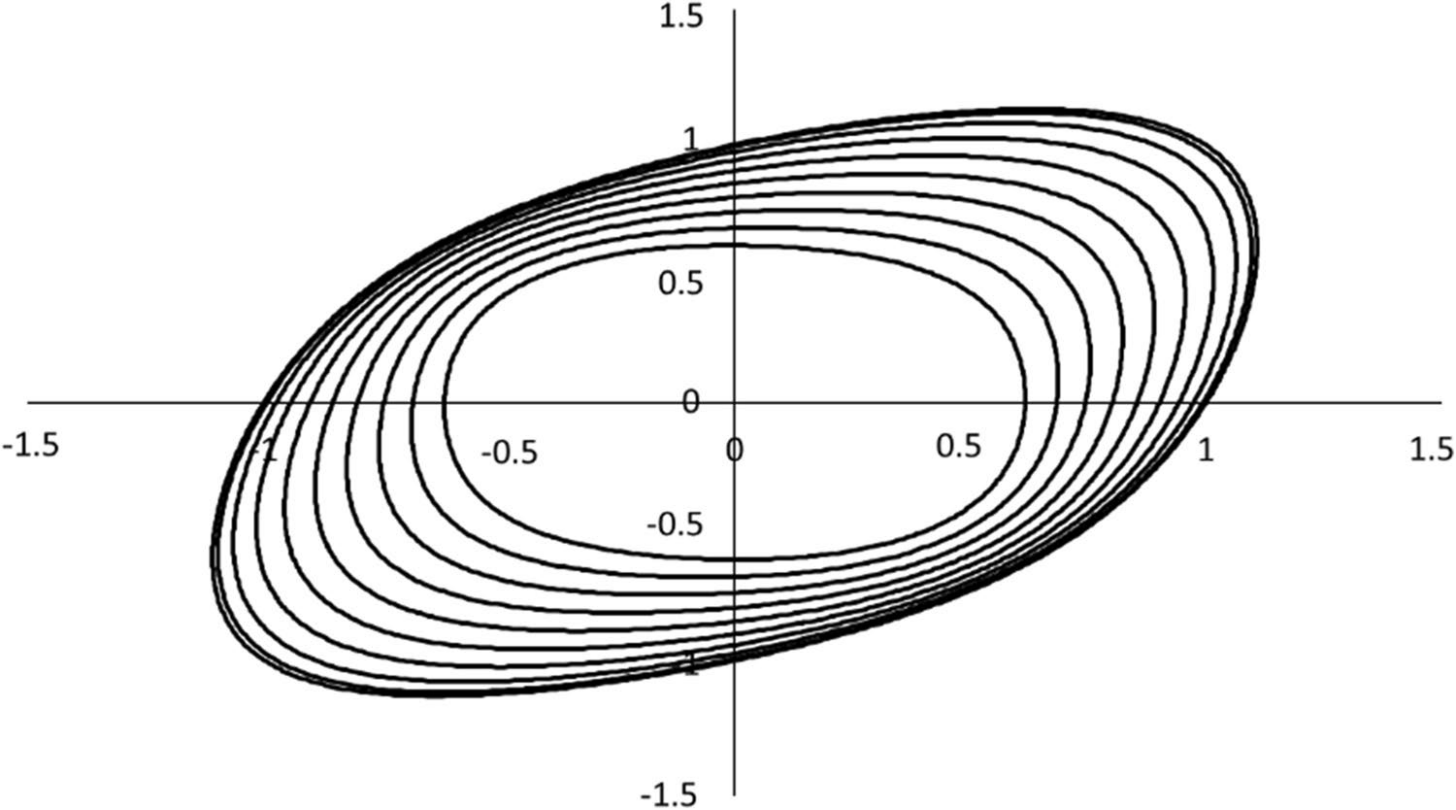
The computed contours of HomPol6 yield locus with the data set defined in Table 24

Figure 52 shows the predictions of planar variations of plastic properties (stress ratio and *r*-values). HomPol6 yield criterion can simultaneously predict planar variations of yield stress ratio and *r*-values. The computed yield locus contours are smooth and convex as observed in Fig. 53, confirming that the determined coefficients satisfy the convexity condition.

#### Non quadratic plane-stress yield locus Barlat (Yld89)

In this section, the identification methodology of Yld89 criterion (Eq. (7), “2-D orthotropic yield functions (Yld89, Yld2000-2D, HomPol4, HomPol6)” Section) and the validation of its results for AA 6016-T4 aluminium alloy are described. As previously mentioned, it is recommended to select the* m* exponent in function of the crystal structure of the material, i.e. FCC *m* = 8 and BCC *m* = 6 [65]. The other material constants present in Eq. (7): *a* and *h* are obtained through *r*_*0*_*, r*_*45*_, and *r*_*90*_:

68 $$a=2-2\sqrt{\left(\frac{r_0}{1+{r}_0}\right)\left(\frac{r_{90}}{1+{r}_{90}}\right)}$$

69 $$h=\sqrt{\left(\frac{r_0}{1+{r}_0}\right)\left(\frac{1+{r}_{90}}{r_{90}}\right)}$$

while *p* parameter can be found by optimization. In this current benchmark, the tensile tests performed by TUAT were used and the objective function (Eq. 52) was applied, with *η*_i_ = 0,* η*_k_ = 1, which means only the *r*-value error was minimized. Note this Yld89 model is often used in finite element analyses since its implementation is easy and it requires only a few numbers of material parameters.

**Fig. 54:**
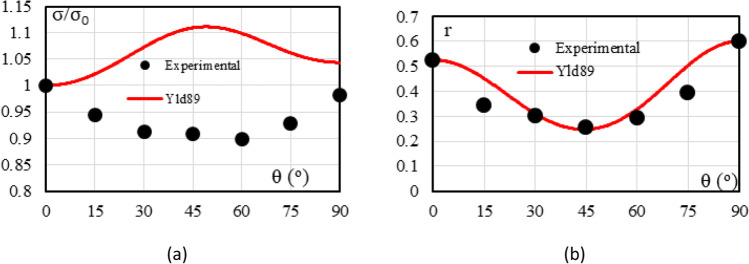
Prediction of angular variation of **a** normalized yield stress and **b** Lankford coefficient obtained with Yld89 compared with TUAT experimental points

**Fig. 55:**
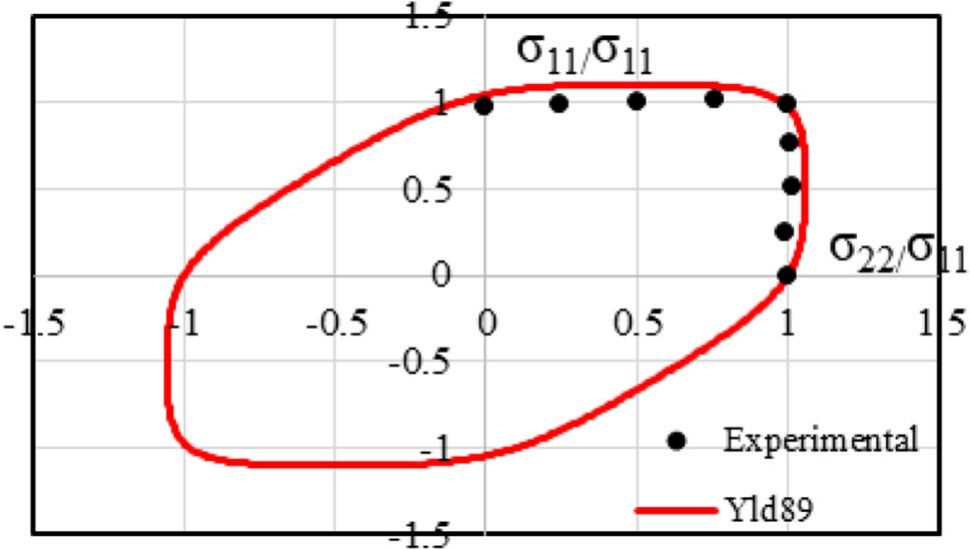
Yield locus cut of AA 6016-T4 obtained with Yld89

The material parameters of the Yld89 criterion for the AA 6016-T4 alloy are given in Table 25. The Lankford coefficient and the normalized uniaxial flow stress for different directions defined by their angle with RD are presented in Fig. 54, with the experimental results of TUAT. It can be seen that the directionality of *r*-values obtained with Yld89 has a good agreement with the experimental results except for 15^o^ and 75^o^, while the criterion shows significantly poor performance for yield stress ratio predictions (see Fig. 54(a)). The yield locus cut shown in Fig. 55 presents a close agreement with the biaxial-points measured by TUAT.

**Table 25:** Coefficients of Yld89 criterion for 6016-T4 aluminium alloy

*a*	*h*	*p*	*m*
1.2806	0.9582	0.86	8

#### CB2001 by Cazacu and Barlat (2001)

The experimental data used for the identification of the CB2001 orthotropic yield criterion [12] for the benchmark material consisted of the uniaxial tensile data generated at UA. The anisotropy coefficients and parameter *c* involved in this criterion (see Eq. 15) were determined by minimizing an error function of the form:

70 $$E=\sum_i{\eta}_i{\left[\frac{{\left({\sigma}_{\theta}\right)}_i^{\mathrm{th}}}{{\left({\sigma}_{\theta}\right)}_i^{\mathrm{exp}}}-1\right]}^2+\sum_k{\eta}_k{\left[\frac{r_k^{\mathrm{th}}}{r_k^{\mathrm{exp}}}-1\right]}^2+\sum_l{\eta}_l{\left[\frac{\rho_l^{\mathrm{th}}}{\rho_l^{\mathrm{exp}}}-1\right]}^2$$

In the above equation, *i*, *k*, *l* represent respectively the number of available experimental normalized tensile yield stresses and *r*-ratios (from UA) and biaxial data (from TUAT), while the superscript indicates whether the corresponding value is experimental or predicted. In Eq. (70), the constants *η*_*i*_, *η*_*k*_ and *η*_*l *_are weight factors and were taken equal. To be recalled that a biaxial stress data point is represented by: $\rho =\sqrt{{\left({\sigma}_{xx} cos\varphi \right)}^2+{\left({\sigma}_{yy} sin\varphi \right)}^2}$ with *φ* =  *arctan* (*σ*_*yy*_/*σ*_*xx*_) (see also “Biaxial tensile tests” Section). The parameters for the yield criterion are provided in Table 26.

**Table 26:** Values of material parameters of CB2001, the orthotropic model [12]

*a*_1_	*a*_2_	*a*_3_	*a*_4_	*a*_5_	*a*_6_	*c*				
1.000	1.900	1.391	0.870	1.000	1.000	1.2				
*b*_1_	*b*_2_	*b*_3_	*b*_4_	*b*_5_	*b*_6_	*b*_7_	*b*_8_	*b*_9_	*b*_10_	*b*_11_
2.000	0.830	1.500	2.000	0.200	1.000	1.000	1.000	1.000	0.566	1.000

**Fig. 56:**
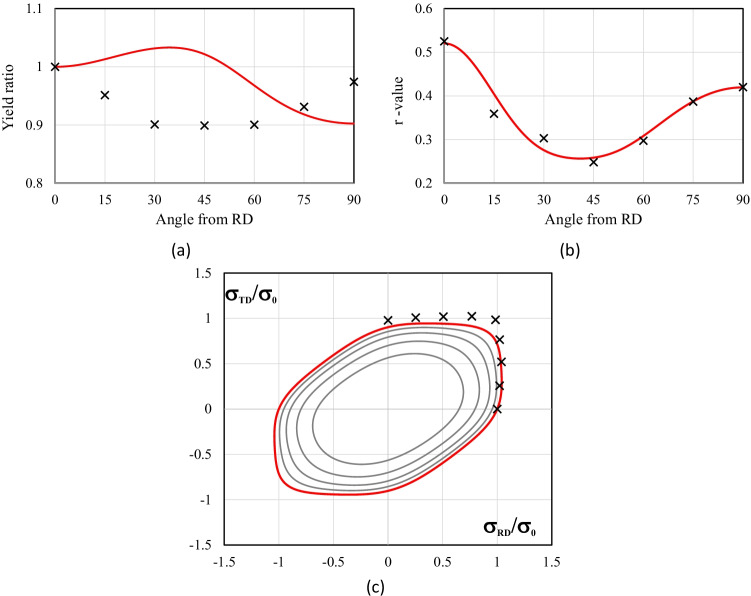
Predicted results by CB2001 criterion (Eq. 15): **a** Tensile flow stresses; **b** Lankford coefficients; **c** Yield surface section in the biaxial plane (*σ*_RD_, *σ*_TD_) (with *σ*_*xy*_/*σ*_0_ *=* 0,0.2,0.3,0.4,0.5). Data represented by symbols. All stresses are normalized by the yield stress along the rolling direction, *σ*_0_

**Fig. 57:**
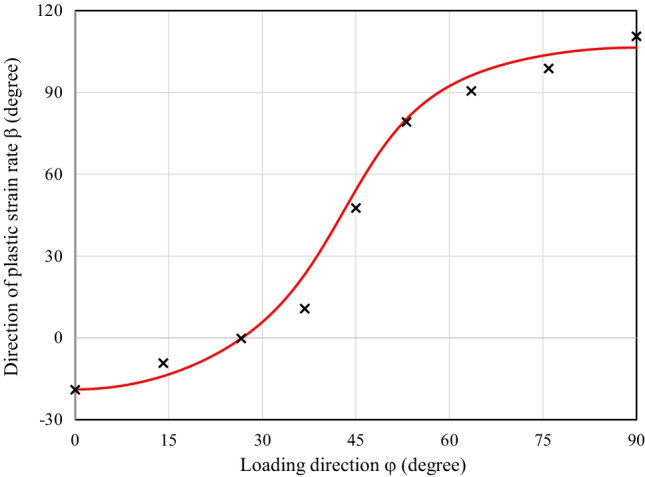
Predicted direction of the plastic strain-rate ${\beta} ={\arctan}\left({D}_{yy}^\mathrm{p}/{D}_{xx}^\mathrm{p}\right)$ for biaxial loading at *φ =  arctan* (*σ*_RD_/*σ*_TD_) by CB2001 criterion. Data represented by cross symbols

Comparison between experimental and predicted anisotropy in yield stresses and *r*-ratios obtained with CB2001 and associated flow rule are shown in Fig. 56(a)-(b). The predicted yield surface cross-section in the plane (*σ*_*xx*_,*σ*_*yy*_) corresponding to constant levels of shear, namely *σ*_*xy*_/*σ*_0_ = 0,0.2,0.3,0.4,0.5 along with available biaxial stress data (TUAT) are shown in Fig. 56(c). Moreover, Fig. 57 shows the predicted direction of the plastic strain-rate $\beta =\mathit{\arctan}\left({D}_{yy}^{\mathrm{p}}/{D}_{xx}^{\mathrm{p}}\right)$ for biaxial loading characterized by the angle *φ* =  *arctan* (*σ*_RD_/*σ*_TD_) in comparison to the experimental data.

#### Yld2000-2D and Yld 2004-18p

POSTECH, as benchmark organizer provided dataset for the participants not performing their own identification. Based on the uniaxial tensile tests from TUAT, the Yld2000-2D and Yld2004–18p coefficients were determined with an optimization algorithm based on the steepest descent method with normalized flow stresses and *r*-values, for different stress states employed as input data. The normalized flow stress is taken at 20.6 MPa plastic work (corresponding to 0.1 plastic strain). The coefficients for Yld2000-2D (Eq. (8), “2-D orthotropic yield functions (Yld89, Yld2000-2D, HomPol4, HomPol6)” Section) and Yld2004–18p (Eq. (18), “3‐D orthotropic yield functions (Hill48, CB2001, Yld2004–18p, CPB06ex2, Caz2018‐Orth)” Section), obtained by taking the yield function exponent *m* = 8 are presented in Table 27 and Table 28, respectively. UGent approach to identify Yld2000-2D has already been described in “VPSC model” Section, as virtual crystal plasticity tests are involved.

**Table 27:** Coefficients of Yld2000-2D (Line 1: POSTECH identification method based on classical experiments and Line 2: UGent identification method based on virtual tests)

*α*_1_	*α*_2_	*α*_3_	*α*_4_	*α*_5_	*α*_6_	*α*_7_	*α*_8_	*m*
0.9238	0.9967	0.9365	1.0227	1.0303	1.0075	0.8385	1.3761	8
0.6062	1.2813	1.2030	1.0214	1.0333	0.8686	0.7258	1.3764	6.11

**Table 28:** Coefficients of Yld2004–18p associated with the common choice of *m* = 8 (Line 1 from POSTECH, Line 2 from NTNU, both based on physical experiments, and Line 3 from NTNU based on virtual tests by Damask crystal plasticity simulations)

${c}_{12}^{\prime }$	${c}_{13}^{\prime }$	${c}_{21}^{\prime }$	${c}_{23}^{\prime }$	${c}_{31}^{\prime }$	${c}_{32}^{\prime }$	${c}_{44}^{\prime }$	${c}_{55}^{\prime }$	${c}_{66}^{\prime }$
1	1	−0.0870	0.4496	1.0192	1.1724	1.2801	1.1487	0
1.1998	1.2289	0.1315	0.8081	1.1386	0.2870	1.0000	1.0000	1.3739
1.1718	1.1284	−0.0403	0.6912	1.1030	0.4439	1.5916	0.0011	0.9922
${c}_{12}^{\prime \prime }$	${c}_{13}^{\prime \prime }$	${c}_{21}^{\prime \prime }$	${c}_{23}^{\prime \prime }$	${c}_{31}^{\prime \prime }$	${c}_{32}^{\prime \prime }$	${c}_{44}^{\prime \prime }$	${c}_{55}^{\prime \prime }$	${c}_{66}^{\prime \prime }$
1.3329	1.2510	1.0948	1.0855	−0.0065	0.4812	0.5991	0.7366	1.5461
1.1510	−0.2576	0.4873	0.9934	0.9110	0.7965	1.0000	1.0000	0.2679
1.2910	−0.1740	0.7416	1.0787	1.0278	0.9367	−0.0021	1.5917	0.8146

**Fig. 58:**
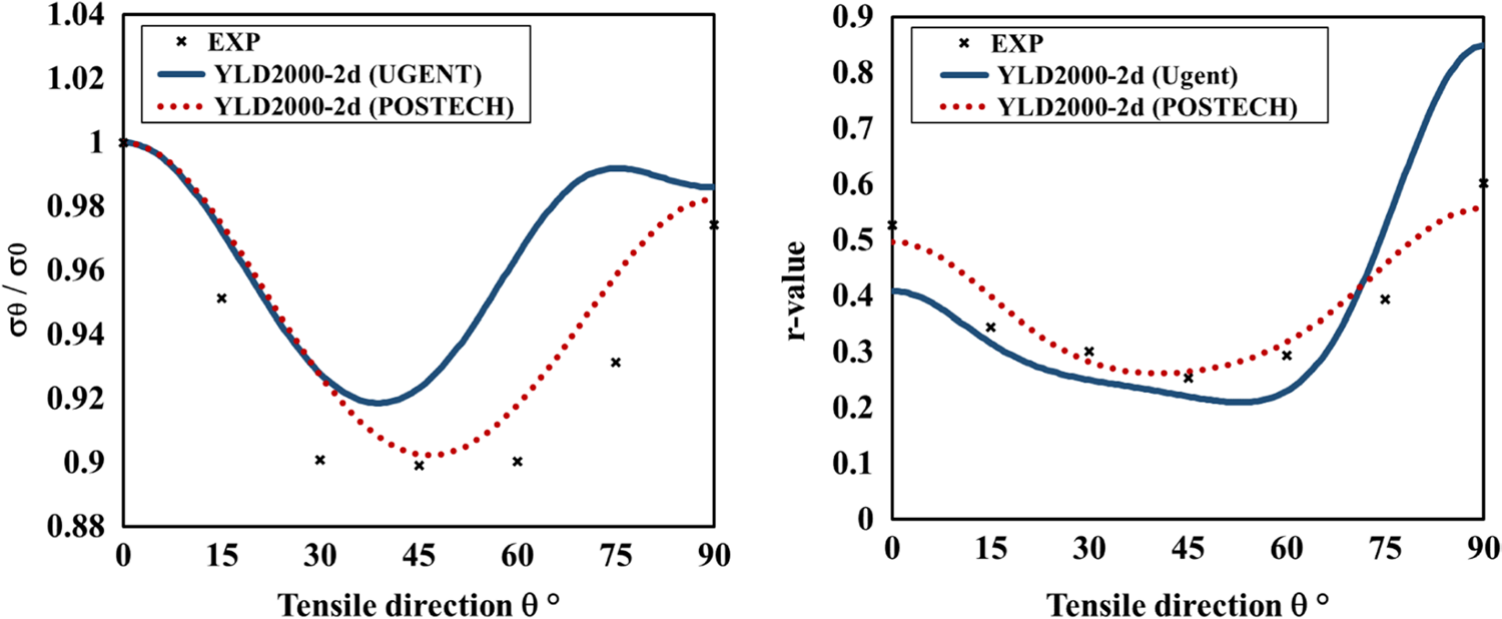
Validation of Yld2000-2D predictions by UGent and POSTECH vs. experiments at 10% of effective plastic strain: **a** normalized yield stresses and **b** *r*-values

**Fig. 59:**
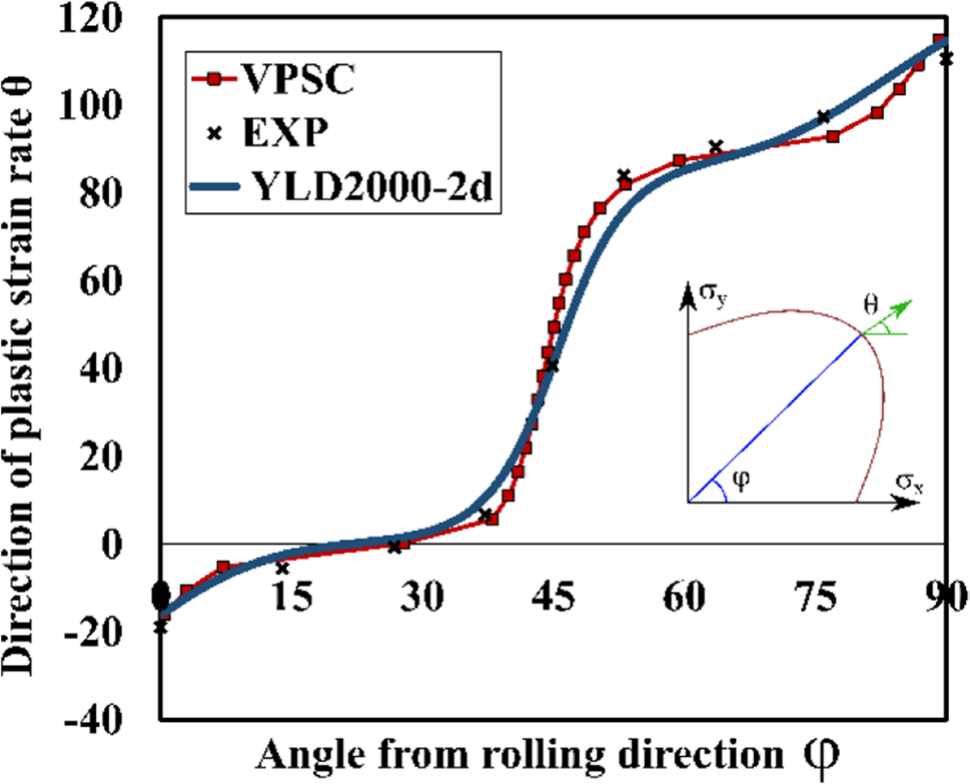
Directions of plastic strain rate obtained in the experimental tests and predicted by VPSC and Yld2000-2D as a function of the angle ***φ*** at 10% of effective plastic strain

Comparisons between the experimental and predicted (Yld2000-2D) yield stresses and *r*-values, with respect to the angle *θ*, based on the data sets of Table 27 are shown in Fig. 58(a) and (b), respectively. Note that the UGent calibration procedure for Yld2000-2D by VPSC model is further validated in Fig. 59 by comparing the measured evolution of the direction of plastic strain rates with the predicted ones by VPSC and Yld2000-2D simulations.

The flexible model Yld2004–18p proposed in [7] and described by Eq. (18), “3-D orthotropic yield functions (Hill48, CB2001, Yld2004-18p, CPB06ex2, Caz2018-Orth)” Section, was identified by POSTECH based on physical experiments, but also by NTNU using the same kind of experimental data and also relying on crystal plasticity results (see “RVE Damask Simulations” Section). Their sets of parameters are provided in Table 28.

**Fig. 60:**
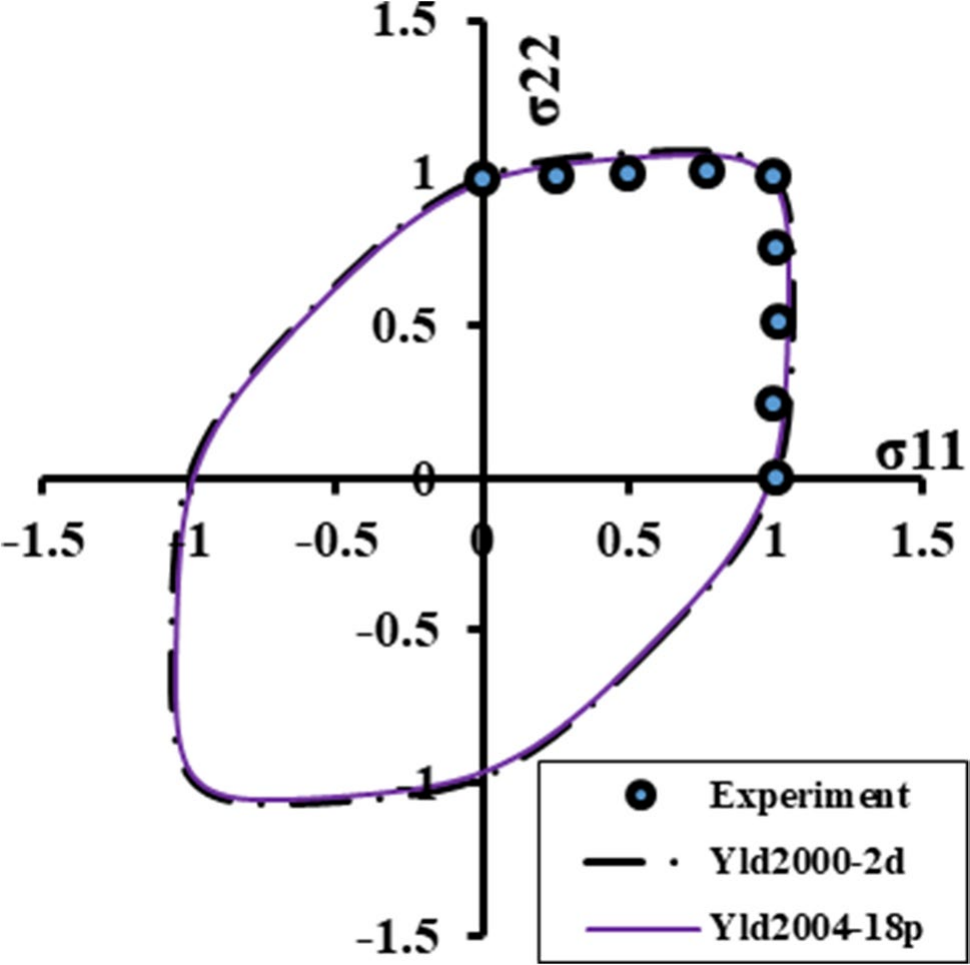
Comparison of the experimental yield locus and the predictions using Yld2000-2D and Yld2004–18p identified by POSTECH on physical experiments

**Fig. 61:**
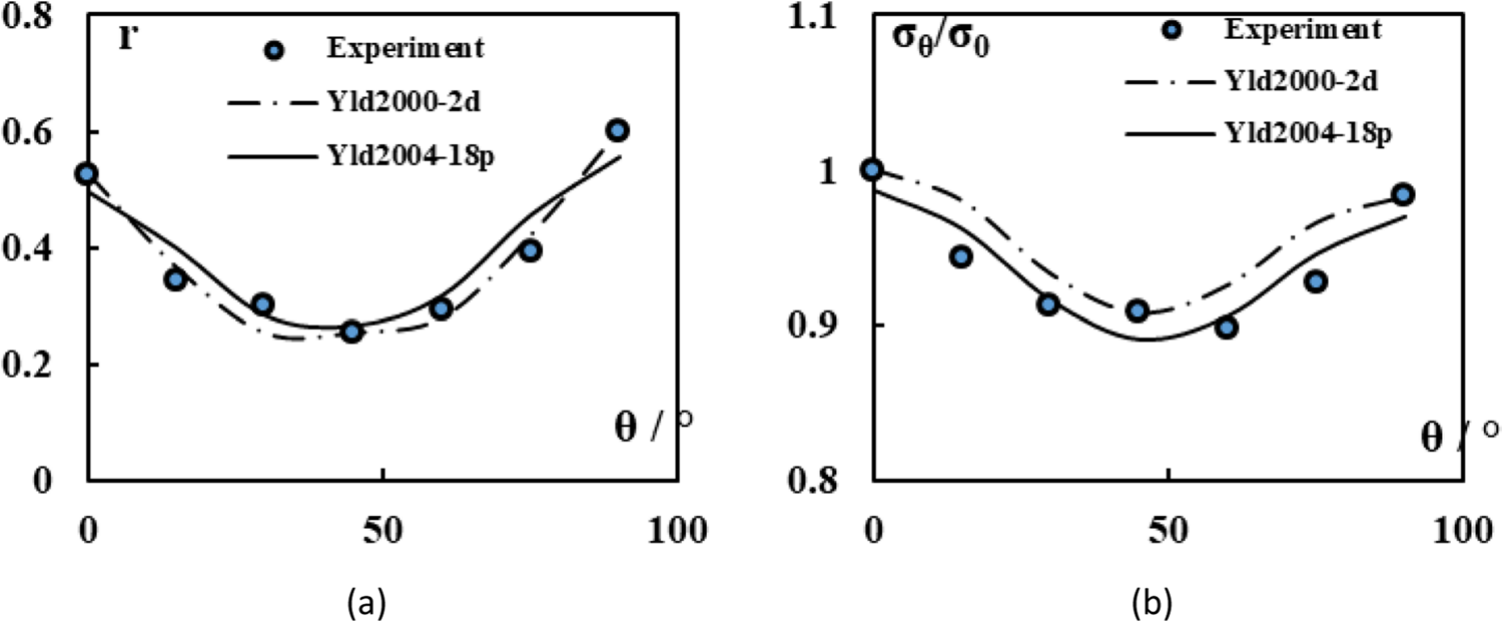
Comparison of the experimental with the predictions using Yld2000-2D and Yld2004–18p identified by POSTECH on physical experiments: **a** normalized flow stress and **b** *r*-values

The results of POSTECH enhance a comparison between Yld2000-2D and Yld2004–18p predictions results (see yield locus cut in Fig. 60 and anisotropy properties in Fig. 61). Let us remind that the predicted anisotropic properties by NTNU were already presented in “RVE Damask Simulations” Section. They are directly based on virtual crystal plasticity experiments (Square Dots in Fig. 40) or predicted by the Yld2004–18p identified from those virtual data (blue curves in Fig. 40). The predictions based on the yield locus identified by crystal plasticity show some discrepancy with the experiments (overestimation, although the trends are good), while the results generated by Yld2004–18p directly identified by NTNU based on physical experiments (red curves in Fig. 40) are similar to the experimental curves shown in Figs. 5 and 61.

#### CPB06 criterion by Cazacu, Plunket and Barlat (2006)

The procedure to determine the material parameters of the model developed by Cazacu, Plunket and Barlat [14] is described hereafter. The experimental data used for the simultaneous identification of the Voce hardening law (Eq. 4) and the CPB06ex2 orthotropic yield locus (Eq. (22) of “3-D orthotropic yield functions (Hill48, CB2001, Yld2004-18p, CPB06ex2, Caz2018-Orth)” Section) consisted of the uniaxial tensile and shear tests performed by UA, as well as the biaxial data, generated at TUAT. These experiments assume uniform simple stress state, even if for simple shear and large shear strains, it is clearly more complex. These stress states can be implemented within an analytical integration of the constitutive law or computed by an element simulation with a FE software, to identify associated equivalent anisotropy stress and strain values, stress-strain curves and Lankford coefficients.

The identification strategy consists in changing the parameters of the yield criterion and the hardening law, in order to minimize an error function similar to Eq. (52). Three differences are noted in this approach. Here, the whole stress-strain curve is taken into account using from 6 to 50 equivalent strain levels to compute the difference between the predicted and measured values. Another weight ponderation is applied: for tensile state, shear state, biaxial point and Lankford coefficient, values of 3, 1, 1, 1 are used, respectively. Note that seven biaxial equivalent stress-strain curves were also included in the error function. The parameters for the Voce hardening law are reported in Table 32, which gathers all sets of hardening values, while the parameters for the yield criterion are provided in Table 29.

**Table 29:** Anisotropy parameter values of the CPB06ex2 yield criterion (*k = k*^′^ *=* 0)

*C*_11_	*C*_22_	*C*_33_	*C*_44_ = *C*_55_	*C*_66_	*C*_12_	*C*_13_	*C*_23_	
1.0000	1.0610	0.1421	1.0000	1.0086	0.2321	−0.5621	−0.3188	
${C}_{11}^{\prime }$	${C}_{22}^{\prime }$	${C}_{33}^{\prime }$	${C}_{44}^{\prime }={C}_{55}^{\prime }$	${C}_{66}^{\prime }$	${C}_{12}^{\prime }$	${C}_{13}^{\prime }$	${C}_{23}^{\prime }$	*a*
1.1065	0.4034	1.1488	1.0000	1.1017	−0.3162	0.3983	−0.3531	8

**Fig. 62:**
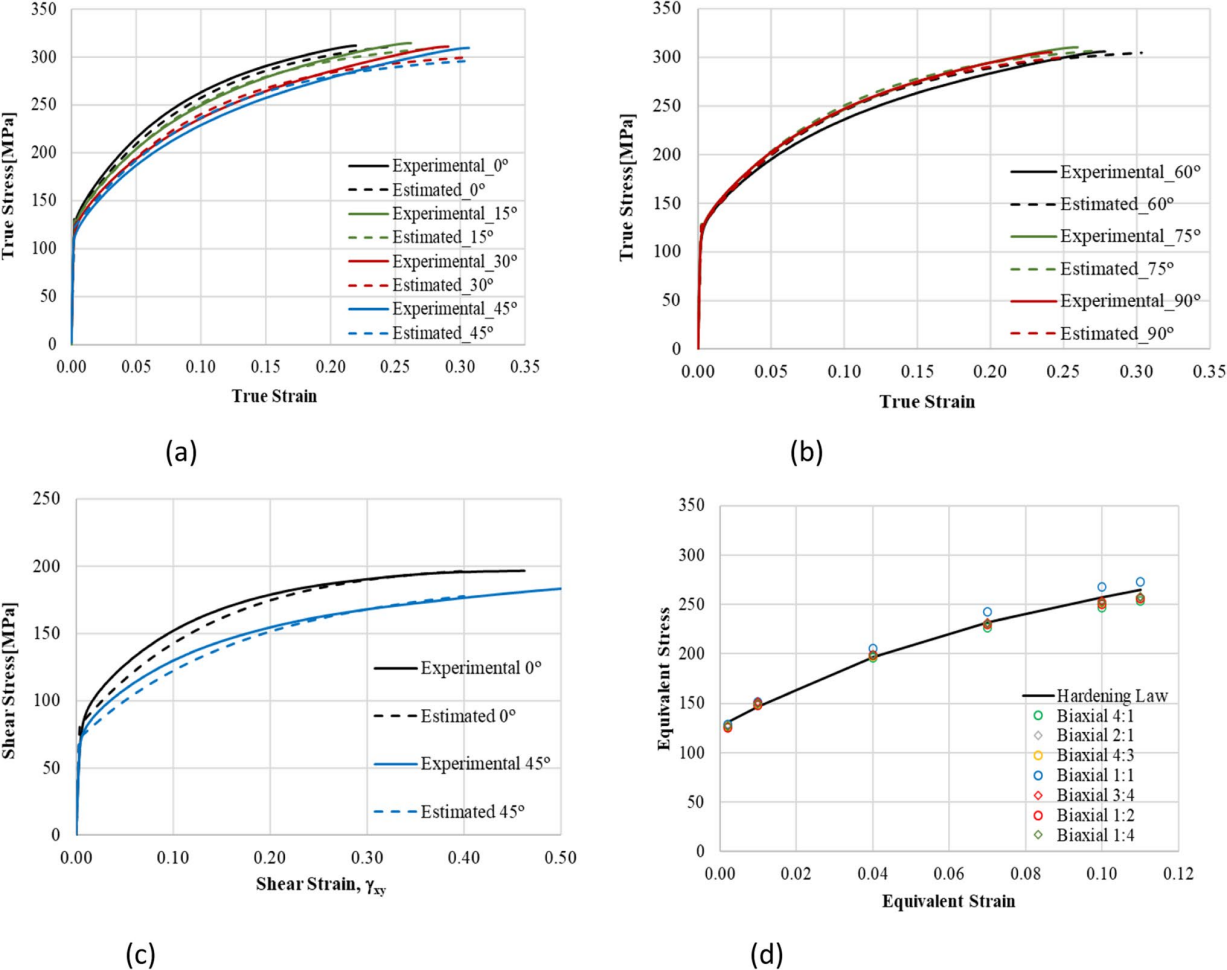
Comparison between the experimental and the CPB06ex2 predicted values: **a** and **b** true stress vs. true strain curves, for uniaxial tensile tests; **c** shear stress vs. shear strain curves; and **d** equivalent stress vs. equivalent strain curve, for biaxial tests

The comparison between the experimental and predicted true stress vs. true strain curves for uniaxial tensile tests is presented in Fig. 62(a) and (b), where it is possible to observe that the model underestimates the hardening behaviour for RD. Fig. 62(c) presents the comparison between the experimental and predicted shear stress vs. shear strain curves, highlighting higher differences for shear strains lower than 0.25. In Fig. 62(d), the comparison between the predicted equivalent stress vs. equivalent strain curve with the experimental results from biaxial tests, for different reference plastic strain values is performed. Globally, it is observed that the equibiaxial stress is underestimated by the model, while the others stress ratios and the hardening are overestimated.

**Fig. 63:**
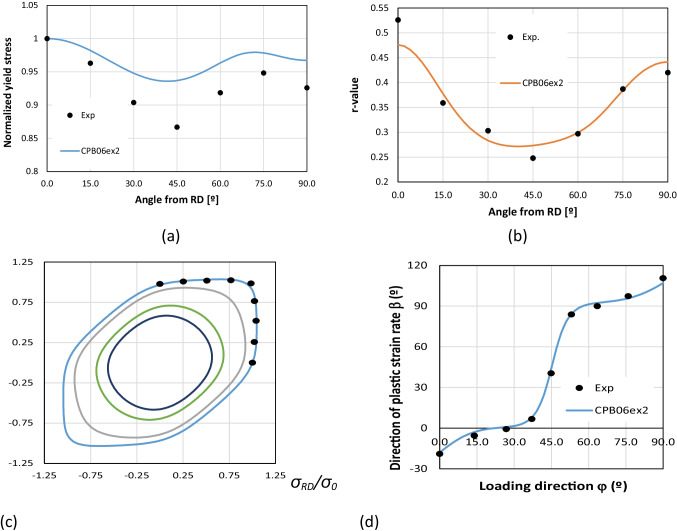
Predicted anisotropy by CPB06ex2 orthotropic yield criterion: **a** Uniaxial tensile flow stresses; **b** Lankford coefficients (*r*-values); **c** Predicted yield surface section in the biaxial plane *σ*_RD_ *σ*_TD_ (with *σ*_*XY*_*/σ*_*0*_ *= 0.,* 0.2, 0.3, 0.4 and 0.5). Data for the reference plastic strain of 0.002 are represented by symbols. All stresses are normalized by the yield stress along the RD *σ*_*0*_; **d** Predicted direction of the plastic strain-rate β for biaxial loading at φ. Data are presented by symbols

The comparisons between experimental and predicted anisotropy in yield stresses and *r*-ratios obtained with the CPB06ex2 yield criterion and associated flow rule are shown in Fig. 63(a) and (b). The predicted yield surface cross-sections for constant levels of shear (normalized by the elastic limit in RD), namely 0, 0.2, 0.3, 0.4 and 0.5 along with available experimental biaxial stress data (from TUAT) are shown in Fig. 63(c). Figure 63(d) presents the predicted direction of the plastic strain-rate for biaxial loading characterized by the angle β (see angle definition in Fig. 41(b)) in comparison to the experimental data. It is to be noted that the experimental data associated with the direction of the plastic strain-rate β for biaxial tests have not been used in the identification procedure. However, a very good agreement is observed.

#### Caz2018-Orth or Cazacu (2018) orthotropic yield criterion

For the benchmark material, the experimental data used for identification of the orthotropic yield criterion called Caz2018-Orth (details in [15]) consisted of the uniaxial tensile data generated at UA. Specifically, the anisotropy coefficients *a*_*k*_, *k* = 1…6 and *b*_*j*_, *j* = 1…11 and parameter *α* (see Eq. (13), (14) and (16), in “3-D orthotropic yield functions (Hill48, CB2001, Yld2004-18p, CPB06ex2, Caz2018-Orth)” Section) were determined by the REEF organizers by minimizing the error function given by (Eq. 52) with equal weights on stresses and Lankford coefficients. The parameters for the yield criterion are provided in Table 30.

**Table 30:** Values of material parameters of Caz2018-Orth, the orthotropic Cazacu [15] model determined by the organizer REEF

*a*_1_	*a*_2_	*a*_3_	*a*_4_	*a*_5_	*a*_6_	*α*				
0.311	0.505	0.444	0.309	0.309	0.309	2.7				
*b*_1_	*b*_2_	*b*_3_	*b*_4_	*b*_5_	*b*_6_	*b*_7_	*b*_8_	*b*_9_	*b*_10_	*b*_11_
0.253	0.206	0.223	0.288	0.220	0.174	0.205	0.220	0.213	0.213	0.172

**Fig. 64:**
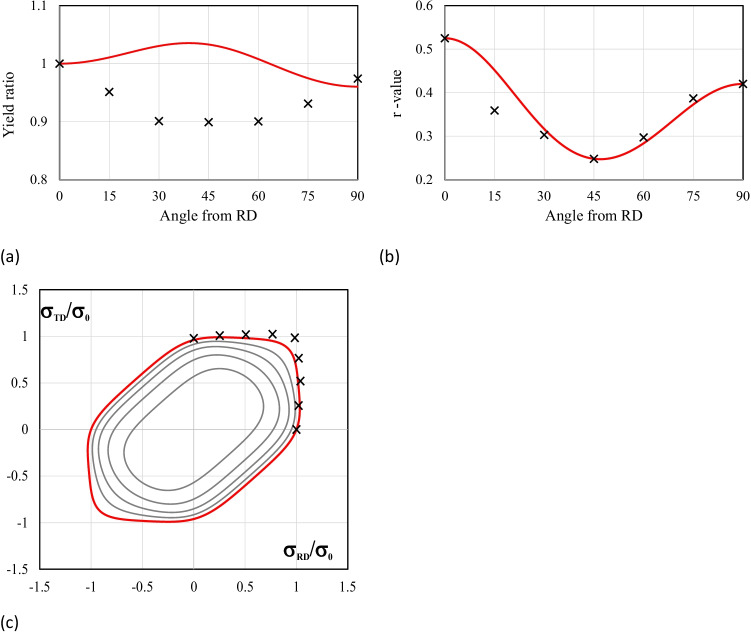
Predicted anisotropy by Caz2018-Orth yield criterion (Eq. 16): **a** Uniaxial tensile flow stresses; **b** Lankford coefficients (*r*-values); **c** Predicted yield surface section in the biaxial plane (*σ*_RD_, *σ*_TD_) (with *σ*_*xy*_/*σ*_0_ *=* 0,0.2,0.3,0.4,0.5). Data are presented by cross symbols. All stresses are normalized by the yield stress along the rolling direction *σ*_0_

**Fig. 65:**
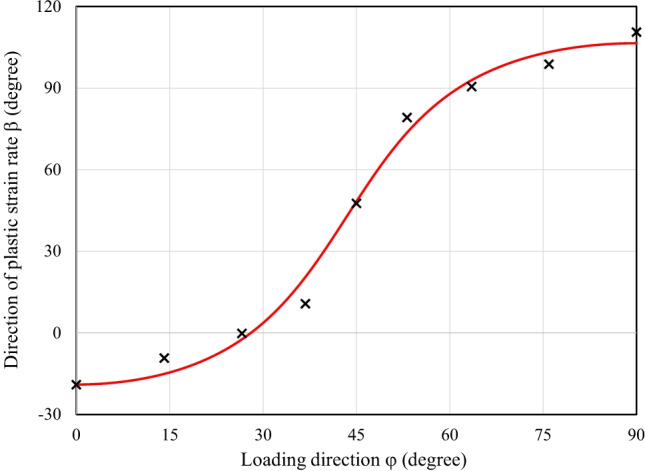
Predicted direction of the plastic strain-rate β for biaxial loading at φ by Caz2018-Orth yield criterion. Data are presented by cross symbols, angles β and φ are defined in Fig. 41(b)

Comparison between experimental and predicted anisotropy in yield stresses and *r*-ratios obtained with Caz2018-Orth yield locus and associated flow rule are shown in Fig. 64(a)-(b). The predicted yield surface cross-section in the plane (*σ*_RD_, *σ*_TD_) corresponding to constant levels of shear, namely with *σ*_*xy*_/*σ*_0_ = 0,0.2,0.3,0.4,0.5 along with available biaxial stress data (from TUAT) are shown in Fig. 64(c). Figure 65 presents the predicted direction of the plastic strain-rate β in comparison to the experimental data. It is to be noted that none of the experimental data for biaxial testing have been used for identification.

#### Hill48 and non-associated flow rule

For non-associative Hill model, as reminded at the end of the introduction of “Main features of constitutive laws” Section, 12 anisotropy parameters are necessary. Six for defining the yield stress function *f* and six for the flow potential *g*. The calibration for the anisotropy parameters is performed by analytical formulae defined hereafter by Eqs. (71), similar to the one described in associated Hill48 model (Table 21). They are reminded here to clearly define notations and to enhance that UAalto model assumes distortional hardening as explained hereafter.

The flow potential controls the update of strain components according to the non-associated flow rule Eq. (1)_4_. This approach provides a new definition of the equivalent plastic strain rate ${\dot{\overline{\varepsilon}}}^{\mathrm{p}}$, which is not equal to the conventional non-negative plastic multiplier $\dot{\lambda}$, the details can be found in [63].

As shown by many experiments in the literature [89], during the monotonic and proportional plastic deformation process of anisotropic material, the hardening is not isotropic, but rather anisotropic and/or distortional. In addition to the hardening behaviour, the plastic anisotropy (*r*-values) is also not constant (see Fig. 5(c)), as assumed by most of the benchmark participants which define an average value or a value associated to a specific stage of the strain (see Table 4). An evolving non-associated Hill48 model has been formulated with a straight updating of the anisotropic parameters, by the stress–strain curves and *r*-value evolution curves, as a function of the equivalent anisotropic plastic strain from uniaxial and biaxial tests [63]. Therefore, the parameters are defined as follows:

71 $${\displaystyle \begin{array}{c}{F}_{\upsigma}=\frac{\sigma_0^2\left({\overline{\varepsilon}}^{\mathrm{p}}\right)}{\sigma_{90}^2\left({\overline{\varepsilon}}^{\mathrm{p}}\right)}-1+\frac{\sigma_0^2\left({\overline{\varepsilon}}^{\mathrm{p}}\right)}{\sigma_{\mathrm{b}}^2\left({\overline{\varepsilon}}^{\mathrm{p}}\right)}\\ {}{H}_{\upsigma}=1+\frac{\sigma_0^2\left({\overline{\varepsilon}}^{\mathrm{p}}\right)}{\sigma_{90}^2\left({\overline{\varepsilon}}^{\mathrm{p}}\right)}-\frac{\sigma_0^2\left({\overline{\varepsilon}}^{\mathrm{p}}\right)}{\sigma_{\mathrm{b}}^2\left({\overline{\varepsilon}}^{\mathrm{p}}\right)}\\ {}\begin{array}{c}{F}_{\mathrm{r}}=\frac{2\bullet {r}_0\left({\overline{\varepsilon}}^{\mathrm{p}}\right)}{r_{90}\left({\overline{\varepsilon}}^{\mathrm{p}}\right)\bullet \left(1+{r}_0\left({\overline{\varepsilon}}^{\mathrm{p}}\right)\right)}\\ {}{H}_{\mathrm{r}}=\frac{2\bullet {r}_0\left({\overline{\varepsilon}}^{\mathrm{p}}\right)}{1+{r}_0\left({\overline{\varepsilon}}^{\mathrm{p}}\right)}\end{array}\end{array}}\kern1.25em {\displaystyle \begin{array}{c}{G}_{\upsigma}=1-\frac{\sigma_0^2\left({\overline{\varepsilon}}^{\mathrm{p}}\right)}{\sigma_{90}^2\left({\overline{\varepsilon}}^{\mathrm{p}}\right)}+\frac{\sigma_0^2\left({\overline{\varepsilon}}^{\mathrm{p}}\right)}{\sigma_{\mathrm{b}}^2\left({\overline{\varepsilon}}^{\mathrm{p}}\right)}\\ {}{N}_{\sigma }=\frac{4\bullet {\sigma}_0^2\left({\overline{\varepsilon}}^{\mathrm{p}}\right)}{\sigma_{45}^2\left({\overline{\varepsilon}}^{\mathrm{p}}\right)}-\frac{\sigma_0^2\left({\overline{\varepsilon}}^{\mathrm{p}}\right)}{\sigma_{\mathrm{b}}^2\left({\overline{\varepsilon}}^{\mathrm{p}}\right)}\\ {}\begin{array}{c}{G}_{\mathrm{r}}=\frac{2}{1+{r}_0\left({\overline{\varepsilon}}^{\mathrm{p}}\right)}\\ {}{N}_{\sigma }=\frac{2\bullet \left({r}_0\left({\overline{\varepsilon}}^{\mathrm{p}}\right)+{r}_{90}\left({\overline{\varepsilon}}^{\mathrm{p}}\right)\right)\bullet \left({r}_{45}\left({\overline{\varepsilon}}^{\mathrm{p}}\right)+0.5\right)}{r_{90}\left({\overline{\varepsilon}}^{\mathrm{p}}\right)\bullet \left(1+{r}_0\left({\overline{\varepsilon}}^{\mathrm{p}}\right)\right)}\end{array}\end{array}}$$

However, as shear tests were available, the final identification of *N*_*σ*_ was performed considering the results from the simple shear test from UA at 0° (final data set). As shown in Eqs. (71), the non-associated Hill48 model involves eight anisotropic parameters to be identified, assuming *L* and *M* parameters have the ideal isotropic values of three. When the evolving feature is considered in the enHill48 model, the identification of the four parameters for yield function ( *F*_*σ*_,  *G*_*σ*_,  *H*_*σ*_, *N*_*σ*_ ) would require stress-strain curves from uniaxial tensile tests along 0° and 90°, equibiaxial test, and simple shear test along 0°, while for the four parameters in the flow potential, the evolution curves of *r*-values from uniaxial tests along 0°, 45°, and 90° are needed. For all the stress-strain curves, the Voce equation (Eq. 4) is used, leading to 12 hardening parameters for four cases, while for the evolution curves of *r*-values, simple linear equations are used, leading to 6 parameters in total for three directions. Therefore, it could be stated that there are in total 18 hardening or evolution parameters to be identified for the enHill48 model. As the anisotropic parameters are explicitly correlated with these hardening or evolution parameters, no further calibration is needed after the simple fitting of the stress-strain curves and *r*-value evolution curves. Table 31 shows the calibrated anisotropic parameters for four discrete strain levels. A clear evolving trend of the parameters is evident, especially comparing the initial yielding with moderate strains.

**Table 31:** Example of evolving parameters for Hill48 non-associated model at various plastic strain levels

Plastic strain value	*F*_σ_	*H*_σ_	*G*_σ_	*N*_*σ*_	*F*_r_	*G*_r_	*H*_r_	*N*_*σ*_
0.002	0.61	0.41	0.59	2.12	0.71	0.65	0.35	1.24
0.1	0.61	0.41	0.59	2.21	0.86	0.66	0.34	1.12
0.18	0.58	0.44	0.56	2.03	0.84	0.65	0.35	1.13
0.4	0.54	0.48	0.52	1.60	0.79	0.64	0.36	1.17

**Fig. 66:**
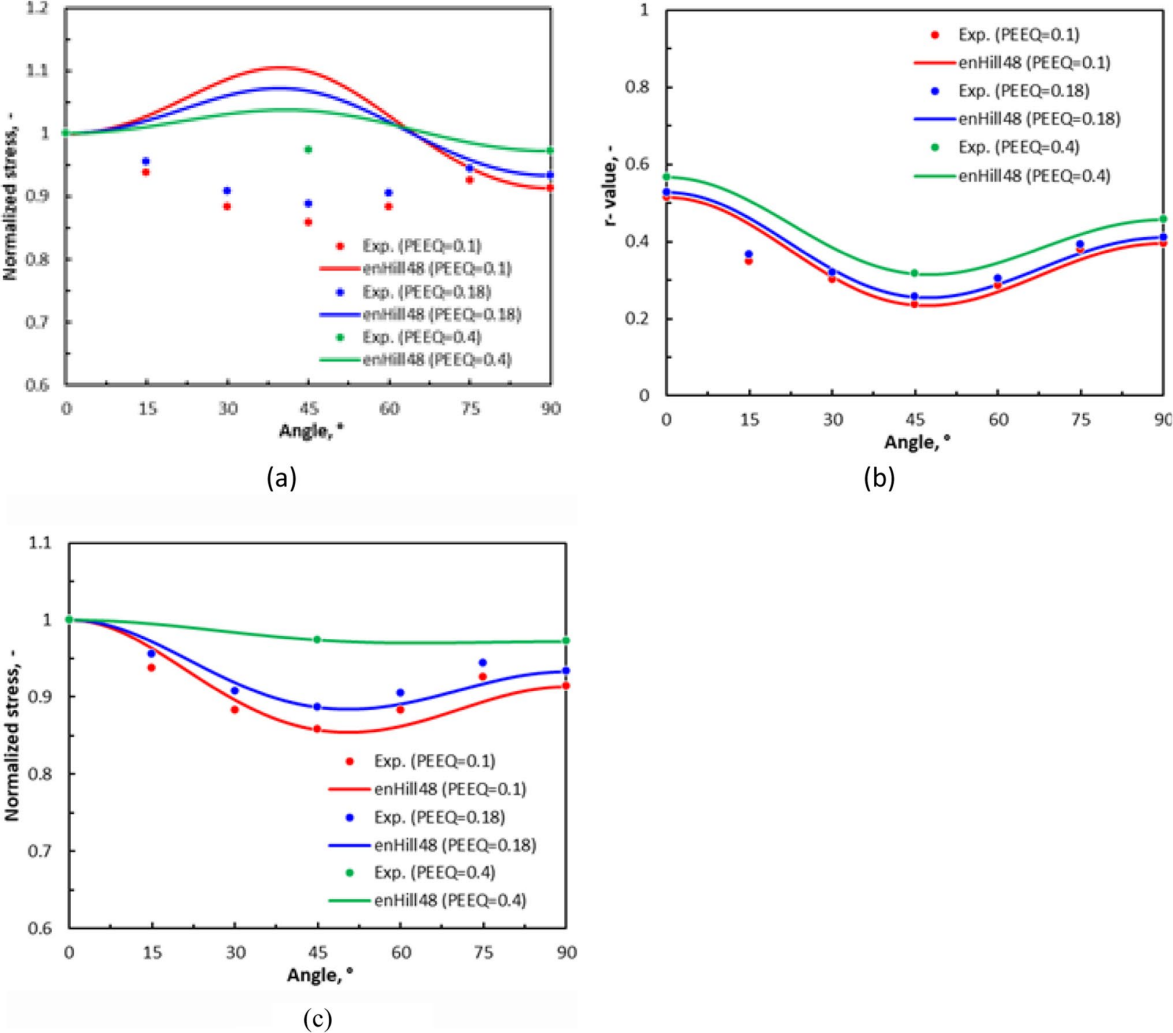
**a** Predicted anisotropy for the AA 6016-T4 alloy according to the evolving non-associated Hill48 yield locus (enHill48) for the final data set with *N*_*σ*_ based on shear test **a** uniaxial tensile flow stresses; **b** Lankford coefficients (r-values), **c** predicted uniaxial tensile flow for data set with *N*_*σ*_ based on Eq. 71, at equivalent plastic strain (PEEQ) of 0.10, 0.18, and 0.40; Symbols present the measured data of UA

**Fig. 67:**
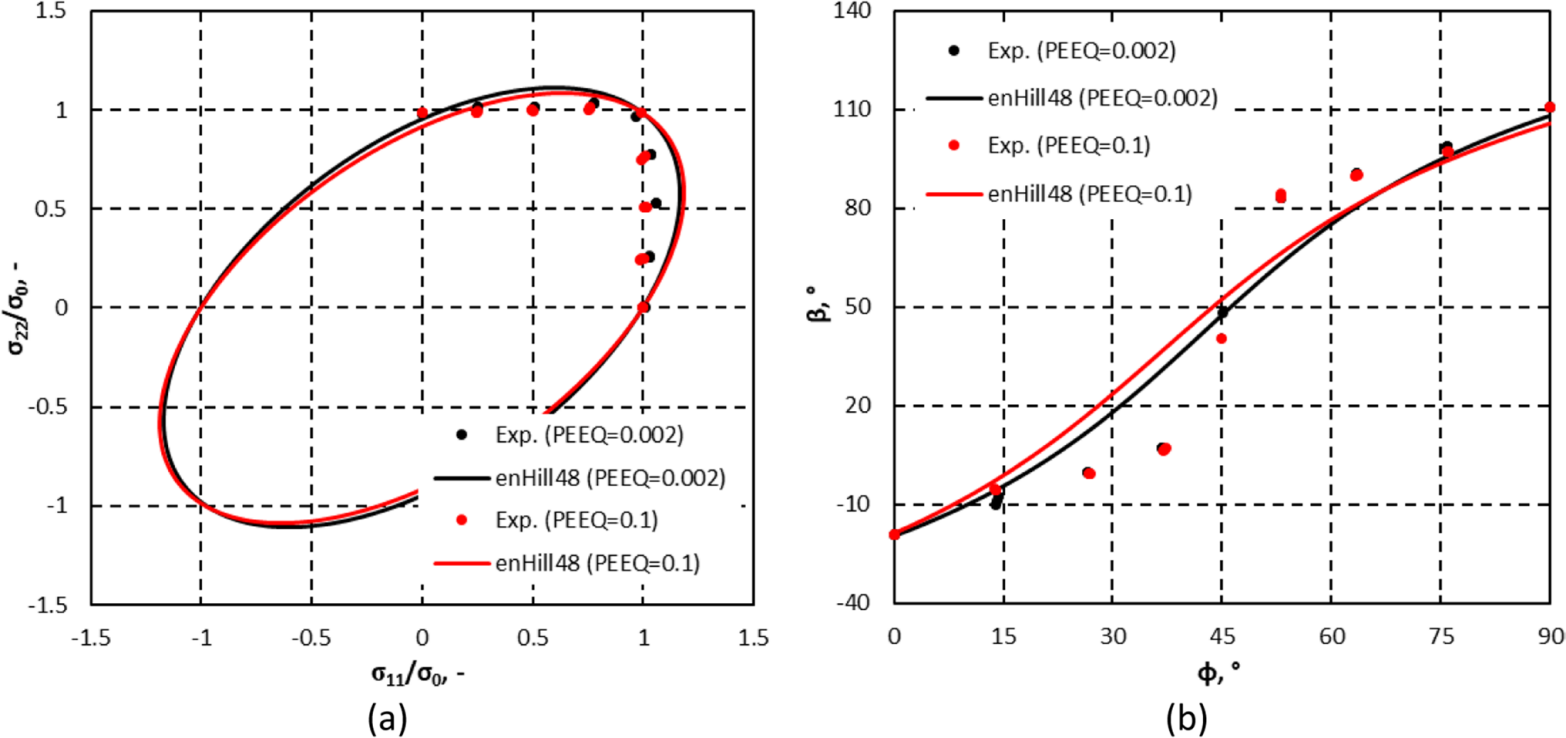
**a** Evolving non-associated Hill48 yield locus (enHill48) section in the biaxial plane *σ*_RD_ *σ*_TD_ at equivalent plastic strain (PEEQ) of 0.002 and 0.1 All stresses are normalized by the yield stress along the rolling direction, *σ*_*0*_, **b** Predicted direction of the plastic strain-rate β for biaxial loading at φ (see Fig. 41 for angle definition)

Figure 66 shows the comparison between the predicted and the experimental normalized yield stresses and *r*-values, for three values of plastic strain respectively for the final data set and the one based on Eqs. (71). The experimental results are the ones from UA. Figure 67 presents the yield surface section in the biaxial plane, only for two values of plastic strain, since the experimental data corresponds to the ones from TUAT. It is noted that due to the use of simple shear data, the model (with the final data set) does not capture the uniaxial tension stress directionality well especially at 45° (Fig. 66a) compared to the calculation of parameter *N*_*σ*_ in the initial enHill48 model (Fig. 66c). The model enables an accurate description of the Lankford coefficient, including its evolution. For large strains, the *r*-values evolve with a linearly increasing trend, which is similar to the results presented in Fig. 5(c). However, the model fails in capturing the shape of the yield locus and the directions of the plastic strain-rate β, for biaxial loadings (Fig. 67). In the cup drawing simulations, the parameters are continuously updated with the equivalent strain values. The current formulation significantly improves the model performance for simple shear anisotropy as well as for the cupping results (punch force and earing profile) and, consequently, the final data set will be the only one discussed in “Hill48 with non-associated flow rule” Section.

### Identification of hardening laws and data used

**Fig. 68:**
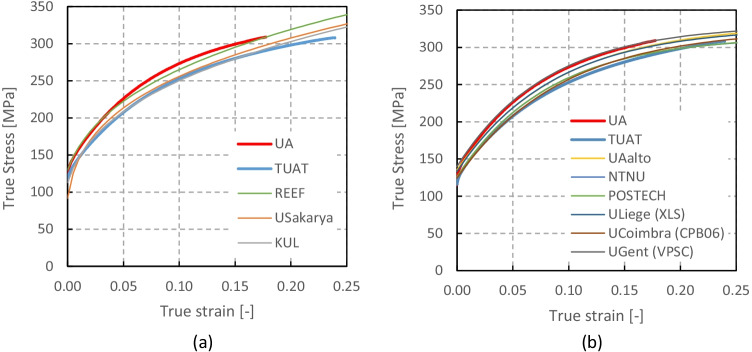
Experimental RD tensile curve of AA 6016-T4 of TUAT and UA along with the modelling results obtained with isotropic hardening laws: **a** Swift model and **b** Voce model

In this section, the identification procedures of isotropic hardening and combined isotropic – kinematic hardening parameters are briefly reminded. The benchmark organizer (REEF) provided the parameter values for the isotropic Swift and Voce hardening laws, identified using the UA tensile tests in RD and least-square fit (see Table 32). Other participants used different data and procedures. For instance, to determine the set of parameters of isotropic Swift law, USakarya approach was based on TUAT tensile tests in RD. Hardening curve parameters were obtained by using a curve fitting method from MATLAB Curve Fitting Tool software (Levenberg – Marquardt algorithm was selected). The values of the parameters used by all teams in the simulations presented in “Cup drawing simulations, analysis and discussion” Section are provided in Table 32. Figure 68 shows a comparison between modelling results and experimental RD tensile curves, designated as UA and TUAT.

**Table 32:** Values of hardening parameter data sets used by the participants, for phenomenological laws and if not presented in “Parameter identification, validation and/or use of crystal plasticity models” Section, for polycrystalline models

Participant & Selected law & Identification Method	Hardening law				
$Y\left({\overline{\varepsilon}}^p\right)={K}_0{\left({\varepsilon}_0+{\overline{\varepsilon}}^p\right)}^n$	**Swift Isotropic Hardening**				
	Set provided by Benchmark organizer (REEF)				
	*K*_*0*_ *(*MPa*)*	*ε*_0_		*n*	
Used by REEF, UCoimbra, UPorto, USiegen (LS-DYNA & PAMSTAMP)	498.8	0.0089		0.285	
	Own identified dataset				
USakarya (MSC.MARC, LS-DYNA) Matlab Levenberg Marquardt, RD Tokyo tests	474.1	0.002299		0.2703	
KUL (Tokyo test in RD – Fitted with Excel solver)	478.2	0.0068		0.2895	
$Y={Y}_0- Bexp\left(-n{\overline{\varepsilon}}^p\right)$	**Voce Isotropic Hardening**				
	Set provided by Benchmark organizer (REEF)				
	*Y*_0_(MPa)	B (MPa)		*n*	
	326.8	189.4		12.0	
	Own identified dataset				
UAalto	331.18	195.46		11.04	
NTNU	314.98	189.03		12.22	
POSTECH	315.21	188.97		12.17	
ULiege (XLS minimization)	326.99	193.60		11.64	
UCoimbra (CPB06)	324.7	200.23		10.83	
UGent (VPSC)	330.2	190.0		12.4	
USakarya (MSC.MARC, LS-DYNA) See formulae hereafter*	**Mixed hardening**				
	K_0(iso)_ (MPa)	n_(iso)_	ε_0(iso)_	C (MPa)	γ
	480	0.46	0.0089	15,000	166.66

$$\ast \upsigma ={\mathrm{K}}_{0\left(\mathrm{iso}\right)}{\left({\upvarepsilon}_{0\left(\mathrm{iso}\right)}+{\overline{\varepsilon}}_p\right)}^{{\mathrm{n}}_{\left(\mathrm{iso}\right)}}+\frac{\mathrm{C}}{\upgamma}\left(1-\exp \left(-\upgamma {\overline{\varepsilon}}^p\right)\right)$$

**Fig. 69:**
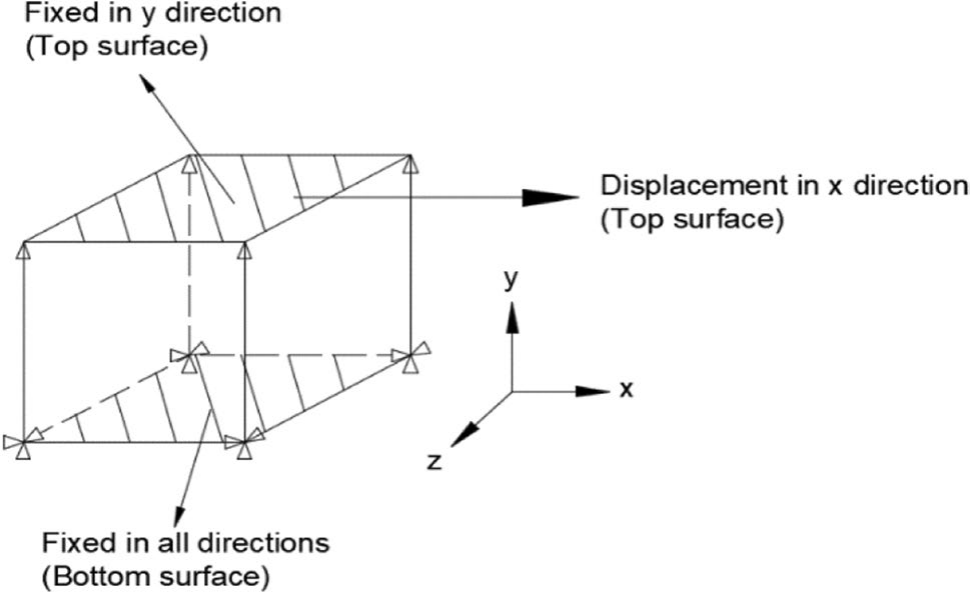
Reversal shear boundary conditions on a representative single element

**Fig. 70:**
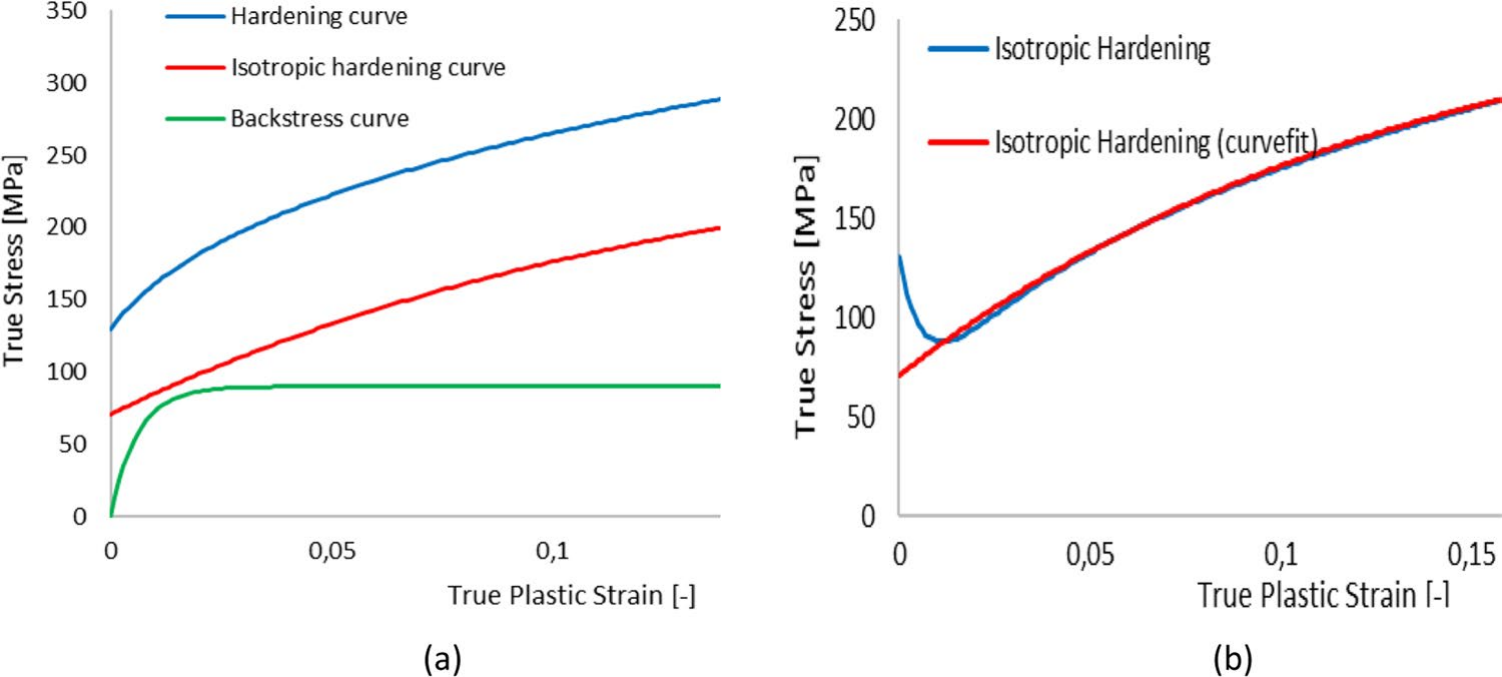
**a** Isotropic hardening, kinematic hardening and total hardening curves, **b** Curve fitting of isotropic part in combined hardening

Kinematic hardening parameters of a mixed hardening model was obtained by USakarya, considering the reversal shear test data in RD of UA. Simulations of single element tests under reversal shear conditions were carried out to identify the values in Table 32 by inverse modelling. Figure 69 shows the boundary conditions of reversal shear conditions on a representative single element. Figure 70 presents the kinematic and isotropic parts of the hardening model, separately. In the curve fitting procedure, a slight decreased of the isotropic part of the mixed hardening model is observed, within a small range of small true strain values. Nevertheless, the isotropic part is globally well captured. The developed Hypela2 user subroutine code of USakarya considers this situation.

### Discussion on the methodology for identification of the models

For polycrystalline models, we already pointed out the large differences in the number of grains considered in the final representative texture (FRT). Each team made its choice based on its experience and its focus, e.g. to model the tensile test behaviour (yield stress and Lankford predictions) or to take into account more stress states.

The results reported in “Parameter identification, validation and/or use of crystal plasticity models” Section for the polycrystalline approach demonstrate the care required in the representative crystal data set and that the verification and comparison with experiments (yield stress and Lankford values in different directions from RD) is quite important to guide the user.

Table 33 summarizes these details as well as the CPU time required for performing the identification steps and the numerical simulation of the uniaxial test with the polycrystalline model. Note that the manpower required to generate, from EBSD data and mechanical tests, the final set of polycrystal model parameters, considers a skilled user.

**Table 33:** Effort required to model the texture and to identify the data set for the polycrystalline models including hardening data. In the last column, referring to the tensile test with the polycrystal model, the number of Finite elements (FE) or the FFT grid size used is indicated in square brackets

Team	Target constitutive law and software used	Number of crystals in the FRT	Physical tests used for calibration	Manpower to generate the final set of polycrystal model parameters	CPU time & hardware (PC or HPC configuration for identification steps)	CPU time to model one tensile test
KUL	ALAMEL model, In-house code for virtual tests and Facet identification (FNG)	10,000	EBSD + tensile test along RD for hardening	~ 30 min	~ 10 s on HPC: Xeon Gold 6140 CPU@2.3 GHz – Skylake (7 s for generating discrete texture from EBSD data +3 s for 200 virtual tests in stress space)	_
REEF	Caz2018polycrys ABAQUS, MTEX	250	EBSD + tensile test along RD	~10 min	Total: 10 h for complete identification (i.e. including the FE inverse analysis stage which requires calculation of 7 *r*-values (see “Texture description for Caz2018polycrys” Section)	20 min on 1 PC [570 FE, see Fig. 34] (Intel Core i7–4770, 1 core, 16 GB RAM).
ULiege	Minty law, Lagamine, MTM MTM-FHM (1995)	1000	EBSD + tensile test along RD for hardening	~ 30 min	< 5 s	1.8 s [1 FE]
NTNU	3D RVE for FFT (DAMASK)	7509	EBSD + tensile test along RD	~ 30 min	120 min for the identification, PC (dual Intel Xeon E5–2696 v4, 88 core, 128GB RAM)	8 h on 1, PC (dual Intel Xeon E5–2696 v4, 88 core, 128GB RAM), [120X120X120] FFT grids
UGent	VPSC (own implementation) ABAQUS	1500	EBSD + tensile tests in 7 different directions	~ 30 min	< 30 min (each step, the seven simulations are run at least once for each fitting parameter)	~ 45 s to run 7 simulations (one per direction) in parallel (i7-8650U)

Table 34 details the number of tests used for the phenomenological yield locus identification. Note that the analytical expressions were used with the Hill48 yield criterion. Interestingly, most teams did not used all types of experimental data available. In particular, only two teams used the information from shear tests results, which can be related with the interpretation of the experimental results pointing kinematic hardening, as previously mentioned in “Monotonic and reverse simple shear tests” Section. In addition, only two teams took into account the direction of the plastic strain-rate in the identification procedure.

**Table 34:** Mechanical tests used for the phenomenological yield locus identification

Team	Yield criterion	Lankford coef. (RD, TD and DD)	Tensile flow stress (RD, TD and DD)	Tensile test in 15, 30, 60, 75 from RD	Equibiaxial test point	More biaxial tests	Shear tests	Optim. tool = O Analy. Form. =A
ULiege	Hill48(A)	X	X	X	–	–	–	O
UCoimbra	Hill48(A)	X	Only RD	–	–	–	–	A
UAalto	Hill48(NA)	X	X	–	X	–	X	A
USakarya	Yld89	X	X	–	–	–	–	O
POSTECH	Yld2000-2D	X	X	X	X	–	–	O
USakarya	HomPoI4	X	X	X	–	–	–	O
USakarya	HomPoI6	X	X	X	–	–	–	O
UCoimbra	CB2001	X	X	X	X	X	–	O
POSTECH	Yld2004–18p	X	X	X	X	–	–	O
NTNU	Yld2004–18p	X	X	X	X	–	–	O
UCoimbra	CPB06ex2	X	X	X	X	X	X	O
REEF	Caz2018-Orth	X	X	X	–	–	–	O

## Cup drawing simulations, analysis and discussion

The correlation between the predicted earing profile and *r*-values as well as the yield stresses directionalities has been shown in [13, 109]. In order to verify the implementation of the constitutive models, in commercial codes (PAMSTAMP, LS-DYNA) or done by the participants through user-subroutines (ABAQUS, DD3IMP, MSC.MARC, Lagamine), the participants were asked to perform uniaxial tensile tests for discrete values of the specimen orientation relatively to RD and report the estimated *r*-values and yield stresses. Therefore, the predicted *r*-values and yield stresses, obtained from FE simulations, as well as the earing profiles are reported in this section, for each yield function. The thickness distribution along the cup circumference at different heights was evaluated after unloading and it was an optional result, due to the work involved in its evaluation.

The result presentation is subdivided in four sections. The first and second ones concern simulations based on phenomenological models identified only by physical tests (“Results of phenomenological models identified with physical tests” Section) and also relying on virtual crystal plasticity computations (“Results of phenomenological models identified with physical and virtual crystal plasticity tests” Section). The next section (“Results with crystal plasticity based constitutive models” Section) analyses the results of crystal plasticity simulations. Finally “Summary of the results and discussion” Section presents a discussion of all these results.

### Results of phenomenological models identified with physical tests

#### 3-D orthotropic yield functions and solid or shell elements

##### Hill48 with associated flow rule

 Four teams reported results obtained using the Hill48 yield criterion and the Swift hardening law, with the set of parameters provided by the benchmark committee. These four teams are UCoimbra, UPorto, REEF and USiegen. As described in “Summary of features of FEM simulations” Section, UCoimbra used solid elements and the implicit code DD3IMP. UPorto also adopted solid elements and the simulations were performed with ABAQUS (explicit). REEF used ABAQUS (implicit) also with solid elements. Finally, USiegen used both solid and shell elements, with the explicit codes LS-DYNA (DYNA) and PAMSTAMP (PAM). In this section, the results obtained by ULiege using the Hill48 yield criterion (Set 2 of Table 22, in “Hill48 yield locus (associated law)” Section) and the Voce hardening law are also presented. According to the information given in “Summary of features of FEM simulations” Section, ULiege used solid elements and its own FE implicit software Lagamine.

As reported in Table 9 and Fig. 28, the finite elements adopted by USiegen have a small average in-plane size (nominal edge length of 0.5 mm). The velocity imposed to the punch in the explicit codes was 1 mm/ms; a constant friction coefficient of *μ* = 0.075 was applied to all tool surfaces in contact to the sheet metal blank. This value of the friction coefficient was adopted after performing a preliminary series of simulations using shell elements. Specifically, the friction coefficient value was taken such as to obtain the best fit for the first peak of the punch drawing force. UCoimbra considered a constant friction coefficient of *μ* = 0.020, while UPorto considered *μ* = 0.050 and REEF *μ* = 0.070. The simulations performed by ULiege considered a constant friction coefficient of *μ* = 0.082 applied to all tool surfaces in contact with the blank. ULiege used the same approach as USiegen to select the friction coefficient, by fitting the maximum punch force. The fact that ULiege attained a slightly higher value for the friction coefficient can be related with the different characteristics of the codes, but also with the adoption of the Voce hardening law, which tends to saturate for an equivalent plastic strain higher than 0.4. Note that the hardening presents an increasing trend when described with the Swift law (see Fig. 68).

**Fig. 71:**
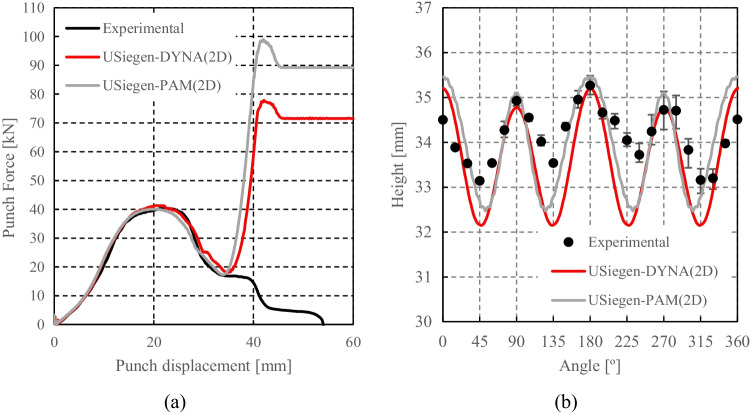
Comparison between experimental and predicted results by Hill48 criterion with the data set identified in “Hill48 yield locus (associated law)” Section (Table 22) with shell elements: **a** punch force-displacement, **b** earing profile

**Fig. 72:**
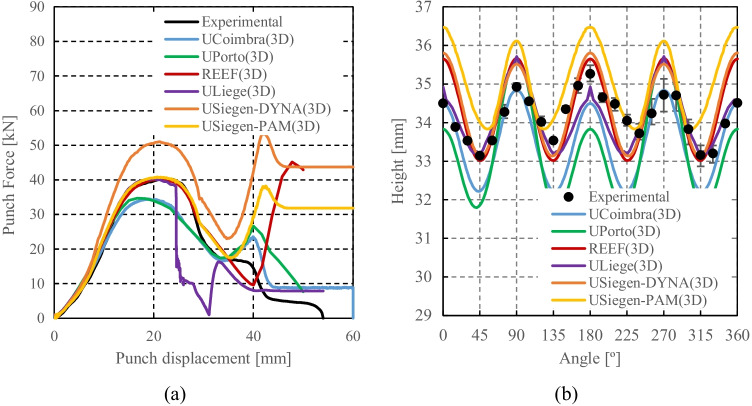
Comparison between experimental and predicted results by Hill48 criterion with the data set identified in “Hill48 yield locus (associated law)” Section (Table 22) with solid elements: **a** punch force-displacement, **b** earing profile

First, in Fig. 71 are reported the results obtained by USiegen with shell elements. Next, comparison between simulation results with solid elements, obtained with the explicit and implicit codes, are presented in Fig. 72. This enabled to compare the LS-DYNA and PAMSTAMP explicit code predictions obtained with the same process parameters, constitutive model, parametrization and FE meshes (for solid elements only (see Table 9)).

First, let us note that irrespective of the integration strategy (explicit vs. implicit) and irrespective of whether the simulations were conducted with shell or solid elements, Hill48 in conjunction with an isotropic hardening law correctly describes the overall experimental trends for both force vs. displacement and earing profile, i.e. 4 ears as observed experimentally.

**Fig. 73:**
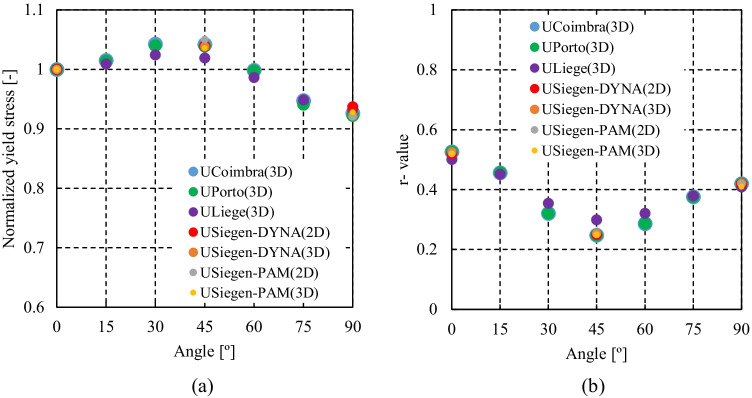
Predicted FE results by Hill48 criterion with the data set identified in “Hill48 yield locus (associated law)” Section (Table 22): **a** uniaxial tensile flow stresses, **b** Lankford coefficients (*r*-values)

For the first phase of the drawing operation (0 < displacement <30 mm), the results obtained with shell elements are very close to the experimental ones. The results from LS-DYNA with solid elements show a larger error than the one with shell elements, although the *r*-value and yield stress anisotropies are identical, as shown in Fig. 73. USiegen performed the simulations with a different type of integration scheme (elform = −1), in order to exclude the possibility that the element formulation might lead to this error. However, negligible differences were observed in the results. Because UCoimbra and UPorto used a smaller value for the friction coefficient, overall they predict a lower punch force.

Note that in the second phase of the drawing operation, where ironing of the initial flange area of the blank occurs in the drawing gap (displacement >30 mm), a secondary peak is observed in all the simulations, while the experimental curve exhibits a brief plateau and subsequently drops towards a constant, very low force. These ironing forces are significantly lower when using solid element than shell element, but are quite sensitive to the value of the friction coefficient (*μ* range [0.02,0.082]). The evolution of the predicted punch force shows its sensitivity to the contact algorithm, FE strategy (implicit, explicit), particularly for the ironing stage. For instance, the results with DD3IMP (UCoimbra, implicit, *μ* = 0.02) are close to the ones predicted by ABAQUS (UPorto, explicit, *μ* = 0.05). While for the same element (C3D8R) and software (ABAQUS), but implicit or explicit strategy and different contact algorithms and values of the friction coefficient (*μ* = 0.05 or 0.07), the ironing peak computed by REEF is twice the one computed by UPorto. The single effect of a low or high friction coefficient value will be discussed in Fig. 75.

Figure 72(a) shows that the evolution for the punch force predicted by Lagamine (ULiege) presents a sudden drop. This event occurs for a punch displacement for which the blank progressively starts to lose contact with blank holder. The control of the downward movement of the blank holder does not seem to fulfil the gap imposed by the stopper. This leads to issues in the punch force prediction and to the pinching of the ears located at 0° and 90° with the RD, as shown in Fig. 72(b).

It is worth noting that the predicted amplitude of the ears obtained with shell elements is approximately 1 mm larger than the experimental one. In contrast, the amplitudes of the predictions obtained with solid elements are more accurate. The only exception is the profile obtained by UPorto, which can be related with the fact that the blank holder was controlled by a fixed position, leading to a much lower restraining force. In fact, the trend obtained by UPorto is identical to the one of DD3IMP at TD, but shows a lower height at RD. This observation is certainly related with the fact that UPorto considered a constant gap between the blank holder and the die, which reduces the friction effect induced by the blank holder, but it can also produce some wrinkles that influence the trend for the earing profile. Overall, the earing profile is being dictated by the in-plane distribution of the normalized yield stress and *r*-values, which are similar for all models, as shown in Fig. 73.

**Fig. 74:**
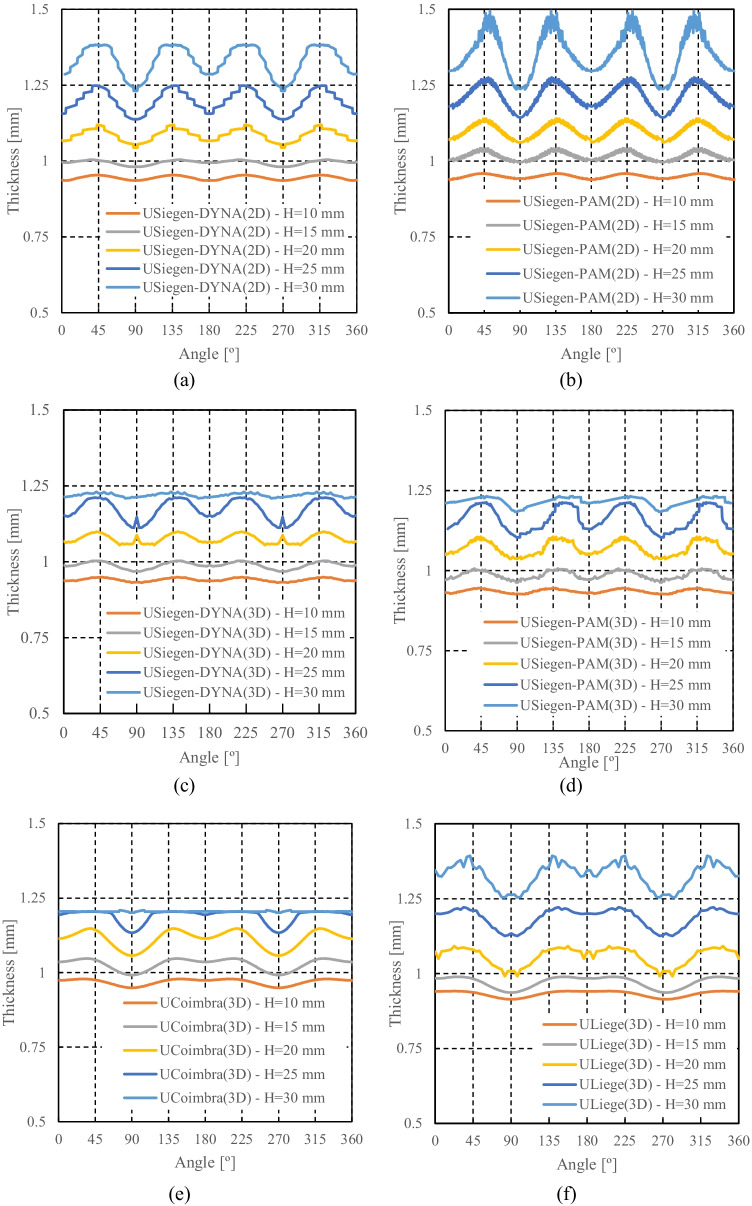
Evolution of the thickness along the cup circumference at different heights H = 15, 20, 25 and 30 mm from the cup bottom, by Hill48 criterion with the data set identified in “Hill48 yield locus (associated law)” Section (Table 22): **a** LS-DYNA with shell elements, **b** PAM-STAMP with shell elements, **c** LS-DYNA with solid elements, **d** PAM-STAMP with solid elements, **e** DD3IMP with solid elements, and **f** Lagamine with solid elements

The better prediction of the earing amplitude by solid element simulations can be explained by the fact that these elements can deform over thickness, within the drawing gap during ironing. The resulting transversal strain leads to an overall higher cup wall. Obviously, transverse strains are neglected when using shell elements. This fact also explains the lack of accuracy of the shell elements predictions for the thickness distribution along the cup circumference, for different heights, presented in Fig. 74. For example, at a height of 30 mm, the predicted thickness value obtained with shell elements is higher than the gap between the punch and the die. On the other hand, for the simulations with solid elements the predictions of the thickness values for the same height are closer to the gap value, particularly the one obtained with DD3IMP. This indicates the stricter fulfilment of the normal contact constraint in the algorithm adopted in this code. Overall, the results obtained by DD3IMP present slightly higher thickness values for the lower heights of the cup, which can be related with the lower average cup height, i.e. with the fact that a lower friction coefficient was considered. The only exception for results obtained with solid elements is the one from Lagamine (ULiege), for which the thickness distribution predicted shows values higher than the gap between the punch and the die. Once again, this indicates either that the contact algorithm is not imposing the fulfilment of the gap or that the solid elements adopted are leading to an improper prediction of the transverse strains. The height predicted for RD is quite similar to the one obtained with DD3IMP, while at TD it is closer to the one obtained by UPorto and USiegen LS-DYNA. These results indicate that the earing trend is affected by the contact with friction algorithms, including the ones adopted to control the blank holder and by the formulation adopted for the solid element.

**Fig. 75:**
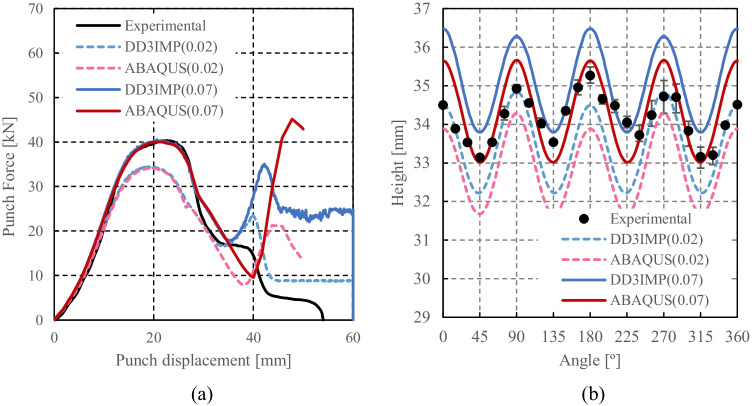
Comparison between experimental and predicted results by Hill48 criterion obtained with DD3IMP and built-in ABAQUS for the data set (Table 22 line1), solid elements and two different values of friction coefficient (0.02 and 0.07): **a** punch force-displacement, **b** earing profile

**Fig. 76:**
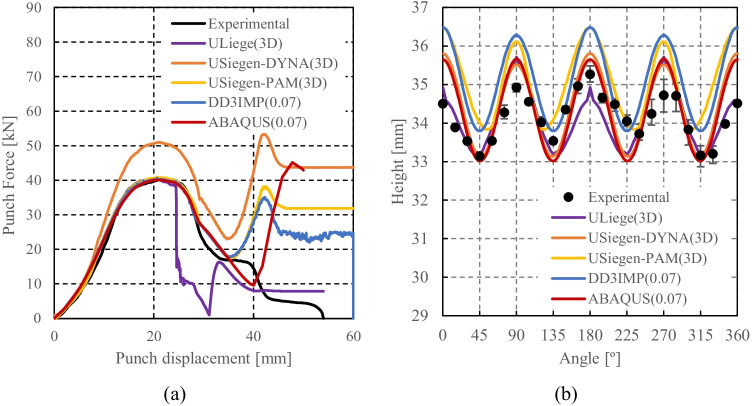
Comparison between experimental and predicted results by Hill48 criterion with the data set identified in “Hill48 yield locus (associated law)” Section (Table 22) with solid elements and three similar values of friction coefficient (0.07, 0.075 USiegen and 0.082 ULiege): (**a**) punch force-displacement, (**b**) earing profile

The influence of the friction on the results is depicted in Fig. 75, which shows the results for two friction coefficients obtained with two implicit codes: DD3IMP (UCoimbra) and ABAQUS (REEF), using solid elements. The value for the friction coefficient of 0.07 enables capturing the maximum drawing force, while the ironing force is clearly overestimated. Regarding the earing, the increase of the friction coefficient from 0.02 to 0.07 leads to an increase of the average height, as previously reported in “Discussion about friction coefficient” Section for the von Mises yield criterion. The increase of the friction coefficient also induces a slight increase on the earing amplitude. For instance, for the results obtained by REEF, the amplitude increases from 2.62 mm to 2.65 mm (against the experimental value of 2.29 mm). This is also associated with the change in the location of the global maximum, from 90° (TD) to 0° (RD). This small effect is more pronounced in the results obtained by DD3IMP. This fact indicates that the earing trend is influenced by the contact algorithm adopted, since this effect is related with the uneven distribution of the blank holder force, resulting from the material orthotropic behaviour and the contact with friction conditions. The influence of the formulation adopted can also be observed in Fig. 76, which shows the results obtained by different codes (Lagamine, LS-DYNA, ABAQUS, DD3IMP, PAMSTAMP), using similar values for the friction coefficient (μ = 0.07 to 0.082) and solid elements. As shown in Fig. 76(a), this high value for the friction coefficient enables to capture the maximum drawing force, except for LS-DYNA. Note the excellent correlation between the earing profiles predicted by ABAQUS (implicit, REEF) and LS-DYNA (USiegen) and between DD3IMP (UCoimbra) and PAMSTAMP (USiegen). Thus, Fig. 76 highlights the small dispersion in the average height and amplitude of the earing profiles predicted by different codes, when using solid elements and the Hill48 yield criterion.

**Fig. 77:**
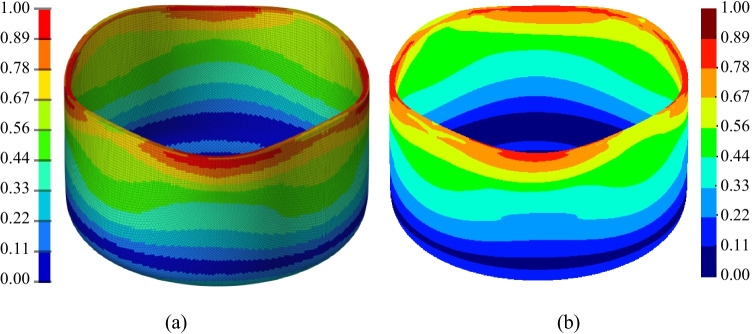
Distribution of the equivalent plastic strain predicted by Hill48 criterion for the data set (Table 22 line 1): **a** LS-DYNA with solid elements; **b** DD3IMP with solid elements

Figure 77 shows the isocontours of the equivalent plastic strain obtained in the simulations conducted with LS-DYNA and DD3IMP, with solid elements. Note that both codes can successfully predict/account for the high values of the plastic strains reached on the cup wall, due to the bending/unbending around the die radius, but also because of the ironing.

##### Hill48 with non-associated flow rule

As described in “Main features of constitutive laws” Section, the use of a non-associated flow rule with a potential of the same mathematical form as the Hill48 yield criterion allows increasing the number of anisotropy parameters in the elastoplastic model.

Three teams, USiegen, UPorto and UAalto used a non-associated flow rule in conjunction with the Hill48 yield criterion. While USiegen and UPorto used constant anisotropy properties (initial yield stresses and Lankford coefficients) in Eqs. (71) to compute the input material data set, UAalto considered that both the parameters related with the plastic potential and the yield function evolve as a function of the equivalent anisotropic plastic strain (see “Hill48 and non-associated flow rule” Section). They also considered a different isotropic hardening law. Namely, USiegen and UPorto considered the Swift law (REEF parameters in Table 32), while UAalto adopted the Voce law (UAalto parameters in Table 32). USiegen adopted shell elements and the explicit code PAMSTAMP, while UAalto and UPorto used solid elements, with the implicit and explicit versions of ABAQUS, respectively. Moreover, the values adopted for the friction coefficient were *μ* = 0.075, *μ* = 0.05 and *μ* = 0.01, for USiegen, UPorto and UAalto, respectively.

**Fig. 78:**
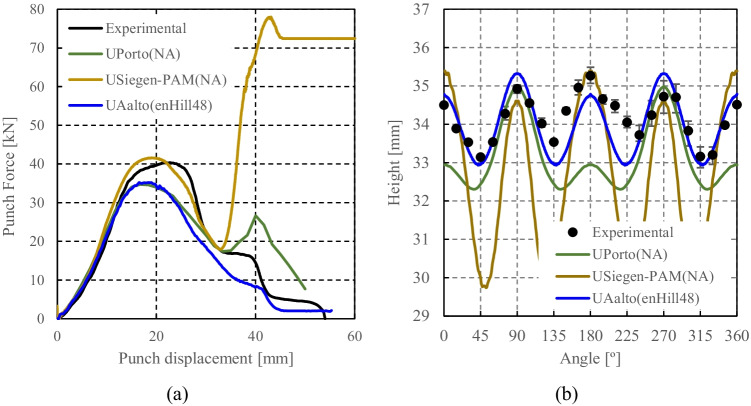
Comparison between experimental and predicted results by (NA) Hill48 criterion with constant or evolving anisotropy parameters identified in “Hill48 and non-associated flow rule” Section (Eqs. 71): **a** punch force-displacement, **b** earing profile

The predicted punch force evolution with its displacement and the earing profiles are compared to the experimental ones in Fig. 78(a). Regarding the punch force evolution, the differences between the predictions during the drawing phase reflect the use of a higher friction by USiegen. The differences between the predictions for the ironing stage are mainly related to the fact that USiegen used shell elements while UAalto and UPorto adopted solid elements (see also the discussion in “Hill48 with associated flow rule” Section on the errors associated with performing simulations with shell elements and thus neglecting the thickness strains). Note that UAalto and UPorto adopted the same type of solid element and the same contact algorithm, which may allow some penetration (see Table 9 and Table 10). Interestingly, the numerical simulation performed by UAalto presents a lower ironing force, which is even slightly lower than the experimental one. This can be related with the very low friction coefficient (*μ* = 0.01), considering the very high contact pressure, which prevails during this stage of the cup drawing process. It can also be attributed to the use of the Voce type law, which saturates for an equivalent plastic strain higher than 0.40 (see Fig. 68 for Voce and Swift stress-strain curves and the equivalent strain distribution, reaching values as high as 1.0 in Fig. 77). However, in this particular case, the fact that the *r*-values present an increasing trend with the increase of the equivalent plastic strain (see “Hill48 and non-associated flow rule” Section) also modifies the plastic flow. It justifies a lower increase of the thickness in the outer edge of the vertical wall, similar to what is observed when adopting the von Mises yield criterion (see also Fig. 27 in “Discussion about friction coefficient” Section).

**Fig. 79:**
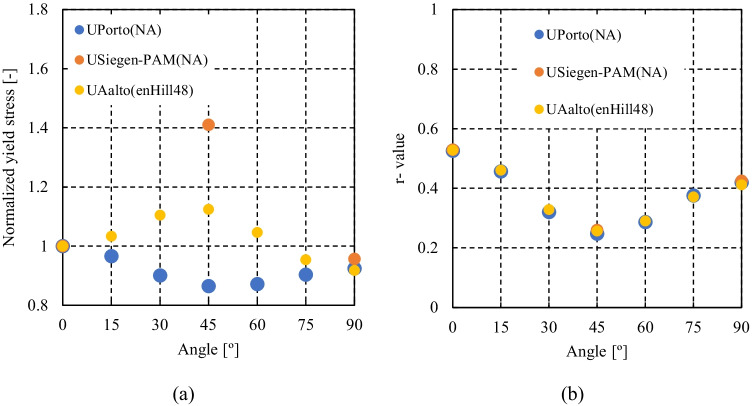
Predicted FE results by (non-associated flow rule) Hill48 criterion with constant or evolving anisotropy parameters identified in “Hill48 and non-associated flow rule” Section (Eqs. 71): **a** uniaxial tensile flow stresses, **b** Lankford coefficients (*r*-values). Note that for UAalto (enHill48) the results are plotted only for an equivalent plastic strain of 0.18

Regarding the prediction of the earing phenomenon, the results plotted in Fig. 78(b) show that the use of non-associated plasticity, as implemented in PAMSTAMP, leads to a strong overestimation of the earing amplitude. The material at 45° with RD clearly shows a large resistance against plastic deformation, which correlates with the high value predicted for the normalized yield stress along this direction, as shown in Fig. 79(a). Although the experimental stresses are used as input for the model, they are not properly described by the approach adopted in PAMSTAMP Shell case.

On the other hand, as shown in Fig. 79, the solid element simulations with constant anisotropic parameters used by UPorto enable a proper description of both the normalized yield stresses and *r*-values in-plane distribution (see experimental reference in Fig. 5(a) and (b)). UPorto results predict an earing amplitude closer to the experimental one, when compared with the one obtained by USiegen with PAMSTAMP, since the amplitude of the in-plane distribution of the normalized yield stress is lower. A high difference in the height predicted for RD and TD is observed in UPorto results, when compared with the one this team obtained with the associated flow rule (see Fig. 72(b)). Finally, the approach adopted for the implementation of the non-associated flow rule by UPorto leads to a worse prediction of the earing profile than the one using Hill48 with an associated flow rule. Note that the difference between the height predicted along RD and TD is always higher for the simulations performed by UPorto, which can be related with the fixed gap between the die and the blank holder.

The UAalto simulation with solid element and evolving anisotropic parameters within the simulation (see Table 31) is able to provide a result closely related to the experimental profile.

**Fig. 80:**
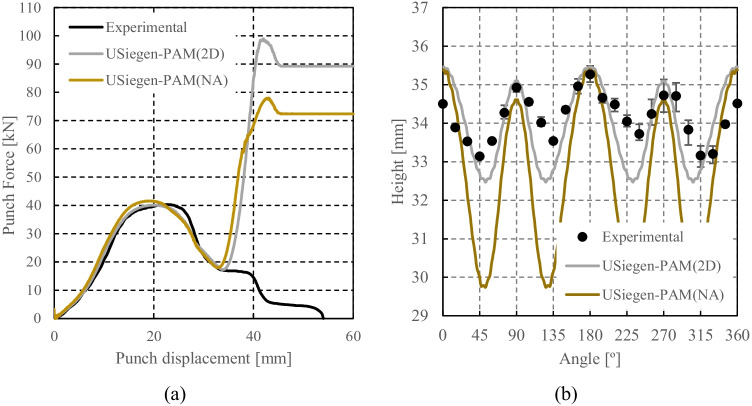
Comparison between experimental and predicted results with shell elements and Hill48 criterion with associated flow rule (data set Table 22-line 1) and non-associated flow rule (data set computed by Eqs. (71) for constant anisotropy properties (see Tables 4 and 5)): **a** punch force-displacement, **b** earing profile

The results from the simulation performed with the associated flow rule by USiegen, using PAMSTAMP and shell elements are compared with the ones obtained with the non-associated flow rule in Fig. 80. Both models enable a proper description of the in-plane distribution of the *r*-values (see Figs. 73 and 79). However, unlike what was expected, for the non-associated flow rule the in-plane distribution of the normalized yield stress presents a higher amplitude then the one obtained with the associated flow rule (see Fig. 73 and 79). During the first part of the drawing stage, the force predicted with the non-associated flow rule is slightly higher, which correlates with the higher value predicted for the normalized yield stresses (see Fig. 80(a)). The higher amplitude observed for the in-plane distribution of the normalized yield stress also justifies the increase in the earing amplitude observed in Fig. 80(b).

##### CB2001 yield criterion

In the following, CB2001 or the Cazacu [12] orthotropic yield criterion (Eq. 15) was applied to model the cup drawing process, with the anisotropy coefficients identified in “CB2001 by Cazacu and Barlat (2001)” Section (Table 26) and the Swift hardening law in “Identification of hardening laws and data used” Section (Table 32). UCoimbra performed the numerical simulation with DD3IMP code, using solid elements and a value of *μ* = 0.020 for the friction coefficient.

**Fig. 81:**
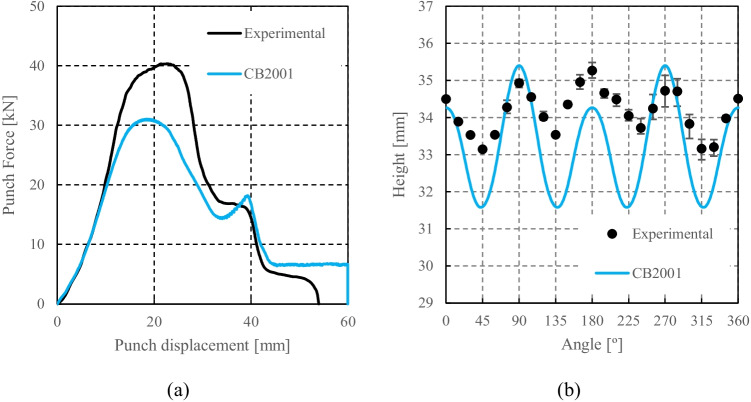
Comparison between experimental and predicted results by CB2001 with the anisotropy coefficients identified in “CB2001 by Cazacu and Barlat (2001)” Section (Table 26): (**a**) punch force-displacement, (**b**) earing profile

**Fig. 82:**
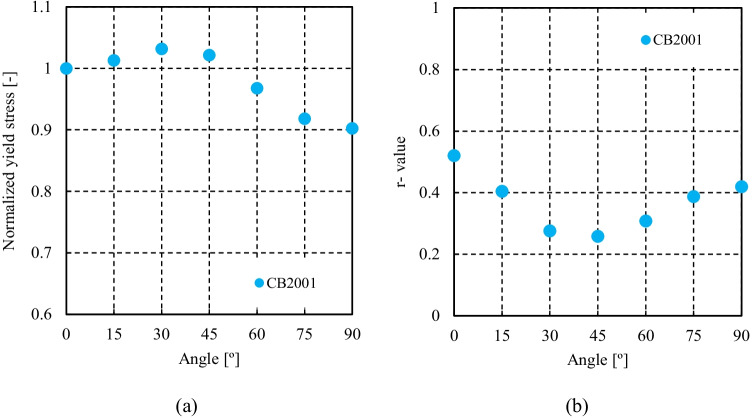
Predicted FE results by CB2001 criterion with the anisotropy coefficients identified in “CB2001 by Cazacu and Barlat (2001)” Section (Table 26): **a** uniaxial tensile flow stresses, **b** Lankford coefficients (*r*-values)

Figure 81(a) compares the predicted forming force vs. the punch stroke with the experimental data, while the earing profile prediction is plotted in Fig. 81(b). It shows four ears, as observed in the experimental tests. The earing profile is similar to the one obtained with Hill48(A) (see Fig. 72). CB2001 predicts an average height of *h*_*avg*_ = 33.14 mm against *h*_*avg*_ = 34.10 mm in experiments (2.8% difference) and a cup profile amplitude of 3.82 mm, against 2.29 mm experimental value (67% difference) and 2.62 mm obtained with Hill48(A), using the same friction coefficient and code with the data set of Table 22-line 1. The slightly higher amplitude of Hill48 results may be correlated with the fact that CB2001 predicts a slighter more pronounced anisotropy in the yield stresses than Hill48 (see Figs. 73(a) and 82(a)).

**Fig. 83:**
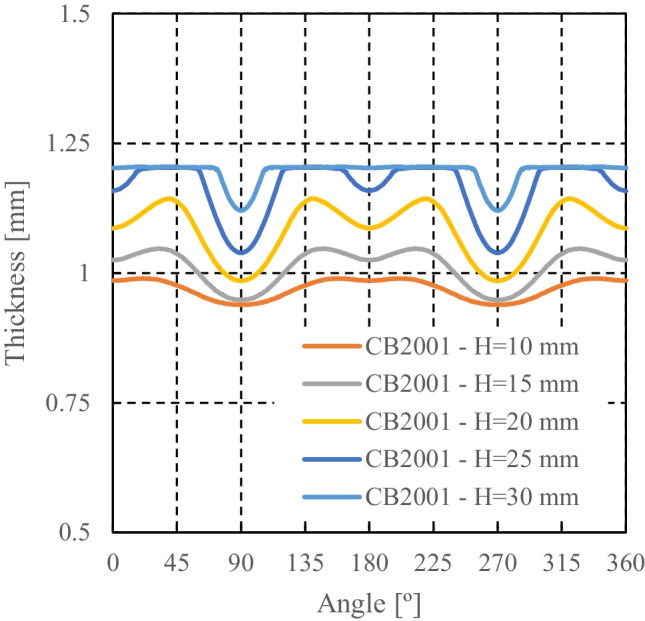
Evolution of the thickness along the cup circumference at different heights H = 15, 20, 25 and 30 mm from the cup bottom, by CB2001 with the anisotropy coefficients identified in “CB2001 by Cazacu and Barlat (2001)” Section (Table 26)

Figure 83 shows the evolution of the wall thickness along the cup circumference (taken at different cup height from the cup bottom). The comparison with the results obtained with the Hill48 yield criterion (see Fig. 74(e)), indicates that the CB2001 leads to a slightly sharper variation along the circumferential direction. For example, for H = 20 mm, the thickness variation is 0.09 mm for Hill48 while for CB2001, it is 0.16 mm.

##### Yld2004–18p yield criterion

Two teams contributed with FE simulations for the Yld2004–18p yield criterion using POSTECH data set of anisotropy coefficients (Table 28-line 1) and solid elements. POSTECH used their own implementation in ABAQUS explicit solver (VUMAT), Voce hardening law (see Table 32) and *μ* = 0.100, while USiegen used the built-in implementation of Yld2004–18p in LS-DYNA (explicit), the Swift hardening law (see Table 32 data set of REEF), and *μ* = 0.075.

NTNU performed an identification of the parameters for the Yld2004–18p yield criterion based on the experimental data provided by TUAT (see “Yld2000-2D and Yld 2004-18p” Section and Table 28, line 2). This team performed simulations using their own implementation of Yld2004–18p in ABAQUS implicit solver (UMAT), considering the same type of solid elements as POSTECH, a Voce hardening law (see Table 32) and *μ* = 0.090. USiegen performed simulations using the same FE model for both data sets, the one of POSTECH and the one of NTNU.

**Fig. 84:**
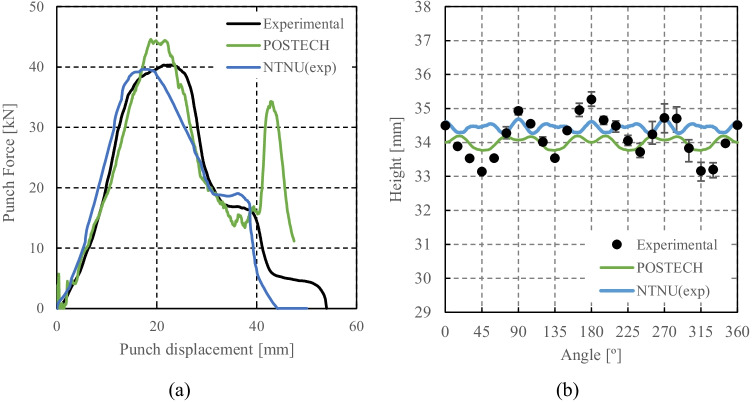
Comparison between experimental and predicted results by Yld2004–18p criterion with data set of POSTECH, using ABAQUS explicit (POSTECH VUMAT) and with data set of NTNU for their own UMAT implementation in ABAQUS (see “Yld2000-2D and Yld 2004-18p” Section (Table 28)): (**a**) punch force-displacement, (**b**) earing profile

The results with the FE code ABAQUS obtained for POSTECH and NTNU data sets and C3D8R solid elements are shown in Fig. 84. The punch force evolution is well captured, although it is overestimated by POSTECH results, for both the drawing and the ironing stage (see Fig. 84(a)). This can be related with the higher friction coefficient value but also with the coarse discretization adopted, when compared with the one used by UAalto using also an explicit strategy and the same solid element (see Table 9). Figure 84(b) shows that the cup drawing profiles obtained by POSTECH and NTNU, using their own implementations of Yld2004–18p in ABAQUS. Each team predicts 8 ears but their minimum and maximum locations in the cup height are opposite. However, the POSTECH and NTNU predictions for *r*-values and yield stresses are practically identical (see “RVE Damask Simulations” and “Yld2000-2D and Yld 2004-18p” Sections), using their respective sets of parameters for Yld2004–18p. Note that with both sets of anisotropy coefficients, the predicted earing amplitudes are similar and much smaller than the experimental one.

**Fig. 85:**
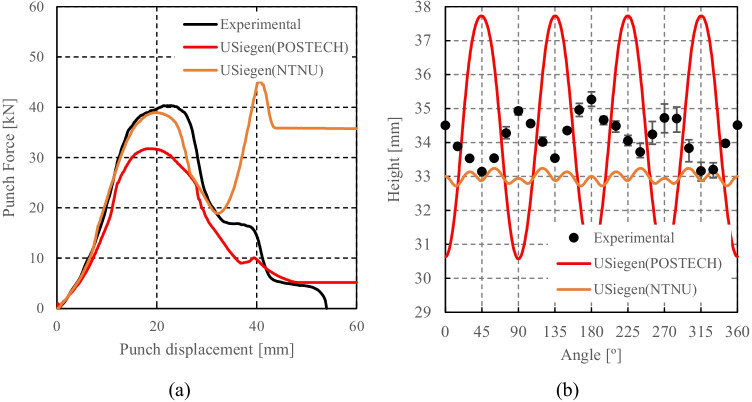
Comparison between experimental and predicted results by Yld2004–18p criterion with the anisotropy coefficients provided by POSTECH and NTNU (see “Yld2000-2D and Yld 2004-18p” Section (Table 28)), using LS-DYNA (built-in; USiegen): **a** punch force-displacement, **b** earing profile

**Fig. 86:**
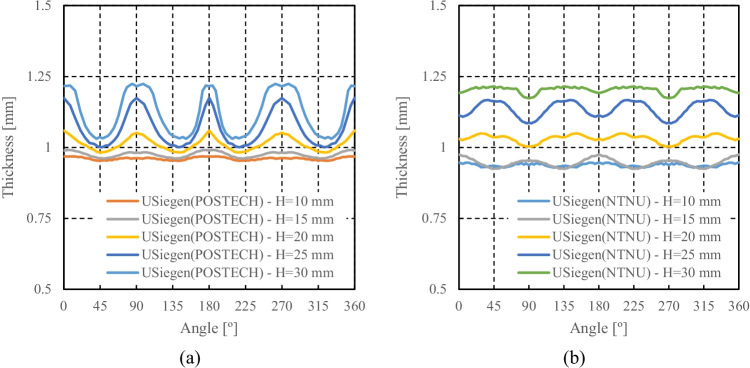
Evolution of the thickness along the cup circumference at different heights H = 15, 20, 25 and 30 mm from the cup bottom, using LS-DYNA (built-in; USiegen) and the set of anisotropy coefficients provided by: **a** POSTECH and **b** NTNU (see “Yld2000-2D and Yld 2004-18p” Section (Table 28))

In Fig. 85, the results obtained by USiegen, with the built-in Yld2004–18p in FE code LS-DYNA, for POSTECH and NTNU data sets of parameters are shown. Figure 85(a) shows that with POSTECH anisotropy coefficients and a friction coefficient slightly lower (USiegen μ = 0.075, POSTECH μ = 0.10), both the drawing and the ironing force are underestimated. On the other hand, for the set of anisotropy parameters identified by NTNU, the predicted trend during the drawing stage is the same irrespective of the code used (LS-DYNA or ABAQUS). The trend for the ironing stage is similar to the one observed with Hill48(A) (see Fig. 72(a)), which can be related with the element type. Note also that the predicted trends for the earing profile obtained with the model built-in LS-DYNA are consistent with the predicted thickness distributions in the cup wall, as shown in Fig. 86. The fact that the set of parameters provided by POSTECH leads to lower average values for the thickening of the cup wall can also help explaining the differences observed in the evolution of the punch force (see Fig. 85(a)).

Note that with POSTECH anisotropy coefficients, the FE cup drawing results obtained with LS-DYNA show four ears, as in the experiments but the amplitude is strongly overestimated (see Fig. 85(b)). For the same set of anisotropy coefficients, the number of ears is different and the maxima and minima in cup height are at opposite location with respect to the results reported by POSTECH (see Fig. 84(b)).

**Fig. 87:**
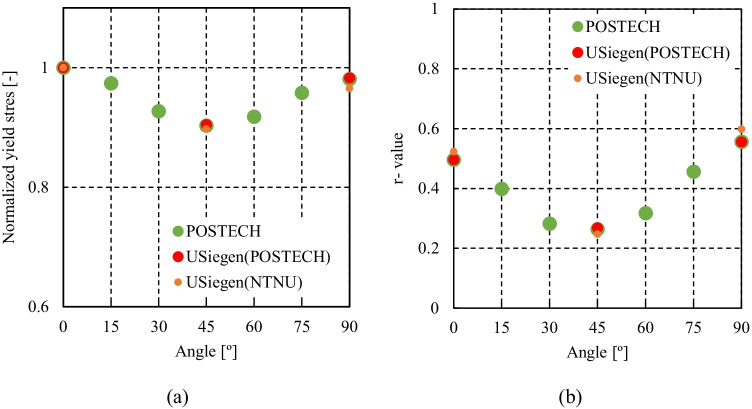
FE predictions of the anisotropy for uniaxial loadings according to Yld2004–18p criterion for the anisotropy coefficients, provided by POSTECH and NTNU (see “Yld2000-2D and Yld 2004-18p” Section (Table 28)), using LS-DYNA (USiegen) and ABAQUS (POSTECH): **a** uniaxial tensile flow stresses, **b** Lankford coefficients (*r*-values)

For the NTNU anisotropy coefficients, the LS-DYNA profile shows 12 ears and the amplitude is underestimated. The comparison with the results obtained by NTNU, for the same data set, shows that although the number of ears is different (12 vs. 8), the amplitude and the location of the global maxima and minima are similar, but contrary to the experimental observations. However, for both sets of anisotropy coefficients for Yld2004–18p, the predictions for the anisotropy in yield stresses and *r*-values obtained with the LS-DYNA code are the same (see Fig. 87) and are close to the experiments (Fig. 5).

The analysis of the results obtained with other yield criteria with different FE models/codes, including different types of elements and contact algorithms, indicates that these parameters mainly affect the average height and the ears amplitude, but have no effect on the number of ears or on the location of the ears, which are driven by the yield locus. Therefore, the Yld2004–18p criterion seems to be very sensitive to the formulations adopted in its implementation.

##### CPB06ex2 yield criterion

The results presented in this section were obtained with DD3IMP (implicit) code, using the CPB06ex2 yield criterion (anisotropic parameters Table 29) and the Voce hardening law (Table 32, UCoimbra). In this case, a constant friction coefficient *μ* = 0.1 was used (see Table 10).

**Fig. 88:**
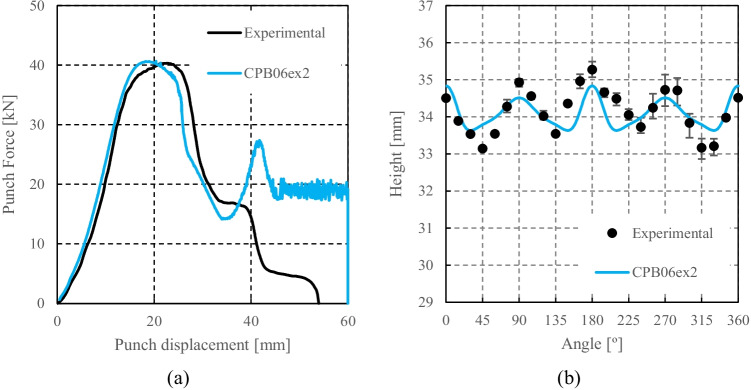
Comparison between experimental and predicted results by CPB06ex2 criterion with the anisotropy coefficients identified in “CPB06 criterion by Cazacu, Plunket and Barlat (2006)” Section (Table 29): **a** punch force-displacement, **b** earing profile

**Fig. 89:**
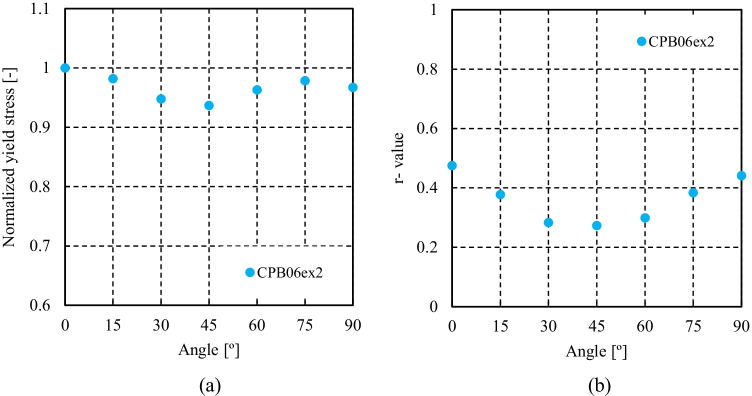
Predicted FE results by CPB06ex2 criterion with the anisotropy coefficients identified in “CPB06 criterion by Cazacu, Plunket and Barlat (2006)” Section (Table 29): **a** uniaxial tensile flow stresses, **b** Lankford coefficients (*r*-values)

**Fig. 90:**
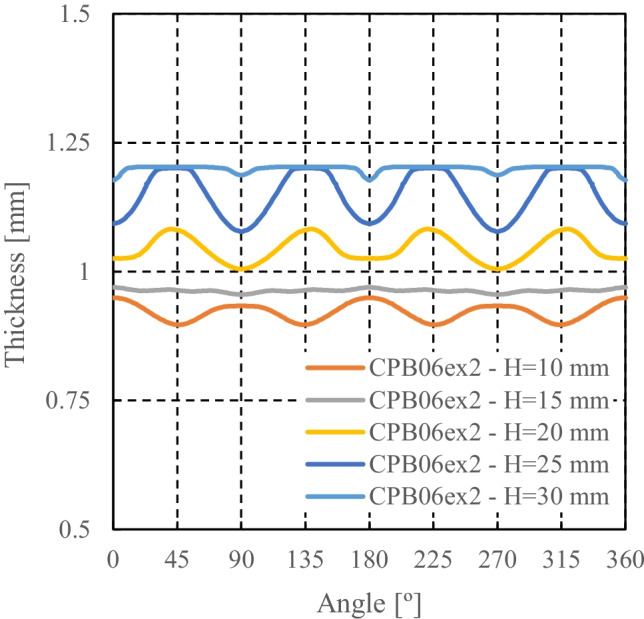
Evolution of the thickness along the cup circumference at different heights H = 10, 15, 20, 25 and 30 mm from the cup bottom, by CPB06ex2 criterion with the anisotropy coefficients identified in “CPB06 criterion by Cazacu, Plunket and Barlat (2006)” Section (Table 29)

The predicted punch force evolution with its displacement and the earing profiles are compared to the experimental ones in Fig. 88(a). The use of a high value for the friction coefficient enables a good prediction of the experimental drawing force (0.8% difference) while the ironing force is overestimated (see in Figs. 72, 75 and 81 the light blue curves obtained for other yield criteria with DD3IMP and *μ* = 0.02 or 0.07). Regarding the earing, 4 ears are predicted, with an amplitude of 1.2 mm against the experimental value of 2.29 mm; the predicted minimum in cup height is deviated to approximately 25° from RD, which can be correlated to the predicted yield stress anisotropy shown in Fig. 89(a) (i.e., an extra inflexion point in the yield stress distribution). Figure 90 presents the thickness distribution along the cup circumference, for different heights. The higher thinning predicted by CPB06ex2 for the lower heights (H = 10 and H = 15 mm), when compared to other results obtained with DD3IMP (see Figs. 74(e) and 83), can be related with the higher value used for the friction coefficient.

##### Caz2018-Orth yield criterion

In the following, Caz2018-Orth or the Cazacu (2018) orthotropic yield criterion (Eq. 16) in conjunction with the anisotropy coefficients of Table 30, (see “Caz2018-Orth or Cazacu (2018) orthotropic yield criterion” Section) will be applied to model cup drawing of the AA 6016-T4 alloy. Two teams reported results for this yield criterion using the same data set: UCoimbra and REEF. UCoimbra performed the numerical simulation with DD3IMP code, while REEF used ABAQUS (implicit). In this case, both teams used the Swift hardening law (Table 32), the same value of 0.020 for the friction coefficient and solid elements.

**Fig. 91:**
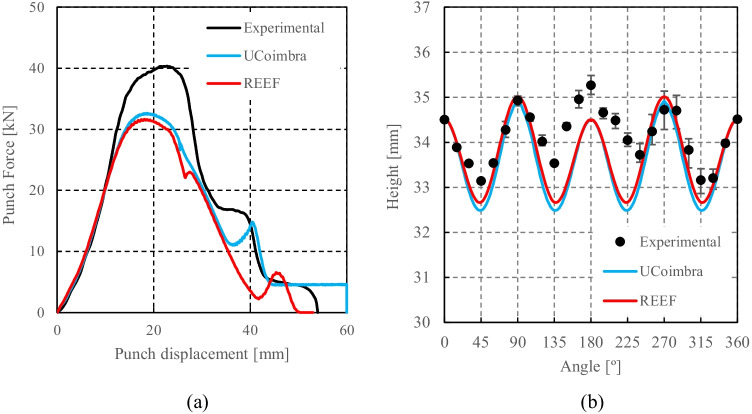
Comparison between experimental and predicted results by Caz2018-Orth criterion with the anisotropy coefficients identified in “Caz2018-Orth or Cazacu (2018) orthotropic yield criterion” Section (Table 30): **a** punch force-displacement, **b** earing profile

**Fig. 92:**
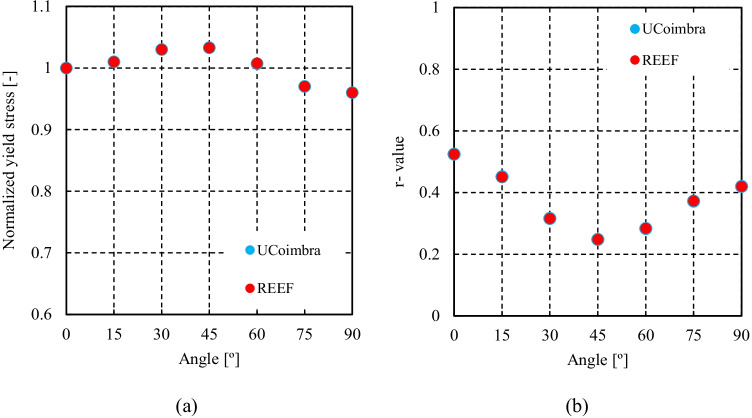
Predicted FEM results by Caz2018-Orth criterion with the anisotropy coefficients identified in “Caz2018-Orth or Cazacu (2018) orthotropic yield criterion” Section (Table 30): **a** uniaxial tensile flow stresses, **b** Lankford coefficients (*r*-values)

The predictions of Caz2018-Orth model for cup drawing are shown in Fig. 91. Note that the cup-height profile is well described. A four-ear profile with ears located at RD and TD and a minimum cup height at 45° from RD is predicted, i.e. as observed in the test. The results obtained with ABAQUS (REEF) predict an average height of *h*_*avg*_ = 33.70 mm against *h*_*avg*_ = 34.70 mm experimentally (1.3% difference), a cup profile amplitude of 2.35 mm against 2.29 mm experimentally (2.5% difference). Furthermore, the predicted maximum height of the cup is 35.01 mm against 35.27 mm experimentally (0.8% difference) and the minimum height of the cup is 32.67 mm against 32.99 mm experimentally (1% difference). In this context, the excellent correlation in the results obtained for the earing profile by both codes should be noted. As shown in Fig. 91(b), the results obtained with DD3IMP present a minimum height of the cup of 32.49 mm against 32.67 mm from ABAQUS, with negligible differences in the maximum height. Both codes predict the same in-plane yield stress and *r*-values evolutions, as shown in Fig. 92.

**Fig. 93:**
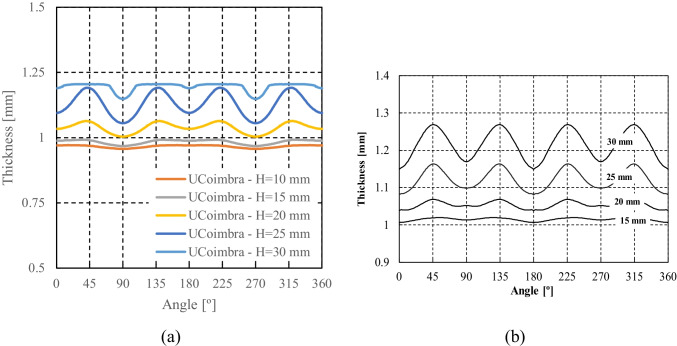
Evolution of the thickness along the cup circumference at different heights H = 15, 20, 25 and 30 mm from the cup bottom, by Caz2018-Orth criterion with the anisotropy coefficients identified in “Caz2018-Orth or Cazacu (2018) orthotropic yield criterion” Section (Table 30): **a** DD3IMP, **b** ABAQUS

Figure 93 presents the evolution of the wall thickness along the cup circumference (taken at different heights from the cup bottom). The slight differences observed in the earing profile can be related with the contact algorithms adopted in both codes (see Table 10). As previously mentioned, the algorithm adopted in DD3IMP imposes the normal constraint more strictly, leading to a higher ironing force (see Fig. 91(a)) and a lower thickness value in the top of the vertical wall (see Fig. 93). Nevertheless, for Caz2018-Orth the earing results are not as sensitive to the differences in the contact algorithm as in the case of Hill48 (see Fig. 72(b)). Note that with von Mises (see Fig. 27(b)) the cup height was also not sensitive to the differences between the codes.

**Fig. 94:**
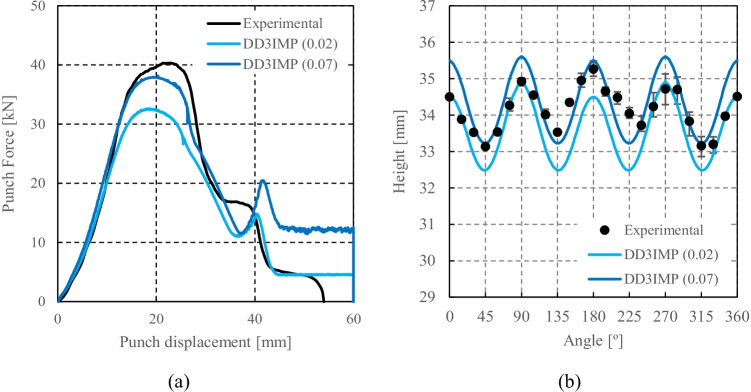
Comparison between experimental and predicted results by Caz2018-Orth criterion with the anisotropy coefficients provided by the organizers (“Caz2018-Orth or Cazacu (2018) orthotropic yield criterion” Section (Table 30)), obtained with DD3IMP for two values of the friction coefficient, 0.02 and 0.07 respectively: (**a**) punch force-displacement, (**b**) earing profile

The influence of the value of the friction coefficient on the results is depicted in Fig. 94, which shows the results obtained with the implicit code DD3IMP (UCoimbra). As observed for the case of von Mises and Hill48, increasing the friction coefficient leads to an increase in maximum drawing force (for Caz2018-Orth a friction coefficient slightly higher than 0.07 may be required to give a perfect match with the experimental one). Likewise, the increase of the friction coefficient leads to the same number of ears (slight decrease in the earing amplitude from 2.42 mm to 2.38 mm, against the experimental value of 2.29 mm).

#### 2-D orthotropic yield functions and solid or shell elements

##### Yld89 yield criterion

USakarya performed the numerical simulation using the Yld89 yield criterion (anisotropy parameters in Table 25) and the Swift law (hardening parameters in Table 32), identified with the experimental data provided by TUAT, as described in “Non quadratic plane-stress yield locus Barlat (Yld89)” Section. The simulation was performed with LS-DYNA code, using shell elements (see details in “Summary of features of FEM simulations” Section).

**Fig. 95:**
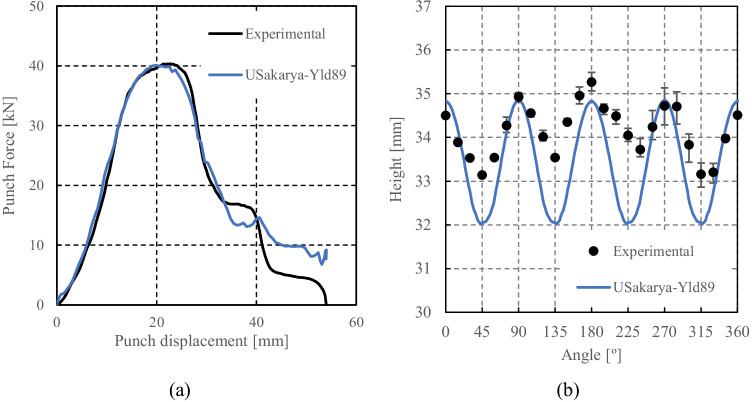
Comparison between experimental and predicted results by Yld89 criterion with the anisotropy coefficients identified in “Non quadratic plane-stress yield locus Barlat (Yld89)” Section (Table 25): **a** punch force-displacement, **b** earing profile

The predicted punch force evolution with its displacement and the earing profile are compared to the experimental ones in Fig. 95. The drawing force is accurately predicted by using *μ* = 0.100. The fact that USakarya used a clearance between the punch and the die of 1.4 mm instead of 1.2 mm, explains the smoother evolution predicted for the punch force, although the ironing effect is not totally avoided.

**Fig. 96:**
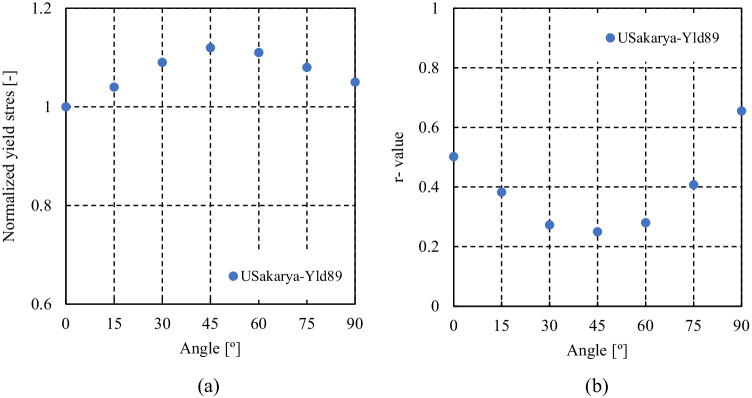
Predicted FEM results by Yld89 criterion with the anisotropy coefficients identified in “Non quadratic plane-stress yield locus Barlat (Yld89)” Section (Table 25): **a** uniaxial tensile flow stresses, **b** Lankford coefficients (*r*-values)

Regarding the earing profile, the trend observed in Fig. 95(b) shows 4 ears, with an amplitude higher than the experimental one. The comparison with the simulations performed with Hill48 (associated flow rule (see Fig. 71)), indicates that Yld89 predicts a similar amplitude for the ears. The sets of material parameters lead to an accurate in-plane distribution of the *r*-values, but overestimate the normalized yield stresses (see Fig. 96 and for experimental results Fig. 5(a)). The average height predicted with Yld89 is closer to the one obtained with Hill48(A) and smaller values of friction coefficient (0.02 and 0.05 instead of 0.10). This can also be related with the higher value considered for the gap between the punch and die, which reduces the ironing effect and, consequently, the average height.

##### Yld2000-2D yield criterion

In this section, the contributions from two teams are analysed. The results presented by USakarya were all obtained using LS-DYNA FE code. USiegen performed numerical simulations using both LS-DYNA and PAMSTAMP. Both teams considered the Swift hardening law (Table 32) and the POSTECH set of anisotropy parameters (see “Yld2000-2D and Yld 2004-18p” Section, Table 27). All numerical simulations were performed with shell elements, (the same type in the case of LS-DYNA (see Table 9)). Moreover, the contact algorithm adopted in LS-DYNA was also identical, although USakarya adopted a slightly higher friction value (*μ* = 0.100 instead of *μ* = 0.075). Moreover, USakarya used a clearance between the punch and the die of 1.4 mm instead of 1.2 mm.

**Fig. 97:**
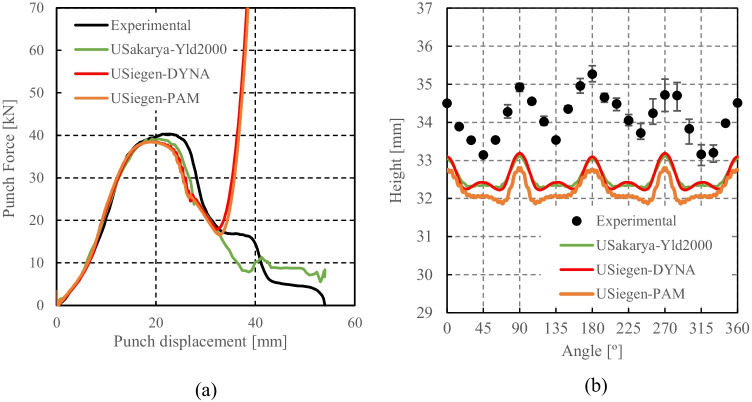
Comparison between experimental and predicted results by Yld2000-2D criterion with the anisotropy coefficients identified in “Yld2000-2D and Yld 2004-18p” Section (Table 27): **a** punch force-displacement, **b** earing profile

The predicted punch force evolution with displacement and the earing profiles are compared to the experimental ones in Fig. 97. All the models slightly underestimate the drawing force. The value predicted by LS-DYNA is slightly higher for the USakarya model, which can be related to the slightly higher value for the friction coefficient. Regarding the ironing force, the use of a higher clearance between the punch and the die, by USakarya, explains the smoother evolution predicted for the punch force in this stage. These results indicate that the effect of the clearance between the die and the punch is mainly visible in the ironing stage.

**Fig. 98:**
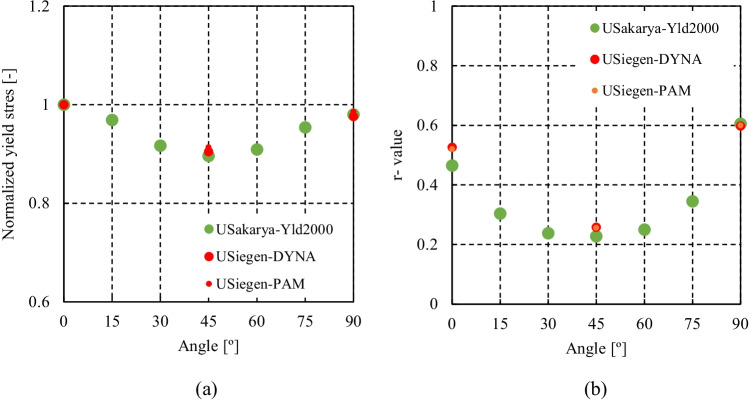
Predicted FE results according to the Yld2000-2D criterion with the anisotropy coefficients identified in “Yld2000-2D and Yld 2004-18p” Section (Table 27): **a** uniaxial tensile flow stresses, **b** Lankford coefficients (*r*-values)

Regarding the earing profile, 8 ears are predicted, with an amplitude lower than the experimental one, as for the Yld2004–18p with the POSTECH data set, although in that case solid element were used (see Fig. 84(a)). USakarya and USiegen simulations provide a similar description of the normalized yield stress and *r*-values in-plane distribution (see Fig. 98). Interestingly, the earing trend for Yld2000-2D presents maxima at 0°, 45° and 90° from RD, as the results obtained by NTNU with Yld2004–18p, identified using only experimental data (parametrization presented in “Yld2000-2D and Yld 2004-18p” Section). Both yield criteria underestimated the earing amplitude. In the case of Yld2000-2D, the average height of the cup is underestimated, although the drawing force is well described. Finally, the clearance between the die and the punch presents a negligible effect in the earing profile, which confirms the previously mentioned difficulties of shell elements in the analysis of the ironing process.

**Fig. 99:**
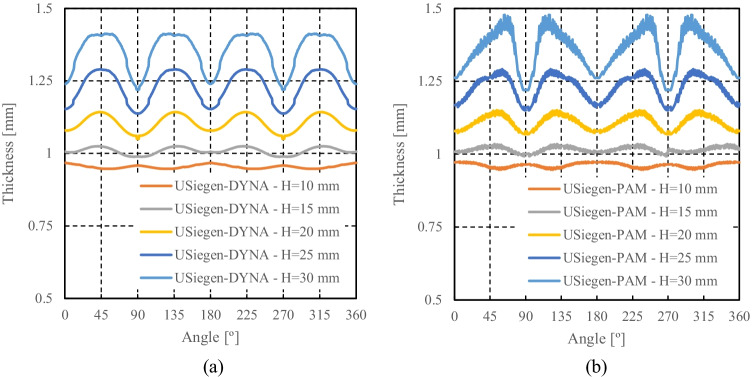
Evolution of the thickness along the cup circumference at different heights H = 15, 20, 25 and 30 mm from the cup bottom, by Yld2000-2D criterion with the anisotropy coefficients identified in “Yld2000-2D and Yld 2004-18p” Section (Table 27): **a** LS-DYNA, **b** PAM-STAMP

Figure 99 presents the thickness distribution along the cup circumference, for different heights, only for the results obtained with Yld2000-2D, by USiegen. Although the evolution predicted for each height is similar, the results obtained with PAMSTAMP present the maximum thickness height slightly away from the 45° from RD, which seems to correlate with the different trend for the earing profile close to this location (see Fig. 97(b)).

##### HomPol4 and HolmPol6 yield criteria 

Hereafter, the contributions from the USakarya team, based on HomPol4 and HolmPol6 yield criteria (homogeneous polynomial yield functions described in “2-D orthotropic yield functions (Yld89, Yld2000-2D, HomPol4, HomPol6)” Section) are presented. The anisotropy parameters were identified in “4^th^ order polynomial model (HomPol4)” Section (Table 23) and “6^th^ order polynomial model (HomPol6)” Section (Table 24), respectively. They were associated either with a Swift isotropic hardening or a single term Chaboche kinematic hardening (see “Identification of hardening laws and data used” Section). The models were separately implemented into LS-DYNA (explicit) and MSC.MARC (implicit) FE codes, using user defined material subroutines, UMAT and Hypela2, respectively. The blank was discretized with shell elements in the model built in LS-DYNA, while solid elements were used in MSC.MARC (see details in Table 9), although it may be arguable the use of 2-D orthotropic yield functions with solid elements. Moreover, in the LS-DYNA model, symmetry conditions were considered, while in MSC.MARC the complete geometry is meshed.

##### HomPol4 and HolmPol6 yield criteria with isotropic hardening

**Fig. 100:**
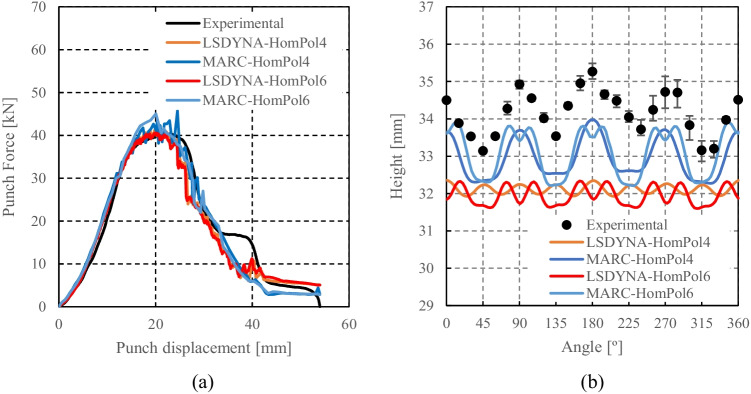
Comparison between experimental and predicted results by HomPol4 and HomPol6 criterion with the anisotropy coefficients identified in “4^th^ order polynomial model (HomPol4)” and “6^th^ order polynomial model (HomPol6)” Sections (Table 23 and Table 24), using LS-DYNA and MSC.MARC (additive decomposition): **a** punch force-displacement, **b** earing profile

For isotropic hardening law (Swift) case, the predicted punch force-displacement curves and earing profiles are compared with the experimental data in Fig. 100. As observed in Fig. 100(a), the slopes of the predicted punch force-displacement curves are compatible with each other and with the experimental data. All models approximately captured the maximum punch force. It should be mentioned that USakarya team avoided the ironing effect by increasing the gap between the punch and the die in the numerical model. Figure 100(b) shows that the cup height is underestimated as well as the earing amplitude, particularly in the simulations performed with LS-DYNA, i.e. with shell elements. A similar behaviour was observed in Fig. 97(b) when the same type of elements, but the Yld2000-2D yield criterion was used. Only the numerical simulation performed with MSC.MARC and the homogeneous polynomial yield function of degree 4 enables the prediction of 4 ears, as in the experimental results.

**Fig. 101:**
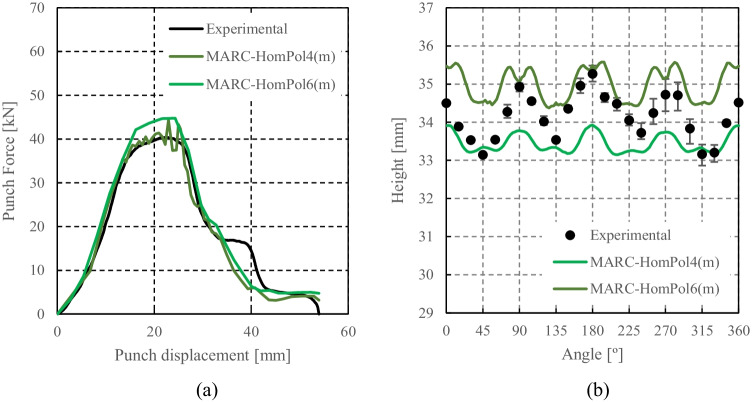
Comparison between experimental and predicted results by HomPol4 and HomPol6 criterion with the anisotropy coefficients identified in “4^th^ order polynomial model (HomPol4)” and “6^th^ order polynomial model (HomPol6)” Sections (Table 23 and Table 24), using MSC.MARC (multiplicative decomposition): **a** punch force-displacement, **b** earing profile

**Fig. 102:**
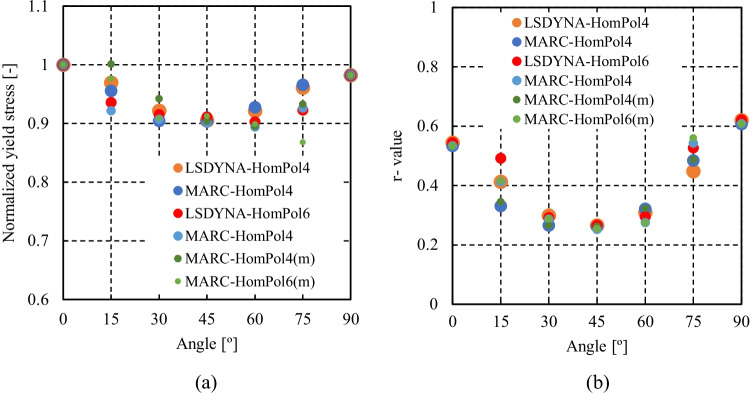
Predicted FEM results by HomPol4 and HomPol6 criterion with the anisotropy coefficients identified in “4^th^ order polynomial model (HomPol4)” and “6^th^ order polynomial model (HomPol6)” Sections (Table 23 and Table 24): **a** uniaxial tensile flow stresses, **b** Lankford coefficients (*r*-values). The label (m) corresponds to results obtained with MSC.MARC (multiplicative decomposition)

In MSC.MARC code, FE analyses were also performed based on multiplicative decomposition theory, which considers the decomposition of the deformation gradient into elastic and plastic components. Mandel’s intermediate isoclinic configuration was adopted for orthotropic plasticity model. Figure 101 shows the numerical results of punch force – displacement curve and earing profile predicted by multiplicative decomposition methodology. It can be seen from Fig. 101(a) that the numerical punch force – displacement curves were consistent with the experimental curve. As for the earing profile, the multiplicative decomposition leads to an increase of the cup height. Nevertheless, the earing amplitude shows a decrease, particularly for HomPol4. Moreover, both the HomPol4 and the HomPol6 predict more than 4 ears. As shown in Fig. 102, all models enable a similar description of the in-plane distribution of the normalized yield stress and *r*-values, but in this case it is very difficult to correlate these results with the earing profile.

##### HomPol4 and HolmPol6 yield criteria with isotropic and kinematic hardening

The results from USakarya team considering a combined hardening law relies on a Swift hardening law and the single term Chaboche nonlinear kinematic hardening rule, as described in “Identification of hardening laws and data used” Section, Table 32.

**Fig. 103:**
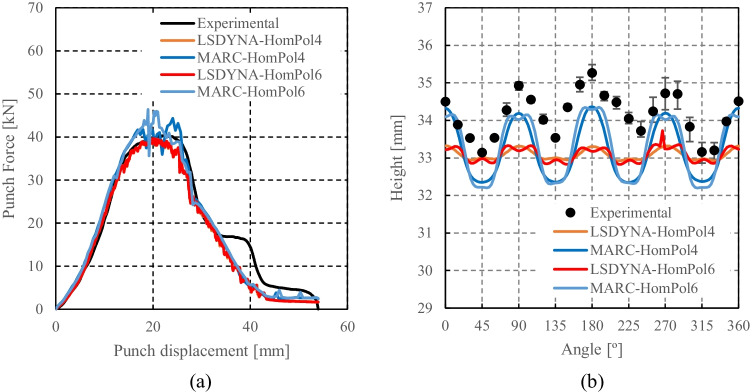
Comparison between experimental and predicted results by HomPol4 and HomPol6 criterion with the anisotropy coefficients identified in “4^th^ order polynomial model (HomPol4)” and “6^th^ order polynomial model (HomPol6)” Sections (Table 23 and Table 24), using LS-DYNA and MSC.MARC (additive decomposition): **a** punch force-displacement, **b** earing profile

The predicted punch force-displacement curves and earing profiles are compared with the experimental results in Fig. 103. Also in this case, the predicted punch force-displacement curves of both criteria are compatible with each other and with the experimental data (see Fig. 100(a)). The comparison of Fig. 100(b) with Fig. 103(b) shows that the average cup height is better predicted when adopting the combined hardening law, particularly by the simulations performed with MSC.MARC. In fact, wrinkling was observed at several locations around the cup perimeter when the isotropic hardening rule was assumed. These wrinkles were substantially eliminated when the combined hardening rule was adopted.

**Fig. 104:**
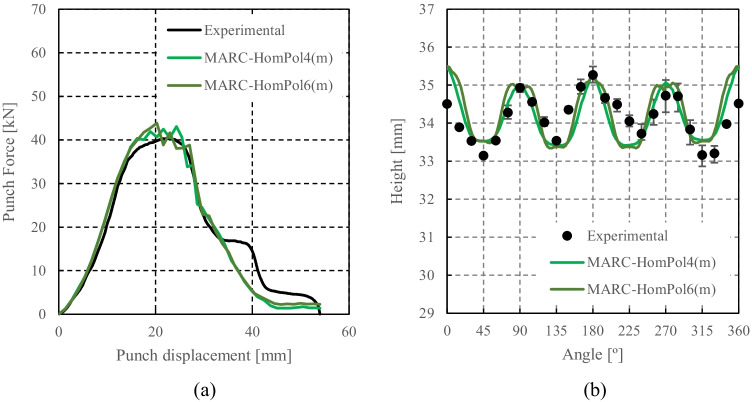
Comparison between experimental and predicted results by HomPol4 and HomPol6 criterion with the anisotropy coefficients identified in “4^th^ order polynomial model (HomPol4)” and “6^th^ order polynomial model (HomPol6)” Sections (Table 23 and Table 24), using MSC.MARC (multiplicative decomposition): (**a**) punch force-displacement, (**b**) earing profile

**Fig. 105:**
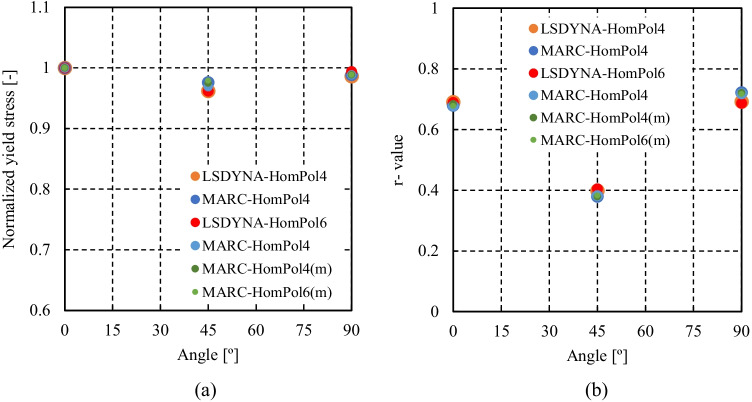
Predicted FEM results by HomPol4 and HomPol6 criterion with the anisotropy coefficients identified in “4^th^ order polynomial model (HomPol4)” and “6^th^ order polynomial model (HomPol6)” Sections (Table 23 and Table 24): **a** uniaxial tensile flow stresses, **b** Lankford coefficients (*r*-values). The label (m) corresponds to results obtained with MSC.MARC (multiplicative decomposition)

Finally, the multiplicative decomposition theory was also tested in MSC.MARC FE code. Figure 104 shows the predictions of punch force – displacement curve and earing profile. As before, it can be seen from Fig. 104(a) that the impact on the numerical punch force – displacement curves is marginal. As for the earing profile, combined hardening seems to affect the results positively. The cup height values were increased for HomPol4 and decreased for HomPol6, in comparison with the results based on isotropic hardening. It is seen that the predicted results from multiplicative decomposition with combined hardening were in best agreement with experimental data. Furthermore, the predicted cup profiles obtained from HomPol4 and HomPol6 with combined hardening are almost identical with each other. Nevertheless, it should be mentioned that while the HomPol4 predicts four ears, the HomPol6 results in a higher number. Finally, the trend predicted for the normalized yield stress and *r*-value directionalities is shown in Fig. 105. The comparison with Fig. 101 highlights small differences, particularly for the *r*-values. Previous results indicate that taking into account the kinematic hardening behaviour has small impact on the earing trend [103].

### Results of phenomenological models identified with physical and virtual crystal plasticity tests

#### Yld2004–18p based on physical experiments and virtual tests with DAMASK

NTNU team performed two identifications for the Yld2004–18p yield criterion: using only mechanical data or considering also computer experiments, using DAMASK, as detailed in “RVE Damask Simulations” and “Yld2000-2D and Yld 2004-18p” Sections (anisotropy coefficients are given in lines 2 and 3 of Table 28). Numerical simulations were computed with these sets of anisotropy parameters associated with the Voce hardening law (see Table 32) and a friction coefficient *μ* = 0.09 in ABAQUS (implicit).

**Fig. 106:**
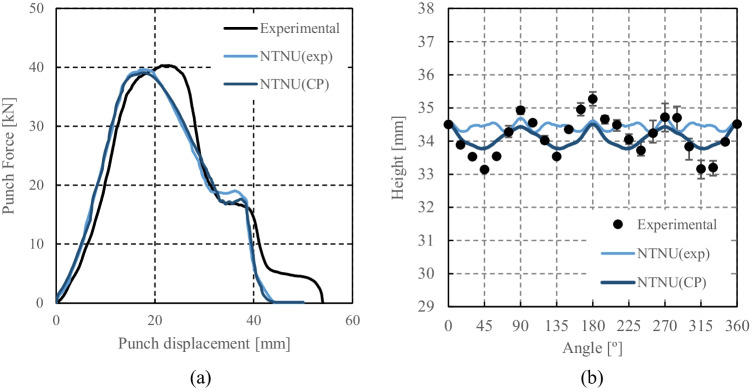
Comparison between experimental and predicted results by Yld2004–18p criterion with the anisotropy coefficients identified based only in mechanical data or considering also computer experiments, as described in “RVE Damask Simulations” and “Yld2000-2D and Yld 2004-18p” Sections (Table 28): **a** punch force-displacement, **b** earing profile

Figure 106(a) shows the comparison between the experimental and numerical punch force evolution with displacement, for both sets of parameters. The predicted drawing force agrees well with the experiments. The maximum value of punch force obtained from the simulations is similar to that in the experiment, but it occurs for a smaller displacement than in the experiment. After this peak point, both simulations underestimate the punch force, but the use of solid elements enables capturing the ironing effect. The comparison of the forces predicted by the two sets of parameters identified by NTNU shows that they present a good agreement; i.e. this variable is not sensitive to the calibration method of the constitutive model used in the analysis. The fact that the set of parameters identified based on the experimental data leads to a slightly higher value for the ironing force can be related with the fact that this model presents globally lower *r*-values (see “RVE Damask Simulations” Section), which justifies a slightly higher increase of thickness of the material initially located in the flange.

Figure 106(b) shows the experimental and simulated earing profiles at the end of the cup drawing. The set of parameters identified using both experimental and computer results, leads to 4 ears, with an amplitude lower than the experimental one; while the earing profile with the other set of parameters has a higher average level, a profile closer to 12 ears and very low amplitude. Thus, the prediction accuracy of the model using virtual tests in its identification process is higher than the one based only on experiments. For this model, the amplitude of the ears increases for the identification performed with both mechanical and computer experiments although the amplitude of variation for the normalized yield stress is lower (see “RVE Damask Simulations” Section, Fig. 40(a)).

Note that Han et al. [42] compared the simulation results of FE cup drawing model for an AA 2090-T3 aluminium alloy sheet with Yld2004–18p and Yld2000-2D yield loci identified by DAMASK. They pointed the impact of the texture representation of the central sheet layer or of the complete sheet in their FE results. They demonstrated that taking into account successive yield locus identified for different plastic strain values, during the simulations, improved the earing predictions of Yld2000-2D, but not of Yld2004–18p. The average error for Yld2004–18p was higher than for Yld2000-2D with or without texture updating effect. Their assumption to explain this surprising observation of lower performance of Yld2004–18p is related to the impact of neglecting the strong texture gradient across the thickness direction of the sheet (Representative volume elements of central layer texture or complete texture within DAMASK computation). As Yld2004–18p law has more parameters than Yld2000-2D, the former is able to describe in a finer way the texture effect and a systematic error could be magnified in the plastic anisotropy modelling.

#### Yld2000-2D based on virtual tests with VPSC

UGent team performed the numerical simulations with ABAQUS (implicit) considering the blank discretized with shell elements (see Table 9). The hardening Voce law and the phenomenological yield function Yld2000-2D, presented in “2-D orthotropic yield functions (Yld89, Yld2000-2D, HomPol4, HomPol6)” Section, are implemented in ABAQUS (implicit) via a UMAT subroutine. The calibration of the yield locus is described in “VPSC model” Section (Table 19) and the hardening parameters are provided in “Identification of hardening laws and data used” Section, Table 32.

**Fig. 107:**
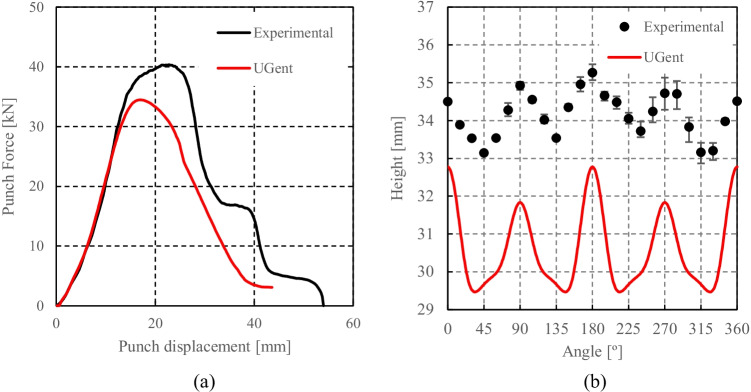
Comparison between experimental and predicted results by Yld2000-2D criterion, with the anisotropy coefficients identified by VPSC virtual tests, as described in “VPSC model” Section: **a** punch force-displacement, **b** earing profile

Figure 107(a) shows the punch force measured during the cup drawing process together with the one extracted from the simulation. The punch force magnitude during the initial stage of the experiment is well predicted by the numerical model. However, the FE analysis slightly underestimates the peak force and reaches a maximum earlier than in the experiment. The shell element adopted by UGent (S4R, see Table 9) leads to a clear underestimation of the ironing force, unlike other shell formulations (see e.g. Fig. 71), which is certainly connected with the assumptions adopted to estimate the transverse strain.

**Fig. 108:**
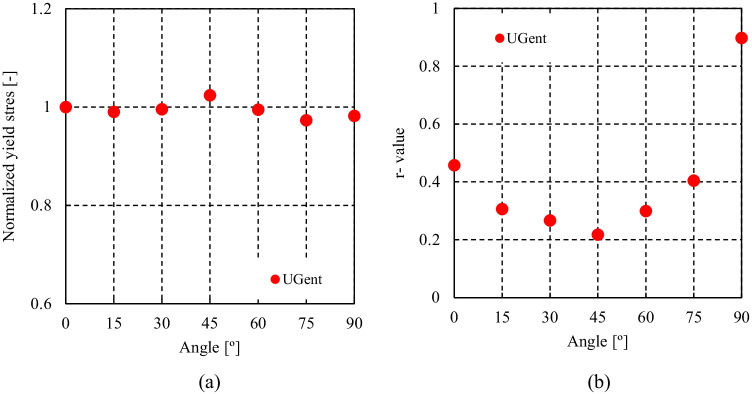
Predicted FE results by Yld2000-2D criterion, with the anisotropy coefficients identified by VPSC virtual tests, as described in “VPSC model” Section: **a** uniaxial tensile flow stresses, **b** Lankford coefficients (*r*-values)

Figure 107(b) shows the experimental and predicted earing profiles at the end of the cup drawing process. The FE model predicts the same number of ears as in the experiment. A quasi-perfect agreement is observed for the angles at which the highest and lowest heights are measured. On the other hand, the model cannot replicate the exact evolution of the measured profile. It is interesting to note that the minimum presents a shift towards the RD, similar to the one obtained with CPB06ex2 (see Fig. 88(b)). This can be related with the trend observed for the in-plane distribution of the normalized yield stress for both models, which presents an extra inflexion point at a similar location (see Figs. 89 and 108). Interestingly, all models that consider the Yld2000-2D (see also Fig. 97(b)) underestimate the average cup height, whatever the parametrization adopted. The parametrization proposed by UGent leads to the best prediction of the number of ears and their amplitude.

It is important to mention that the shape of the yield function is calibrated using the data from the virtual experiments at 10% of effective plastic strain. This assumption is supported by the EBSD measurements performed after pre-straining that show that no significant texture evolution is observed (see Figs. 6 and 14). Besides, the parameter identification method used for the calibration of the anisotropic parameters for the phenomenological Yld2000-2D criterion is performed giving more weight to the *r*-values rather than to the stress amplitude (see “VPSC model” Section). This approach is chosen in order to get a better prediction of the earing profile. However, the inaccurate description of the yield strengths (see Fig. 43 the shear strength) might have resulted in the underestimation of the global punch force.

#### Caz2018singlecrys assuming or not a pure cube texture

As discussed in “Caz2018polycrys, a polycrystalline model based on Cazacu single crystal law Caz2018singlecrys” Section, a simplified approach was used by POSTECH based on the assumption that the AA 6016-T4 material has a pure cubic texture (i.e. 100% of the grains are with [100] along RD, and [001] along ND). POSTECH uses the single crystal law, Caz2018singlecrys (see Eq. (30), with crystal axes coinciding with (x,y,z) axes), to simulate the cup drawing. For this purpose, POSTECH implemented the single crystal model in the FE code ABAQUS explicit (subroutine VUMAT) in conjunction with a Voce hardening law (parameters in Table 32). They used their own set of anisotropy parameters (see “Caz2018singlecrys” Section, Table 20), solid elements (C3D8R), a coarse mesh (see Table 9 and Fig. 28) and a friction coefficient *μ* = 0.1.

**Fig. 109:**
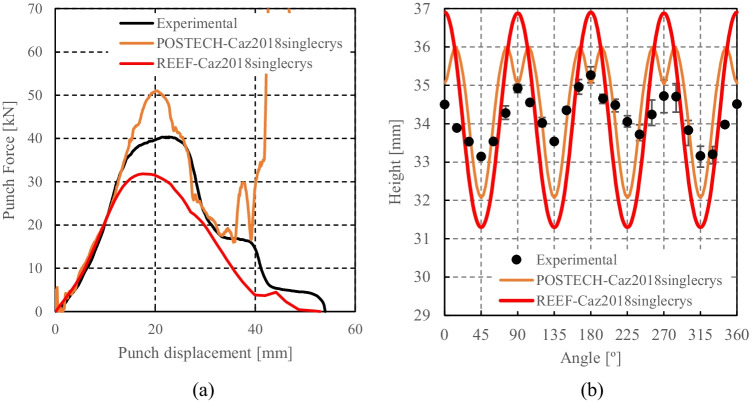
Comparison between experimental and predicted results by Caz2018singlecrys for the data set of “Caz2018polycrys” Section (Table 15 - REEF) and of “Caz2018singlecrys” Section (Table 20 – POSTECH): **a** punch force-displacement, **b** earing profile

The FE simulation results reported in Fig. 109 for the POSTECH set of anisotropy parameters (see Table 20) predict 8 ears and the punch force evolution with displacement is overestimated for both the drawing and the ironing stage. As discussed in “Caz2018singlecrys” Section, for this set of anisotropy parameters the yield surface is not convex (see Fig. 46(b)).

**Fig. 110:**
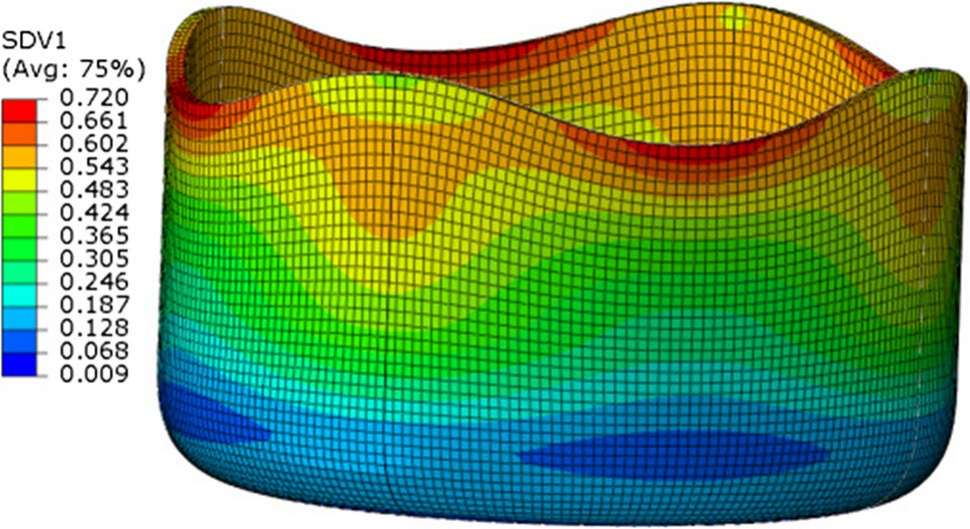
Predicted isocontours of equivalent plastic strain predicted by Caz2018singlecrys yield criterion with the anisotropy coefficients given in “Caz2018polycrys” Section (Table 15 - ideal cubic texture)

As shown in “Caz2018polycrys” Section, if the assumption of a pure cube texture is made for the AA 6016-T4 material, REEF identified in a different way the same single crystal law (i.e. they did not impose *n*_4_=0). The set of anisotropy parameters is given in Table 15-line 1. The implementation by REEF of the single crystal model was done in ABAQUS Standard, using a fully implicit integration algorithm for solving the governing equations. The predicted punch force vs. displacement and earing profile obtained using this set of anisotropy coefficients, a Swift hardening law (parameters in Table 32), and this team’s implementation are shown in Fig. 109. The predicted isocontours of the equivalent plastic strain for the full drawn cup are given in Fig. 110. The FE REEF mesh for a quarter of the blank consisted of a refined mesh of 10,900 solid elements (C3D8R), with three elements in the thickness direction and friction coefficient *μ* = 0.02. Note that the predicted earing profile shows the same trends as the experimental one, namely 4 ears with maximum in cup height at 0° and 90° and a minimum cup height at 45°. Moreover, there is correlation between the predicted *r*-values variation (see Fig. 33(b)) and the predicted earing profile. However , the assumption of ideal cube texture (which implies *r*_0_ = *r*_90_ = 1) is not valid for the AA 6016-T4 material which has *r*_0_ = 0.525 and *r*_90_ = 0.429, hence, a quantitative agreement for the ears amplitude cannot be expected.

#### Facet-3D based on ALAMEL virtual tests

**Fig. 111:**
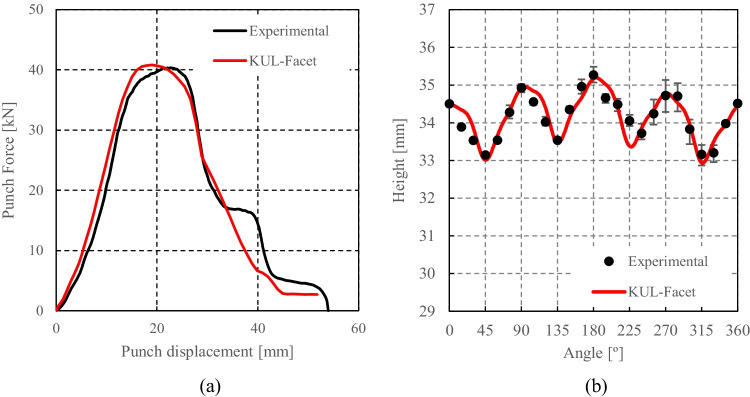
Comparison between experimental and predicted results by the Facet-3D model identified by ALAMEL, as described in “Facet-3D model” Section: **a** punch force-displacement, **b** earing profile

**Fig. 112:**
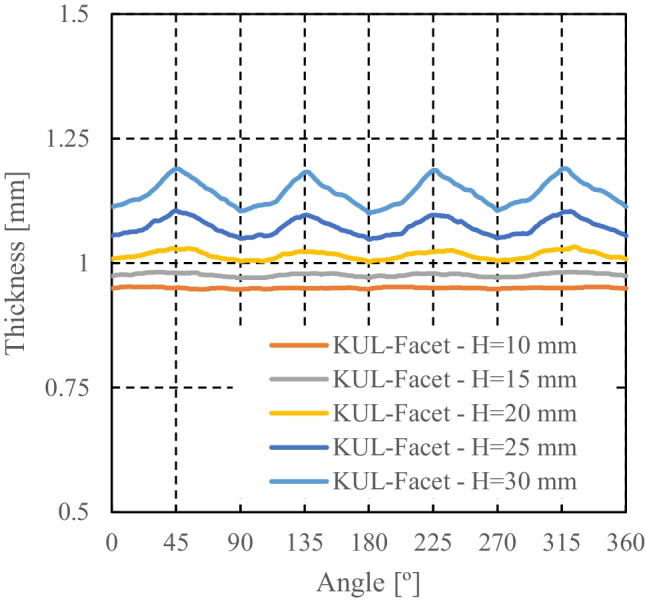
Evolution of the thickness along the cup circumference at different heights H = 15, 20, 25 and 30 mm from the cup bottom, by the Facet-3D model identified by ALAMEL (see “Facet-3D model” Section)

This section presents the results reported by KUL, using the Facet-3D model identified with ALAMEL, as described in “Facet-3D model” Section. As mentioned in “Summary of features of FEM simulations” Section, the numerical simulation was performed with ABAQUS (explicit), using the complete model, with the blank discretized with continuum shell elements (SC8R). The blank initial position was offset (−0.3 mm in RD and 0.008 mm in TD, i.e. half of the cup height difference) to account for the asymmetry in the experimental earing profile. Figure 111 presents the comparison between the experimental and numerical punch force evolution with its displacement and the earing profiles. The evolution of the thickness along the cup circumferential direction, at different heights, is shown in Fig. 112. The use of a friction coefficient of 0.090 enables an accurate description of the drawing force as well as of the earing profile. Nevertheless, the ironing force is underestimated, although the contact algorithm selected (see Table 10) guarantees a maximum thickness value equal to the clearance between the die and the punch (see Fig. 112). A previous study that compares the use of different types of elements implemented in ABAQUS indicates that continuum shell elements tend to predict slightly lower ironing forces than solid elements [22]. In this case, it is interesting to note that this constitutive model predicts a lower average thickness value for the higher cup heights (H = 25 and H = 30 mm), when compared with the ones predicted by other phenomenological models (see Figs. 74, 83, 90 and 93), which can also help justifying the lower value predicted for the ironing force. Regarding the earing profile, the amplitude and average height are well captured, although the experimental normalized tensile yield stress values are not well predicted by ALAMEL and Facet-3D (see Fig. 32(a)).

### Results with crystal plasticity based constitutive models

#### Caz2018polycrys based on Caz2018singlecrys

In the following are presented the cup drawing results obtained with Caz2018polycrys, the polycrystalline model based on the Cazacu et al. [15] single crystal criterion (Eq. (30) and the values of anisotropy coefficients corresponding to the actual texture, given in Table 15) and Swift isotropic hardening model (see parameters Table 16). As already mentioned, a polycrystalline aggregate is associated with each FE integration point (see “Caz2018polycrys, a polycrystalline model based on Cazacu single crystal law Caz2018singlecrys” Section for more details). A friction coefficient of 0.02 was applied and a total of 10,900 solid elements (ABAQUS C3D8R) have been used to mesh the quarter of the blank, resulting in a total of 2,725,000 grains in the FE simulation.

**Fig. 113:**
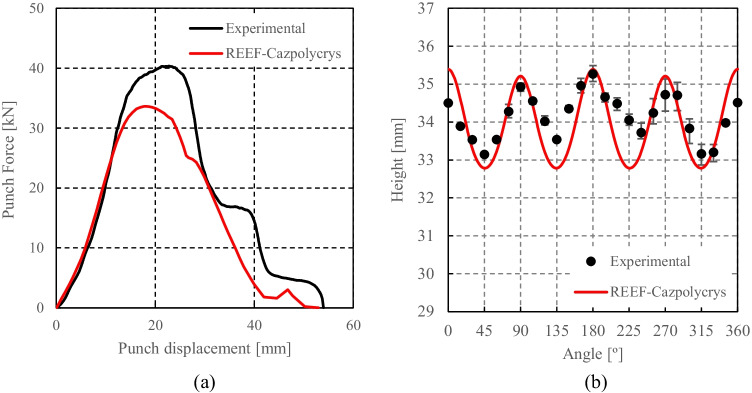
Comparison between experimental and predicted results by Caz2018polycrys model based on Caz2018singlecrys (Eqs. 30–32): **a** punch force-displacement, **b** earing profile

**Fig. 114:**
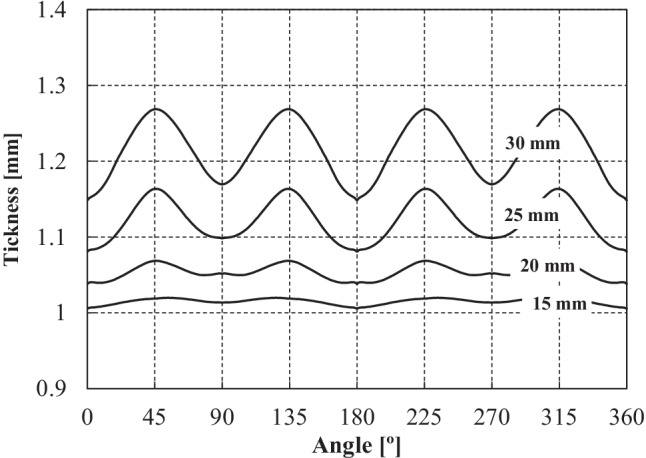
Evolution of the thickness along the cup circumference at different heights H = 15, 20, 25 and 30 mm, respectively from the cup bottom, by Caz2018polycrys model based on the Caz2018singlecrys model (Eqs. 30–32)

Figure 113(a) shows the predicted forming force as a function of the punch stroke in comparison with experimental data. The underestimation is consistent with the low friction coefficient used. The prediction of Caz2018polycrys model for the cup earing profile is shown in Fig. 113(b). Note that the predicted cup-height profile is in good agreement with the experimental data. Specifically, it is a four-ear profile with ears located at RD and TD and a minimum cup height at 45° from RD, as observed experimentally. Moreover, the predicted average height is *h*_*avg*_ = 33.93 mm against *h*_*avg*_ = 34.10 mm experimentally (0.6% difference), the cup profile amplitude being of 2.61 mm against 2.29 mm experimentally (10% difference). Furthermore, Caz2018polycrys model predicts a maximum height of the cup of 35.39 mm against 35.27 mm experimentally (0.3% difference) and a minimum height of the cup of 32.78 mm against 32.99 mm experimentally (0.6% difference). In Fig. 114, the predicted evolution of the wall thickness along the cup circumference, taken at different heights from the cup bottom, is presented.

#### Minty

In this section, the results obtained by ULiege using Minty (see “Minty law” Section) and the Swift hardening law are presented. According to the information given in “Summary of features of FEM simulations” Section, ULiege used solid elements and its own FE implicit software Lagamine, considering a constant friction coefficient of μ = 0.082.

**Fig. 115:**
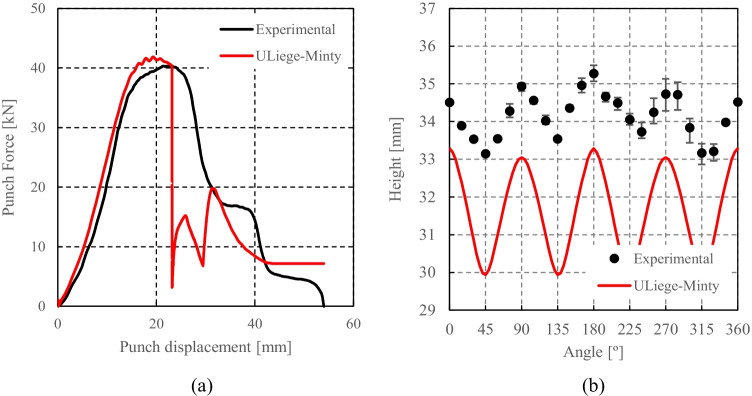
Comparison between experimental and predicted results according to the Minty law, as described in “Minty law” Section: **a** punch force-displacement, **b** earing profile

**Fig. 116:**
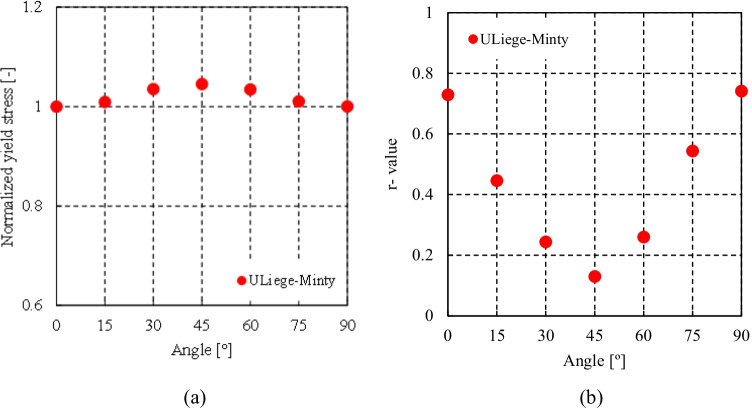
Predicted FEM results according to the Minty law, as described in “Minty law” Section: **a** uniaxial tensile flow stresses, **b** Lankford coefficients (*r*-values)

Figure 115(a) shows the predicted forming force as a function of the punch stroke in comparison with the experimental data. Despite the problems previously mentioned in “Hill48 with associated flow rule” Section, overall, the maximum drawing force is well predicted. The comparison with the results obtained with Lagamine for Hill48 yield criterion (see Fig. 72) shows that the model adopting the Minty law leads to a slightly higher value for the maximum drawing force, which can be related with the selection of the Swift hardening law instead of Voce. Figure 115(b) presents the earing profile predicted with the model adopting the Minty law. As for Hill48 yield criterion (see Fig. 72(b)), 4 ears are predicted with the same trend of the experimental ones. However, the average cup height is clearly underestimated by the model adopting the Minty law. In addition, the amplitude of the earing profile becomes higher, when compared with the one predicted by the Hill48 yield criterion. This change in amplitude can be explained by the increase in the amplitude of variation of the *r*-values in-plane distribution predicted by the model adopting the Minty law, when compared with the one obtained by Hill48 yield criterion (see Fig. 73(b) and Fig. 116(b)). In this context, it is interesting to mention that the Minty law and Caz2018polycrys model predict the same trend; the predictions for *r*-values for RD and TD are very close, with maximum differences being observed for the 45° orientation (see Figs. 35(b) and 116(b)).

### Summary of the results and discussion

Here after, first the punch force and then the earing profile predictions are discussed.

**Fig. 117:**
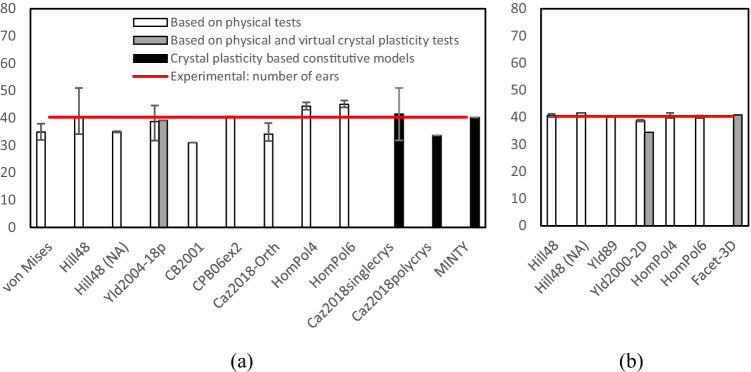
Maximum punch force predicted in *the drawing stage* in function of the yield criterion or constitutive model, for FE models using for the blank discretization: **a** solid (team used values from μ = 0.01 up-to μ = 0.100) and **b** shell elements (all teams used μ = 0.07 or higher values)

**Fig. 118:**
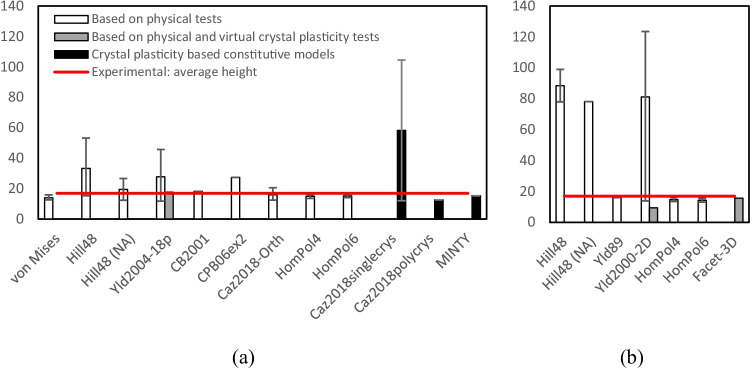
Maximum punch force predicted in *the ironing stage* in function of the yield criterion or constitutive model, for FE models using for the blank discretization: **a** solid and **b** shell elements

Globally, all FE models enable the accurate prediction of the first peak of the punch force related to cup drawing, with the adjustment of the friction coefficient (within a reasonable physical range). Nevertheless, they show a considerable raise of the punch force during the ironing stage, particularly when using shell elements (except for ABAQUS: the S4R element used by UGent and SC8R element used by KUL). These results are summarized in Figs. 117 and 118, which show the average of the maximum punch force during the drawing and the ironing stages, for each yield criterion/constitutive model and identification strategy adopted. Note that some models were selected by only one team, which justifies the lack of error bars.

Some teams (REEF, UCoimbra) that used solid elements did not perform any optimization of the friction coefficient and just select a low value (μ = 0.02), which explains the underestimation of the drawing force (Fig. 117). However, the teams using friction values from 0.05 until 0.1 and solid elements predicted the drawing force peak with higher accuracy than the teams that used shell elements, which all relied on friction values equal or higher than 0.07.

In Fig. 118, the overestimation of the ironing force is observed for many participants and it is related with the strategies adopted to deal with transverse strains when using shell elements (different assumptions in LS-DYNA, PAMSTAMP and ABAQUS (S4R and SC8R), as detailed in Table 9). The lack of accuracy in the transversal strain prediction also explains the poor precision of shell elements for the thickness distribution along the cup circumference, for different heights.

Globally, the solid formulations clearly provide a more realistic ironing force (see Fig. 118). Nevertheless, even for this type of elements, the ironing force tends to be overestimated, which seems to be related with the assumed tool rigidity. An elastic behaviour would slightly widen the drawing gap during ironing where very large surface pressures prevail and therefore a lower force is expected. The improved accuracy of the ironing force and shape of the force curve are pointed by the simulations using an increased clearance (USakarya with Yld89, HomPoI4 and HomPoI6). Note that just based on these synthetic figures, one could enhance 3 interesting configurations:

- Yld2004–18p - ABAQUS implicit – solid element from NTU, with an identification based on 7 virtual tensile tests by Damask with an FFT approach, 7509 grains in a RVE and a physical tensile test in RD (see “Yld2004-18p based on physical experiments and virtual tests with DAMASK” Section).
- FACET-3D – ABAQUS explicit - continuum shell from KUL, with an identification based on 200 virtual tests relying on 10,000 grains and the ALAMEL crystal plasticity model (see “Facet-3D based on ALAMEL virtual tests” Section) although, the ironing force is underestimated and the start of the ironing stage occurs for higher values of punch displacement than in the experimental test (see Fig. 111).
- Minty – Lagamine implicit - solid element from ULiege, with an interpolation yield locus approach based on 1000 crystals and a simple Full Taylor plasticity approach (see “Minty” Section); although, the start of the ironing stage is not correctly predicted and some issues still need to be further analysed.

This indicates that the use of crystal plasticity computations to complement physical tests in the identification of the parameters of phenomenological models may contribute to improve the prediction of the ironing force.

Regarding the punch force prediction, in conclusion, as the friction coefficient was not measured or suggested by the benchmark committee, most teams calibrated a constant Coulomb friction coefficient by fitting the experimental evolution of the punch drawing force. This has led to a very good description of the drawing force (see Fig. 117) for models using solid elements. Nevertheless, using a constant friction coefficient does not enable to predict with great accuracy both the drawing and ironing force. While an evolving friction coefficient may help to further fine tune the predictions of the forces, improved prediction of the thickness at the top of the vertical wall may require consideration of the deformation of the forming tools. In practice, an easy and quick assessment of the friction coefficient for drawing or ironing stages seems difficult, due to experimental issues and the uncertainty related with the tools surface state and lubrication conditions. This topic as well as the reasons for the measured thickness being greater than the gap (see “Measurement of tools and cup thickness by different methods” Section) clearly need to be addressed in future works. Within the next ESAFORM 2022 and Numisheet 2022 conferences, new studies on this benchmark will be published and, as all data will be public, the whole community will also be able to exploit it.

**Fig. 119:**
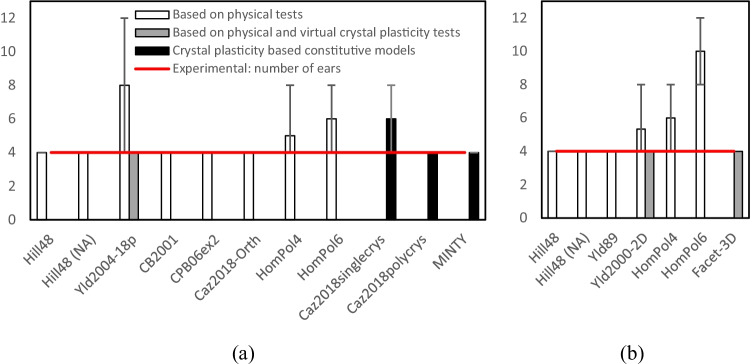
Number of ears predicted in function of the yield criterion or constitutive model, for FE models using for the blank discretization: **a** solid and **b** shell elements

**Fig. 120:**
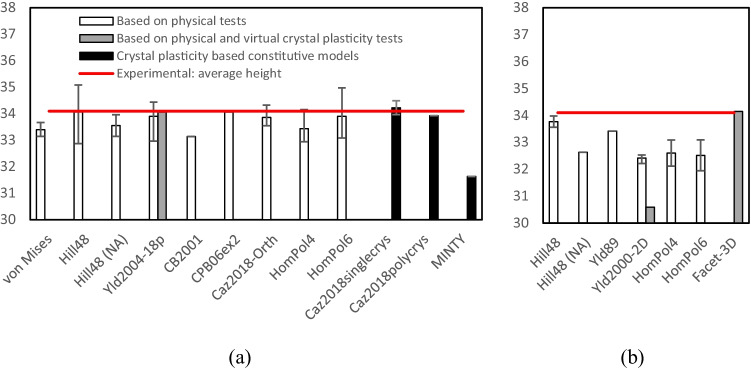
Average height of the cup predicted in function of the yield criterion or constitutive model, for FE models using for the blank discretization: **a** solid and **b** shell elements

**Fig. 121:**
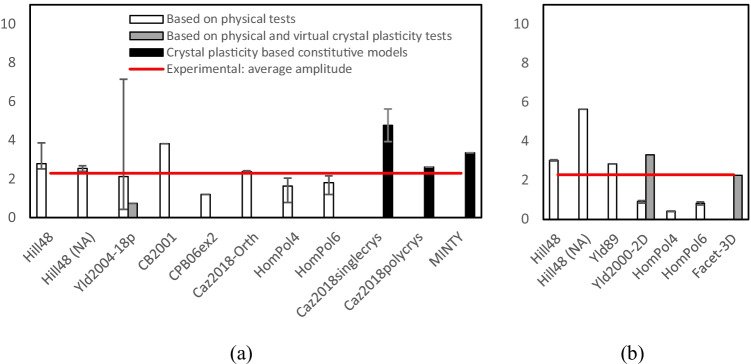
Average amplitude of the ear profile predicted in function of the yield criterion or constitutive model, for FE models using for the blank’s discretization: **a** solid and **b** shell elements

The number of ears, average height and average amplitude predicted by each FE model are presented in Table 35 and Table 36, for solid and shell/continuum-shell elements, respectively. In each table, the FE models are also separated taking into account the strategy used for the parameters identification. Table 35 and Table 36 also present the global error, evaluated as:

72 $$\mathrm{Global}\; \mathrm{error}={\left(\frac{n_{\mathrm{avg}}^{\mathrm{num}}-{n}_{\mathrm{avg}}^{\mathrm{exp}}}{n_{\mathrm{avg}}^{\mathrm{exp}}}\right)}^2+{\left(\frac{h_{\mathrm{avg}}^{\mathrm{num}}-{h}_{\mathrm{avg}}^{\mathrm{exp}}}{h_{\mathrm{avg}}^{\mathrm{exp}}}\right)}^2+{\left(\frac{a_{\mathrm{avg}}^{\mathrm{num}}-{a}_{\mathrm{avg}}^{\mathrm{exp}}}{a_{\mathrm{avg}}^{\mathrm{exp}}}\right)}^2$$

where *n*_avg_, *h*_avg_ and *a*_avg_ represent the number of ears, average height and average amplitude, respectively, and the superscripts “num” and “exp”, correspond to numerical and experimental results. Global error values lower than 0.01 are highlighted in bold in Tables 35 and 36. In Fig. 119, 120 and 121, the average number of ears, average height and average amplitude were determined, for each yield criterion/constitutive model and identification strategy adopted.

**Table 35:** FE prediction synthesis and error vs. experimental values for models using a blank discretization by solid elements. Global error values lower than 0.01 are highlighted in bold

					Number of ears	Average height (mm)	Average amplitude (mm)	
	Experimental result				4	34.1	2.29	
	Yield criterion	Code	Team	μ	Number of ears	Average height(mm)	Average amplitude(mm)	Global error
Phenomenological models identified based on mechanical data	von Mises	ABAQUS	REEF	0.020	0	33.15	0	2.001
		ABAQUS	REEF	0.070	0	33.62	0	2.000
		DD3IMP	UCoimbra	0.020	0	33.17	0	2.001
		DD3IMP	UCoimbra	0.070	0	33.67	0	2.000
	Hill48	ABAQUS	REEF	0.020	4	32.88	2.62	0.022
		ABAQUS	REEF	0.070	4	34.34	2.65	0.025
		DD3IMP	UCoimbra	0.020	4	33.42	2.62	0.021
		DD3IMP	UCoimbra	0.070	4	35.07	2.69	0.031
		Lagamine	ULiege	0.082	4	34.15	2.53	0.011
		ABAQUS	UPorto	0.050	4	33.25	3.86	0.472
		LSDYNA	USiegen	0.075	4	34.41	2.67	0.028
		PAMSTAMP	USiegen	0.075	4	35.09	2.64	0.024
	Hill48 (NA)	ABAQUS	UAalto	0.010	4	33.96	2.39	**0.002**
		ABAQUS	UPorto	0.050	4	33.15	2.68	0.030
	Yld2004–18p	ABAQUS	POSTECH^(1)^	0.100	8	34.00	0.43	1.660
		LSDYNA	USiegen(POSTECH)^(2)^	0.075	4	34.22	7.15	4.498
		LSDYNA	USiegen(NTNU)^(3)^	0.075	12	32.97	0.52	4.599
		ABAQUS	NTNU(exp)^(4)^	0.090	8	34.44	0.43	1.662
	CB2001	DD3IMP	UCoimbra	0.020	4	33.14	3.82	0.449
	CPB06ex2	DD3IMP	UCoimbra	0.100	4	34.07	1.20	0.226
	Caz2018-Orth	ABAQUS	REEF	0.020	4	33.70	2.35	**0.001**
		DD3IMP	UCoimbra	0.020	4	33.54	2.42	**0.004**
		DD3IMP	UCoimbra	0.070	4	34.33	2.39	**0.002**
	HomPol4	MSC.MARC	USakarya(Iso)	0.100	4	32.94	1.71	0.065
		MSC.MARC	USakarya(Iso + KH)	0.100	4	33.20	2.02	0.015
		MSC.MARC	USakarya(Iso + m)^(5)^	0.100	8	33.45	0.78	1.436
		MSC.MARC	USakarya(Iso + KH + m)	0.100	4	34.16	2.04	0.012
	HomPol6	MSC.MARC	USakarya(Iso)^(1)^	0.100	8	33.09	1.70	1.067
		MSC.MARC	USakarya(Iso + KH)	0.100	4	33.28	2.15	**0.004**
		MSC.MARC	USakarya(Iso + m)^(1)^	0.100	8	34.98	1.20	1.227
		MSC.MARC	USakarya(Iso + KH + m)	0.100	4	34.29	2.16	**0.003**
Same + virtual tests	Yld2004–18p with DAMASK	ABAQUS	NTNU(CP)	0.090	4	34.06	0.74	0.458
	Caz2018singlecrys	ABAQUS	POSTECH^(1)^	0.100	8	34.49	3.93	1.512
		ABAQUS	REEF	0.020	4	33.97	5.61	2.103
CP	Caz2018polycrys	ABAQUS	REEF	0.020	4	33.93	2.61	0.020
	Minty	Lagamine	ULiege	0.082	4	31.63	3.35	0.220

(1) Minima at 0°, 45° and 90° and maxima at 22.5° and 77.5°.

(2) Minima at 0° and 90° and maxima at 45°.

(3) Minima at 15°, 45° and 75° and maxima at 0°, 30°, 60° and 90°.

(4) Minima at 22.5° and 77.5° and maximum at 0°, 45° and 90°.

(5) Minima at 30° and 60° and maximum at 0°, 45° and 90°.

**Table 36:** FE prediction synthesis and error vs. experimental values for models using a blank discretization by shell elements. Global error values lower than 0.01 are highlighted in bold

					Number of ears	Average height (mm)	Average amplitude (mm)	
	Experimental result				4	34.1	2.29	
	Yield criterion	Code	Team	μ	Number of ears	Average height (mm)	Average amplitude (mm)	Global error
Phenomenological models identified based on mechanical data	Hill48	LSDYNA	USiegen	0.075	4	33.56	3.06	0.113
		PAMSTAMP	USiegen	0.075	4	33.97	2.99	0.094
	Hill48 (NA)	PAMSTAMP	USiegen	0.075	4	32.63	5.65	2.157
	Yld89	LSDYNA	USakarya	0.100	4	33.42	2.84	0.058
	Yld2000-2D	LSDYNA	USakarya	0.100	4	32.53	0.83	0.409
		LSDYNA	USiegen^(2)^	0.075	8	32.51	0.97	1.337
		PAMSTAMP	USiegen	0.075	4	32.22	0.94	0.349
	HomPol4	LSDYNA	USakarya (Iso)^(1)^	0.100	8	32.11	0.44	1.660
		LSDYNA	USakarya (Iso + KH)	0.100	4	33.08	0.40	0.683
	HomPol6	LSDYNA	USakarya (Iso)^(2)^	0.100	8	31.94	0.74	1.462
		LSDYNA	USakarya (Iso + KH)^(3)^	0.100	12	33.09	0.90	4.369
Same + virtual tests	Yld2000-2D with VPSC	ABAQUS	UGent	0.070	4	30.59	3.31	0.208
	Facet-3D with ALAMEL	ABAQUS	KUL	0.090	4	34.15	2.26	**0.0001**

(1) Minima at 22.5° and 77.5° and maximum at 0°, 45° and 90°.

(2) Minima at 0°, 45° and 90° and maxima at 22.5° and 77.5°.

(3) Minima at 0°, 30°, 60° and 90° and maxima at 15°, 45° and 75°.

Regarding the number of ears, most models that adopt a 3-D orthotropic yield function predict 4 ears (see Fig. 119), as observed in the experimental results. Figure 120 shows that the average cup height is well predicted by all models, including the von Mises isotropic yield criterion. The fact that the Hill48, using solid elements, presents a higher amplitude of variation than most of the other models is related with the fact that it was selected by many teams, adopting different friction coefficient values and modelling strategies. Globally, the 2-D orthotropic yield functions (Yld89, Yld2000-2D, HomPoI4 and 6) tend to underestimate the average height, particularly when combined with shell elements. The average amplitude of the ear profile is presented in Fig. 121. It shows that 3-D orthotropic yield functions tend to predict values that are more accurate than 2-D orthotropic models, for which only shell elements can be used without additional assumptions (7 accurate average predictions out of 12 models for 3-D approach versus 4 reasonable predictions out of 8 shell models).

As previously mentioned, it has been reported that there is a correlation between the earing profile and the *r*-values and the yield stresses directionalities (e.g. see [13, 109]). Considering this assumption and based on the experimental data presented in “Material experimental characterization” Section, it is expected that all models that accurately predict the experimental *r*-values anisotropy will predict 4 ears, with maxima at RD and TD and minima at 45°. This is the case for the 3-D orthotropic yield functions of Hill48, CB2001, CPB06ex2 and Caz2018-Orth. Note also that all these 3-D orthotropic yield functions predict similar in-plane distributions for the *r*-values. Moreover, all these yield criteria predict a similar earing profile in agreement with the experimental data, in terms of both average height and locations of minima and maxima.

However, the results from each FE model that uses Yld2004–18p strongly varies:

- “Yld2004-18p – ABAQUS implicit - solid element (C3D8R) with NTNU implementation” predicts 12 ears (low amplitude) for the set of parameters based on physical experiments and 4 ears (medium amplitude) for the set based on crystal plasticity virtual experiments, see Fig. 106;
- “Yld2004-18p – ABAQUS explicit -solid element (C3D8R) with POSTECH implementation” predicts 8 ears (low amplitude), see Fig. 84;
- “Yld2004-18p - LS-DYNA explicit - solid element with the built-in implementation” used by USiegen predicts 4 ears with the data set of POSTECH (very high amplitude) and 12 with the set of parameters based on physical experiments from NTNU (very low amplitude), see Fig. 85.

Moreover, for some sets of parameters, the predicted locations of the minima and maxima of the cup height are contrary to the experimental observations (see Table 35). This volubility of the results points a very strong sensitivity to the implementation as well as to the identification strategy adopted to determine the anisotropy parameters. It reminds also that just relying on a correct prediction of the *r*-values and the yield stresses directionalities is far from being a secure approach, as all the implementations and sets of coefficients enable an accurate description of those experimental results. Finally, for this specific yield criterion, only the set of parameters identified based on virtual crystal plasticity results and the implementation of NTNU in ABAQUS generates an acceptable result (Fig. 106), although the earing amplitude is underestimated. As pointed by [42], the sensitivity of Yld2004–18p to the representative volume element of the texture (sheet central layer texture or sheet complete texture) used in DAMASK simulations could explain some systematic error. In addition, [55] confirmed for two different 6XXX series alloys (AA 6016 and AA 5182) that for crystal plasticity, the initial texture modelling has a larger impact than hardening assumptions (latent, direct, or isotropic in crystal plasticity).

The results obtained with Hill48, associated flow rule, isotropic hardening and solid elements reported in Table 35 enable an accurate prediction of an earing profile with 4 ears, with the same location experimentally observed. Most teams used the set of parameters provided by UCoimbra (formula of second line of Table 21 and values of line 1 of Table 22), based on Lankford coefficients at 0°, 45° and 90° like advised in many deep drawing simulation guidelines. The variations in the results come mostly from different friction coefficient values, contact algorithms, mesh refinements, time integration strategies (explicit or implicit) and code selection. These differences generate some non-negligible dispersion in the results: average height [32.88; 35.07] mm; and amplitude [2.53; 3.86] mm, compared to experimental mean values of 34.1 mm and 2.29 mm, respectively. Note that ULiege results provide the lowest average amplitude (closer to the experimental value), but a different set of parameters was used. The identification gave the same weight to the Lankford coefficients and the yield stresses in 7 directions, obtained from UA tensile tests (values of line 3 of Table 22). The results of REEF and UCoimbra for two different friction coefficient values confirm that friction has a non-negligible impact on the average height but a small one on the average amplitude. In this context, if the results from ULiege are removed from the analysis, as well as the ones from UPorto, the dispersion in the amplitude reduces to [2.62; 2.69] mm.

The choice of using a non-associative Hill48 model was done by 3 teams: USiegen, UAalto and UPorto. However, a comparison between their results is not straightforward. UPorto simulation works with a fixed gap between the blank holder and the die, which can affect the material flow. UPorto identification methodology is based on Eq. (71), assuming the *r*-values and yield stress evaluated from UA tensile tests (see Table 4 and Table 5, respectively), which clearly allows predicting the experimental profile of both the uniaxial tensile yield stress and the Lankford coefficients (Fig. 79). It predicts larger ear amplitude at 90° than in 0° (see Fig. 78).

UAalto model not only takes into account a non-associated flow rule but also textural hardening: *F*, *H*, *G* and *N* anisotropic parameters for both yield criterion and flow potential (Table 31) evolve with the plastic strain. This assumption is based on an accurate analysis of the data collected during the tensile tests in 7 directions, performed by UA, which can justify the evolution of the Lankford coefficients (see Fig. 5(c)) and yield stress with the plastic strain. Nevertheless, the model extrapolates the evolution beyond the maximum tensile strain measured in the tests (maximum value of 0.3 in Fig. 4), as shown for instance at 0.4 plastic strain in Fig. 66. The model “Hill48(NA) - ABAQUS explicit - solid element C3D8R - Voce hardening law - μ=0.01” results are very accurate, for both earing average height and amplitude, as the associated global error 0.002 is among the smallest ones (see Table 35).

Finally, USiegen tested PAMSTAMP non-associated Hill48 model and found some unexpected results, regarding the value predicted for the yield stress at 45°, which lead to a very large earing amplitude. Some interaction with PAMSTAMP staff is ongoing to better understand where this issue comes from.

Concerning the amplitude of the ears obtained with the 3-D orthotropic yield functions that predict the correct number of ears, let us discuss the earing profile simulated with the FE code DD3IMP, using the same FE model and different yield functions. It can be seen that CB2001 (Fig. 81) leads to an amplitude higher than Hill48 (Fig. 72) while the Caz2018-Orth (Fig. 91) presents an amplitude lower than Hill48 and closer to the experimental one. Finally, the CPB06ex2 (Fig. 88) presents the lowest amplitude for the earing profile. This may be correlated with the amplitude of the in-plane variation of the normalized yield stresses predicted by each model. On the other hand, Yld2004-18p predicts extremely well the anisotropy in yield stresses irrespective of the set of the anisotropy coefficients. However, the set of coefficients for which a 4 ears profile is predicted, the earing amplitude is either 0.74 (set identified by NTNU) or 7.15 (set provided by organizers-POSTECH), i.e. much higher than the one obtained with Hill48 or the experimental one (see Fig. 121).

The models that predict 8 ears (the 2-D orthotropic yield criteria HomPol4, HomPol6, Yld2000-2D) and the 3-D orthotropic yield criterion Yld2004–18p (POSTECH data set and NTNU based on physical experiments and simulations conducted with their own implementations in the FE code ABAQUS) have an earing amplitude much lower than Hill48 or the experimental one (see Fig. 121 or Tables 35 and 36). Note that all these yield criteria lead to a similar description of the in-plane distribution of the *r*-values.

The impact of using a pure isotropic hardening model (Swift) or mixed hardening (isotropic plus kinematic) model has been tested by USakarya, who uses HomPoI4 and HomPoI6 2-D orthotropic yield criteria, for both “LS-Dyna explicit - shell element” and “MSC.MARC implicit - solid element”. Whatever the FE model adopted, the hardening model has a negligible effect on the punch force prediction. However, it affects the wall average height, as for Swift model wrinkling occurs while it disappears with the use of mixed hardening. The selected hardening law has a medium effect on the earing profile (see Figs. 100, 101, 103 and 104). However, the latter is also strongly dependent on the type of element. The use of solid element (MSC.MARC) with an additive or multiplicative decomposition of the deformation gradient also modifies the earing profile.

Within the simulations considering a pure isotropic hardening model, the participants selected either Swift or Voce laws. As shown in “Identification of hardening laws and data used” Section, the predicted stress-strain curves (see Figs. 68) exhibit small variations, with a clear tendency to saturation with Voce law. This feature has a negligible effect on the stress-strain curve for the strain range present in tensile tests (maximum value of 0.25 at RD, in Fig. 4). On the other hand, it would justify an effect on the force prediction in deep drawing cup case, since strain values from 0.4 to 0.9 appear in the wall (Fig. 77). However, no FE model was tested strictly changing the hardening law by the participants, so it is not possible to quantify this effect neither on the force nor or on the earing profile.

Regarding the prediction of the evolution of the thickness along the cup circumference, at different heights, the results presented show that all FE models that predict 4 ears lead to similar trends, with maximum at 45° and minima at RD and TD. Note that the earing profiles obtained with the FE code DD3IMP using the same FE model but different yield functions and associated flow rule, show different amplitudes of thickness variation along the circumferential direction (see Hill48 in Fig. 74(e), CB2001 in Fig. 83, CPB06ex2 in Fig. 90 and Caz2018-Orth in Fig. 93(a)), for each height. This result highlights the influence of the shape of the yield locus on the thickness predictions.

The application of crystal plasticity models to conduct virtual tests that complement the physical test data, used for identification of the anisotropy parameters of phenomenological models, seems to enable not only a better qualitative prediction of the punch force, but also of the earing profiles (see grey rectangles in synthesis Figs. 119, 120 and 121). This affirmation is confirmed:

- for solid element (ABAQUS implicit - C3D8R), by the predicted number of ears (Fig. 119) and average height (Fig. 120), when using Yld2004–18 (with NTNU identification based on DAMASK simulations). Nevertheless, the earing amplitude is underestimated (Fig. 121) and, as previously pointed-out, this yield locus is very sensitive to the implementation.

-for shell element (ABAQUS implicit – S4R), by the predicted number of ears (Fig. 119) and earing amplitude (Fig. 121), when using Yld2000-2D (UGent identification based on VPSC simulations), but not by the average height (Fig. 120). Although all the results indicate that this yield criterion tends to underestimate the average height (see Fig. 97), the effect of choices such as, the low number of integration points through the thickness and the identification of anisotropic parameters based on virtual experiments for 0.1 strain could be investigated.

The ALAMEL crystal plasticity model used to identify the Facet-3D (ABAQUS explicit - SC8R continuum shell element) provides very interesting results in total agreement with both force and earing experimental results (see Figs. 117, 118, 119, 120 and 121 and the global error value).

If an ideal cube texture is assumed, although this specific orientation occurs only for 52% of the grains, the simulations using Caz2018singlecrys coupled with “ABAQUS C3D8R - solid element” and explicit (POSTECH) or implicit (REEF) strategies, predict 8 and 4 ears, respectively. Each team had its own identification procedure. Both simulations overestimate the earing amplitude and lead to a reasonable average height (with a higer accuracy for REEF approach).

The two polycrystal models that rely during the FE simulations on the single crystal behaviour (Minty and Cazacu2018polycrys) lead to a correct description of the *r*-values, number of ears (Fig. 119) and, for Cazacu2018polycrys, average height (Fig. 120) and earing amplitude (Fig. 121). For this latter model, the results, from the earing profile point of view belongs to the group of models providing results with a low global error (≤0.02: Hill48 Lagamine, Hill48(NA) ABAQUS, Caz2018-Orth ABAQUS and DD3IMP, HomPoI4 and HomPol6 MSC. Marc, Facet-3D ABAQUS, see Tables 35 and 36).

The fact that none of the FE models that use shell elements attains the threshold of 0.01 for the global error (see Eq. 72) can be related with the observation that such models cannot account for the occurrence of ironing. Regarding the FE models adopting solid or continuum-shell elements, three models present global error values lower than 0.002: the 3-D orthotropic yield functions Hill48(NA), using the identification strategy proposed by UAalto; and respectively, Caz2018-Orth, with the set of parameters provided by the organizers (REEF); and the Facet-3D model (identified with the approach of KUL) with a specific continuum-shell element. Nevertheless, it should be noted that some models might seem “penalized” because the respective teams using them did not seek to obtain the best possible prediction for the average cup height by fine-tuning the friction coefficient. The fact that different models can lead to similar results seems to highlight the robustness of the respective orthotropic yield functions and the experience of the analysts.

## Conclusion

The finite element simulation of the cup drawing process of an AA6016-T4 aluminum alloy sheet sample, proposed in the ESAFORM benchmark 2021, has generated a huge collaborative work. Based on the result analysis, the following points are highlighted:

- No information was provided by the organizers regarding the friction value, except through the experimental evolution of the punch force during the cup drawing and the earing profile. It is a challenge for phenomenological models using a constant friction value, to predict both drawing and ironing forces (present in the experiment), while respecting the imposed clearance between the die and the punch. A close agreement with the experimental results for the force signal is difficult, but possible, for simulations relying on solid elements. However, it is nearly impossible for the simulations using shell elements. Use of crystal plasticity computations to complement physical tests in the identification of the parameters of phenomenological models may contribute to improve the prediction of the ironing force.
- With the same set of experiments for the material parameter identification, trained scientists using the well-known Hill48 model, but different codes, meshes and element types have predicted quite similar earing profiles. This observation inspires confidence in the robustness of the FE implementation of this model in various codes. Seven participants out of eight have a global error for the earing profile smaller than 3% for this strong cube texture material. This observation shows that for this aluminium alloy, this criterion can lead to adequate predictions of the earing profile.
- Trying to enhance the FE prediction accuracy, numerous phenomenological orthotropic yield loci have been applied. Out of all these models, the 3-D Hill48 model with a non-associated flow rule and anisotropy parameters that evolve with the plastic strain [63] and the 3-D Caz2018-Ortho model [15], which uses an associated flow rule and constant anisotropy parameters were highlighted. Both models predict earing profiles with less than 0.5% error.
- The 3-D orthotropic yield criteria which rely on experimental tensile yield stresses and Lankford coefficients in 7 orientations in their identification procedure are: Yld2004–18p, CPB06ex2, CB2001, and Caz2018-Orth. All these criteria predict with high accuracy the shape of the yield locus in the tension-tension quadrant as well as the plastic flow direction. About the anisotropy in yield stresses, compared to Hill48 and Caz2018-Orth yield criteria, CB2001 presents a small improvement, while Yld 2004–18p, and CPB06ex2 predict a better accuracy. Except Yld 2004–18p that predicts an earing profile with 4, 8 or 12 ears according its identification and implementation, the other criteria predict 4 ears, as observed experimentally. While CB2001 and CPB06ex2 have accuracy levels in the earing profile close to models that involve fewer parameters, Caz2018-Orth leads to a clear improvement in the predictions, with maximum and minimum cup height within 1% of the experimental values.
- It is to be noted that, within the parameter identification methodology, particular relevance was given by the participants to the description of the anisotropy of the Lankford coefficients, since it is known that it has a strong impact on the trends of the earing profile. This also highlights the importance of accurate experiments to measure Lankford values.
- No in-depth investigation about the choice of the hardening model has been performed. However, it appears that for the level of deformation reached in this cup drawing, both Swift and Voce isotropic laws can predict reliable results. Nevertheless, the interest of a mixed hardening approach is pointed by the HomPoI4 and HomPol6 simulations.
- If 250 crystals, representative of the texture of the material, are used as input, simulations with Caz2018polycrys model, which uses Caz2018singlecrys for the description of the single crystal behaviour, lead to a good description of the material behaviour (tensile and shear tests, as well as the earing profile). However, a careful identification process of the five anisotropy parameters of Caz2018singlecrys model in three steps is applied, exploiting the seven uniaxial tests in directions 0°, 15°, 30°, 45°, 60°, 75°, and 90°.
- The Facet-3D yield locus is a very flexible function. For the benchmark material, 38 terms and a total of 154 non-zero coefficients are needed whereas for other systems with different initial textures, the total number of non-zero coefficients could be different. The most critical requirement in the identification methodology proved to be the selection of the 10,000 discrete crystal orientations for the virtual experiments.
- The interest of using crystal plasticity to enhance the accuracy of the identification of a phenomenological model is a common idea. ALAMEL and Facet-3D cases confirm this approach but the use of VPSC model to identify Yld2000-2D criterion or DAMASK approach to identify Yld2004–18p yield locus are not so convincing. However, for Yld2000-2D law, the element choice, number of integration points through the thickness and the low weight of the yield stress anisotropy in the identification process could explain the low accuracy. For Yld2004–18p constitutive model, the sensitivity of the results to its implementation has been clearly pointed.
- The very basic Minty interpolation law with a set of 1000 representative crystals and a simple full Taylor assumption can describe all the trends in the earing profile but, probably at the cost of a higher CPU time for the deep drawing simulations than Facet-3D and Caz2018polycrys laws. Some issues in the predicted punch force evolution also suggest the need for further investigations.
- The identification methodology is a key point to generate reliable results. This article highlights how the careful parameter selection approach of some modelers led to accurate results. This also points out that this identification work request skilled scientists. The choice of a representative set of crystals, the analysis of Lankford coefficient evolution or not and, the need of pre-validation checks before performing the cup drawing simulations, require a larger training than applying simple analytical formula to identify Hill48 model, from constant Lankford coefficients in three directions.
- Looking at the benchmark from another point of view than the capacity to predict earing profile or force evolution, one could see six types of data. Tensile flow stress anisotropy, *r*-value anisotropy, yield locus (biaxial tests), earing profile, force evolution in cup forming, monotonic and reverse shear tests are available. It has been highlighted that none of the models could accurately describe the complete picture. For instance, the yield stress anisotropy under uniaxial loadings was not well predicted, particularly the one at 45°, by most of the models (including the ones based on crystal plasticity). Although, the very accurate description of the anisotropy of uniaxial yield stresses is not critical for the correct prediction of the earing profile, it is certainly relevant for other processes.
- The strong collaboration between experimental and numerical teams prevents the easy assumption, common for simulation teams, to just think that there is a problem in the experiments. Here, the interaction between the teams allowed fruitful discussions about clearance, defects in the blank positioning and measurements. The need for further investigation in tool deformation, contact modelling and friction measurements is itself a result from the benchmark.

CPU time is only presented in Table 33 and discussed in terms of exploiting polycrystalline models either in the cup drawing simulations or in the virtual predictions used to improve the identification of the optimal set of parameters for a phenomenological law. Two reasons justify this choice.

The first one is the huge discrepancy in the meshes used to discretize the blank, from 1.344 (POSTECH), 2.128 (UPorto) to 55.560 elements (USiegen). This variation makes it impossible to assess the specific cost of a constitutive model. Choosing the mesh refinement is part of the modeller expertise and can, indeed, affect the results accuracy. However, it is the responsibility of each FE user to perform a mesh convergence check.

The second reason is related with the fact that today, as pointed in the introduction, well-trained surrogate models can solve CPU issues and so the first priority is to know which models are the reliable ones.

This benchmark was also an opportunity to discover some issues related to the FE codes or their use. For instance, ULiege simulations using Lagamine present a non-physical drop in the punch force evolution; while the implementation of Hill48(NA) in the commercial code PAMSTAMP with shell elements leads to incoherent results, e.g. the FE prediction of the yield stress at 45° orientation is different from the experimental value. These difficulties highlight the importance of validating the FE models with simple tests or cup simulations and suggest the need for further investigations.

Last but not least, any scientist interested to work on this benchmark can contact Gabriela Vincze (gvincze@ua.pt) who will provide the data. Suggestions to ESAFORM board (current president livan.fratini@unipa.it) for an efficient platform of data exchange are welcome as the board has still to decide the best solution to share ESAFORM 2021 benchmark data and future ones.

Do not hesitate to propose a benchmark in the following years (see the conditions on https://esaform.org/grants/).
